# Impact of pulmonary rehabilitation programme design on effectiveness in COPD: a systematic review and component network meta-analysis

**DOI:** 10.1016/j.eclinm.2025.103433

**Published:** 2025-08-20

**Authors:** Thomas J.C. Ward, Lorna Latimer, Enya Daynes, Suzanne C. Freeman, Sarah Ward, Jiaqing Xu, Muhammed Haris, Majda Bakali, Sophie Reap, Mamoon Iqbal, Lin Wang, Akash Mavilakandy, Aarinola Olaiya, Hnin Aung, Theresa C. Harvey-Dunstan, Sally J. Singh, Neil J. Greening, Rachael A. Evans, Michael C. Steiner, Alex J. Sutton

**Affiliations:** a Department of Respiratory Sciences, University of Leicester, Leicester, UK; b Department of Respiratory Medicine, University Hospitals of Leicester, Leicester, UK; c Institute for Lung Health, National Institute for Health Research Leicester Biomedical Research Centre – Respiratory and Infectious Disease, Glenfield Hospital, Leicester, UK; d Faculty of Medicine and Health Science, University of Nottingham, Nottingham, UK; e School of Physiology, Pharmacology & Neuroscience, University of Bristol, Bristol, UK; f Department of Population Health Sciences, University of Leicester, Leicester, UK

**Keywords:** Pulmonary rehabilitation, Exercise training, Chronic respiratory disease

## Abstract

**Background:**

Pulmonary rehabilitation (PR) is a key treatment for chronic obstructive pulmonary disease (COPD) recommended by all guidelines. However, programmes vary widely and the optimal combination of components to maximise benefits and efficiency remains unknown. We aimed to use the novel technique of component network meta-analysis (cNMA) to investigate the relative contribution of 1) exercise modality and intensity, 2) non-exercise components, 3) type of supervision, and 4) programme duration of PR for people with COPD.

**Methods:**

MEDLINE, EMBASE, CINAHL, and Cochrane databases searched in October 2023 with no date or language restrictions. We included randomised controlled trials (RCTs) which included an intervention involving exercise for people with COPD. We present outcomes of exercise capacity, breathlessness and health related quality of life (HRQoL). Screening and eligibility were assessed by two independent reviewers. cNMA, a technique developed to investigate complex interventions such as PR, was conducted to examine the contribution of single components within diverse multicomponent interventions controlling for cohort demographics. PROSPERO: CRD42022322058.

**Findings:**

We included 337 RCTs with 18,911 participants and 227 intervention components. In-person supervision enhanced gains in exercise capacity (Standardised mean difference (SMD) 0.41, 95% CrI 0.20; 0.63), HRQoL (0.43 95% CrI 0.19; 0.68) and breathlessness (0.31 95% CrI 0.04; 0.58) over exercise training alone with moderate to high certainty. Remote supervision increased gains in exercise capacity (0.40 95% CrI 0.08; 0.73) with trends towards improvements in HRQoL and breathlessness, with low certainty. Aerobic training appeared to be most effective for all outcomes at high or very high intensity but with low certainty. Addition of structured education did not improve any outcome. Psychological interventions led improvements in exercise capacity (0.37 95% CrI 0.01; 0.73, low certainty) and HRQoL (0.54 95% CrI 0.18; 0.91, moderate certainty). There was trend towards improvements in breathlessness with addition of breathing exercises (0.26 95% CrI −0.04; 0.56, low certainty). Programme duration did not impact outcomes. For outcomes of exercise capacity, HRQoL and breathlessness there were 60%, 63% and 59% studies at high risk of bias respectively.

**Interpretation:**

This large-scale analysis of over 300 randomised PR trials found the strongest effects for in-person supervised and prescribed aerobic exercise training with less certainty for the benefit of other commonly used PR components and delivery methods.

**Funding:**

This research was funded through a National Institute for Health and Care Research (NIHR) Applied Research Collaboration East Midlands grant (2.12) and carried out at the 10.13039/501100020013NIHR Leicester Biomedical Research Centre (BRC).

**Research in context**

**Evidence before this study**

Although there is strong evidence supporting the effectiveness of pulmonary rehabilitation (PR), the specific components of these programmes vary widely. The optimal combination of components to maximise benefits and efficiency is still unknown, and there is no international consensus on the design of PR programmes. A literature search conducted in March 2022 indicated that previous meta-analyses have combined various PR interventions into a single ‘package’ compared to usual care, but they have not simultaneously considered the interactions between programme components, supervision, and patient factors.

**Added value of this study**

This large systematic review and component network meta-analysis of 337 randomised controlled trials of PR with nearly 19,000 people with COPD, found the strongest effects were for in-person supervised (Standardised mean difference 0.41, 95% CrI 0.20; 0.63) and prescribed aerobic exercise training with less certainty for the benefit of other commonly used PR components and delivery methods.

**Implications of all the available evidence**

This systematic review and component network meta-analysis implies that pulmonary rehabilitation programmes for people with COPD should include supervised and prescribed aerobic exercise training as a minimum for the greatest improvements in three core outcomes. Future multi-centre prospective randomised trials should investigate the effects of other commonly used PR components.

## Introduction

Pulmonary rehabilitation (PR) is an exercise-based intervention for people with COPD which is recommended by all international guidelines due to unequivocal evidence of effectiveness in improving exercise capacity, breathlessness and health related quality of life (HRQoL) for people with COPD.^1^ The evidence to support this guidance is summarised in the 2015 Cochrane systematic review, which defined PR as “Any … programme of at least four weeks' duration that included exercise therapy with or without any form of education and/or psychological support”.^1^ This review concluded that further clinical trials assessing PR effectiveness were unnecessary and that “future research studies should focus on identifying which components of PR are essential, its ideal length and location, the degree of supervision and intensity of training required”.^1^ Since the conclusion of the Cochrane review numerous trials have investigated different aspects of PR design with interest in home-based PR, in particular, accelerating since the COVID-19 pandemic.

Whilst exercise training is widely accepted as the cornerstone of PR, questions remain about the delivery and intensity of exercise therapy and the effectiveness of other frequently included components. This is largely because PR seeks to provide holistic care addressing a variety of patient needs through a complex tailored intervention and therefore the individual components of PR are rarely tested in isolation. The result of this uncertainly is wide variation of the make-up of PR programmes and lack of consensus about the optimum design of the intervention reflected in the recent American Thoracic Society (ATS) workshop report that lists many commonly used PR components as desirable rather than essential due to lack of strong evidence.^2^ Robust evidence on the effectiveness of different PR components would benefit those developing, delivering, and commissioning clinical services, enhancing patient outcomes.

Previous meta-analyses of PR have pooled interventions of varying designs into a “package” of PR compared with usual care.^1^^,^^3^ Component network meta-analysis (cNMA) allows investigation of the unique contribution of single components within multicomponent interventions that are rarely tested in isolation. cNMA has been used to investigate multicomponent interventions such as smoking cessation interventions^4^ and interventions for early psychosis.^5^ cNMA is well suited to investigate complex interventions such as PR, for which there are a multitude of randomised controlled trials with differing intervention designs allowing us to simultaneously consider interactions between programme components, supervision and patient factors.

Our aim was to quantify the contribution of components of PR design on the most commonly measured short term core outcomes of exercise capacity, breathlessness and health related quality of life (HRQoL) in COPD. We investigated the following factors: 1) exercise modality and intensity, 2) non-exercise components, 3) type of supervision, and 4) programme duration. Furthermore, we investigated whether component effectiveness was affected by patient related factors including disease severity.

## Methods

The systematic review was prospectively registered on PROSPERO (CRD42022322058) and followed the PRISMA network meta-analysis reporting guidelines. A literature search of MEDLINE, EMBASE, CINAHL, and Cochrane databases was performed in March 2022 and updated in October 2023. A further updated search was performed in May 2025 prior to publication but studies identified from this search have not been included in the analysis to prevent further delays to publication. Search terms encompassed lung disease, exercise/rehabilitation and randomised controlled trials with no date or language restrictions (full search terms in Methods Supplement).

### Ethics

This study used publicly available data and no ethical approval was required.

### Eligibility criteria

We adopted a data-driven approach by including a diverse range of intervention types in our analysis that included the very minimum of what might be considered PR; an intervention for people with respiratory disease that included exercise (the definition used in the 2015 Cochrane review of PR). This method allows us to objectively estimate the effects of each component without any preconceived biases about which components should be part of PR.

To be eligible a study had to satisfy the following five criteria: 1) diagnosis of COPD in at least 80% of participants, 2) an intervention that included as a minimum an exercise intervention of at least twice weekly for a minimum of 3 weeks (based on the Cochrane definition,^1^ duration reduced to 3 weeks to encompass inpatient programmes and frequency increased to twice a week to exclude long term maintenance programmes), 3) a second intervention arm meeting the criteria above or no intervention/‘usual care’, 4) at least one of the outcomes listed in the ATS PR workshop report measured using any appropriate, validated tool^2^ (listed below), and 5) a randomised controlled trial. We excluded studies of participants within 4 weeks of an acute exacerbation, studies with insufficient description of intervention to allow classification, studies published in abstract form only, studies focused solely on respiratory muscle training and interventions relevant to a small subgroup of patients (likely to be less than 60% of screened individuals) with a specific characteristic in which the intervention is likely to have a differential effect to those without that characteristic (e.g., use of NIV in hypercapnic patients).

### Outcomes

This paper presents the results of the primary outcomes; exercise capacity, breathlessness and HRQoL. These outcomes were chosen as they are core outcomes of PR programmes^6^ and are the most frequently measured. Secondary outcomes (quadriceps strength, knowledge, patient activation, self-efficacy, adherence, completion, frailty, anxiety, depression, hospitalisation, mortality, cognitive status, fatigue, physical activity, activities of daily living, coping skills, inspiratory muscle strength, and sleep disturbance) will be reported separately. No limit was set on instruments used for each outcome. If a study reported more than one measures for an outcome (e.g., 6-minute walk test and an incremental cycle test), we chose an outcome for the analysis based on a predetermined hierarchy with incremental exercise tests favoured, followed by endurance then self-paced tests. For HRQoL we prioritised the Chronic Respiratory Questionnaire (CRQ), then the St. George's Respiratory Questionnaire, the COPD Assessment Test followed by any other HRQoL questionnaires. For breathlessness, we prioritised the CRQ dyspnoea domain then the Medical Research Council dyspnoea scale followed by other breathlessness measures.

### Study selection and data extraction

Titles and abstracts and subsequently full texts were independently screened by two reviewers with disagreements being settled by consensus or involvement of a third reviewer. Study selection was performed in the online software Rayyan.^7^ Data extraction was performed by a single reviewer (TW) with a subset of 25% checked by a second reviewer. Component coding was extracted by a single reviewer with all codes checked by a second reviewer. Disagreements were settled by consensus or involvement of a third reviewer.

The following data were extracted for each intervention arm in included studies: outcome measure (at baseline, following the intervention, mean change and standard deviation of the change), number of participants, programme design (including location, components and delivery), programme duration and frequency, description of the exercise component, adherence and completion rates, whether an intention to treat analysis was performed, participant demographics (age, gender, height, weight, BMI, GOLD stage, FEV1 (in L and %pred), ethnicity, sexual orientation, marital status, religion, socioeconomic status), proportion of participants diagnosed with COPD, country and whether a practice exercise test was performed.

### Defining intervention components

Initial components were coded in as much detail as possible (for example, including the intensity, modality, supervision, and muscle groups trained for exercise components) and later combined with similar component definitions for analysis. The predominant mode of supervision was coded for each intervention arm which must have been delivered at least once weekly to be considered. Intensity of aerobic training was decided using a predetermined guide (Table 1)^11^ and was based on the lower target intensity for studies that reported a range in intensity prescription. For ‘optional’ components that were delivered to a subset of participants, an attempt was first made to code at a higher level or if not possible, assumed to have been given to half of participants (and given a value of 0.5 in the analysis). A preplanned sensitivity analysis was run excluding optional components.

**Table 1:** Classifying intensity for aerobic training intensity.

	Low intensity (1)	Moderate intensity (2)	High intensity (3)	Very high intensity (4)	Guideline
Borg, dyspnoea	<3	3	4–6	>6	ATS/ERS^8^/ACSM^9^
W_peak_	<40%	40–59%	60–79%	≥80%	ACSM for COPD
Walking speed (%max)	<40%	40–59%	60–79%	≥80%	ACSM for COPD
Walking speed from 6MWT	<80%	–	≥80%	–	^10^
%max heart rate	<64%	64–76%	77–95%	≥96%	ACSM guidelines for healthy adults
% heart rate reserve	<40%	40–59%	60–89%	≥90%	ACSM guidelines for healthy adults
% age predicted max heart rate	<64%	64–76%	77–95%	≥96%	No guidelines, based on ACSM HR guidelines
%V̇O_2peak_	<45%	46–63%	64–90%	≥91%	ACSM guidelines for healthy adults
At anaerobic threshold			All		
Self-selected intensity	All				
Borg RPE (6–20 scale)	<10	10–11	12–14	>14	ATS/ERS, 2013
Borg RPE (CR10 scale)	<3	3	4–6	>6	ATS/ERS, 2013

W_peak_: peak workload during incremental cycle ergometry test; 6MWT: 6-minute walk test; V̇O_2peak_: oxygen uptake at peak exercise; RPE: rating of perceived exertion.

Remote supervision was defined as contact with a rehabilitation provider or a member of the study team via telephone, smartphone, website or teleconferencing at least once week for the duration of the intervention.

### Statistics

We conducted random effects Bayesian component network meta-analyses for each outcome using WinBUGS version 14 interfaced using R v4.3.2 & RStudio^12^ (model code in Supplement). We used the standardised mean difference to combine outcomes measured using different instruments and present 95% credible intervals (95% CrI). Standardised mean difference (SMD) can be interpreted as equivalent to effect size with cut offs of 0.2 for small effects, 0.5 for moderate effects and 0.8 for large effects.^13^ For breathlessness and HRQoL outcomes, we converted to a standardised scale so that a higher score was better for all measures. To aid understanding, SMDs were also presented after conversion to the most common measure for each outcome by multiplying by the pooled baseline standard deviation as recommended by the Cochrane Handbook for Systematic Reviews.^14^

Due to the complexity and magnitude of the analysis for each outcome, we first developed simplified models designed to test the feasibility of the modelling as a sequential step towards more complex modelling. For the interim models we combined similar components into the fewest number possible which included exercise training as a single component. The assumption of additivity was then relaxed by considering a model in which meaningful interactions between components were added. We then created more complex final models designed to address our study aims focussing on intensity for aerobic exercise components and the limbs trained for strength training components which assumed component effects were additive (full list of components in Results Supplement, Supplementary Table S17). For the final model, the following changes to the interim model were made: 1) the exercise component was split focussing on intensity for aerobic exercise components and the limbs trained for strength training components and 2) breathing retraining and inspiratory muscle training were split into separate components. Decisions about combining and splitting components were made during meetings with the wider study team with expertise in PR.

Our initial aim had been to investigate the effect of both PR location and supervision. Supervision and location were strongly correlated and therefore we could not investigate both in the same analysis. Our review steering team (which included clinical experts in PR) felt that the more important question for clinical audiences was supervision rather than location. Supervision was collected prospectively and included in our statistical analysis plan prior to analysis.

### Methods to deal with missing data and identifying influential cases

For missing data, we first attempted to contact authors of included studies to provide this information. If this was not possible, we imputed data where appropriate. For missing data on outcome variances, methods suggested in the Cochrane handbook^14^ to estimate standard deviation from other reported measures were used. If IQR was reported, SD was estimated using the methods of Wan et al. 2014.^15^ A correlation coefficient was estimated from the data to estimate change standard deviation from baseline and follow up standard deviation. For covariate missing data, multiple imputation was performed up to 20%. If covariates had more than 20% missing data, these were excluded from the analysis. For FEV_1_% predicted this was first estimated using linear regression from FEV_1_ in litres if reported. For all other covariates, multiple imputation was performed within WinBugs (code provided in Supplement). Plots of residual deviance and leverage plots were used to identify influential cases. If cases were influential due to implausibly small reported standard deviation of the outcome, these were assumed to be incorrectly reported standard errors and therefore multiplied by the square root of the sample size before the analysis was re-run. Whilst no methods exist to test inconsistency in cNMA, model fit was assessed through monitoring of deviance information criterion (DIC), tau and total residual deviance.

As the components of hypertonic saline and normal saline were included in opposing arms of a single trial, their effects could not be estimated, and these components were removed from the analysis.

### Covariate models

We investigated the effect of the following covariates in our models: mean cohort FEV_1_ (%predicted), age, sex (proportion male as a continuous variable), publication year, income status of country (World Bank definitions^3^), programme duration (weeks), and outcome measure at baseline. We intended to also assess for the impact of mean cohort BMI, adherence and completion. However, these variables were reported in less than 80% of included studies and therefore not investigated further. Covariates were assumed to have separate (independent) effects on components. We performed an additional analysis to explore the effect of programme dose by including the total number of sessions and the total “dose” (number of sessions × duration of session) as an arm level covariate assumed to have a common (interchangeable) effect on components.

### Sensitivity analyses

We performed the following predefined sensitivity analyses: 1) removing studies with high risk of bias, 2) exclusion of studies with high risk of bias in measurement of the outcome, 3) use of a conservative correlation coefficient of 0.5 for imputed standard deviation, 4) removal of studies for which data have been imputed, 5) removal of studies with low quality of intervention reporting (TIDieR checklist score < 8, see below), 6) exclusion of studies in which a proportion of patients did not have COPD, 7) removal of studies without a practice exercise test (for outcome of exercise capacity), 8) removal of studies using endurance and self-paced exercise tests outcomes (for outcome of exercise capacity) to explore whether variation in outcome measures influences the results, and 9) exclusion of components described as optional in original PR programme description.

### Risk of bias and certainty of evidence

Each study was assessed for risk of bias based on the Cochrane risk of bias 2 (RoB2) tool^16^ and certainty of evidence assessed using the GRADE framework (Gradepro.org) by two independent assessors with any disagreements settled by consensus. The quality of reporting of the intervention was assessed using the TIDieR checklist. We present funnel plots to assess asymmetry for comparisons in the interim model for which there were at least 10 studies included in the analysis.^17^

### Role of funding source

The funder of the study had no role in study design, data collection, data analysis, data interpretation, or writing of the report.

## Results

**Fig. 1:**
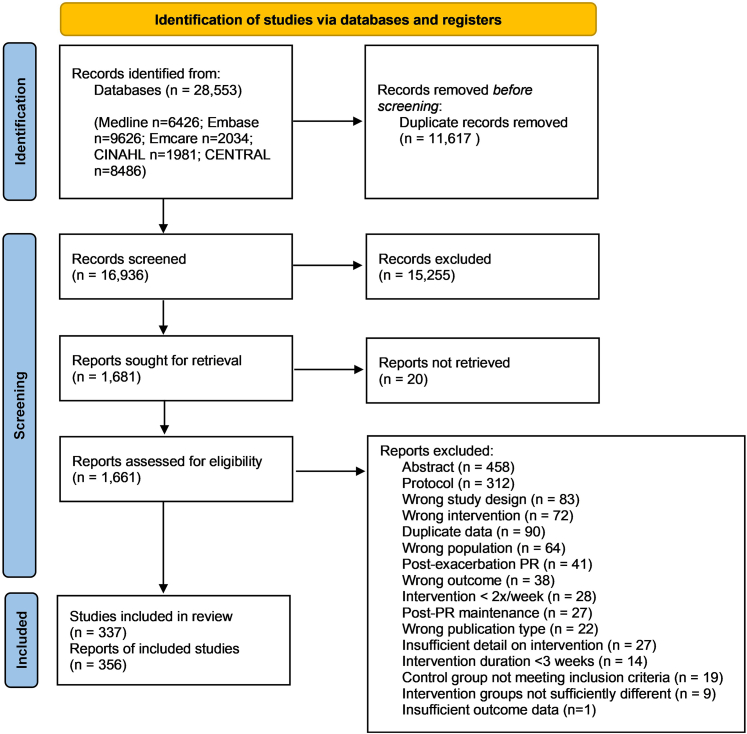
Preferred Reporting Items for Systematic Reviews and Meta-analyses (PRISMA) diagram of study selection. CINAHL: Cumulative Index to Nursing and Allied Health Literature; CENTRAL: Cochrane Central Register of Controlled Trials; PR: Pulmonary rehabilitation.

Literature searches identified 28,553 references of which 356 papers were included, comprising 337 individual randomised controlled trials (Fig. 1) with a total of 18,911 randomised participants. An updated literature search prior to publication identified 2059 further references of which 35 papers met the inclusion criteria (Supplementary Figure S84). These papers are listed in Supplementary Table S18 but are not included in the meta-analysis models. Exercise capacity was reported in 313 studies (93%), HRQoL in 224 studies (66%), and breathlessness in 159 studies (47%). Studies were conducted across 46 countries with 228 (67%) conducted in high income countries, 88 (26%) in high middle-income countries, 22 (7%) in low-middle income countries and none in low-income countries.

In total, 227 individual components were investigated in the included studies (Results Supplement, Supplementary Table S17). The number of individuals in each study intervention ranged from 4 to 281 participants with a mean of 26. The location of the intervention was in an outpatient setting for 462 arms (n = 10,905), inpatient for 70 arms (n = 2930), and at home for 97 arms (n = 3843).

**Fig. 2:**
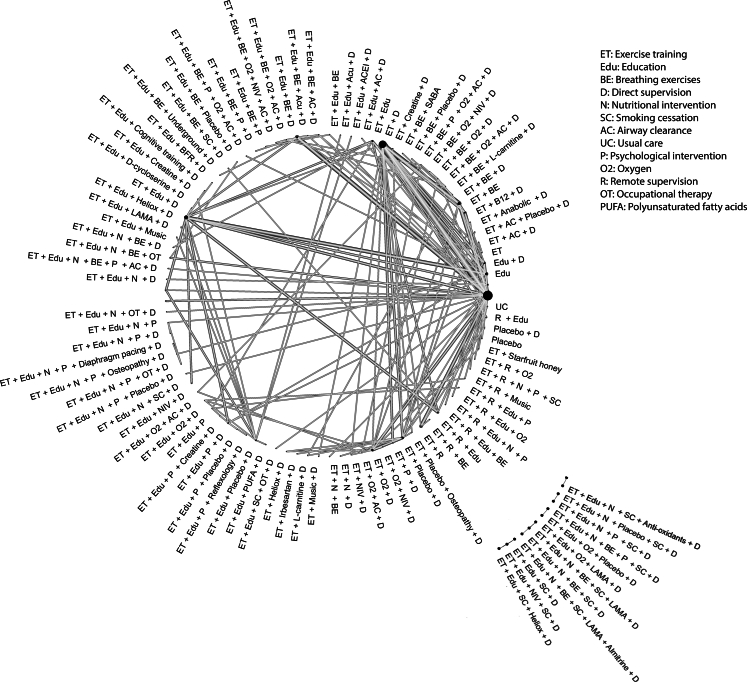
Network plot of studies reporting exercise capacity as an outcome in the interim model. Each node represents an intervention (composed of components) in the included papers and each line represents comparisons within included randomised controlled trials. The size of the nodes is proportional to the number of studies including this intervention and the weight of the lines is proportional to the number of comparisons within the included studies. This figure is included for a visualisation of the data structure however as our analysis is component level, the data is not analysed at treatment node level.

The simplified interim models which combined 227 components into 36 pooled components (with exercise training as a single component), confirmed the viability of our model fitting approach with exercise training regardless of supervision leading to moderate improvements in exercise capacity (SMD 0.56 95% CrI 0.38; 0.73 high certainty, Supplementary Figure S18), HRQoL (SMD 0.72 CrI 0.50; 0.93 high certainty, Supplementary Figure S19), and breathlessness (SMD 0.54 95% CrI 0.32; 0.76, low certainty, Supplementary Figure S20). A network plot demonstrating the structure of the network for the outcome of exercise capacity is presented in Fig. 2. We attempted to add interaction effects to the additive models but were unable to estimate any of these interactions precisely (see Results Supplement).

**Fig. 3:**
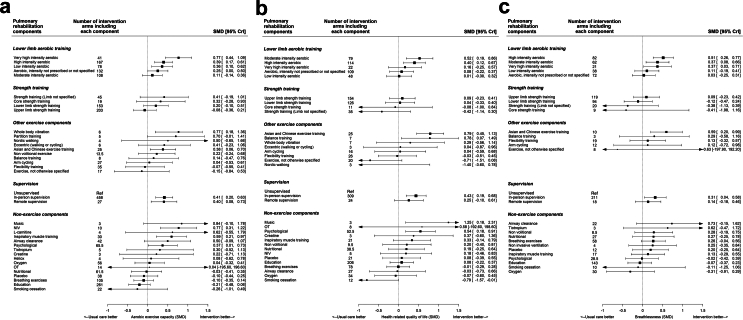
Final additive component network meta-analysis models for outcomes of A) exercise capacity, B) health related quality of life, and C) breathlessness. SMD: standardised mean difference; CrI: credible interval; OT: occupational therapy; NIV: non-invasive ventilation.

The final models combined exercise components into 29 separate components based on the intensity of aerobic training components (very high, high, moderate, low) and the body areas trained for strength training components (upper, lower, trunk) based on expert consensus (Fig. 3 and Table 2). In-person supervision enhanced gains in exercise capacity (SMD 0.41, 95% CrI 0.20; 0.63), HRQoL (SMD 0.43 95% CrI 0.19; 0.68), and breathlessness (SMD 0.31 95% CrI 0.04; 0.58) over exercise training alone with moderate to high certainty of evidence. Remote supervision increased gains in exercise capacity (SMD 0.40 95% CrI 0.08; 0.73) with trends towards improvements in HRQoL and breathlessness over exercise training alone, with low/very low certainty of evidence.

**Table 2:** Summary of component effects heat map with certainty of evidence from GRADE assessment.

Results obtained from final component network meta-analysis models in which the effects of single components were assumed to be additive. Therefore, the expected effect of a single intervention is assumed to be the sum of the effects of the components within that intervention. Dark green indicates significant positive effect, light green indicates trend towards positive effect, light red indicates trend towards antagonistic effect. A Bayesian 95% CrI means there is a 95% probability that the true estimate would lie within the interval (as opposed to a frequentist 95% CI for which if we repeated the research, we would expect the new result to be contained within this interval 95% of the time). To aid interpretation, we have converted to most commonly reported measure for each outcome by multiplying by pooled baseline standard deviation for that outcome.

CrI: Credible interval; 6MWD: 6 minute walk distance, minimal important difference (MID) 30 m; SGRQ: St George's Respiratory Questionnaire, MID 4 points; CRQ dyspnoea domain: Chronic respiratory disease questionnaire dyspnoea domain, MID 0.5 points.

There were trends towards improvements in exercise capacity with the addition of lower limb strength training to a programme (SMD 0.20 95% CrI −0.10; 0.51). Addition of structured education to a programme did not improve HRQoL (SMD 0.08 95% CrI −0.22; 0.37), breathlessness (SMD −0.07 95% CrI −0.37; 0.23) or exercise capacity (SMD −0.21 95% CrI −0.48; 0.06) (low to moderate certainty). Addition of psychological interventions led to improvements in exercise capacity (SMD 0.37 95% CrI 0.01; 0.73) and HRQoL (SMD 0.54 95% CrI 0.18; 0.91) but not breathlessness (low to moderate certainty). There was trend towards improvements in breathlessness with addition of breathing exercises improved breathlessness (SMD 0.26 95% CrI −0.04; 0.56, low certainty) but not other outcomes. Addition of inspiratory muscle training led to improvements in exercise capacity (SMD 0.59 95% CrI 0.21; 0.97) with trends towards improvements in HRQoL (SMD 0.33 95% CrI −0.14; 0.79) but not breathlessness (low to moderate certainty). Nutritional interventions had no impact on outcomes (very low to moderate certainty). Chinese and Asian exercise modalities improved all outcomes (moderate to high certainty).

Very high intensity aerobic exercise prescription resulted in the greatest gains in exercise capacity, with moderate intensity aerobic training the least effective (low certainty) whereas moderate and high intensity aerobic training were the most effective for improvements in HRQoL and breathlessness respectively.

For outcomes of exercise capacity, HRQoL and breathlessness there were 60%, 63%, and 59% at high risk of bias respectively (Supplementary Figures S51–S56). Mean quality score using the TIDieR checklist was 8.3 out of 12 with a quality score less than 8 in 109 out of 337 studies. Results of multiple sensitivity analyses are listed in the Results Supplement (Supplementary Figures S57–S80) and were considered in the GRADE assessment, for example downgrading of certainty for results deemed to be at high risk of being influenced by bias (see footnotes of GRADE assessment tables). Funnel plots are presented (Supplementary Figures S81–S83) for comparisons for which there were at least 10 studies included in the review. However, as this represents 4%, 3%, and 2% of included comparisons for the outcomes of exercise, capacity, HRQoL and breathlessness respectively we do not feel it is possible to make conclusions about the possibility of publication bias. Adherence to the intervention was reported in 36% of studies. Completion rates were reported in 80% of included studies however the definition used to define completion was explicitly stated in only 29% of studies.

Effects of participant related covariates (age, sex, FEV_1_ and baseline outcome measure) are shown in Supplementary Table S2. Controlling for covariate effects by centring of models for the mean of each covariate had minimal effect on interpretation of component effects (Supplementary Figures S24–S50) and no meaningful effect on model fit (Supplementary Table S3) indicating that our results can be applied to typical PR cohorts. However, some components appeared to be marginally more effective in some cohorts for some outcomes. Exercise training was less effective for improvements in breathlessness and HRQoL in cohorts of higher mean age and the higher the proportion of females in a cohort. Remote supervision more effective for exercise capacity in male cohorts. In-person supervision was possibly more effective for improvements in breathlessness in older cohorts. Breathing exercises were possibly more effective for HRQoL in predominantly female cohorts, for breathlessness in those less severe baseline breathlessness and for exercise capacity in those with worse baseline exercise capacity. Psychological interventions were possibly more effective for breathlessness and HRQoL in cohorts with a higher proportion of females. Nutritional interventions were more effective in those with worse HRQoL and breathlessness at baseline.

Programme duration did not impact the effectiveness of any component. The total number of programme sessions did not influence change in exercise capacity (SMD increase for every additional 100 programme sessions 0.005 95% CrI −0.03; 0.04), HRQoL (SMD −0.02 95% CrI −0.05; 0.02) or breathlessness (SMD 0.02 95% CrI −0.02; 0.07). Total programme “dose” (number of sessions × duration of session) had no impact on exercise capacity (SMD 0.01 95% CrI −0.03; 0.03 increase for every additional 100 programme hours), HRQoL (SMD −0.02, CrI −0.06; 0.02) or breathlessness (SMD 0.00 95% CrI −0.002; 0.0001). Controlling for these factors by centring the component effects on the median number of sessions (24) and the median programme “dose” (24–27 h across the three outcomes) resulted in an increase in effect for moderate intensity exercise on exercise capacity but no other change in component effects (Supplementary Figures S45–S50).

## Discussion

This is the largest and most detailed meta-analysis of PR including 337 randomised controlled trials using the novel technique of cNMA to investigate components of PR design in nearly 19,000 people with COPD. We have quantified the contribution of a diverse range of exercise and non-exercise components of PR on important patient centred outcomes which provides key information for payers and those developing PR services to balance effectiveness and efficiency. Despite the unequivocal evidence for the effectiveness of PR as a multicomponent “package”, significant questions remain about the essential PR components and optimal programme supervision, duration and intensity of training. The results of the current study address these evidence gaps and show that the impact of the exercise component of PR is enhanced by supervision (in-person and with less certainty, remote) and intensity of aerobic exercise but not programme duration. Furthermore, there are likely small additional gains in outcomes for PR programmes that include inspiratory muscle training, breathing exercises and psychological interventions but not nutritional interventions, strength training or education in an unselected population.

cNMA is a relatively novel statistical method that has been gaining increasing interest as a tool to investigate complex medical interventions.^4^ Traditional pairwise meta-analysis, whilst having the advantage of simplicity, gives a single result for heterogeneous interventions which may not be equally effective. cNMA is a regression-based technique which, in its simplest form, assumes the effect of an intervention is equal to the sum of the effects of the components within that intervention.^18^ It is therefore able to estimate the effect of single components within complex interventions such as PR. We used broad inclusion criteria for our study to maximise the variety in programme design available for analysis which would allow us to better estimate the effects of individual components. Whilst we understand that this definition would result in including many trials that many PR experts would not consider PR, this was our intention, as our aim was to provide an evidence-base for what PR programmes should include rather than basing the definition on expert opinion, which currently leads to conflicting PR definitions and program designs globally.

The importance of supervision during PR has been widely debated and has been under increasing scrutiny since the COVID-19 pandemic when many PR programmes were forced to use virtual delivery methods.^19^ Whilst we found that exercise training alone, without supervision, improved exercise capacity and HRQoL with a trend towards a small improvement in breathlessness, our results highlight the effectiveness of in-person supervision with a moderate increase in effect sizes compared to unsupervised exercise training for all outcomes considered. Whilst the magnitude of effect appeared similar for remote and in-person supervision, the certainty of evidence was low for remote supervision and the effects for HRQoL and breathlessness did not meet statistical significance. However, due to the strong correlation between supervision and location we cannot rule out location, as opposed to supervision, being the causal factor.

We found clear evidence that supervised programmes were superior to unsupervised programmes. The recent American Thoracic Society (ATS) guidance on PR recommends offering patients a choice between telerehabilitation and centre-based PR^20^ although there have since been calls to ensure that telerehabilitation programmes meet the same standards as face to face programmes.^21^ Our results suggest that in-person and remotely supervised programmes may be equally effective but the certainty around the relative effectiveness of remote programmes is lower. This may reflect the maturity of the literature with a tenfold higher number of RCTs investigating in-person PR compared to remote PR. The current evidence therefore supports a primary offer of an in-person supervised programme to all referred patients and supports the need for prescription of exercise training intensity for all programmes, regardless of the method of delivery to maximise outcomes for patients. Further high-quality trials investigating a variety of remotely delivered PR models are needed to determine if these programs can effectively substitute in-person programmes.

In our primary analysis, we did not identify clear evidence that higher aerobic exercise intensity led to greater improvements for any outcome. However, the most effective aerobic components were those prescribed at high or very high intensity for all outcomes. Lack of a clear dose relationship contrasts with previous analyses which have shown a dose dependent effect between the intensity of aerobic exercise and changes in peak exercise capacity measured by peak workload or peak oxygen uptake.^22^^,^^23^ Our review steering team (which included clinical experts in PR) felt that the effect of intensity of aerobic training was a more important question than the modality or type of exercise performed which was not considered in the current study. Studies included in our analysis used a wide variety of methods to prescribe exercise intensity including physiological measures and symptoms which made categorisation of intensity challenging. This supports the need to prescribe intensity and ensure intensity is sufficient to maximise gains from PR however, more evidence is needed to confirm this effect.

In our primary analysis, low intensity aerobic training appeared to elicit similar improvements in exercise capacity to high intensity aerobic training. Many of the studies coded as low intensity, prescribed exercise intensity based on a 6-minute walk test (6MWT) and there is limited international guidance on prescribing from a 6MWT as this is not a measure of maximal exercise performance.^24^ It is possible that many of these patients actually walked at higher intensities and we hypothesis this is the reason for the effect seen. Additionally, we assessed intensity at the beginning of the programme, and it is possible that some studies commenced training at a low intensity that rapidly increased.

Addition of strength training did not improve HRQoL and breathlessness although there was a trend toward benefit in exercise capacity from lower limb strength training. The reasons for this observation are important to explore. We recognise that muscle strength was not included as an outcome and therefore beneficial effects of strength training are likely to have been missed.

The addition of structured education did not appear to improve any of the outcomes investigated. A structured education programme has traditionally been a core component of PR and forms part of international definitions of PR^8^ however, recent guidelines have questioned whether structured education is required, based on a lack of evidence of additional benefit over supervised exercise alone.^2^^,^^25^ Our analysis shows that education probably doesn't improve these three core outcomes of PR. Moreover, the broader benefits of education may have been underestimated because outcomes that relate to knowledge, behaviour change and self-management capability were not included.

Programme duration and total number of programme hours did not significantly impact programme effectiveness. The lack of effect may be partially explained by the lack of information on adherence and completion rates. Furthermore, we would expect that the relationship is unlikely to be linear with possibly a minimum duration after which the impact of further sessions is reduced. It is likely that our analysis is not ideally suited to answer this question and we are not able to recommend a minimum duration for PR programmes.

Our models suggest that some interventions may be more effective when delivered to certain populations, for example, supervision appeared to be more effective for improving breathlessness in older cohorts. This supports the need for an individualised approach to PR^26^ rather than a “one-size-fits-all” approach and the need for further focused research in this area. However, we were only able to assess the impact of cohort level demographics and individual patient data would be required to truly assess the impact of patient characteristics or “treatable traits” on outcomes. Whilst Asian and Chinese training modalities appear effective, they have almost exclusively been trialled in Asia and their effectiveness in other populations is unclear.

This is a large analysis of randomised controlled trials which was able to overcome many of the limitations of previous meta-analysis by considering the components, delivery and study populations in a single analysis. However, there are several limitations to be acknowledged. Firstly, these studies were conducted in the context of research and the included population may differ from those seen in routine clinical care. The assumption of network meta-analysis that an individual could reasonably have been allocated to any intervention arm required us to consider only a broad population of people with COPD and not subgroups with particular impairments or characteristics (for example nutritional depletion or psychological morbidity). We classified intensity based on prescribed rather than achieved intensity which was rarely reported. We focused on three core short term outcomes and some components may have a differing effect on other specific outcome measures or long-term health outcomes. We have not included results of secondary outcomes here which may be more specific to some included components. We assumed that the effects of components are additive which may not be the case for all included components and were unable to estimate interaction effects. We could not assess the difference in effectiveness of components in patients who were naïve to PR compared to those who had previously attended PR or the effect of regular medication on outcomes. We cannot comment on the effect of publication bias or inconsistency as no methods exist to evaluate these in component network meta-analysis^4^ however previous pairwise meta-analyses of pulmonary rehabilitation have not found evidence of publication bias.^1^^,^^11^ The number of components varied between interventions which may have led to a ceiling effect in studies with more components and we did not attempt to correct for this.

Finally, due to the scale and complexity of our analysis, there was an inevitable delay between the initial literature search and final publication. A subsequent search conducted at the time of publication identified an additional 35 randomised controlled trials that met the inclusion criteria which represents a 10% increase in the number of included studies covering a wide range of differing intervention designs from variations in exercise training, delivery, and non-exercise additions. These studies are listed in the supplement but were not included in the analysis to prevent further delays to publication and there is a possibility they may have introduced minor differences in component effects. However, there were no large pivotal studies included that conflicted with our current conclusions. Our previous update in October 2023 which covered a similar time period and included a similar number of studies did not result in meaningful change to the results. Future analyses of PR should seek to include these studies.

The components in these additional studies were not systematically extracted but appeared to vary widely. The exercise components included low to very high intensity aerobic training, strength training, callisthenics, tai chi, Mawangdui exercise, Qigong, yoga, Baduanjin, neuroelectrical electrical stimulation, and exercise in water. The non-exercise components performed alongside exercise training also varied widely and included some not previous studied in our review. These included melatonin supplementation, manual therapy, whey protein supplementation, cognitive behavioural therapy, participant selected music during exercise, body awareness therapy, chest wall mobilisation and shared decision-making. Some programmes were supervised in person, some remotely and some unsupervised. No clear themes could be identified from these additional studies.

In conclusion, this large analysis combining results of over 300 randomised trials in almost 19,000 people addresses the key outstanding questions from the last Cochrane review of PR and provides clear evidence for a PR programme containing in-person supervised and prescribed aerobic training for people with COPD. However, it highlights the lack of evidence for other PR components including strength training and structured education in an unselected population for the outcomes considered. Unsupervised exercise training whilst effective, is inferior to supervised training and whilst promising, there is lack of robust evidence for remotely supervised programmes. These results should inform future international PR guidelines and suggest future research should consider a wider range of outcome measures when measuring the impact of PR components.

## Contributors

TJCW led the project and drafted the manuscript. TJCW, LL, TCH, SJS, NJG, RAE, MCS, and AJS developed the concept and the analysis plan. LL coordinated the review. TJCW, LL, ED, SW, MH, MB, SR, AM, AO, JX, and HA assessed papers for eligibility. TJCW, LL, JX, and MB extracted data. TJCW, LL, ED, SW, LW, and MI assessed risk of bias. TJCW and LL performed GRADE assessment. TJCW, LL, ED, SW, TCH, SJS, NJG, RAE, and MCS provided expert insight into component classification. TJCW performed data analysis with support from SCF and AJS. TJCW, LL, MB, JX, and AJS had access to and verified the underlying data. All authors read and approved the final version of the manuscript.

## Data sharing statement

We welcome requests for collaborations and access to data (tom.ward@leicester.ac.uk).

## Declaration of interests

TJC Ward reports grants and personal fees from Chiesi and GlaxoSmithKline and is a member of the British Thoracic Society COPD Specialist Advisory Group. E Daynes reports personal fees from Chiesi and the Royal College of Physicians and is a member of the British Thoracic Society Pulmonary Rehabilitation Group. NJ Greening reports personal and institutional grants and fees from Wellcome Leap, GlaxoSmithKline, Roche, Astrazeneca, Chiesi, Pulmonx, Roche, Sanofi and is chair of the British Thoracic Society COPD Specialist Advisory Group. RA Evans reports personal and institutional grants and fees from NIHR, UKRI, Wolfson Foundation, Genentec/Roche, Astrazeneca/Evidera, Moderna and is chair of the European Respiratory Society Pulmonary Rehabilitations and Chronic Care group and the Amercian Thoracic Society Pulmonary Rehabilitation Assembly. MC Steiner reports a conference travel grant from AstraZeneca and unpaid data monitoring committee roles for trials of pulmonary rehabilitation and beta blockers in COPD. All other authors have no relevant conflicts to declare.
