# Supplementary material for: Impact of pulmonary rehabilitation programme design on effectiveness in COPD: a systematic review and component network meta-analysis
Source: eClinicalMedicine. 2025 Aug 20;87:103433. doi: 10.1016/j.eclinm.2025.103433 (PMC12396583; doi:10.1016/j.eclinm.2025.103433)
Supplement: Supplementary Materials [file mmc1.docx]

THE IMPACT OF PULMONARY REHABILITATION PROGRAMME DESIGN ON OUTCOMES IN COPD: A SYSTEMATIC REVIEW AND COMPONENT NETWORK META-ANALYSIS

Supplementary material

Contents

[Supplementary Methods 2](#_Toc201674991)

[Search strategies 2](#_Toc201674992)

[Supplementary results 7](#_Toc201674993)

[Component frequency 8](#_Toc201674994)

[Location of included studies 9](#_Toc201674995)

[Correlation and interaction heat plots 10](#_Toc201674996)

[Frequency of outcome measures 22](#_Toc201674997)

[Leverage plots 23](#_Toc201674998)

[Supplementary results - interim models 25](#_Toc201674999)

[Supplementary results - interaction models 31](#_Toc201675000)

[Supplementary results – covariate models 32](#_Toc201675001)

[**Supplementary table 3** – Assessment of model fit after addition of interaction and covariate effects 64](#_Toc201675002)

[Risk of bias and quality of reporting 66](#_Toc201675003)

[GRADE assessment of certainty of evidence 72](#_Toc201675004)

[Sensitivity analyses 83](#_Toc201675005)

[**Contour adjusted funnel plots** 108](#_Toc201675006)

[Characteristics of included studies 112](#_Toc201675007)

[**Supplementary table 15 -** Characteristics of interventions in included studies 112](#_Toc201675008)

[**Supplementary table 16 -** Demographics of cohorts in included studies. 152](#_Toc201675009)

[**Supplementary table 17 –** Description of individual components in included studies 180](#_Toc201675010)

[**Supplementary figure 84–** PRISMA flow chart for updated search conducted in May 2025 prior to publication 190](#_Toc201675011)

[**Supplementary table 18 –** Included studies in updated review May 2025 (not included in analysis) 191](#_Toc201675012)

[Example WinBugs models 193](#_Toc201675013)

[Additive model 193](#_Toc201675014)

[Interaction model 195](#_Toc201675015)

[Example covariate model – outcome at baseline 197](#_Toc201675016)

[References 199](#_Toc201675017)

# Supplementary Methods

## Search strategies

For the Medline, Embase & Emcare databases, the Cochrane Library’s highly sensitive search filter for RCTs was applied. <https://training.cochrane.org/handbook/version-6/chapter-4-tech-suppl>

Ovid MEDLINE(R) ALL <1946 to March 09, 2022>

1 Lung Diseases, Obstructive/ 18254

2 exp Pulmonary Disease, Chronic Obstructive/ 62363

3 emphysema*.ti,ab,kw,kf. 29540

4 (chronic* adj3 bronchiti*).ti,ab,kw,kf. 11550

5 (obstruct* adj3 (pulmonary or lung* or airway* or airflow* or bronch* or respirat*)).ti,ab,kw,kf. 97957

6 (coad or cobd or copd or aecb).ti,ab,kw,kf. 54169

7 or/1-6 158796

8 Exercise Therapy/ or Endurance Training/ or Muscle Stretching Exercises/ or Plyometric Exercise/ or Resistance Training/ 58029

9 Exercise/ or exercis*.mp. 430121

10 (aerobic* or walk or walking or cycle* or cycling or bicycle* or running or jogging).mp. [mp=title, abstract, original title, name of substance word, subject heading word, floating sub-heading word, keyword heading word, organism supplementary concept word, protocol supplementary concept word, rare disease supplementary concept word, unique identifier, synonyms] 1059780

11 Walking/ 37878

12 Bicycling/ 12277

13 Jogging/ 834

14 exp Running/ 22531

15 rehab*.mp. [mp=title, abstract, original title, name of substance word, subject heading word, floating sub-heading word, keyword heading word, organism supplementary concept word, protocol supplementary concept word, rare disease supplementary concept word, unique identifier, synonyms] 354911

16 or/8-15 1690622

17 7 and 16 16523

18 randomized controlled trial.pt. 560636

19 controlled clinical trial.pt. 94727

20 randomized.ab. 553044

21 placebo.ab. 226175

22 drug therapy.fs. 2453853

23 randomly.ab. 377440

24 trial.ab. 590162

25 groups.ab. 2320084

26 or/18-25 5281886

27 exp animals/ not humans.sh. 4969296

28 26 not 27 4596072

29 17 and 28 5821

Embase <1974 to 2022 March 09>

1 obstructive lung disease/ 219

2 exp chronic obstructive lung disease/ 153037

3 emphysema*.ti,ab,kw,kf. 37347

4 lung emphysema/ 14647

5 (chronic* adj3 bronchiti*).ti,ab,kw,kf. 14335

6 chronic bronchitis/ 12270

7 (obstruct* adj3 (pulmonary or lung* or airway* or airflow* or bronch* or respirat*)).ti,ab,kw,kf. 143202

8 (coad or cobd or copd or aecb).ti,ab,kw,kf. 101749

9 or/1-8 266263

10 kinesiotherapy/ or endurance training/ or stretching exercise/ or plyometrics/ or resistance training/ 66726

11 exercise/ or exercis*.mp. 586295

12 aerobic exercise/ 19248

13 (aerobic* or walk or walking or cycle* or cycling or bicycle* or running or jogging).mp. 1526715

14 walking/ 75903

15 cycling/ 13824

16 jogging/ 2277

17 exp running/ 35364

18 rehab*.mp. 414294

19 or/10-18 2312413

20 Randomized controlled trial/ 699203

21 Controlled clinical study/ 465169

22 random*.ti,ab. 1763736

23 randomization/ 93284

24 intermethod comparison/ 280683

25 placebo.ti,ab. 337672

26 (compare or compared or comparison).ti. 559100

27 ((evaluated or evaluate or evaluating or assessed or assess) and (compare or compared or comparing or comparison)).ab. 2458902

28 (open adj label).ti,ab. 95169

29 ((double or single or doubly or singly) adj (blind or blinded or blindly)).ti,ab. 254187

30 double blind procedure/ 193026

31 parallel group*1.ti,ab. 29033

32 (crossover or cross over).ti,ab. 115149

33 ((assign* or match or matched or allocation) adj5 (alternate or group*1 or intervention*1 or patient*1 or subject*1 or participant*1)).ti,ab. 374643

34 (assigned or allocated).ti,ab. 441195

35 (controlled adj7 (study or design or trial)).ti,ab. 401794

36 (volunteer or volunteers).ti,ab. 265530

37 human experiment/ 568604

38 or/22-37 5465040

39 (random$ adj sampl$ adj7 ("cross section$" or questionnaire$1 or survey$ or database$1)).ti,ab. not (comparative study/ or controlled study/ or randomi?ed controlled.ti,ab. or randomly assigned.ti,ab.) 8885

40 Cross-sectional study/ not (randomized controlled trial/ or controlled clinical study/ or controlled study/ or randomi?ed controlled.ti,ab. or control group*1.ti,ab.) 301383

41 (((case adj control*) and random*) not randomi?ed controlled).ti,ab. 19532

42 (Systematic review not (trial or study)).ti. 202911

43 (nonrandom* not random*).ti,ab. 17628

44 "Random field*".ti,ab. 2662

45 (random cluster adj3 sampl*).ti,ab. 1418

46 (review.ab. and review.pt.) not trial.ti. 972848

47 "we searched".ab. and (review.ti. or review.pt.) 40838

48 update review.ab. 119

49 (databases adj4 searched).ab. 49118

50 (rat or rats or mouse or mice or swine or porcine or murine or sheep or lambs or pigs or piglets or rabbit or rabbits or cat or cats or dog or dogs or cattle or bovine or monkey or monkeys or trout or marmoset*1).ti. and animal experiment/ 1141407

51 Animal experiment/ not (human experiment/ or human/) 2395870

52 or/39-51 3909628

53 38 not 52 4824768

54 9 and 19 and 53 8583

CINAHL

Top of Form

| Thursday, March 10, 2022 4:21:51 PM |
| --- |

| **#** | **Query** | **Results** |
| --- | --- | --- |
| S1 | (MH "Lung Diseases, Obstructive") | 4,434 |
| S2 | (MH "Pulmonary Disease, Chronic Obstructive+") | 21,535 |
| S3 | (MH "Emphysema") | 2,822 |
| S4 | chronic* N3 bronchiti* | 1,315 |
| S5 | obstruct* n3 (pulmonary or lung* or airway* or airflow* or bronch* or respirat*) | 40,785 |
| S6 | coad or cobd or copd or aecb | 25,428 |
| S7 | emphysema* | 21,649 |
| S8 | S1 OR S2 OR S3 OR S4 OR S5 OR S6 OR S7 | 48,274 |
| S9 | MH therapuetic exercise OR MH endurance training OR MH resistance training OR MH stretching OR MH plyometrics | 13,096 |
| S10 | (MH "Exercise") OR "exercis*" | 206,038 |
| S11 | (MH "Aerobic Exercises") OR (MH "Cycling") OR (MH "Walking") OR (MH "Running") | 48,495 |
| S12 | (MH "Jogging") | 279 |
| S13 | rehab* | 197,052 |
| S14 | aerobic* or walk or walking or cycle* or cycling or bicycle* or running or jogging | 175,971 |
| S15 | S9 OR S10 OR S11 OR S12 OR S13 OR S14 | 502,616 |
| S16 | MH randomized controlled trials | 126,324 |
| S17 | MH “double‐blind studies” | 52,455 |
| S18 | MH “single‐blind studies” | 15,474 |
| S19 | MH random assignment | 73,015 |
| S20 | MH “pretest‐posttest design” | 48,810 |
| S21 | MH cluster sample | 4,927 |
| S22 | TI (randomised OR randomized) | 125,170 |
| S23 | MH sample size AND AB ( assigned OR allocated OR control ) | 4,312 |
| S24 | MH placebos | 13,275 |
| S25 | PT randomized controlled trial | 139,681 |
| S26 | AB control N5 group | 143,744 |
| S27 | AB random* | 367,779 |
| S28 | TI trial | 161,483 |
| S29 | MH crossover design OR MH comparative studies | 443,052 |
| S30 | AB cluster N3 RCT | 475 |
| S31 | (MH "Animals+") | 99,237 |
| S32 | MH animal studies | 144,591 |
| S33 | TI animal model* | 3,279 |
| S34 | S31 OR S32 OR S33 | 235,020 |
| S35 | MH human | 2,511,437 |
| S36 | s34 not s35 | 202,443 |
| S37 | S16 OR S17 OR S18 OR S19 OR S20 OR S21 OR S22 OR S23 OR S24 OR S25 OR S26 OR S27 OR S28 OR S29 OR S30 | 946,327 |
| S38 | s37 not s36 | 899,928 |
| S39 | S8 AND S15 AND S38 | 1,843 |

Cochrane CENTRAL

ID Search

#1 MeSH descriptor: [Lung Diseases, Obstructive] explode all trees

#2 MeSH descriptor: [Pulmonary Disease, Chronic Obstructive] explode all trees

#3 emphysema*

#4 chronic* near/3 bronchiti*

#5 obstruct* near/3 (pulmonary or lung* or airway* or airflow* or bronch* or respirat*)

#6 coad or cobd or copd or aecb

#7 {or #1-#6}

#8 MeSH descriptor: [Exercise Therapy] explode all trees

#9 MeSH descriptor: [Endurance Training] explode all trees

#10 MeSH descriptor: [Muscle Stretching Exercises] explode all trees

#11 MeSH descriptor: [Plyometric Exercise] explode all trees

#12 MeSH descriptor: [Resistance Training] explode all trees

#13 MeSH descriptor: [Exercise] explode all trees

#14 exercis*

#15 aerobic* or walk or walking or cycle* or cycling or bicycle* or running or jogging

#16 MeSH descriptor: [Walking] explode all trees

#17 MeSH descriptor: [Bicycling] explode all trees

#18 MeSH descriptor: [Jogging] explode all trees

#19 rehab*

#20 {or #8-#19}

#21 #7 and #20Bottom of Form

# Supplementary results

*Demographics of included studies*

There was variation in reporting of participant characteristics with age reported in 97% of studies, sex or gender in 92% of studies, BMI in 78% of studies, FEV_1_ in 93% of studies, ethnicity in 2% of studies, religion in 1% of studies, socioeconomic status in 11% of studies, and sexual orientation or gender reassignment was not reported. Included participants had a mean age of 64 years, 69% male with mean FEV_1_ of 43% predicted. Study duration ranged from 3 to 52 weeks.

##
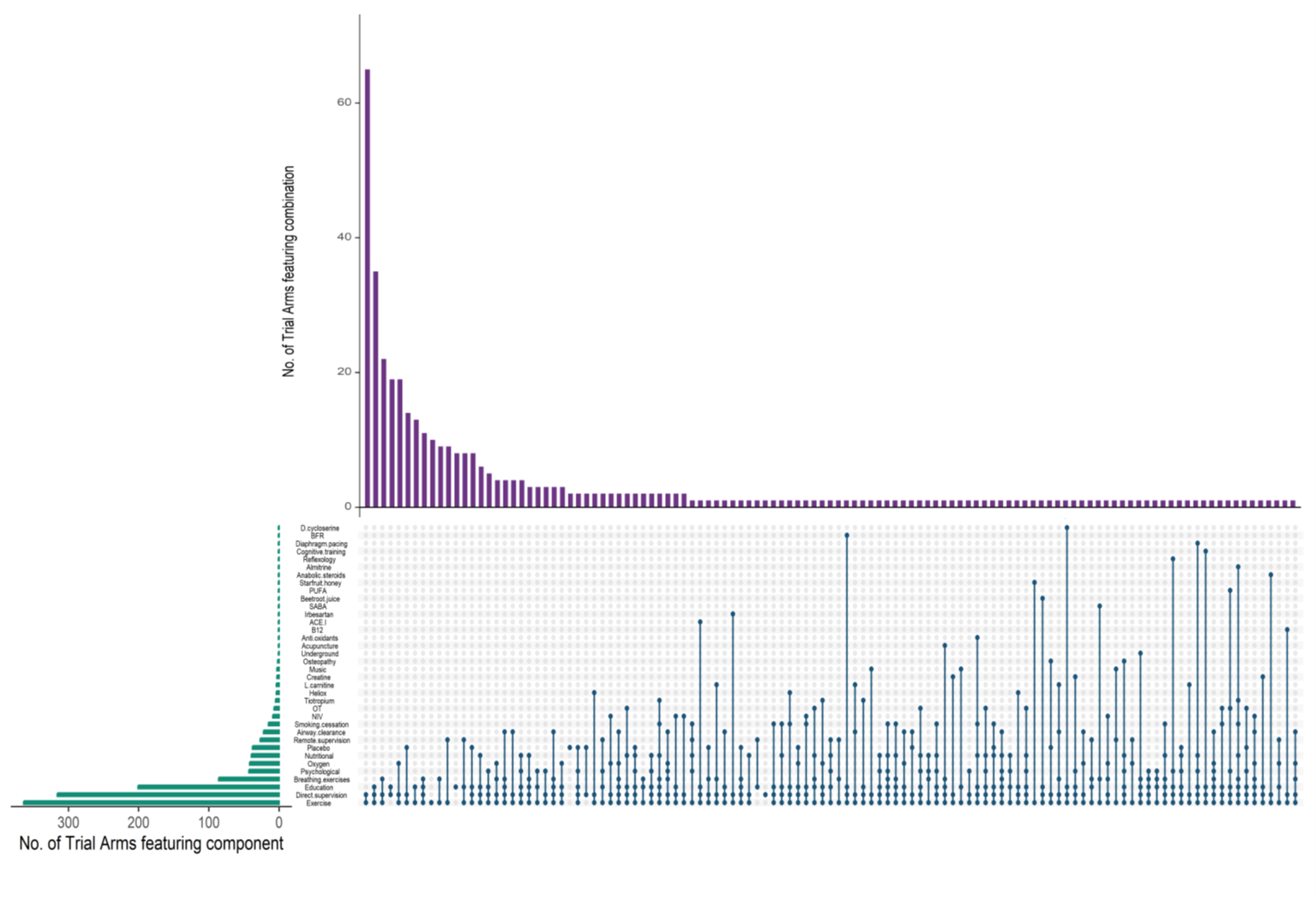
Component frequency

**Supplementary figure 1** - Upset plot – interim model, exercise capacity

## Location of included studies

**a)**


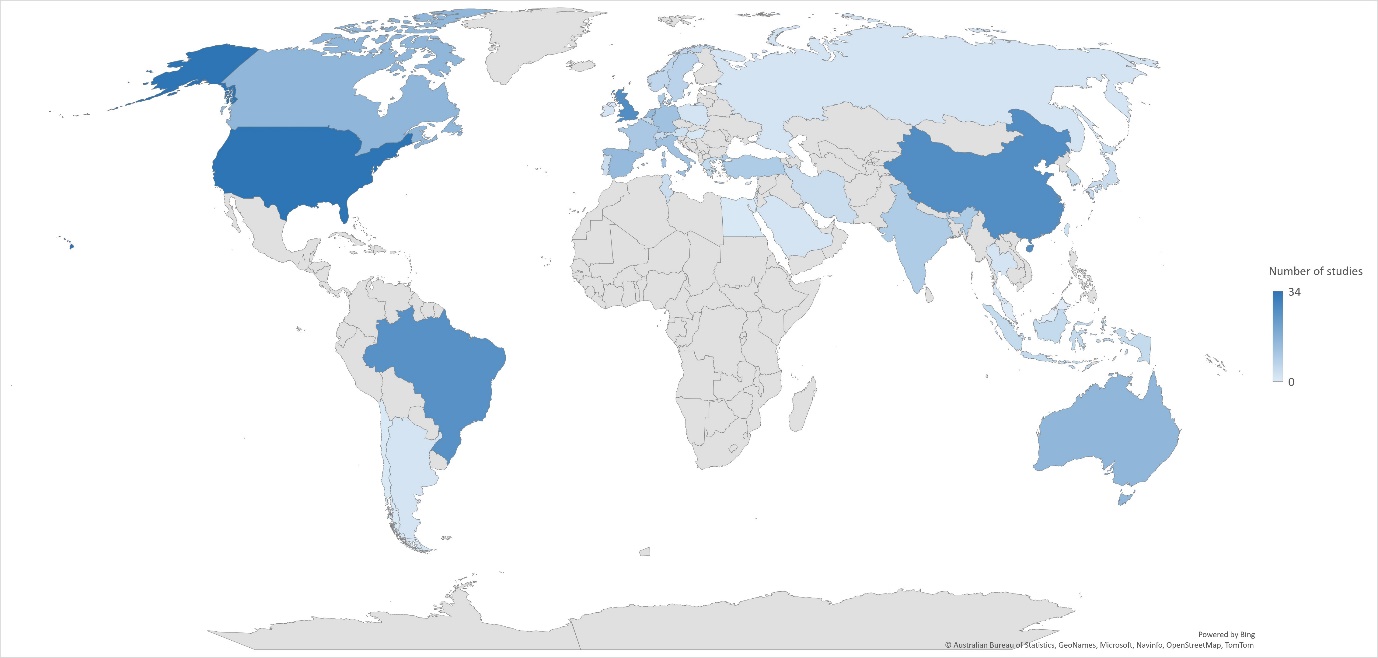


**b)**


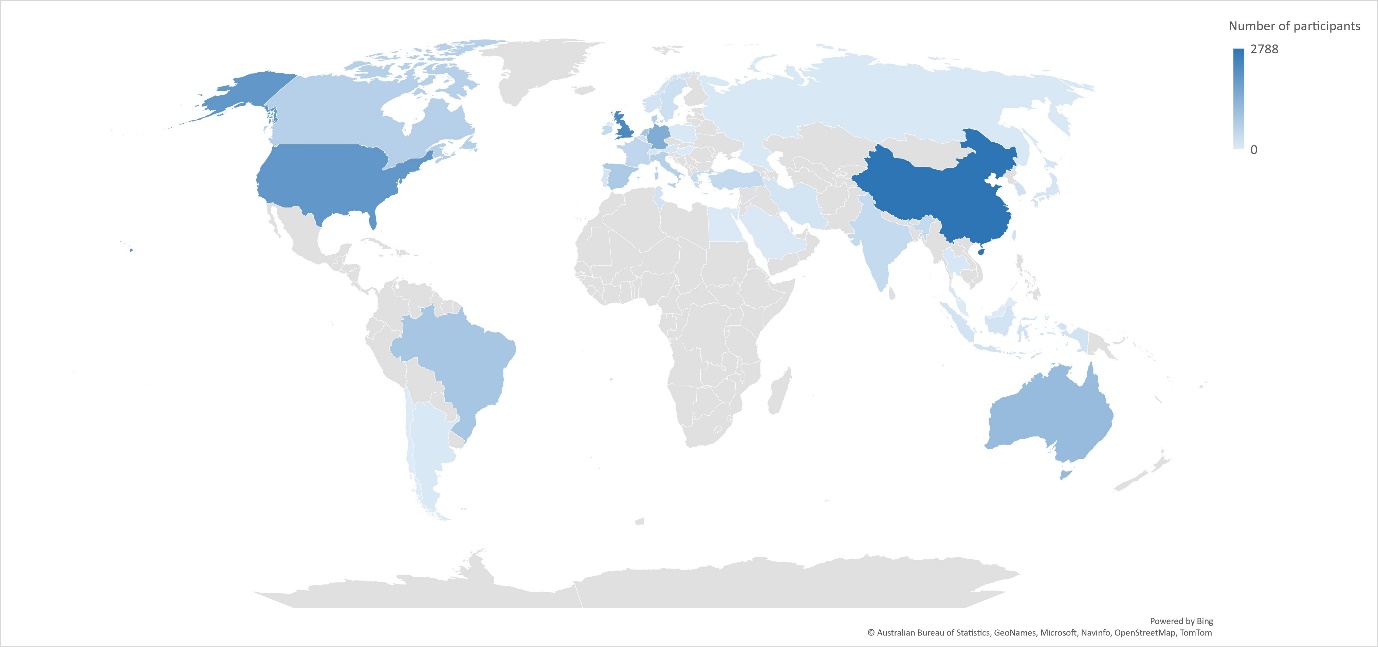


**Supplementary figure 2** – Location of included studies by a) number of studies, b) number of randomised participants.

## Correlation and interaction heat plots


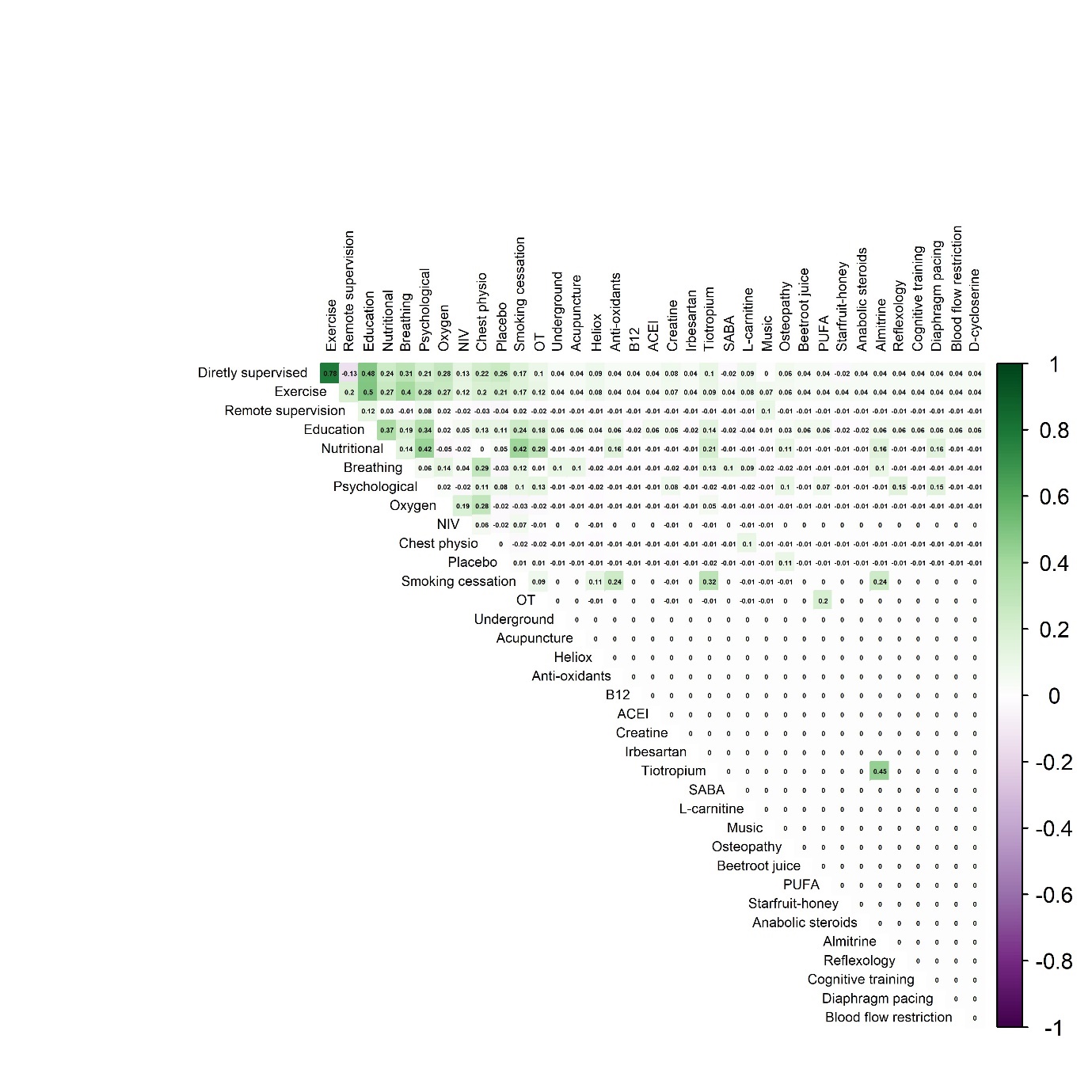


**Supplementary figure 3 – Heat plot for interim model, exercise capacity.** Plot of correlations between components in combined model with exercise capacity as outcome. NIV; non-invasive ventilation, OT; occupational therapy, B12: vitamin B12, ACEI; ACE inhibitor, PUFA: polyunsaturated fatty acids


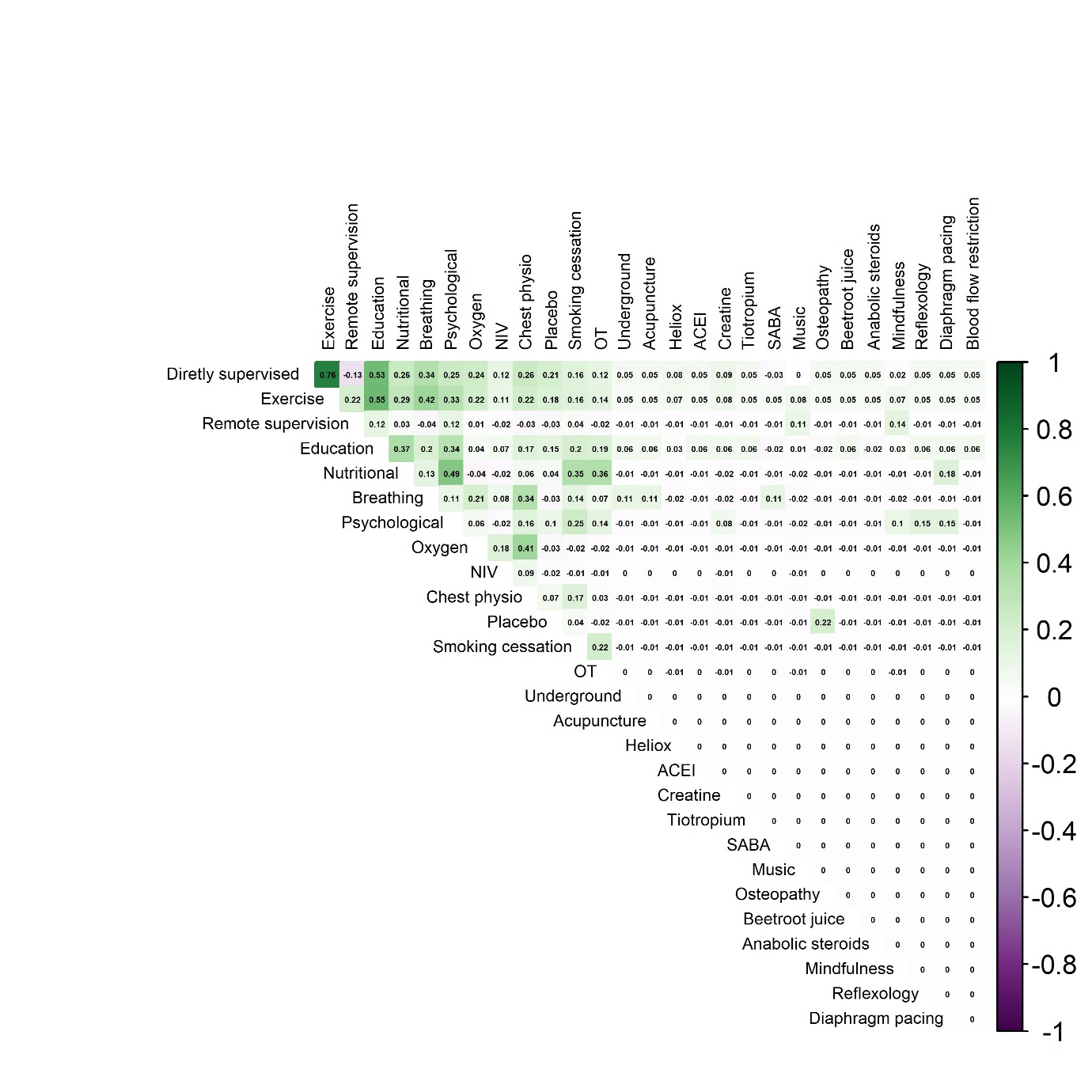


**Supplementary figure 4 – Heat plot for interim model, quality of life.** Plot of correlations between components in combined model with quality of life as outcome. NIV; non-invasive ventilation, OT; occupational therapy, ACEI; ACE inhibitor


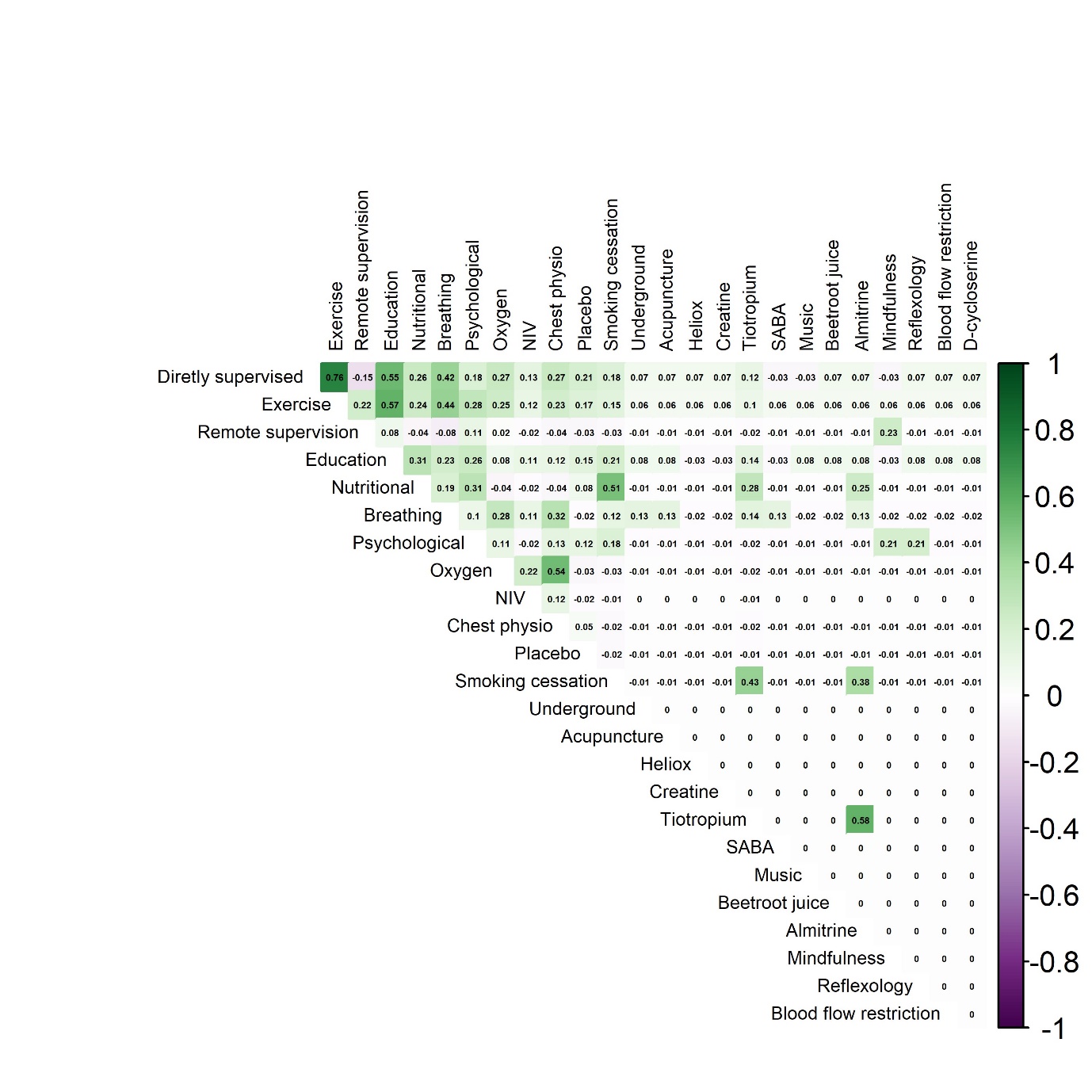


**Supplementary figure 5 – Heat plot for interim model, breathlessness.** Plot of correlations between components in combined model with breathlessness as outcome. NIV; non-invasive ventilation.


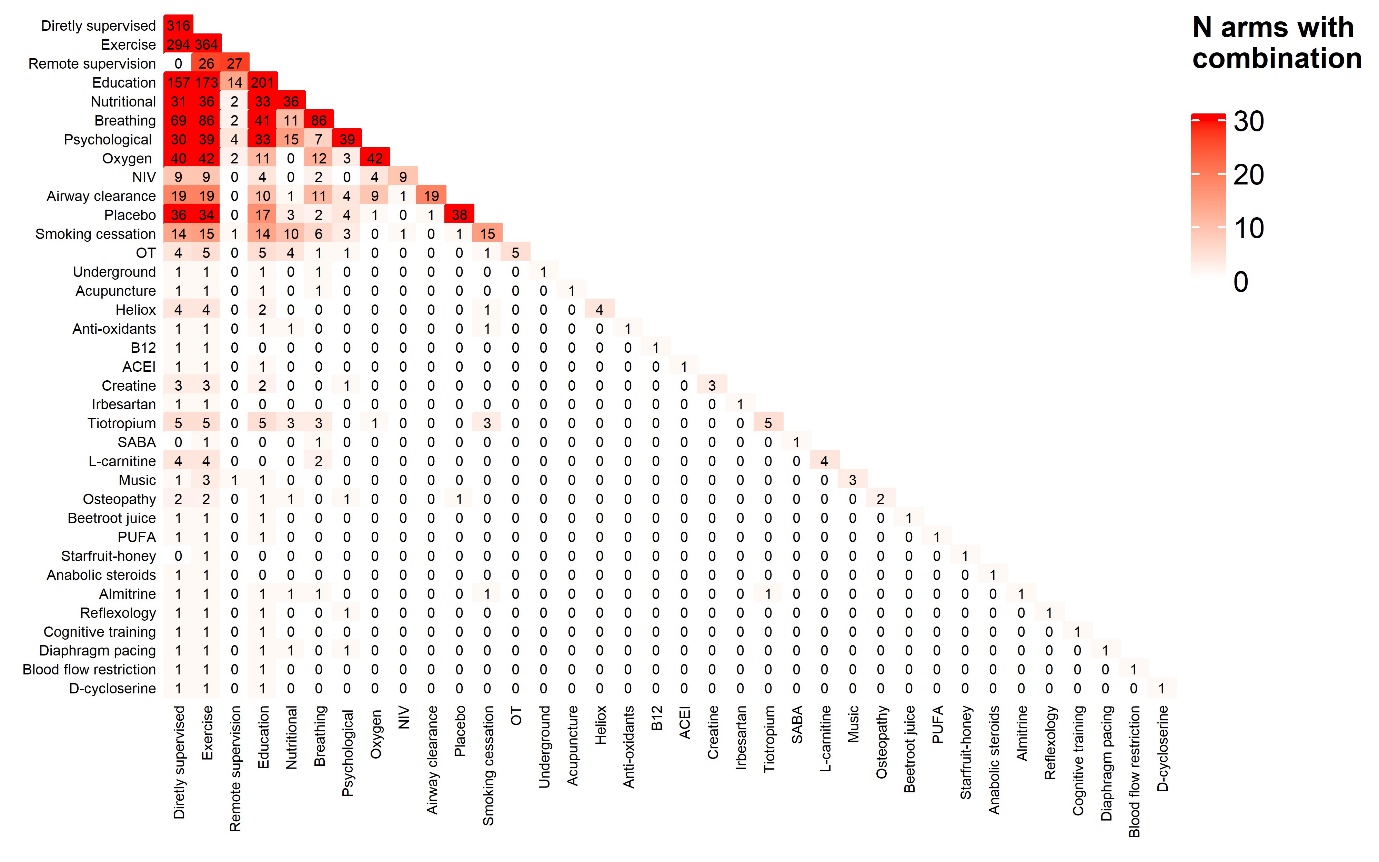


**Supplementary figure 6 – Interaction plot for interim model, exercise capacity.** Plot of frequency of combination of components in combined model with exercise capacity as outcome. NIV; non-invasive ventilation, OT; occupational therapy, B12: vitamin B12, ACEI; ACE inhibitor, PUFA: polyunsaturated fatty acids


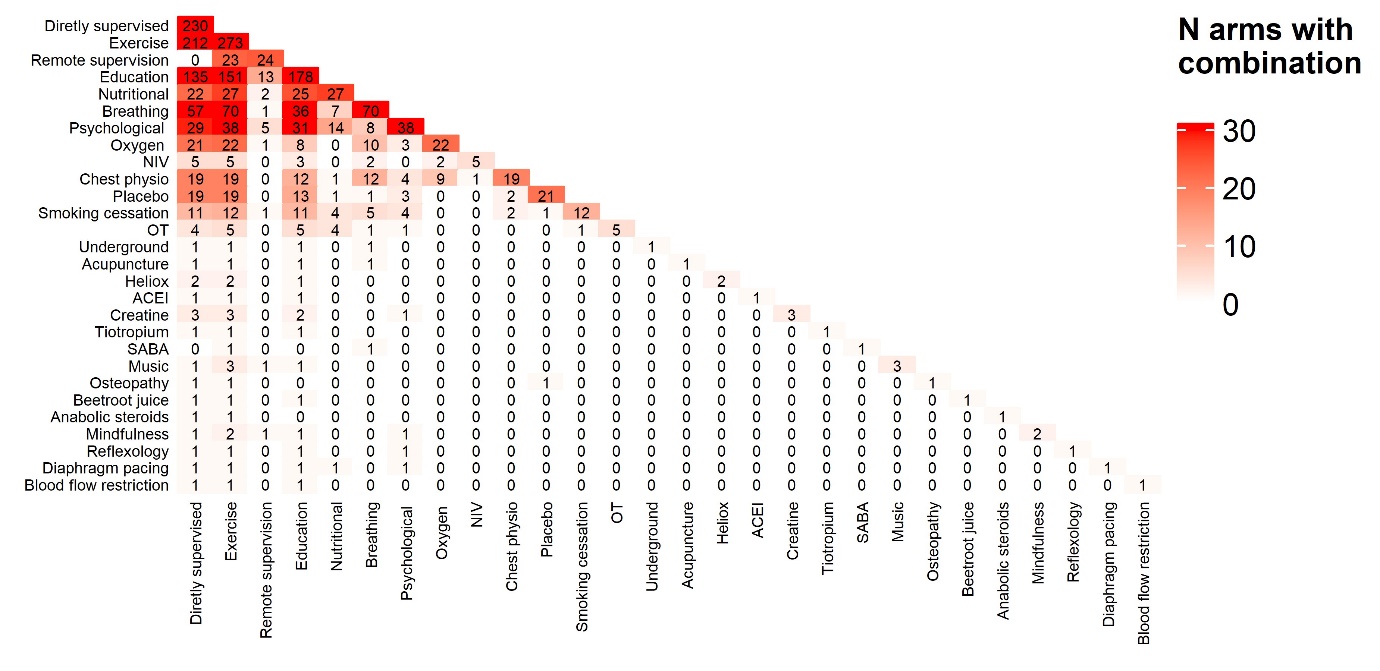


**Supplementary figure 7 – Interaction plot for interim model, quality of life.** Plot of frequency of combination of components in combined model with quality of life as outcome. NIV; non-invasive ventilation, OT; occupational therapy, B12: vitamin B12, ACEI; ACE inhibitor, PUFA: polyunsaturated fatty acids


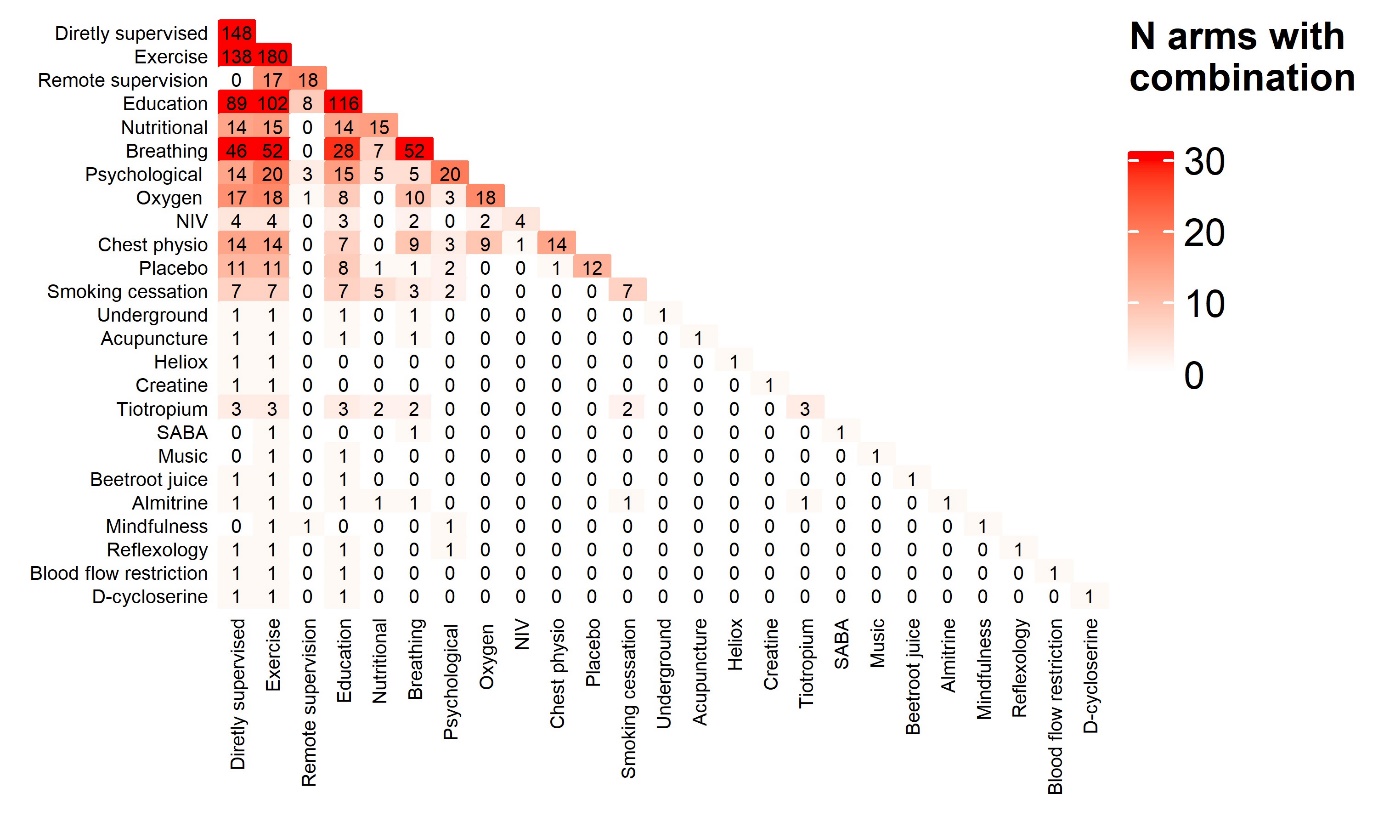


**Supplementary figure 8 – Interaction plot for interim model, breathlessness.** Plot of frequency of combination of components in combined model with dyspnoa as outcome. NIV; non-invasive ventilation.


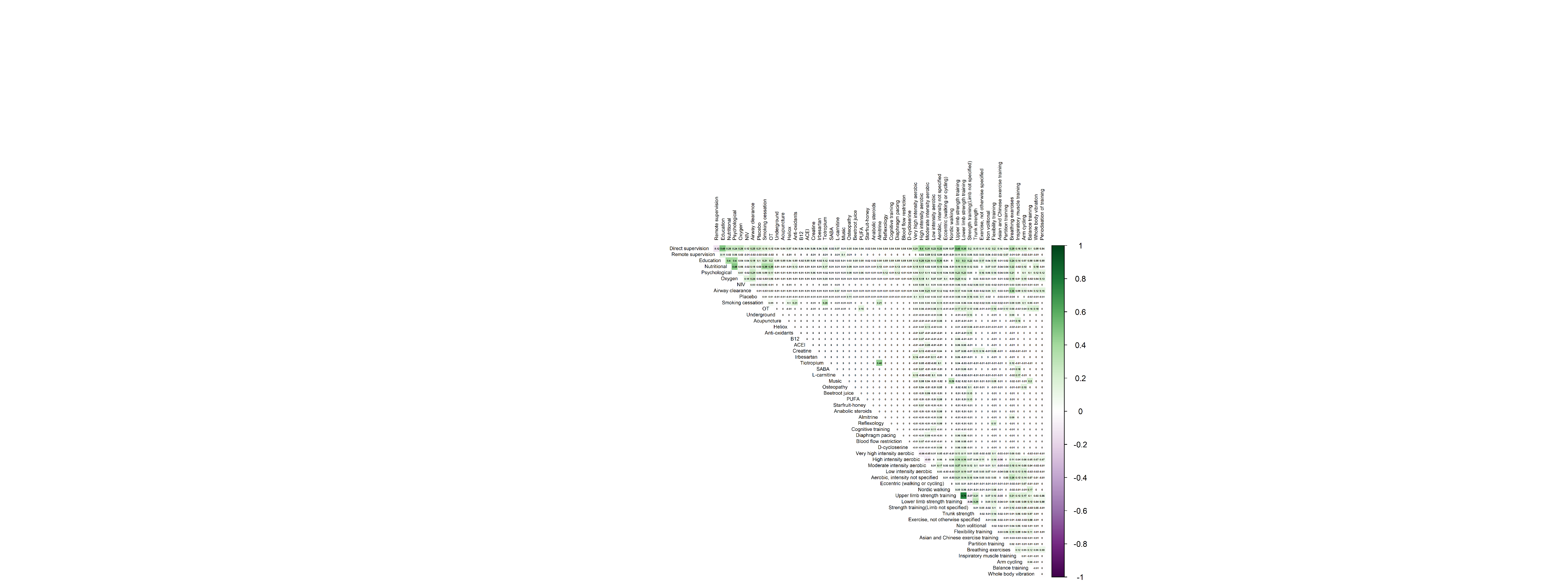


**Supplementary figure 9 – Correlation plot for Final model, exercise capacity.** Plot of correlations between components in combined model with exercise capacity as outcome. NIV; non-invasive ventilation, OT; occupational therapy, B12: vitamin B12, ACEI; ACE inhibitor, PUFA: polyunsaturated fatty acids


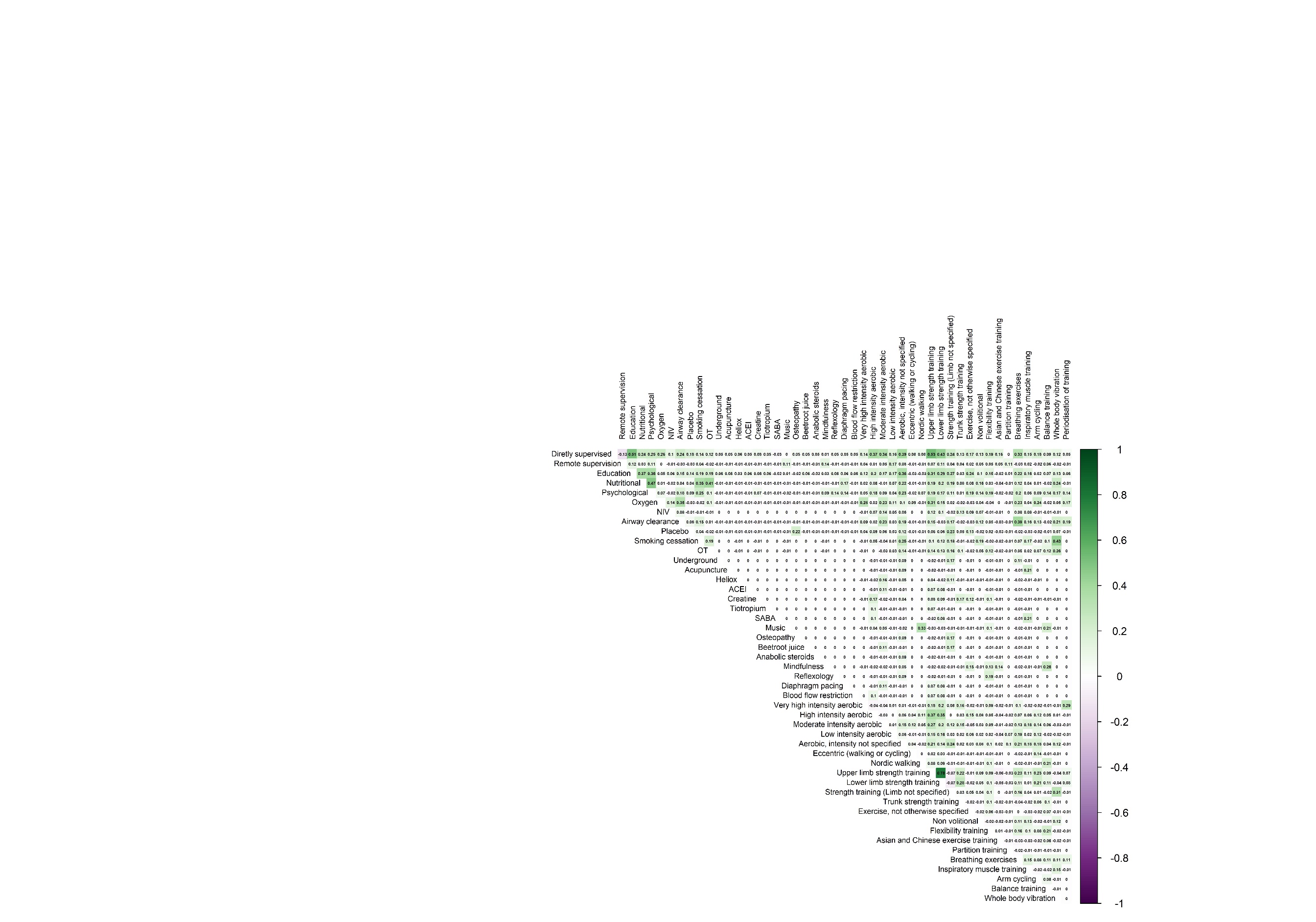


**Supplementary figure 10 – Correlation plot for Final model, quality of life.** Plot of correlations between components in combined model with exercise capacity as outcome. NIV; non-invasive ventilation, OT; occupational therapy, B12: vitamin B12, ACEI; ACE inhibitor, PUFA: polyunsaturated fatty acids


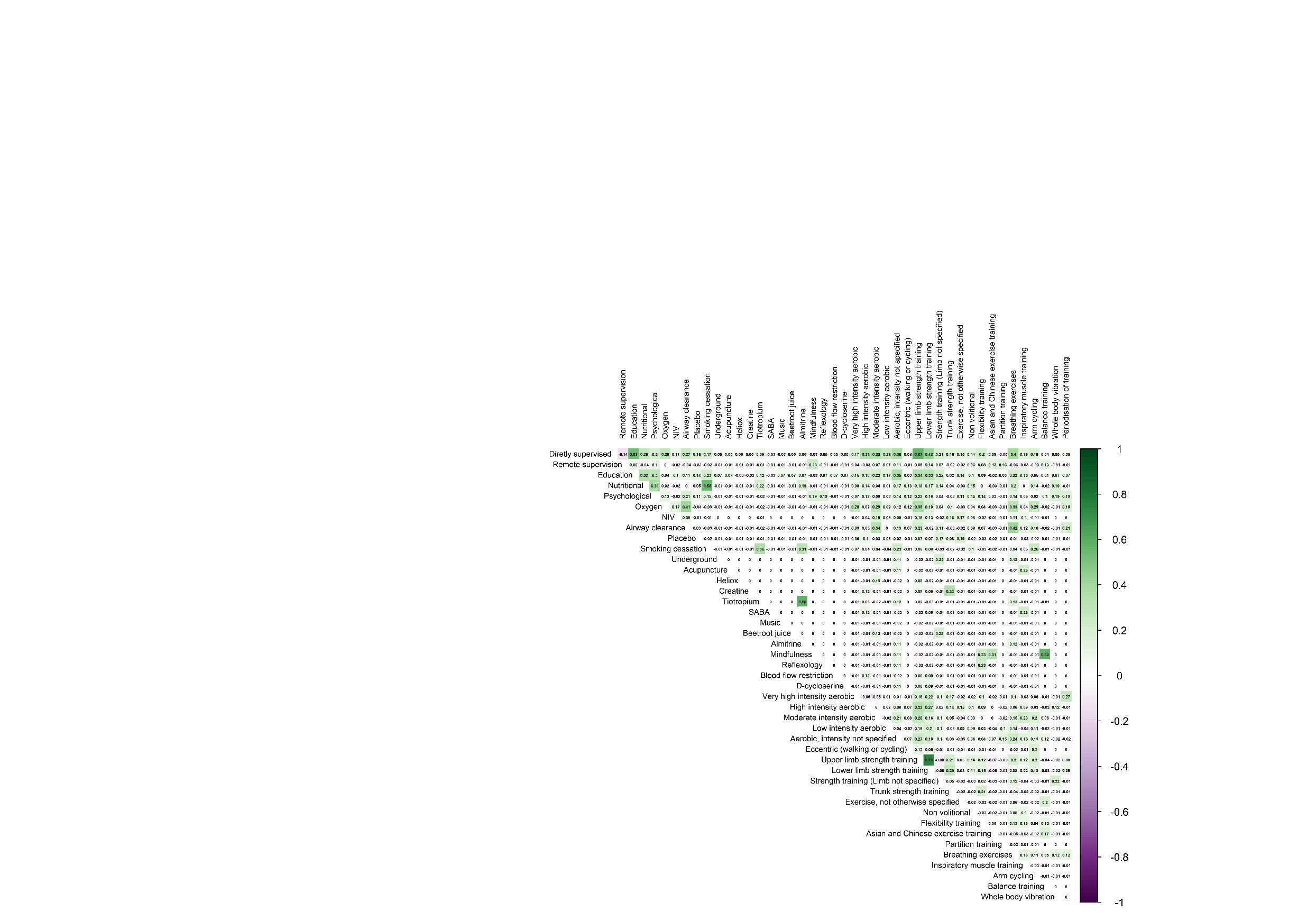


**Supplementary figure 11 – Correlation plot for Final model, breathlessness.** Plot of correlations between components in combined model with exercise capacity as outcome. NIV; non-invasive ventilation, OT; occupational therapy, B12: vitamin B12, ACEI; ACE inhibitor, PUFA: polyunsaturated fatty acids


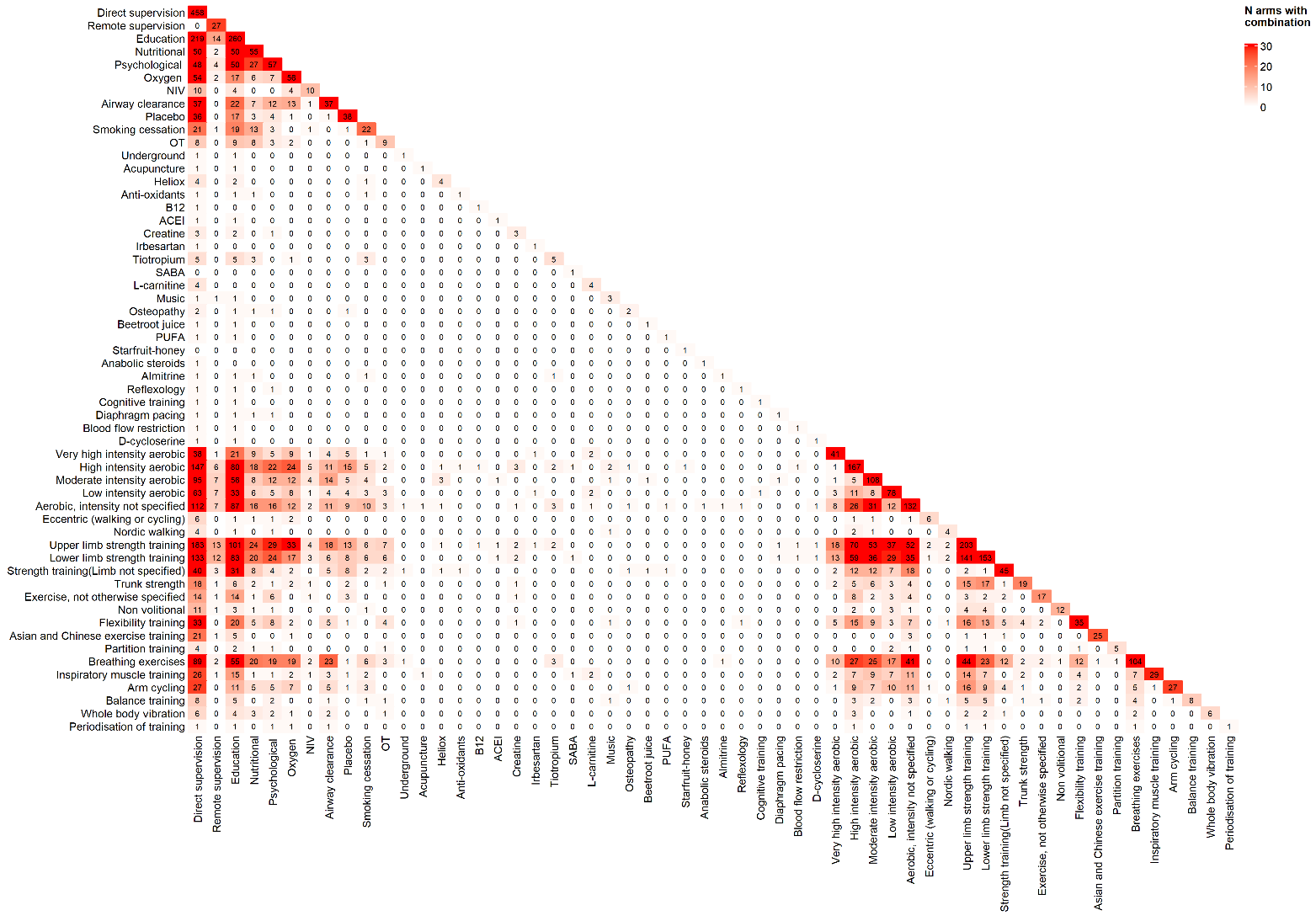


**Supplementary figure 12 – Interaction heat map for Final model, exercise capacity.** Plot of frequency of combination of components in combined model with exercise capacity as outcome. NIV; non-invasive ventilation, OT; occupational therapy, B12: vitamin B12, ACEI; ACE inhibitor, PUFA: polyunsaturated fatty acids


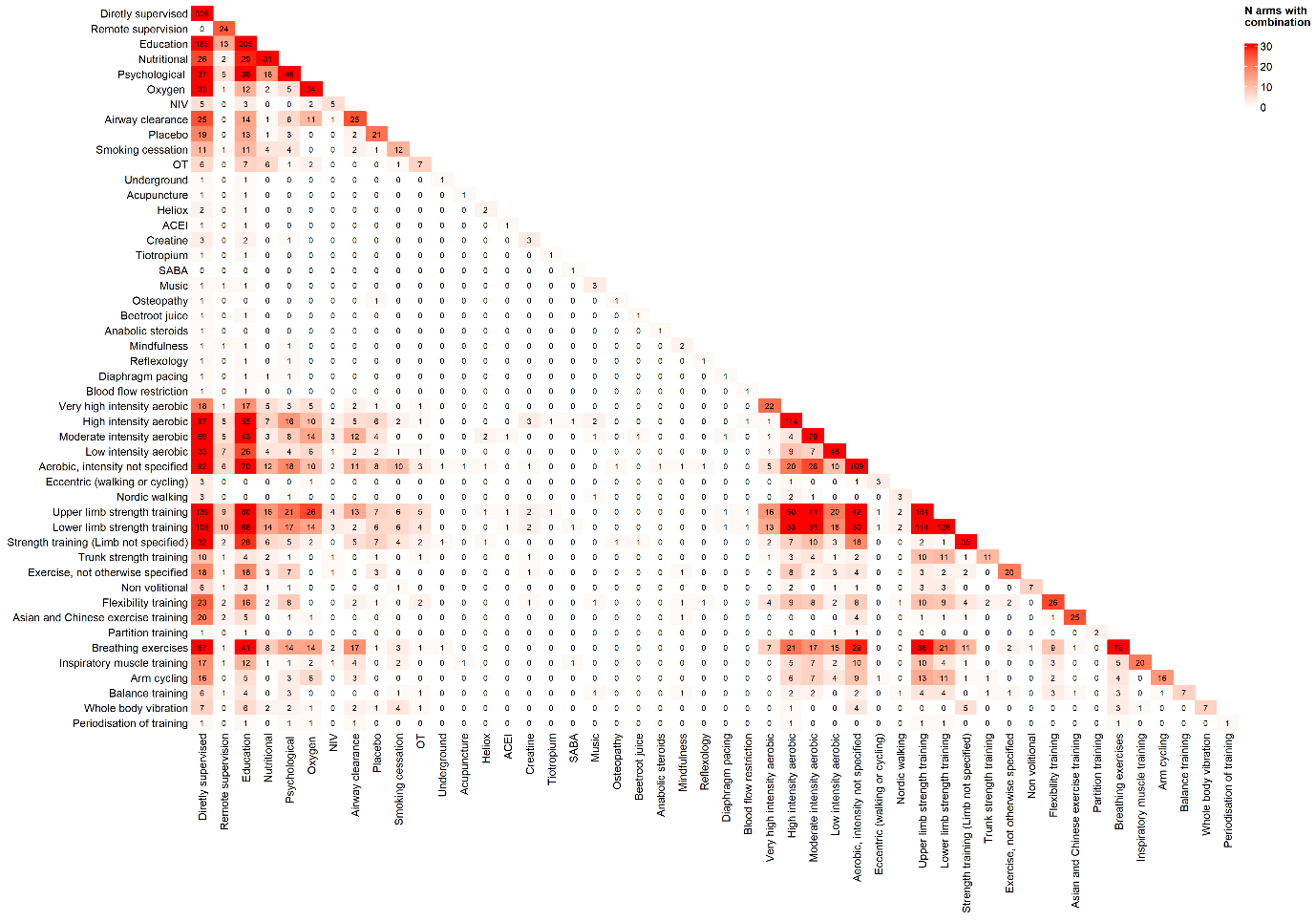


**Supplementary figure 13 – Interaction plot for Final model, quality of life.** Plot of frequency of combination of components in combined model with exercise capacity as outcome. NIV; non-invasive ventilation, OT; occupational therapy, B12: vitamin B12, ACEI; ACE inhibitor, PUFA: polyunsaturated fatty acids


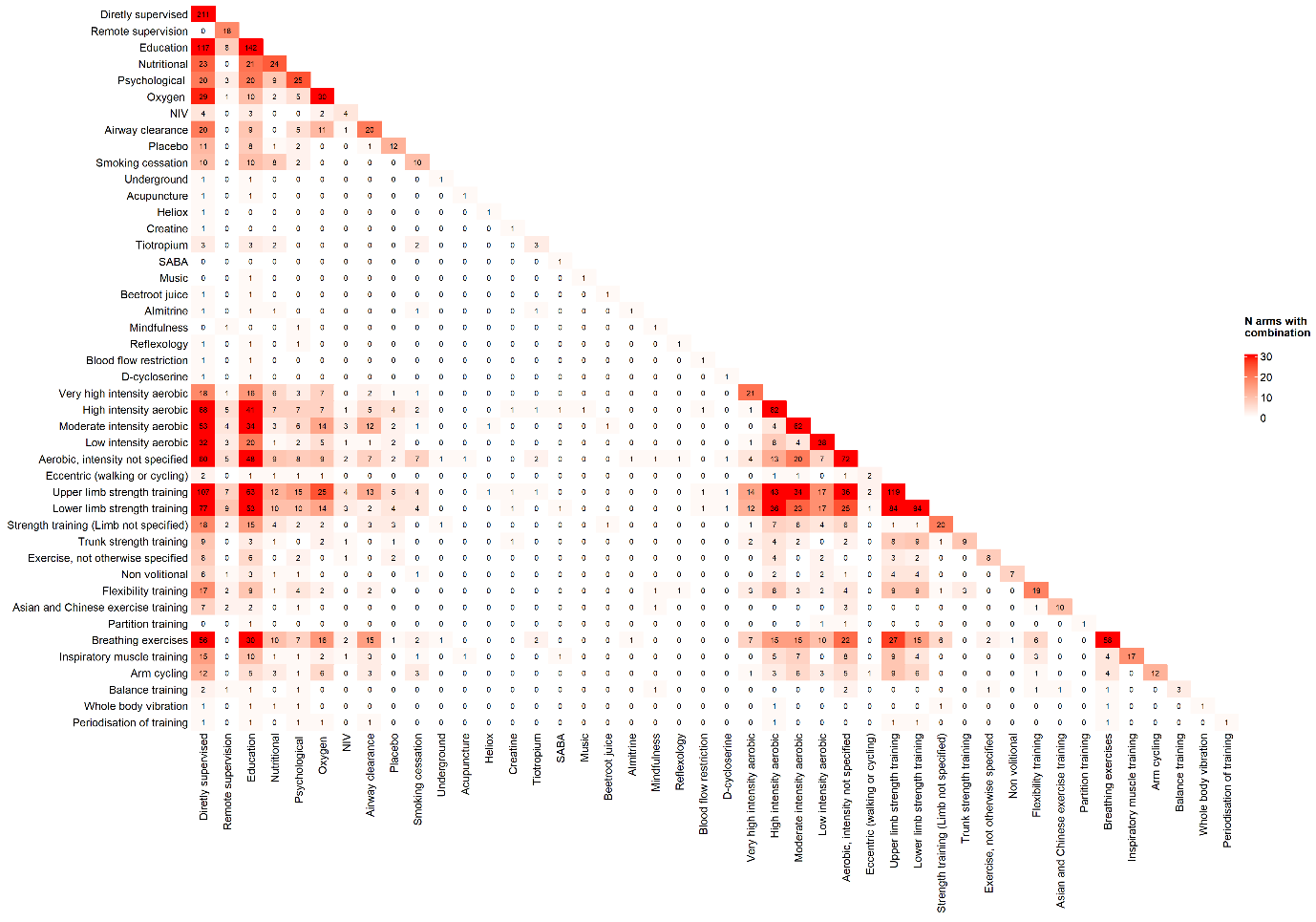


**Supplementary figure 14 – Interaction plot for Final model, breathlessness.** Plot of frequency of combination of components in combined model with exercise capacity as outcome. NIV; non-invasive ventilation, OT; occupational therapy, B12: vitamin B12, ACEI; ACE inhibitor, PUFA: polyunsaturated fatty acids

## Frequency of outcome measures

**Supplementary table 1** – Frequency of outcome measures

| **Exercise capacity** | | | | **Health related quality of life** | | | | **Breathlessness** | | |
| --- | --- | --- | --- | --- | --- | --- | --- | --- | --- | --- |
| **Outcome measure** | **Number of studies** | | | **Outcome measure** | **Number of studies** | | | **Outcome measure** | **Number of studies** | |
| VO2peak on incremental cycle test | | 72 |  | CRQ | | 85 |  | CRQ dyspnoea domain | | 84 |
| VO2peak on incremental treadmill test | | 11 |  | SGRQ | | 102 |  | MRC dyspnoea scale | | 18 |
| Peak workload (incremental cycle test) | | 16 |  | CAT | | 27 |  | mMRC dyspnoea scale | | 35 |
| Treadmill maximal walking speed | | 1 |  | MRF-26 | | 1 |  | Borg dyspnoea (during usual activities) | | 10 |
| Incremental shuttle walk test | | 31 |  | EQ5D | | 3 |  | VAS dyspnoea | | 4 |
| Constant work cycle test | | 12 |  | VQ11 | | 1 |  | Shortness of breath questionnaire | | 1 |
| Endurance shuttle walk test | | 3 |  | SF36 | | 1 |  | Baseline dyspnoea index/Transitional dyspnoea index | | 5 |
| 6-minute walk test | | 152 |  | Quality of Wellbeing scale | | 1 |  | UCSD-SOB | | 1 |
| 3-minute walk test | | 7 |  | Ferran's quality of life index | | 1 |  | Mahler's dyspnoea index | | 1 |
| 6-minute walk test/body weight | | 1 |  | COPD-PRO | | 1 |  |  | |  |
| estimated VO2peak from 6MWD | | 1 |  | SF-12 | | 1 |  |  | |  |
| endurance time from incremental treadmill test | | 1 |  |  | |  |  |  | |  |
| 2-minute walk test | | 1 |  |  | |  |  |  | |  |
| 12-minute walk test | | 3 |  |  | |  |  |  | |  |
| Modified shuttle walk test | | 1 |  |  | |  |  |  | |  |

To aid understanding, SMDs were also presented alongside conversion to the most common outcome measure following multiplication of the SMD by the pooled baseline SD for that measure. For 6MWD we used an SD of 83.7, for SGRQ we used an SD of 13.6 and for the CRQ dyspnoea domain we used an SD of 1.48 (based on the average of scores within the domain). CRQ: Chronic respiratory disease questionnaire, SGRQ: St George’s respiratory disease questionnaire, SF: Short form, CAT: COPD Assessment Test, MRF: Maugeri Foundation Respiratory Failure Questionnaire, EQ5D: European Quality of Life Five Dimension, VQ11: COPD specific HRQoL, COPD-PRO: COPD patient reported outcome, VAS: visual analogue scale, UCSD-SOB: University of California, San Diego Shortness of Breath Questionnaire

## Leverage plots

Supplementary figure **15 –** Leverage plots for interim model, exercise capacity

**Supplementary figure 16 –** Leverage plots for interim model, quality of life


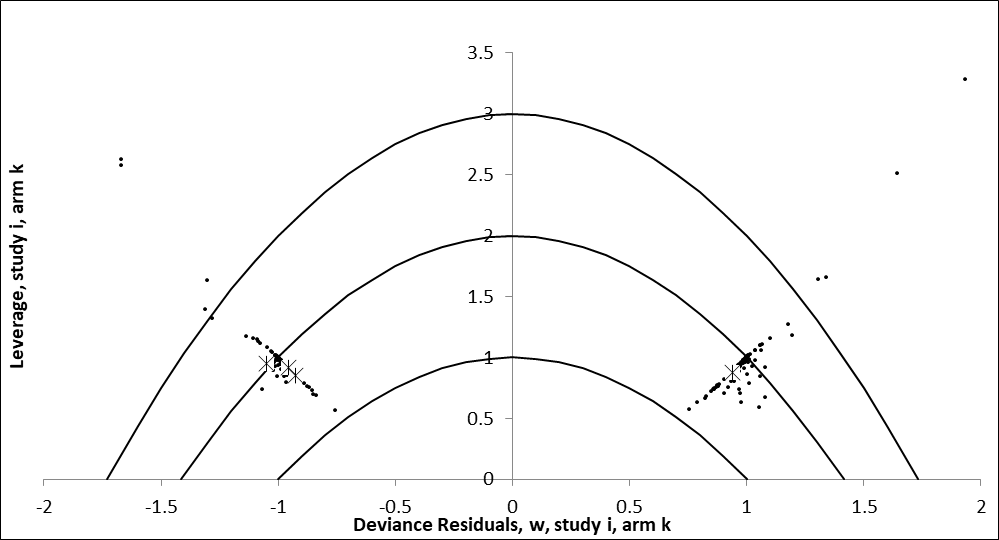


DIC=3

DIC=2

DIC=1

**Supplementary figure 17 –** Leverage plots for interim model, breathlessness

## Supplementary results - interim models


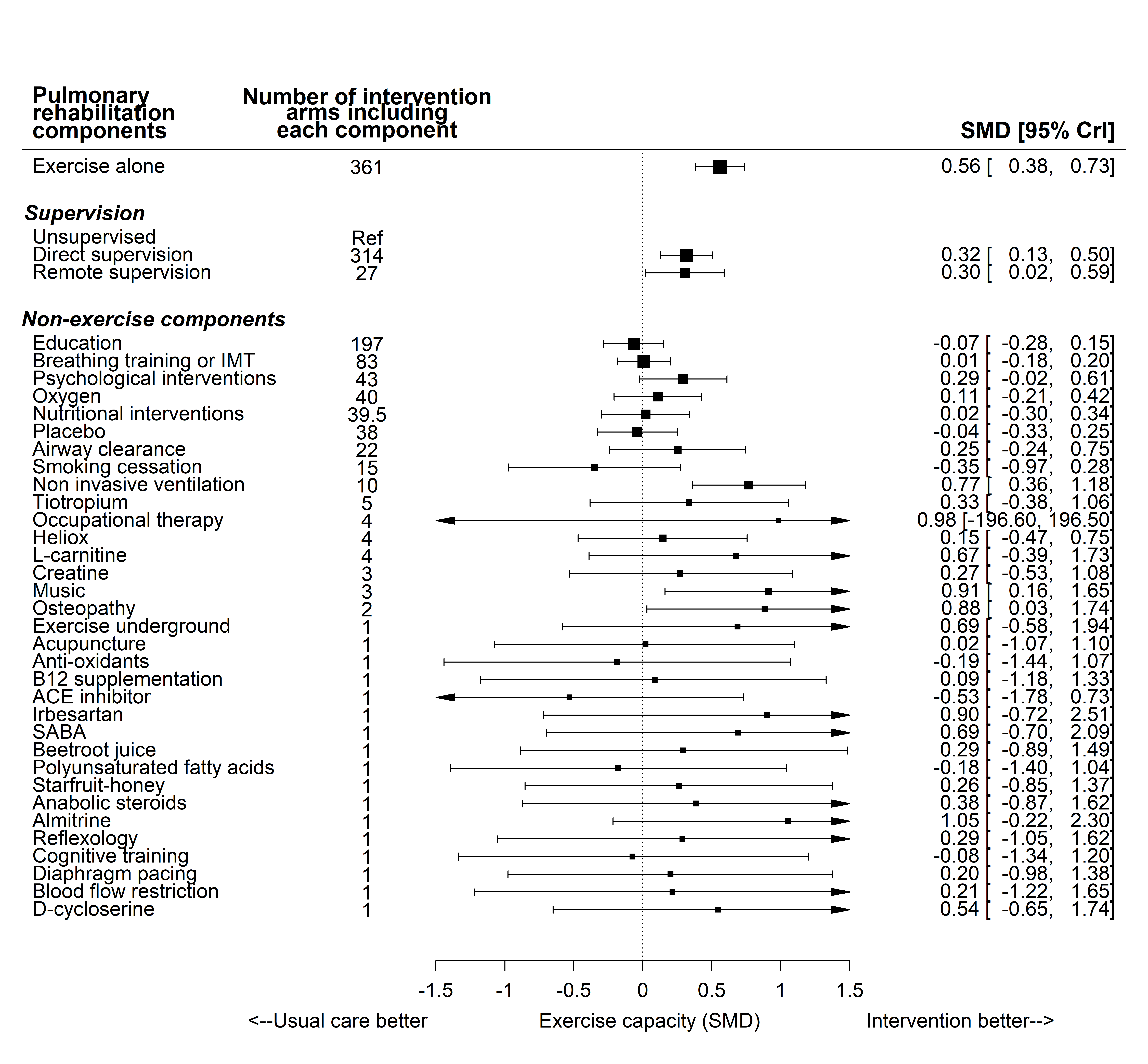


**Supplementary figure 18 –** Interim model with all components presented for outcome of exercise capacity


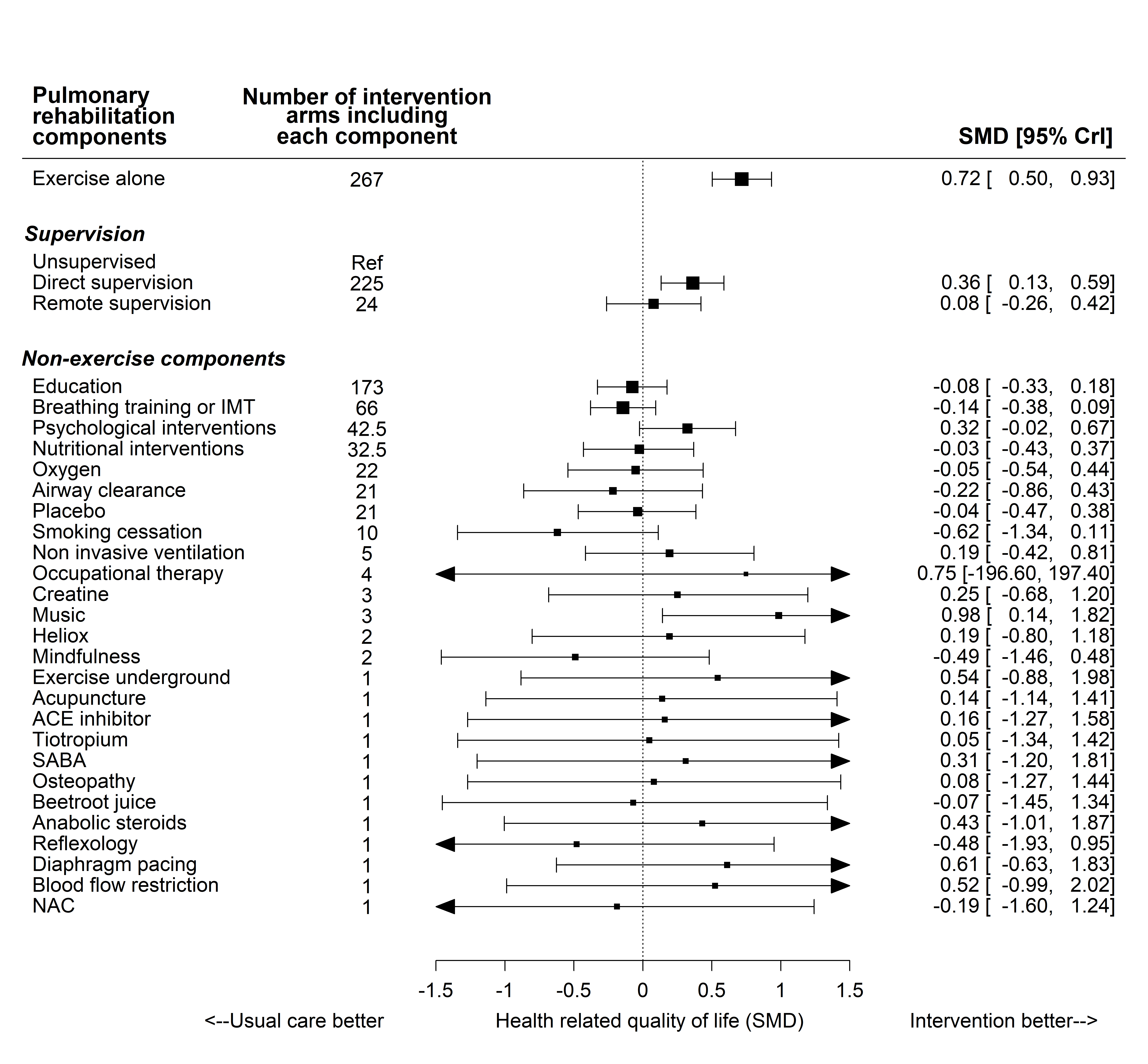


**Supplementary figure 19 –** Interim model with all components presented for outcome of quality of life


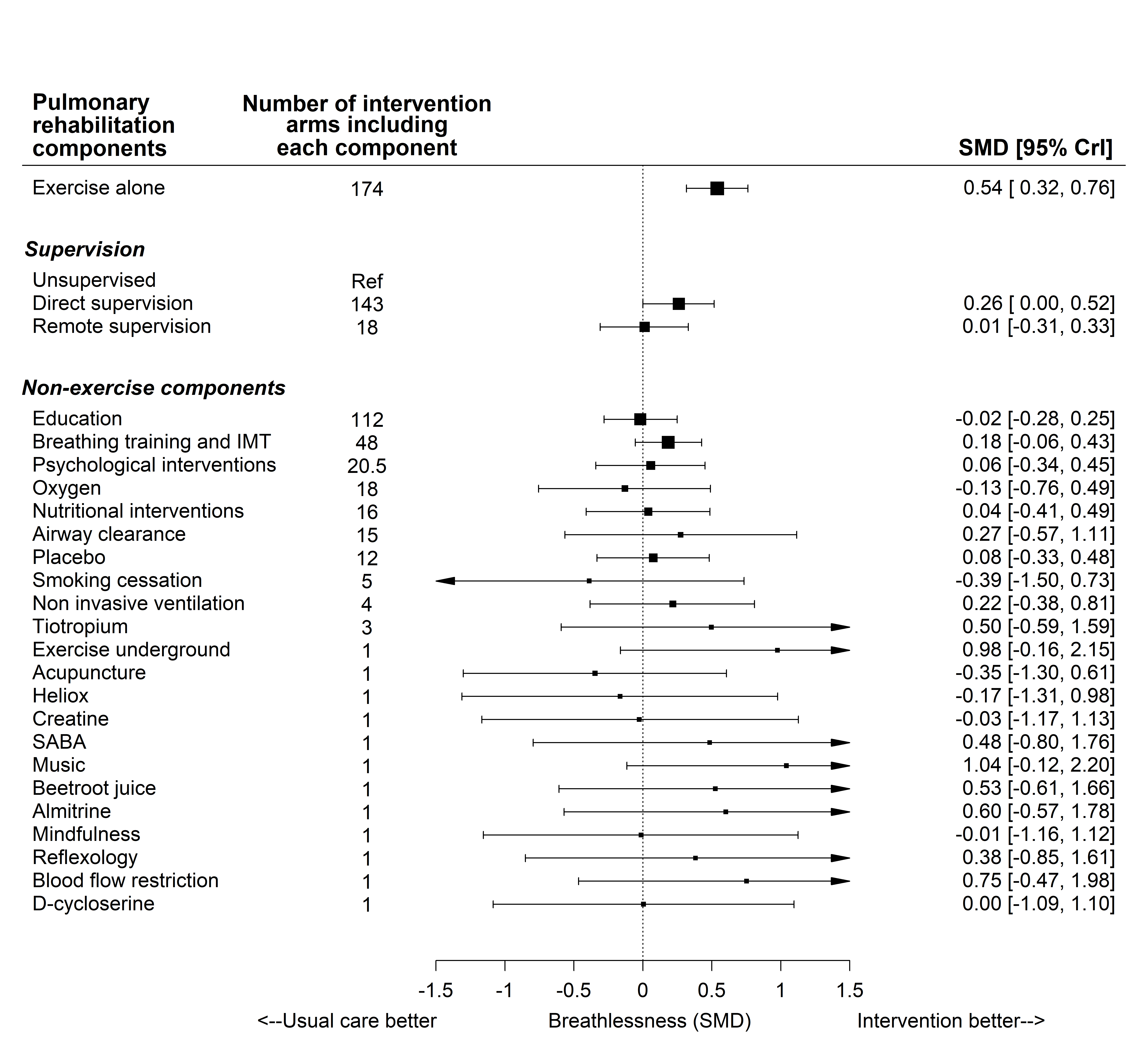


**Supplementary figure 20 –** Interim model with all components presented for outcome of breathlessness





**Supplementary figure 21 –** Final model with all components presented for outcome of exercise capacity





**Supplementary figure 22 –** Final model with all components presented for outcome of quality of life





**Supplementary figure 23 –** Final model with all components presented for outcome of breathlessness

## Supplementary results - interaction models

For components that were combined at least 20 times, we chose meaningful interactions through consensus to add to our additive model: exercise + education, exercise + nutritional interventions, exercise + breathing training, exercise + psychological interventions, exercise + direct supervision, exercise + remote supervision. However, none of these interactions could be estimated precisely as due to our inclusion criteria, studies that included for example breathing training alone would have been excluded from our analysis.

## Supplementary results – covariate models

**Supplementary table 2** – Covariate effect summary

| ***Component*** | ***Covariate*** | ***Exercise capacity*** | ***Quality of life*** | ***Breathlessness*** |
| --- | --- | --- | --- | --- |
| *Exercise* | Age (years) | Probably no effect: 0.01 (-0.03 to 0.05) | Possibly less effective at older Age (years): -0.03 (-0.08 to 0.02) | Less effective at older Age (years): -0.07 (0.01 to 0.13) |
|  | Sex (% male) | Probably no effect: 0.00 (-0.01 to 0.01) | Possibly less effective in female cohorts: 0.01 (-0.01 to 0.02) | Possibly less effective in female cohorts: 0.01 (0.00 to 0.02) |
|  | FEV_1_ (% predicted) | Probably no effect: 0.00 (-0.02 to 0.02) | Probably no effect: 0.01 (-0.01 to 0.03) | Possibly more effective in cohorts with higher FEV_1_: 0.01 (-0.01 to 0.04) |
|  | Baseline measure | Possibly more effective in cohorts with higher baseline exercise capacity: 0.11 (-0.07 to 0.28) | Probably no effect: 0.06 (-0.19 to 0.33) | Probably no effect: -0.17 (-0.48 to 0.14) |
|  | Income of country (World Bank categories) | Probably no effect: -0.03 (-0.30 to 0.24) | Less effective in high-income countries: -0.48 (-0.79 to -0.17) | Less effective in high-income countries: -0.43 (-0.77 to -0.09) |
|  | Programme length (weeks) | Probably no effect: -0.01 (-0.02 to 0.00) | Probably no effect: 0.00 (-0.02 to 0.01) | Probably no effect: -0.01 (-0.02 to 0.01) |
|  | Publication year | Probably no effect: 0.01 (-0.01 to 0.03) | Possibly more effective in more recent publications: 0.02 (-0.04 to 0.01) | Probably no effect: 0.01 (-0.02 to 0.03) |
| *In-person supervision* | Age (years) | Probably no effect: 0.01 (-0.06 to 0.03) | Probable no effect: 0.02 (-0.04 to 0.07) | Possibly more effective at older Age (years): 0.05 (-0.03 to 0.13) |
|  | Sex (% male) | Probably no effect: 0.00 (-0.01 to 0.01) | Possibly less effective in female cohorts: 0.01 (0.00 to 0.02) | Probably no effect: 0.00 (-0.01 to 0.01) |
|  | FEV_1_ (% predicted) | Probably no effect: 0.00 (-0.02 to 0.02) | Probably no effect: 0.01 (-0.01 to 0.04) | Possibly less effective in cohorts with higher FEV_1_: -0.03 (-0.06 to 0.00) |
|  | Baseline measure | Possibly less effective in cohorts with higher baseline exercise capacity: -0.15 (-0.37 to 0.07) | Possibly less effective in cohorts with better baseline quality of life: -0.28 (-0.66 to 0.10) | Possibly more effective in cohorts with less baseline breathlessness: 0.30 (-0.10 to 0.70) |
|  | Income of country (World Bank categories) | Probably no effect: 0.00 (-0.32 to 0.31) | Probably no effect: -0.19 (-0.57 to 0.19) | Possibly less effective in high income countries: -0.23 (-0.76 to 0.23) |
|  | Programme length (weeks) | Probably no effect: 0.01 (-0.01 to 0.03) | Probably no effect: 0.01 (-0.01 to 0.04) | Probably no effect: 0.00 (-0.03 to 0.03) |
|  | Publication year | Probably no effect: -0.01 (-0.03 to 0.02) | Possibly more effective in more recent publications: 0.03 (0.00 to 0.06) | Probably no effect: -0.01 (-0.03 to 0.04) |
| *Remote supervision* | Age (years) | Possibly less effective at older Age (years): -0.04 (-0.11 to 0.04) | Probably no effect: 0.01 (-0.07 to 0.10 | Possibly more effective at older Age (years): 0.08 (-0.01 to 0.17) |
|  | Sex (% male) | Less effective in female cohorts: 0.02 (0.01 to 0.04) | Possibly less effective in female cohorts: 0.02 (0.00 to 0.04) | Probably no effect: 0.00 (-0.02 to 0.02) |
|  | FEV_1_ (% predicted) | Possibly less effective in cohorts with higher FEV_1_: -0.01 (-0.04 to 0.01) | Probably no effect: 0.00 (-0.04 to 0.03) | Possibly less effective in cohorts with higher FEV_1_: -0.02 (-0.05 to 0.02) |
|  | Baseline measure | Probably no effect: -0.07 (-0.41 to 0.27) | Probably no effect: -0.07 (-0.77 to 0.63) | Possibly less effective in cohorts with less baseline breathlessness -0.62 (-1.72 to 0.49) |
|  | Income of country (World Bank categories) | Less effective in high-income countries: -0.76 (-1.36 to -0.17) | Possibly more effective in high income countries: 0.36 (-0.29 to 1.00 | Possibly more effective in high income countries: 0.66 (-0.20 to 1.51) |
|  | Programme length (weeks) | Probably no effect: -0.02 (-0.05 to 0.01) | Probably no effect: 0.00 (-0.04 to 0.03) | Probably no effect: 0.00 (-0.03 to 0.03) |
|  | Publication year | Probably no effect: -0.02 (-0.08 to 0.04) | Probably no effect: -0.02 (-0.08 to 0.05) | Probably no effect: -0.01 (-0.08 to 0.06) |
| *Education* | Age (years) | Possibly less effective at older Age (years): -0.03 (-0.09 to 0.03) | Possibly more effective at older Age (years): 0.03 (-0.04 to 0.10) | Probably no effect: 0.00 (-0.08 to 0.08) |
|  | Sex (% male) | Probably no effect: 0.01 (-0.01 to 0.02) | Possibly more effective in female cohorts: -0.01 (-0.02 to 0.01) | Probably no effect: 0.00 (-0.01 to 0.02) |
|  | FEV_1_ (% predicted) | Possibly more effective in cohorts with higher FEV_1_: 0.01 (-0.01 to 0.04) | Possibly more effective in cohorts with higher FEV_1_: 0.01 (-0.01 to 0.04) | Probably no effect: 0.00 (-0.04 to 0.03) |
|  | Baseline measure | Probably no effect: -0.07 (-0.23 to 0.08) | Possibly more effective in cohorts with better baseline quality of life: 0.20 (-0.25 to 0.64) | Probably no effect: 0.06 (-0.48 to 0.62) |
|  | Income of country (World Bank categories) | Probably no effect: -0.11 (-0.55 to 0.33) | Probably no effect: -0.07 (-0.56 to 0.42) | Probably no effect: -0.14 (-0.81 to 0.52) |
|  | Programme length (weeks) | Probably no effect: -0.01 (-0.03 to 0.01) | Probably no effect: 0.00 (-0.02 to 0.03 | Probably no effect: -0.01 (-0.05 to 0.03) |
|  | Publication year | Probably no effect: 0.00 (-0.02 to 0.03) | Probably no effect: 0.00 (-0.03 to 0.04) | Possibly less effective in more recent publications: -0.02 (-0.05 to 0.02) |
| *Breathing exercises and Inspiratory muscle training* | Age (years) | Probably no effect: 0.00 (-0.04 to 0.04) | Possibly more effective at older Age (years): 0.03 (-0.02 to 0.08) | Probably no effect: 0.00 (-0.06 to 0.06) |
|  | Sex (% male) | Probably no effect: 0.00 (-0.01 to 0.01) | Possibly more effective in female cohorts: -0.01 (-0.02 to 0.00) | Probably no effect: 0.00 (-0.01 to 0.01) |
|  | FEV_1_ (% predicted) | Possibly less effective in cohorts with higher FEV_1_: -0.01 (-0.04 to 0.01) | Possibly less effective in cohorts with higher FEV_1_: -0.02 (-0.05 to 0.01) | Probably no effect: 0.01 (-0.02 to 0.05) |
|  | Baseline measure | Possible less effective in cohorts with higher baseline exercise capacity: -0.13 (-0.41 to -0.14) | Probably no effect: 0.06 (-0.19 to 0.37) | Possibly more effective in cohorts with less baseline breathlessness: 0.31 (-0.20 to 0.82) |
|  | Income of country (World Bank categories) | Probably no effect: 0.02 (-0.30 to 0.34) | Possibly more effective in high-income countries: 0.29 (-0.07 to 0.66) | Possibly more effective in high-income countries: 0.25 (-0.18 to 0.67) |
|  | Programme length (weeks) | Probably no effect: 0.00 (-0.02 to 0.02) | Probably no effect: -0.01 (-0.04 to 0.01) | Possibly less effect in longer programmes: -0.03 (-0.06 to 0.01) |
|  | Publication year | Probably no effect: -0.01 (-0.04 to 0.01) | Possibly less effective in more recent publications: -0.02 (-0.06 to 0.02) | Possibly less effective in more recent publications: -0.02 (-0.06 to 0.02) |
| *Psychological interventions* | Age (years) | Probably no effect: 0.00 (-0.08 to 0.08) | Probably no effect: 0.02 (-0.07 to 0.10) | Probably no effect: 0.05 (-0.08 to 0.17) |
|  | Sex (% male) | Probably no effect: 0.01 (-0.01 to 0.03) | Possibly more effective in female cohorts: -0.02 (-0.04 to 0.00) | Possibly more effective in female cohorts: -0.02 (-0.04 to 0.00) |
|  | FEV_1_ (% predicted) | Probably no effect: -0.06 (-0.06 to 0.04) | Probably no effect: -0.01 (-0.07 to 0.05) | Probably no effect: 0.00 (-0.08 to 0.08) |
|  | Baseline measure | Probably no effect: -0.08 (-0.27 to 0.10) | Possibly less effective in cohorts with better baseline quality of life : -0.31 (-1.18 to 0.61) | Probably no effect: 0.22 (-0.80 to 1.24) |
|  | Income of country (World Bank categories) | More effective in high-income countries: 0.42 (0.01 to 0.84) | Possibly less effective in high-income countries: -1.06 (-2.22 to 0.11) | Probably no effect: 1.30 (-1.54 to 4.10) |
|  | Programme length (weeks) | Probably no effect: -0.01 (-0.02 to 0.04) | Probably no effect: -0.01 (-0.05 to 0.03) | Probably no effect: 0.00 (-0.21 to 0.20) |
|  | Publication year | Probably no effect: -0.01 (-0.05 to 0.02) | Possibly less effective in more recent publications: -0.04 (-0.08 to 0.00) | Possibly less effective in more recent publications: -0.04 (0.00 to 0.09) |
| *Nutritional interventions* | Age (years) | Possibly more effective at older Age (years): 0.08 (-0.04 to 0.20) | Possibly less effective at older Age (years): -0.06 (-0.19 to 0.08) | Possible more effective at older age: 0.09 (-0.08 to 0.26) |
|  | Sex (% male) | Probably no effect: 0.00 (-0.02 to 0.02) | Possibly less effective in female cohorts: 0.02 (0.00 to 0.04) | Probably no effect: 0.00 (-0.03 to 0.02) |
|  | FEV_1_ (% predicted) | Possibly less effective in cohorts with higher FEV_1_: -0.02 (-0.06 to 0.02) | Probably no effect: 0.02 (-0.05 to 0.08) | Probably no effect: -0.03 (-0.12 to 0.07) |
|  | Baseline measure | Possibly more effective in cohorts with higher baseline exercise capacity: 0.25 (-0.35 to 0.86) | Possible less effective in cohorts with better baseline quality of life:  -0.91 (-2.07 to 0.40) | Possibly less effective in cohorts with less baseline breathlessness: -0.82 (-2.05 to 0.37) |
|  | Income of country (World Bank categories) | Possibly more effective in high income countries: 0.89 (-0.23 to 2.03) | Probably no effect: 0.06 (-1.25 to 1.14) | Probably no effect: 0.16 (-1.55 to 1.91) |
|  | Programme length (weeks) | Probably no effect: 0.01 (-0.03 to 0.04) | Probably no effect: 0.02 (-0.02 to 0.06) | Probably no effect: 0.02 (-0.08 to 0.12) |
|  | Publication year | Probably no effect: -0.01 (-0.05 to 0.03) | Possibly more effective in more recent publications : 0.03 (-0.02 to 0.08) | Probably no effect: 0.02 (-0.04 to 0.08) |

**
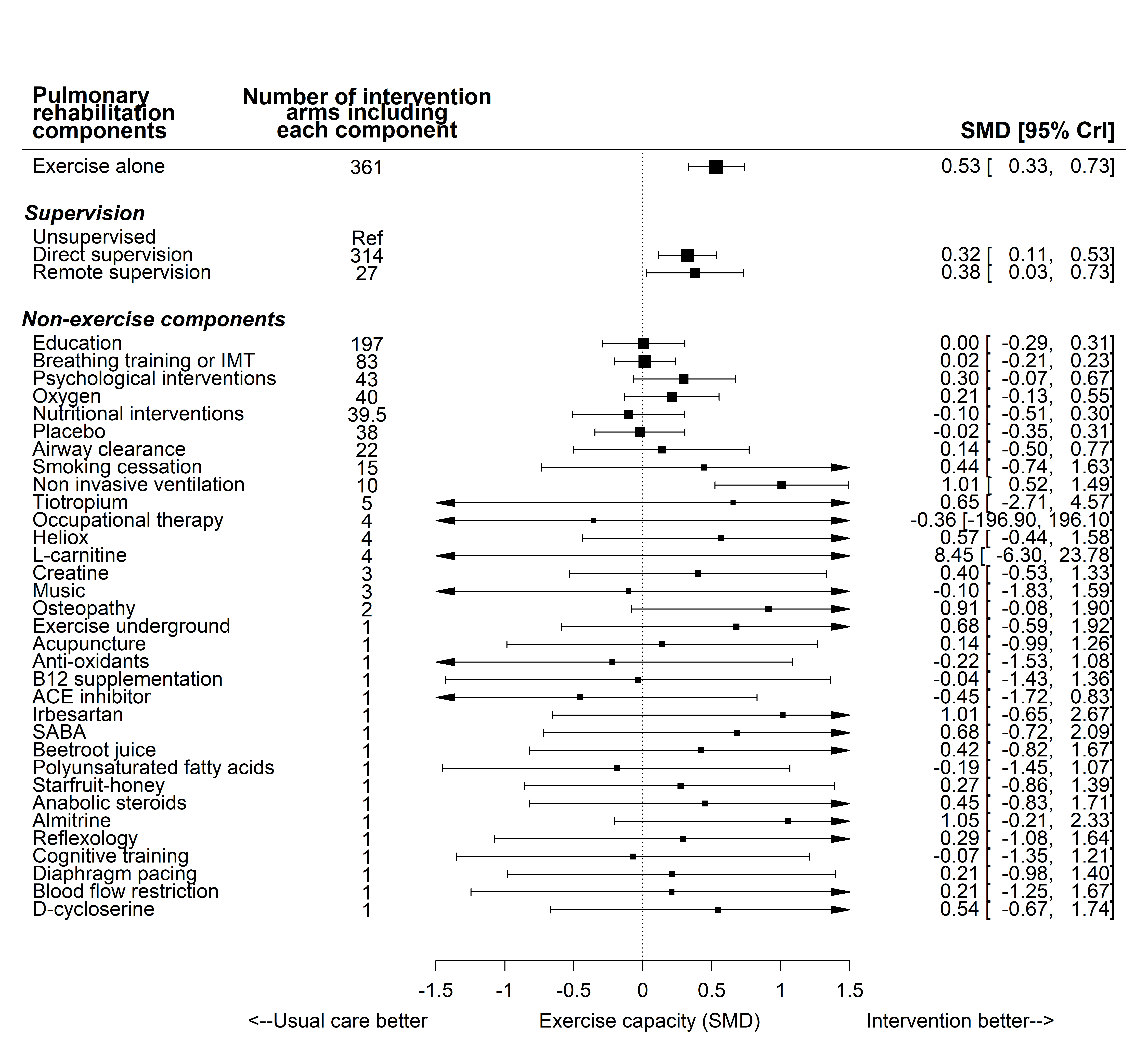
**

**Supplementary figure 24 –** Interim model centred for mean cohort age for outcome of exercise capacity

**
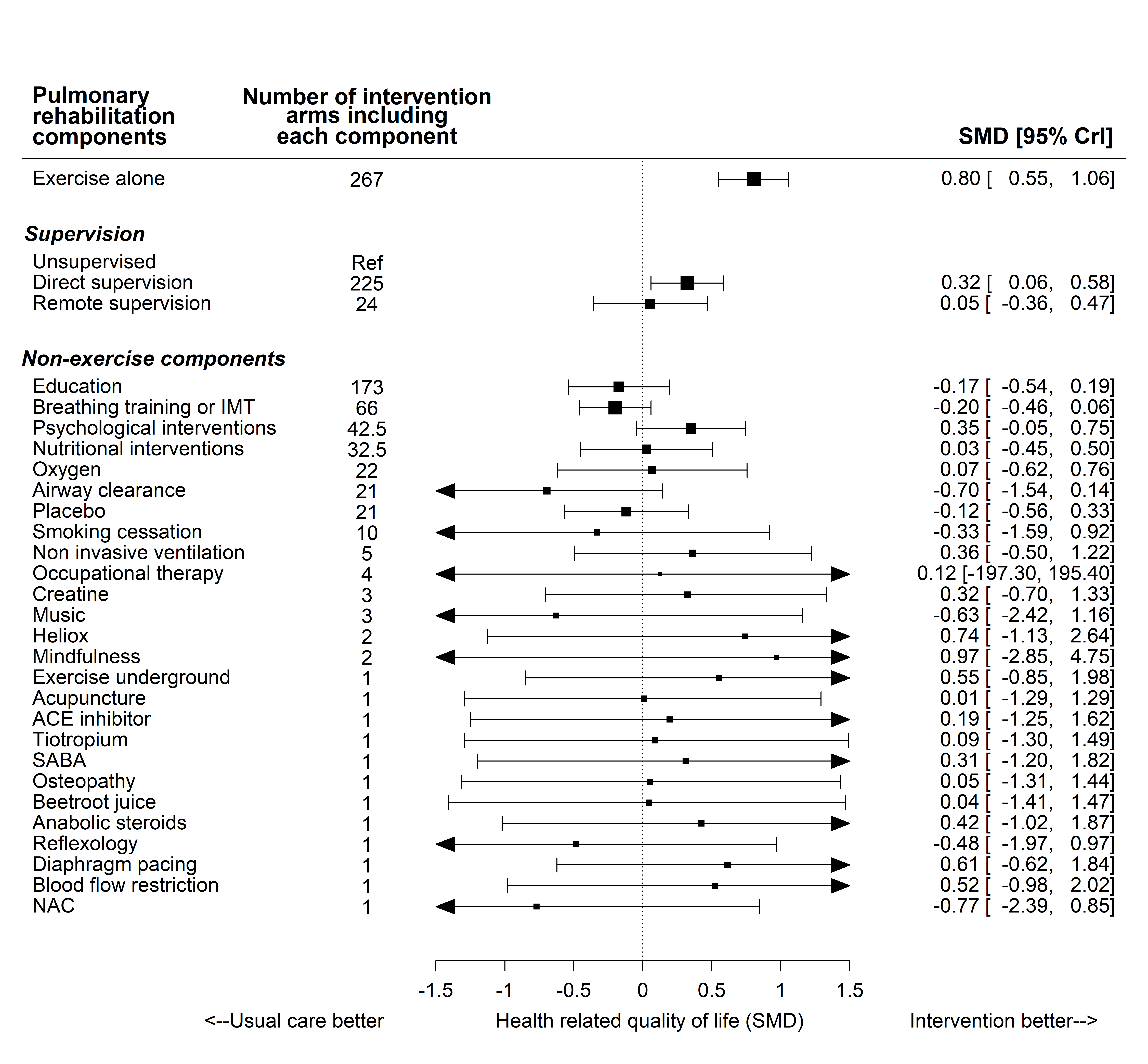
**

**Supplementary figure 25 –** Interim model centred for mean cohort age for outcome of quality of life

**
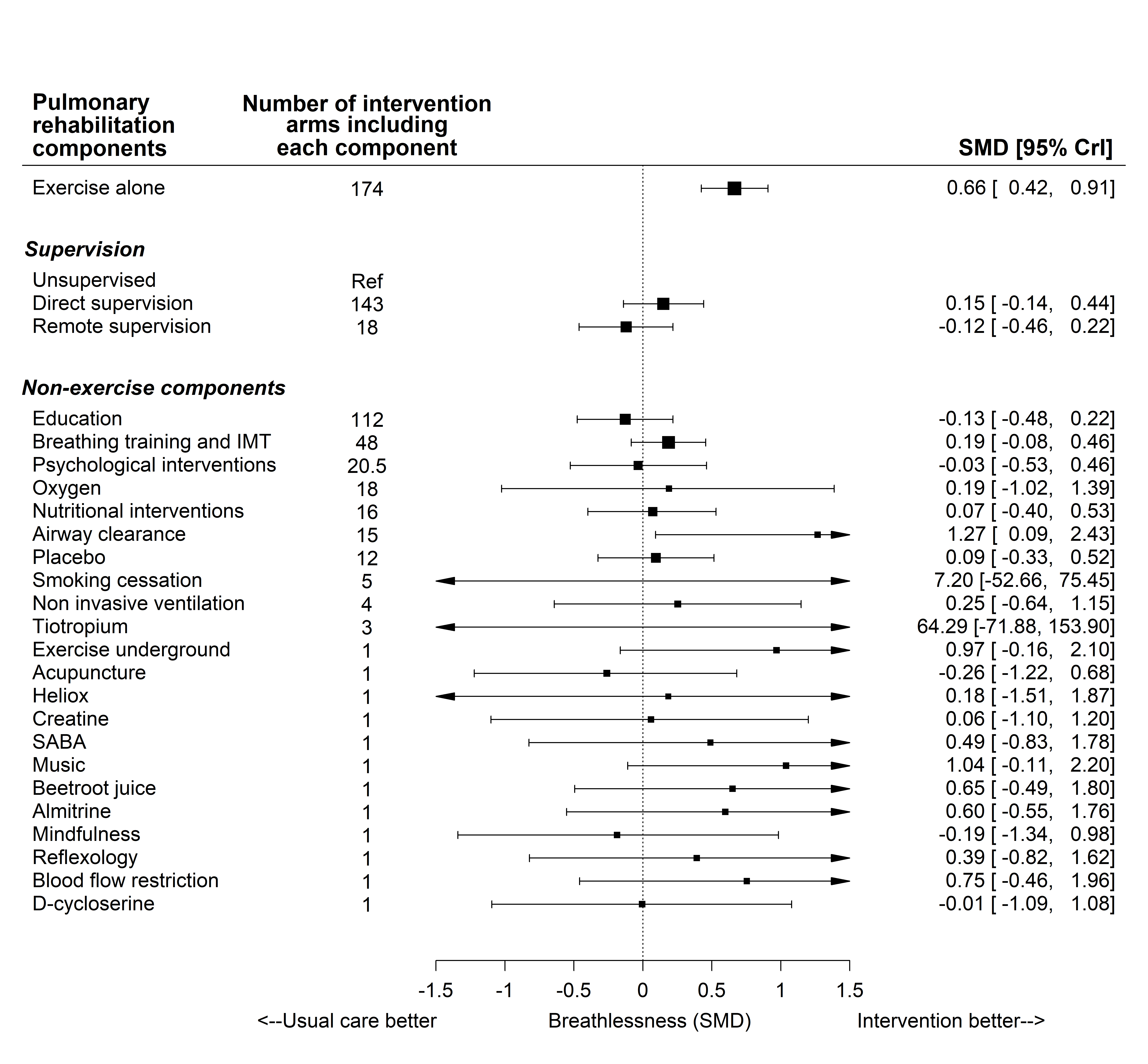
**

**Supplementary figure 26 –** Interim model centred for mean cohort age for outcome of breathlessness

**
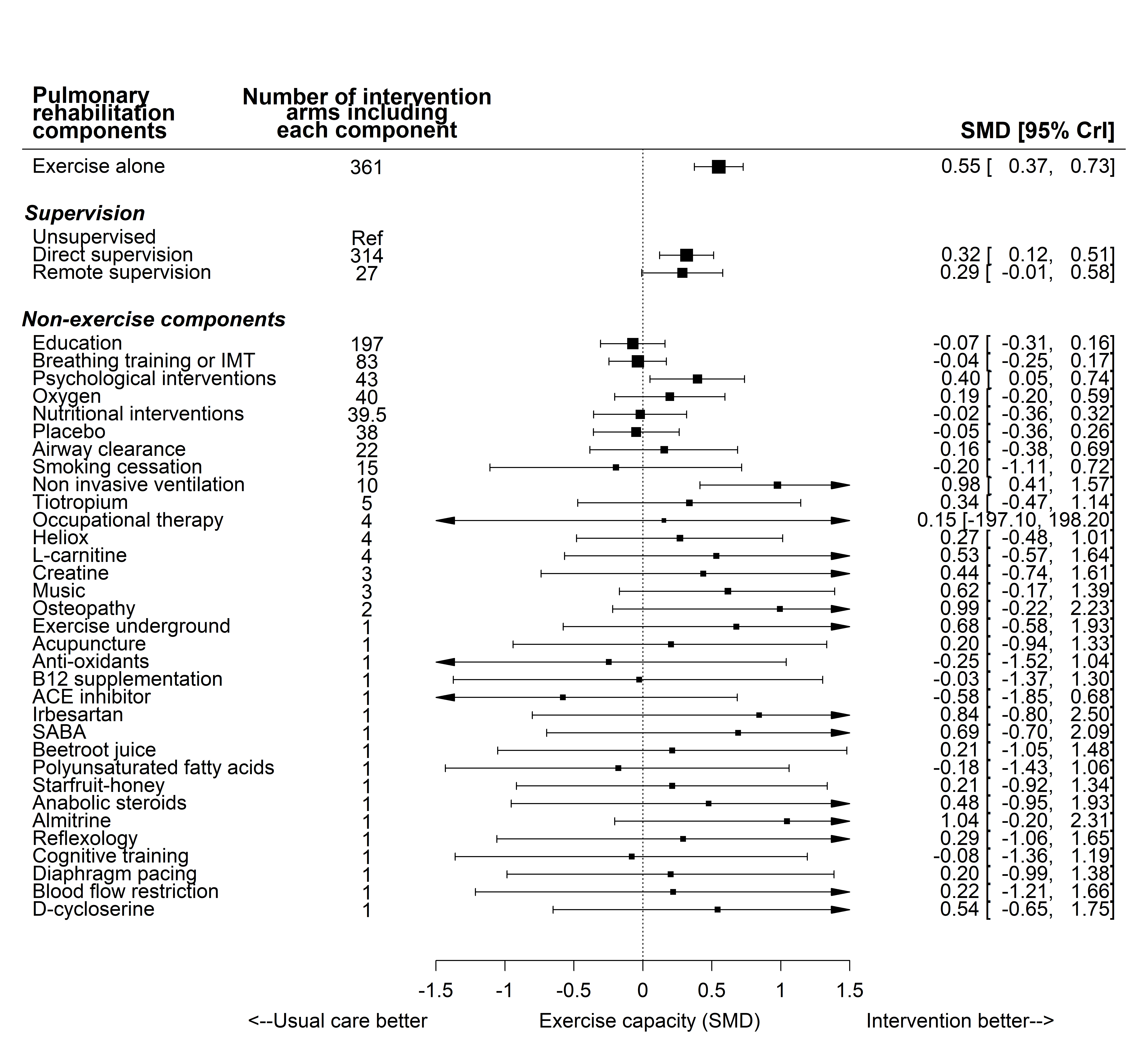
**

**Supplementary figure 27 –** Interim model additive model centred for proportion of males in a cohort for outcome of exercise capacity

**
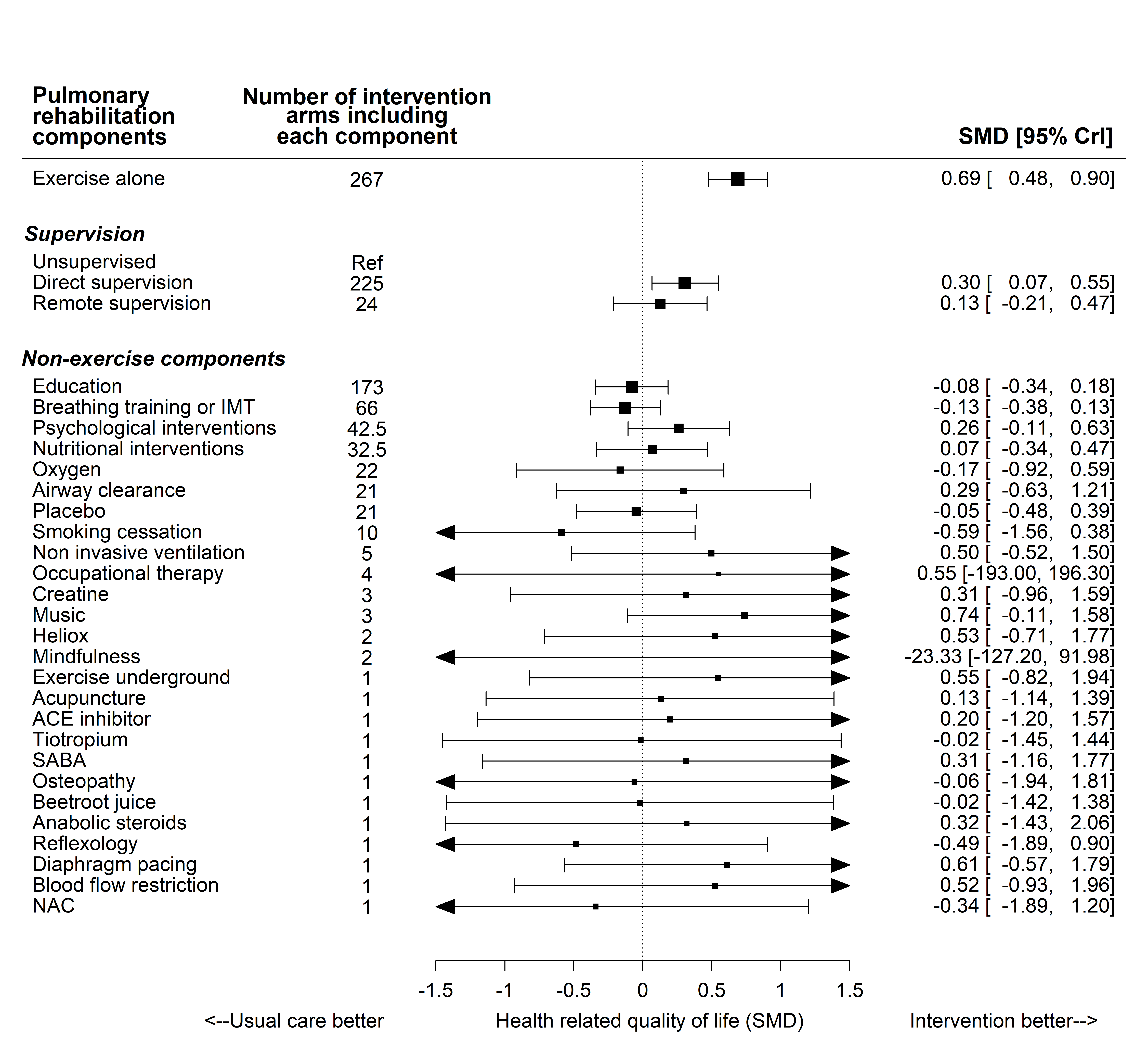
**

**Supplementary figure 28 –** Interim model additive model centred for proportion of males in a cohort for outcome of quality of life

**
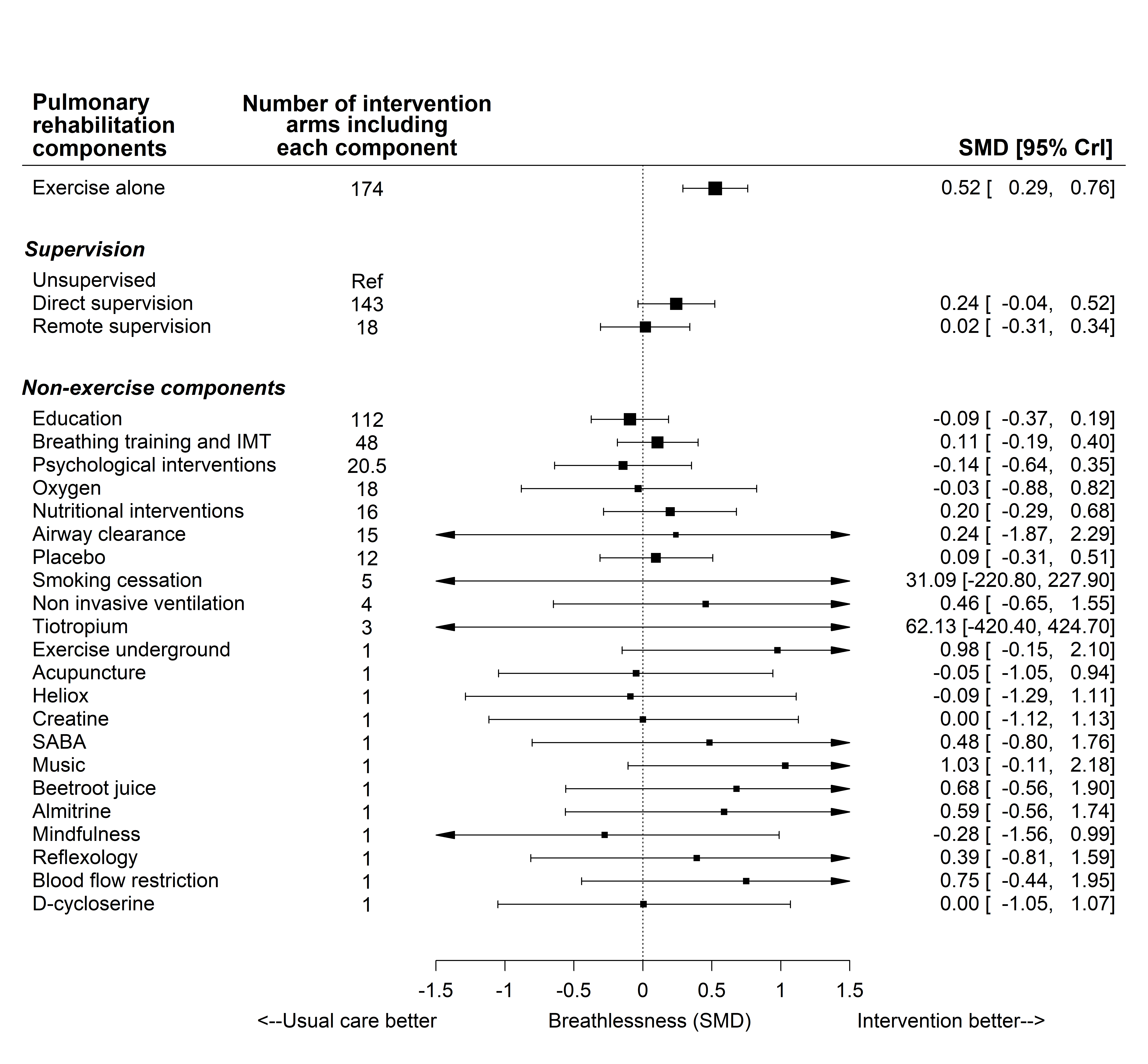
**

**Supplementary figure 29 –** Interim model additive model centred for proportion of males in a cohort for outcome of breathlessness

**
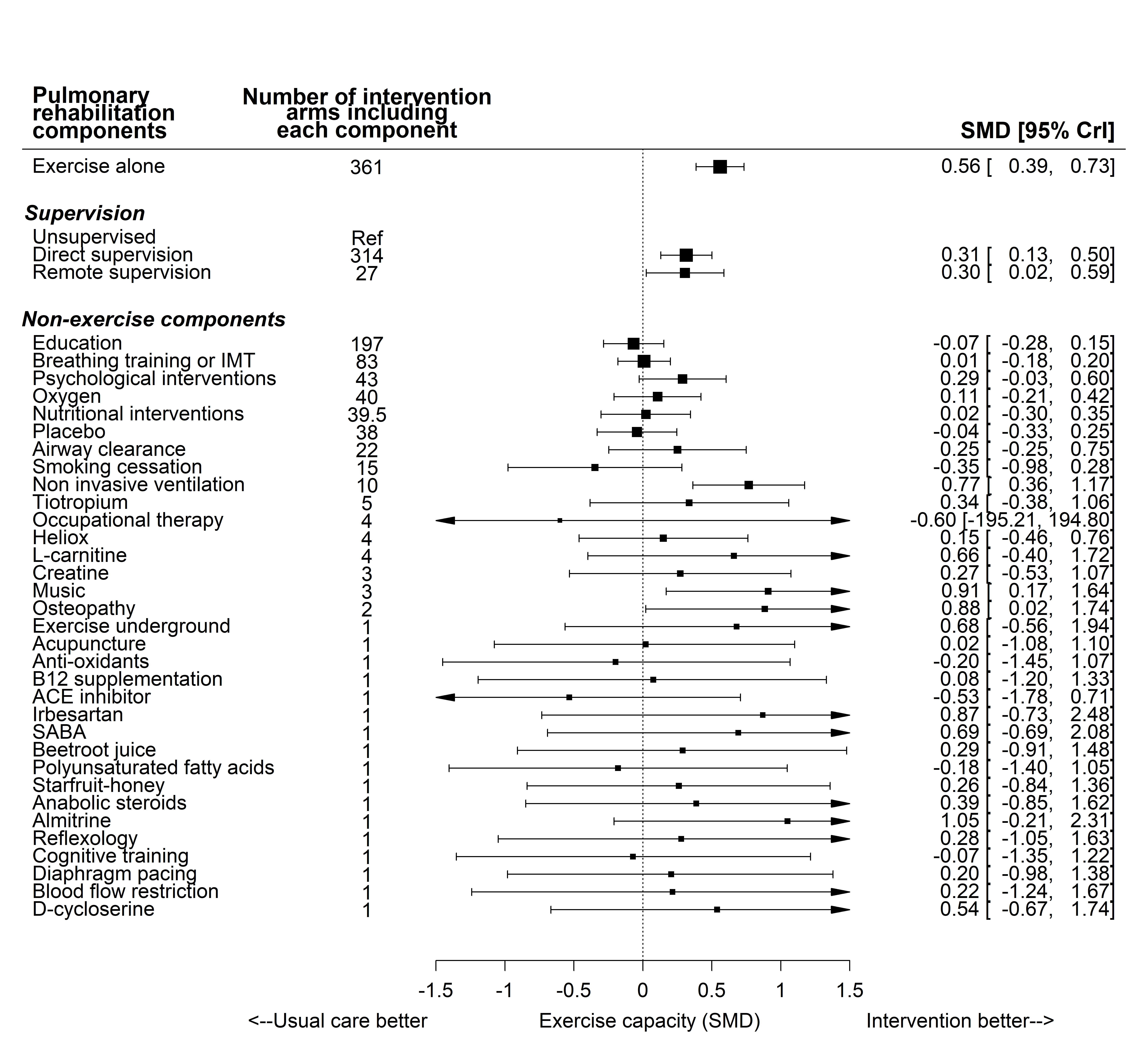
**

**Supplementary figure 30 –** Interim model additive model centred for mean baseline standardised exercise capacity

**
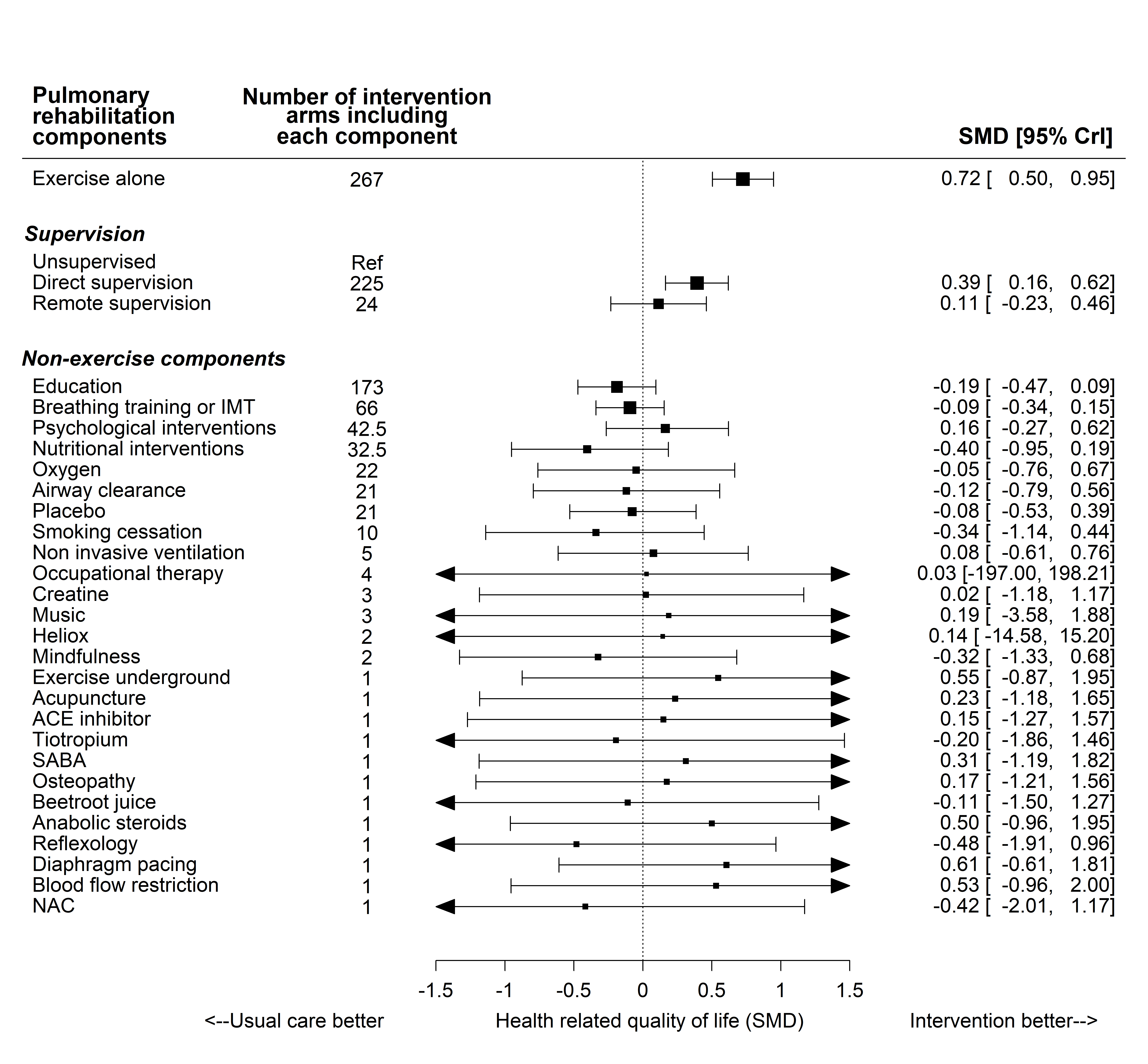
**

**Supplementary figure 31 –** Interim model additive model centred for mean baseline standardised quality of life

**
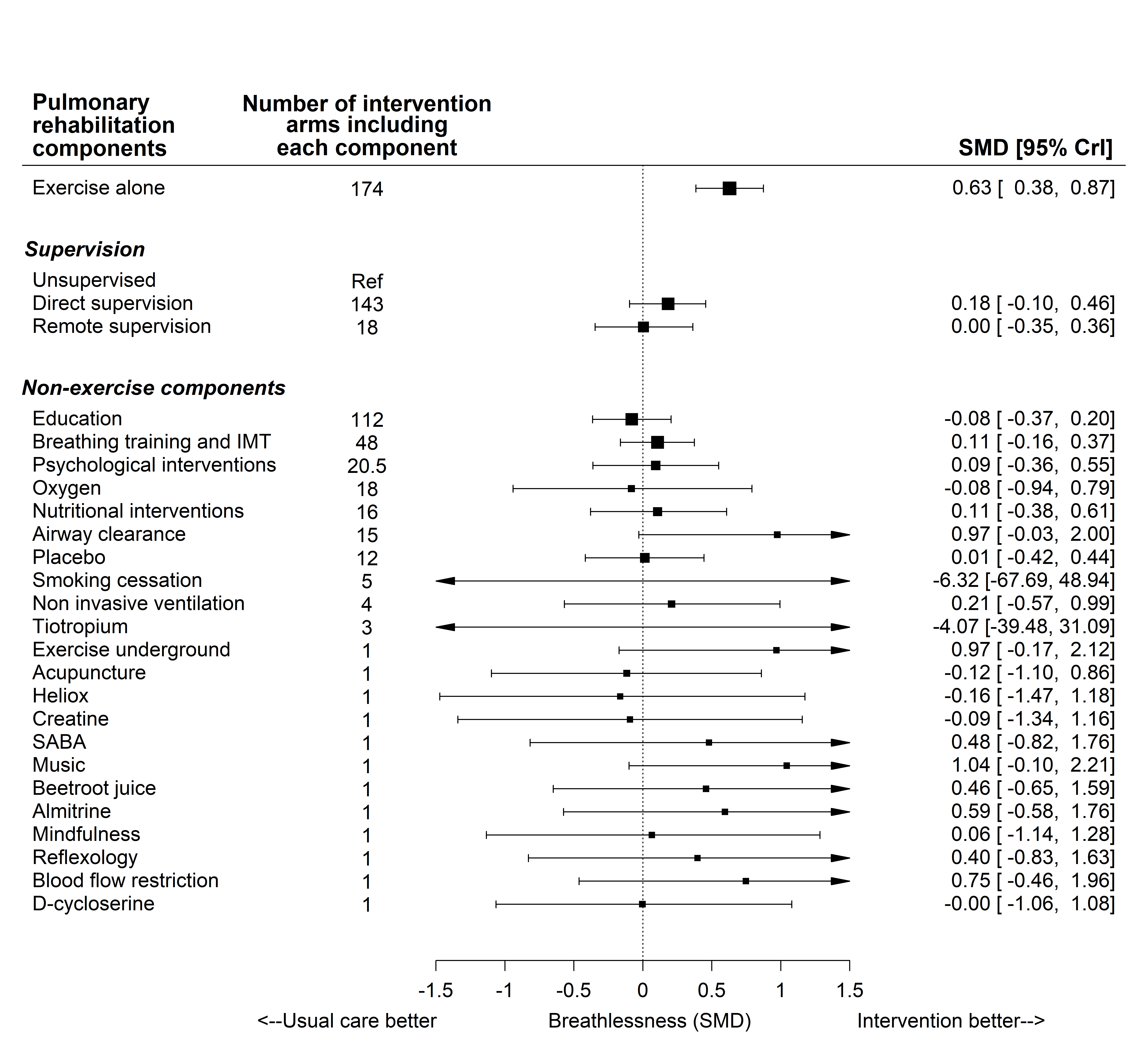
**

**Supplementary figure 32 –** Interim model additive model centred for mean baseline standardised breathlessness

**
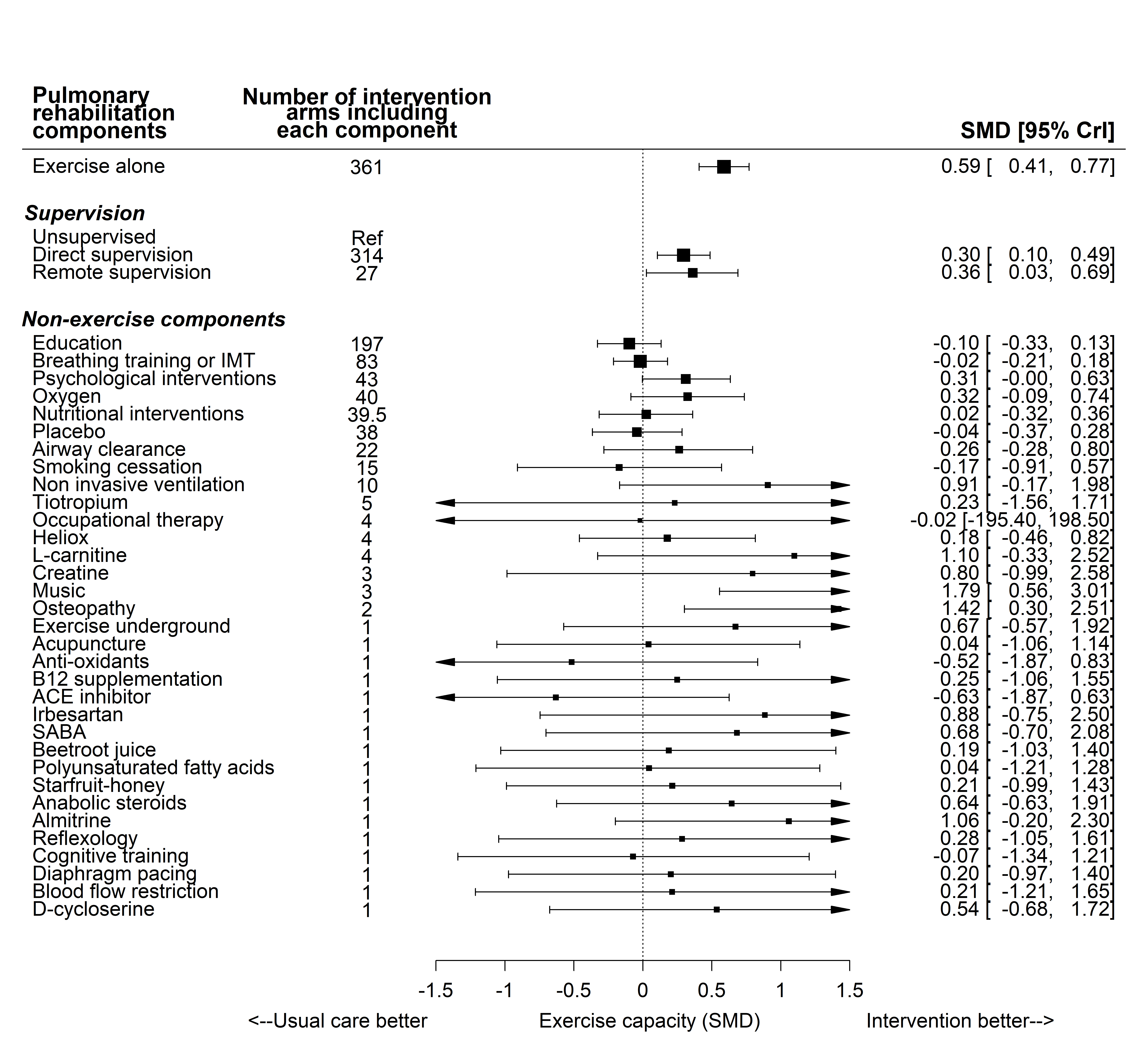
**

**Supplementary figure 33 –** Interim model additive model centred for mean baseline FEV_1_ for outcome of exercise capacity

**
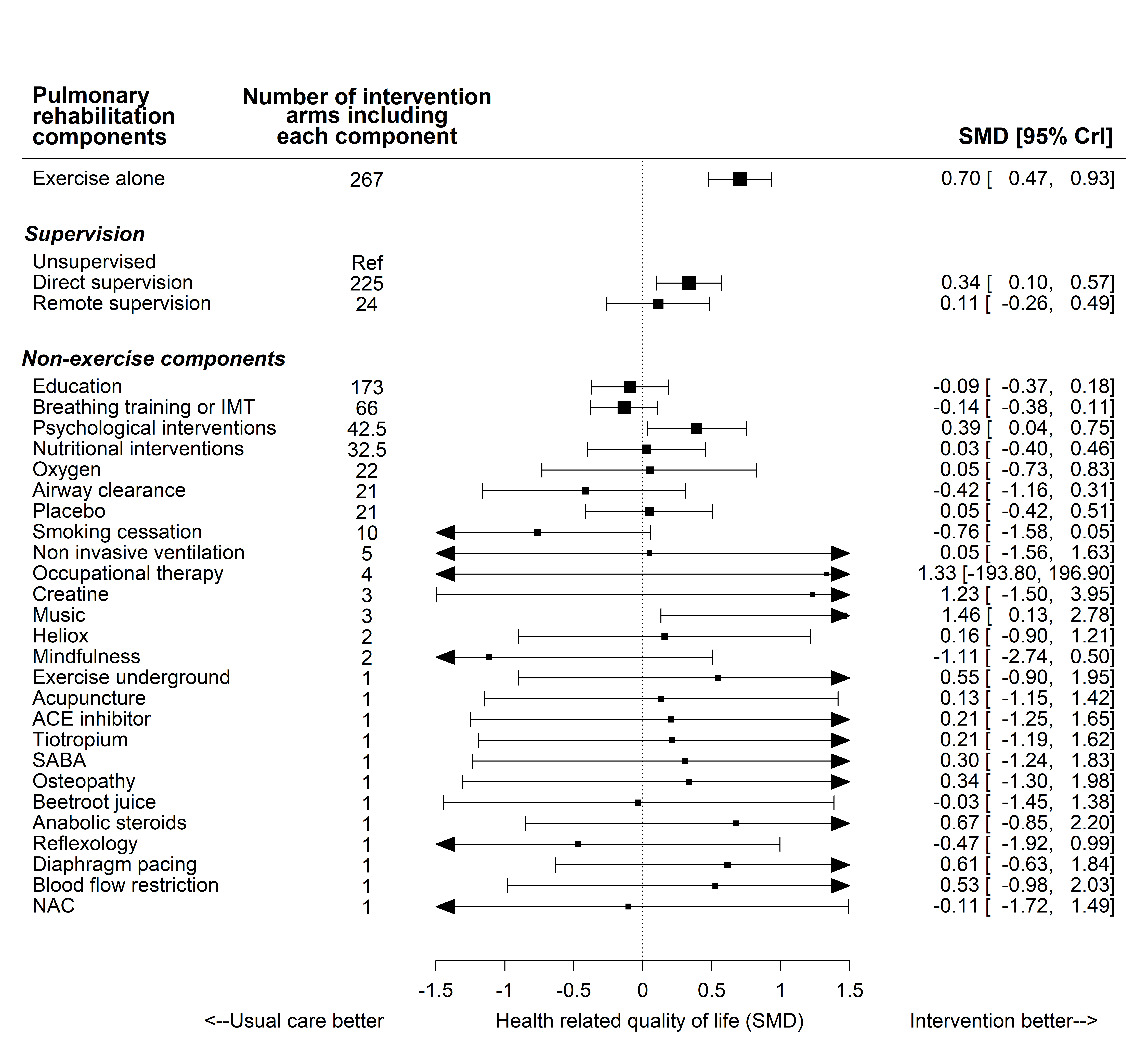
**

**Supplementary figure 34 –** Interim model additive model centred for mean baseline FEV_1_ for outcome of quality of life

**
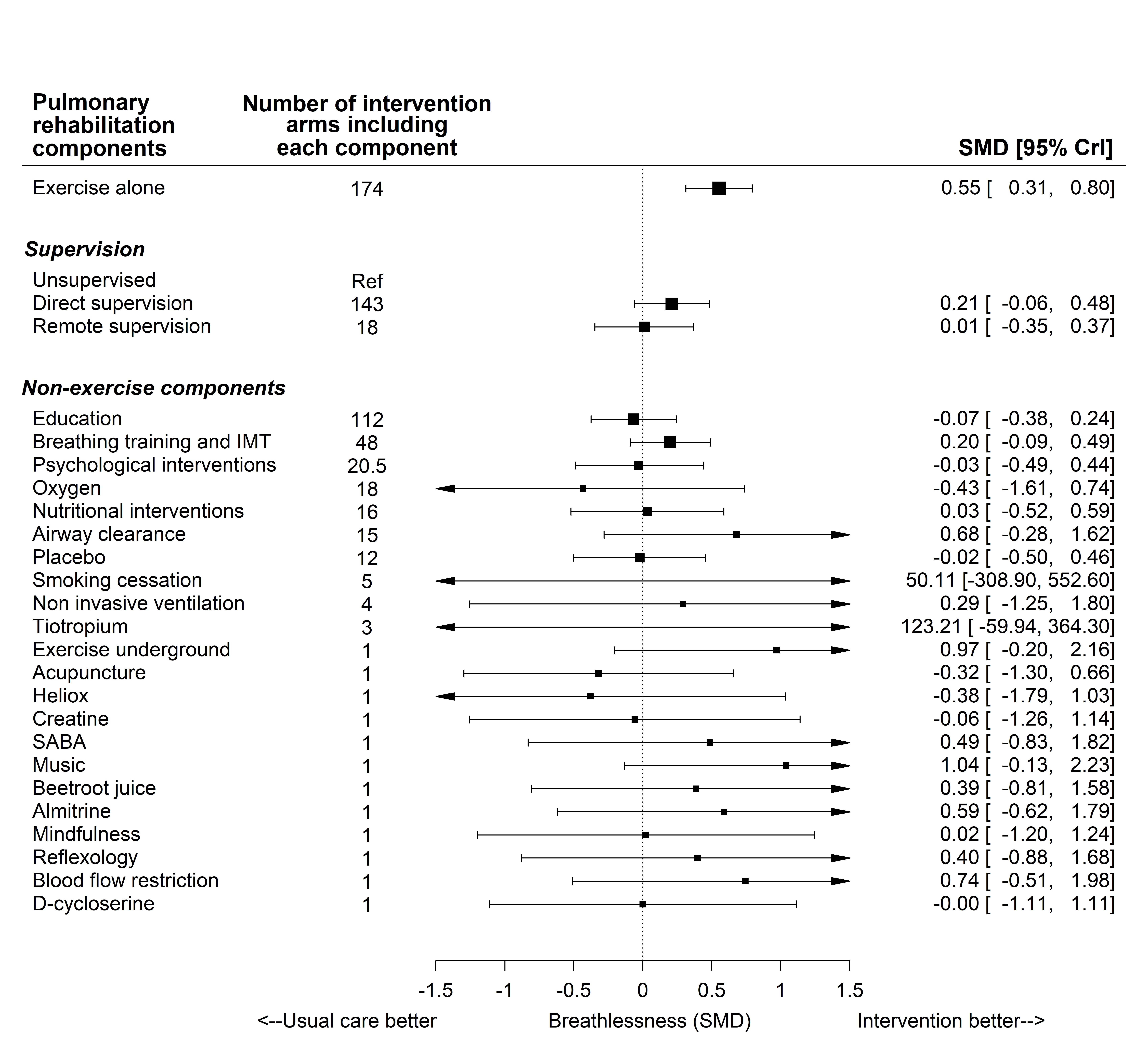
**

**Supplementary figure 35 –** Interim model additive model centred for mean baseline FEV_1_ for outcome of breathlessness

**
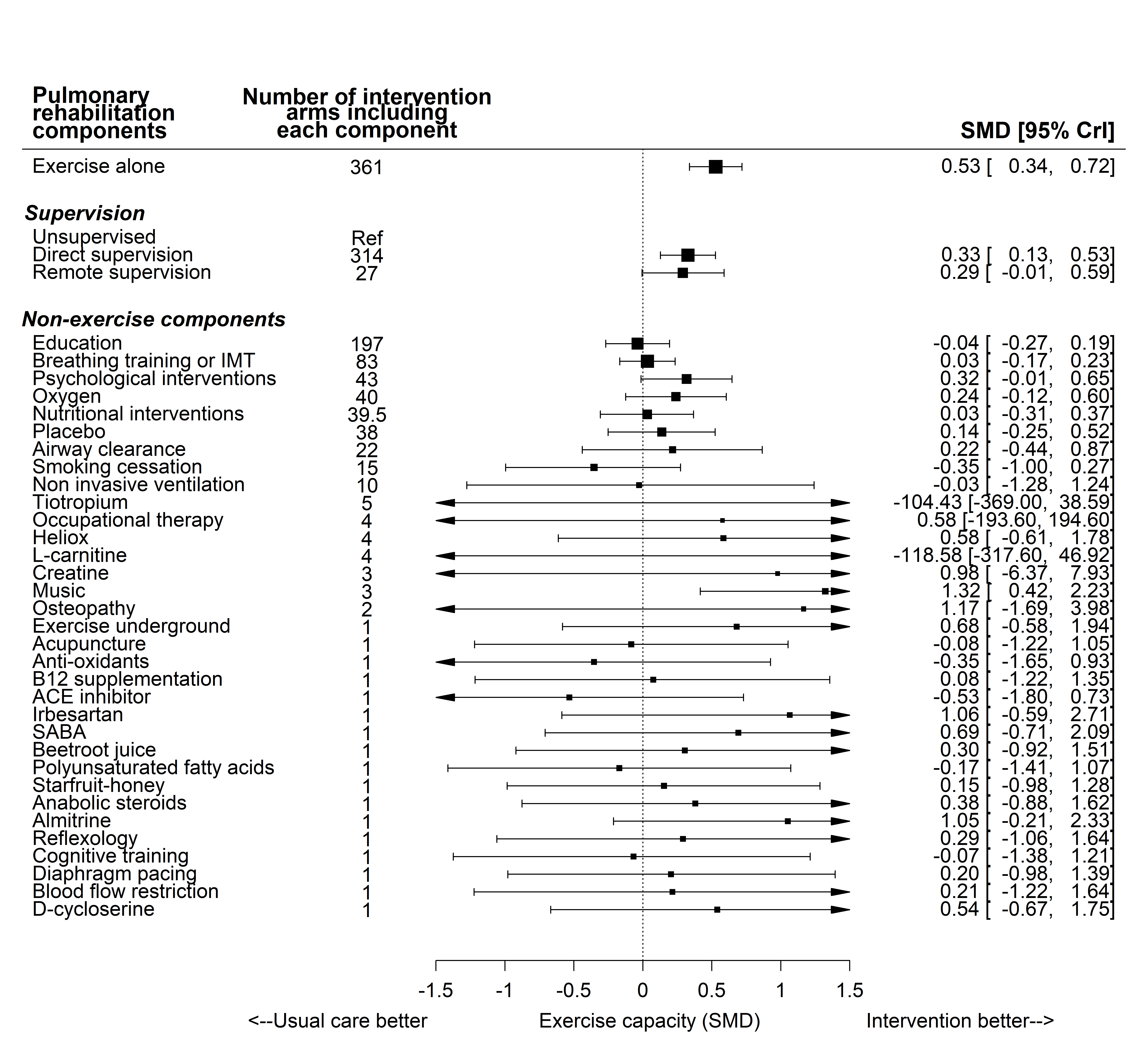
**

**Supplementary figure 36 –** Interim model additive model centred for mean length of programme (weeks) for outcome of exercise capacity

**
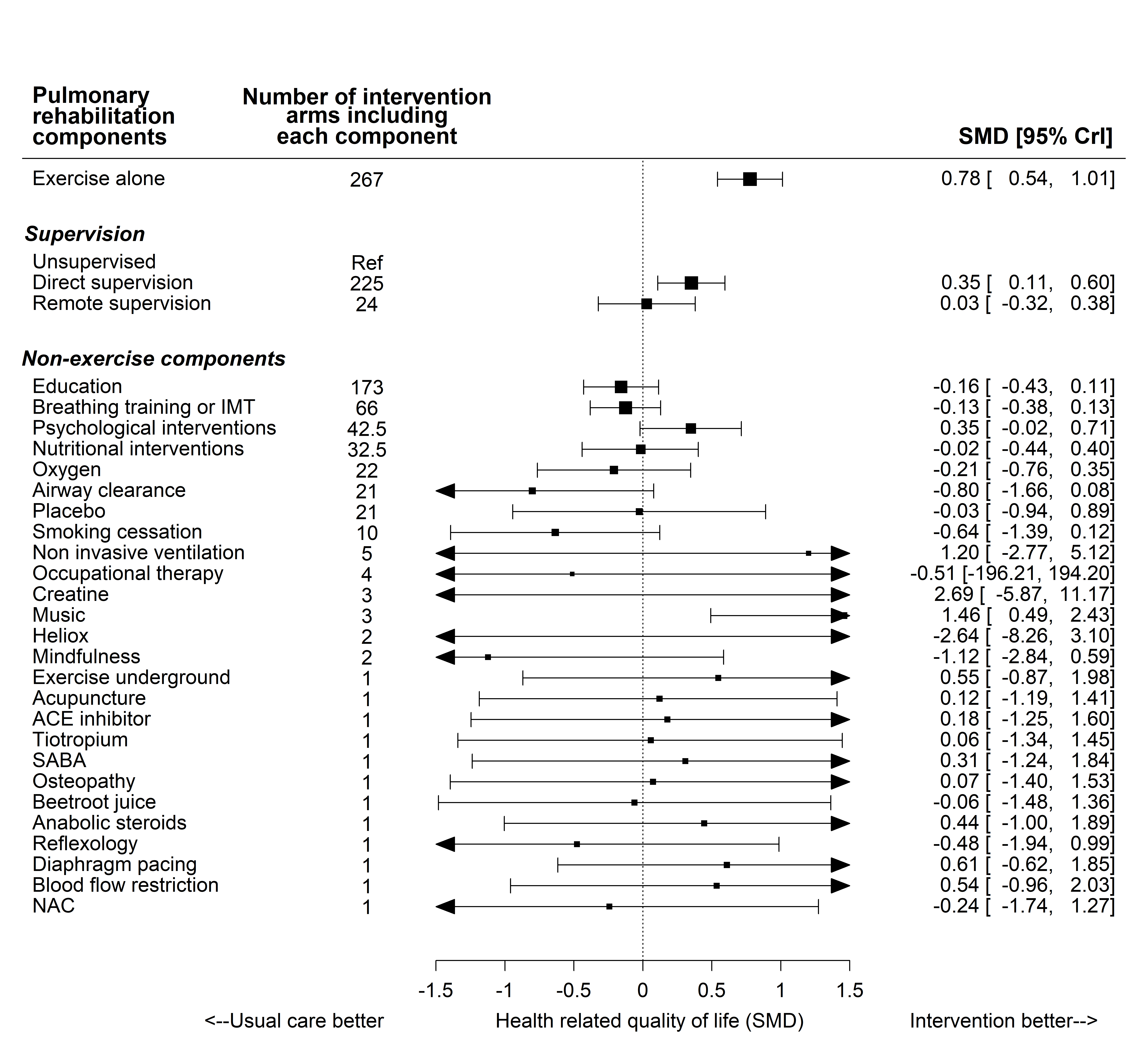
**

**Supplementary figure 37 –** Interim model additive model centred for mean length of programme (weeks) for outcome of quality of life

**
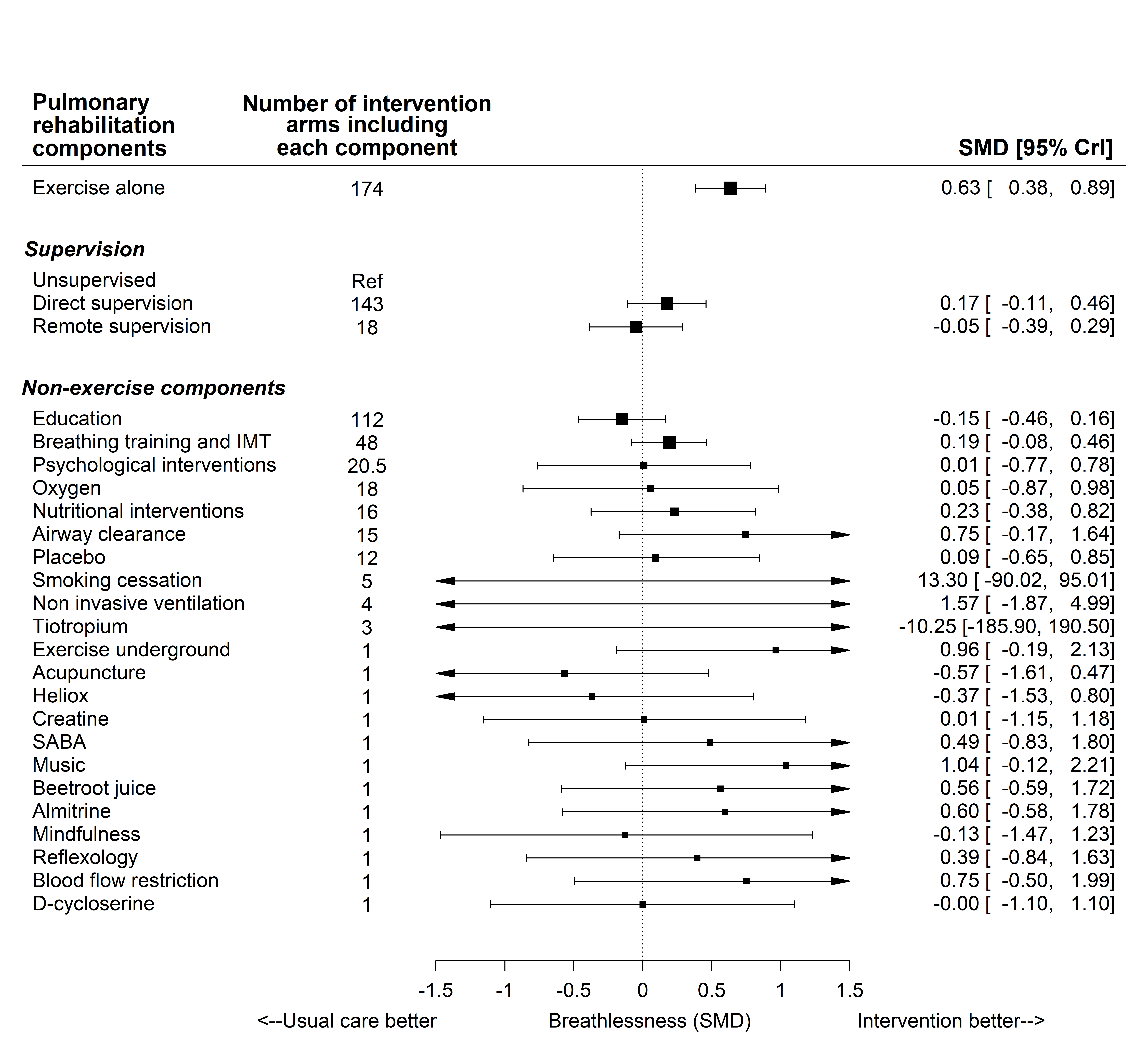
**

**Supplementary figure 38 –** Interim model additive model centred for mean length of programme (weeks) for outcome of breathlessness

**
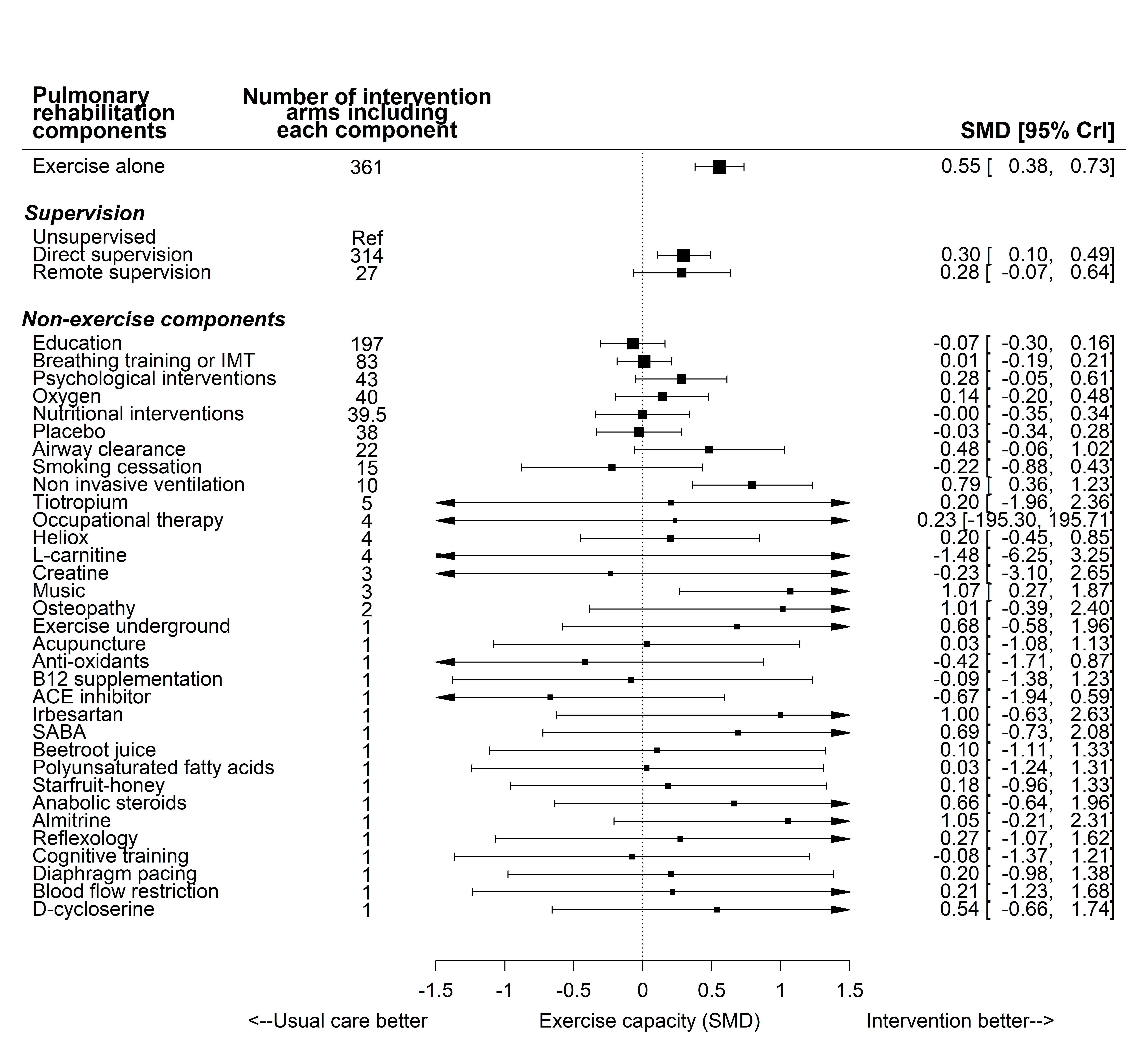
**

**Supplementary figure 39 –** Interim model additive model centred for mean year of publication for outcome of exercise capacity

**
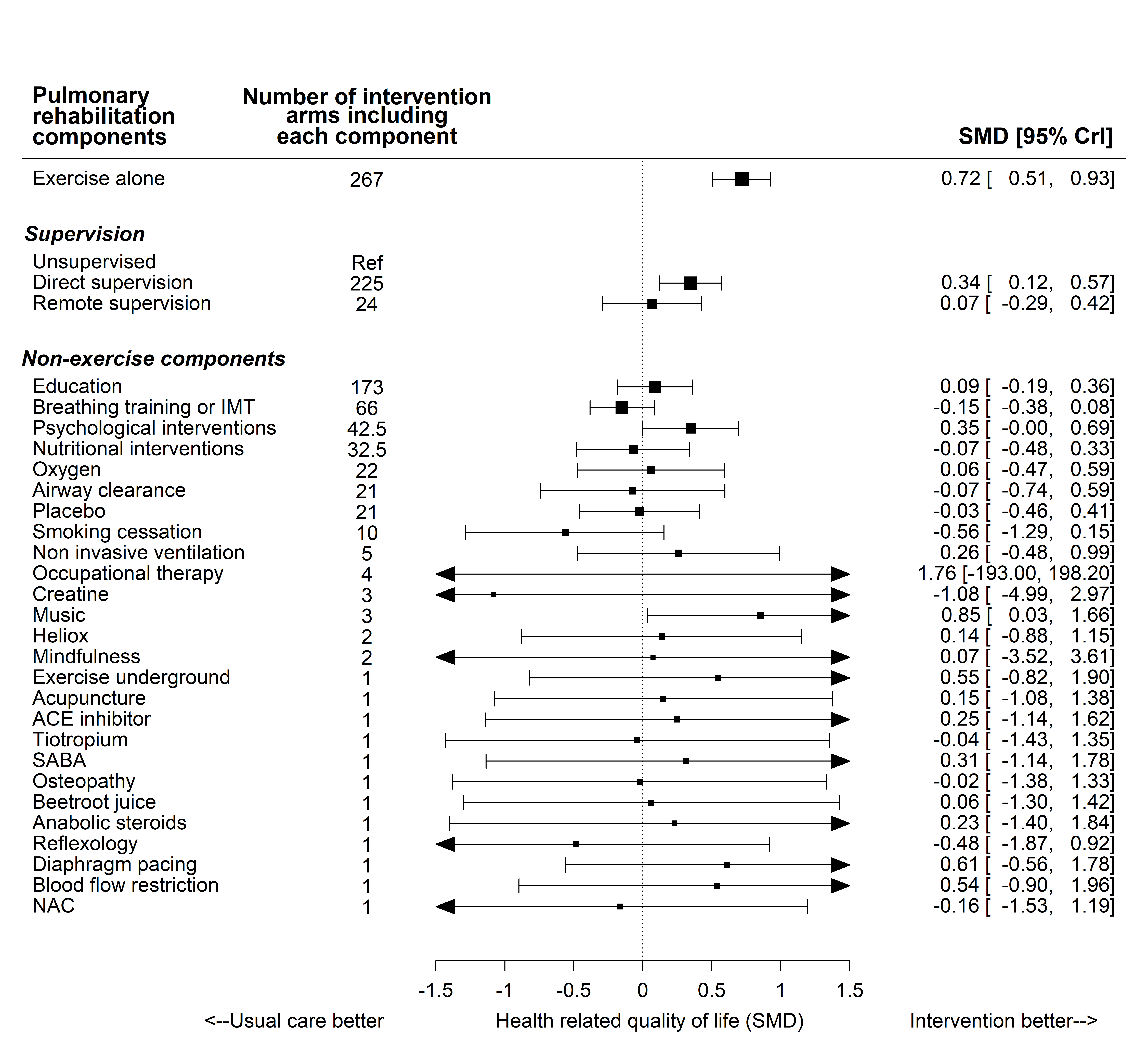
**

**Supplementary figure 40 –** Interim model additive model centred for mean year of publication for outcome of quality of life

**
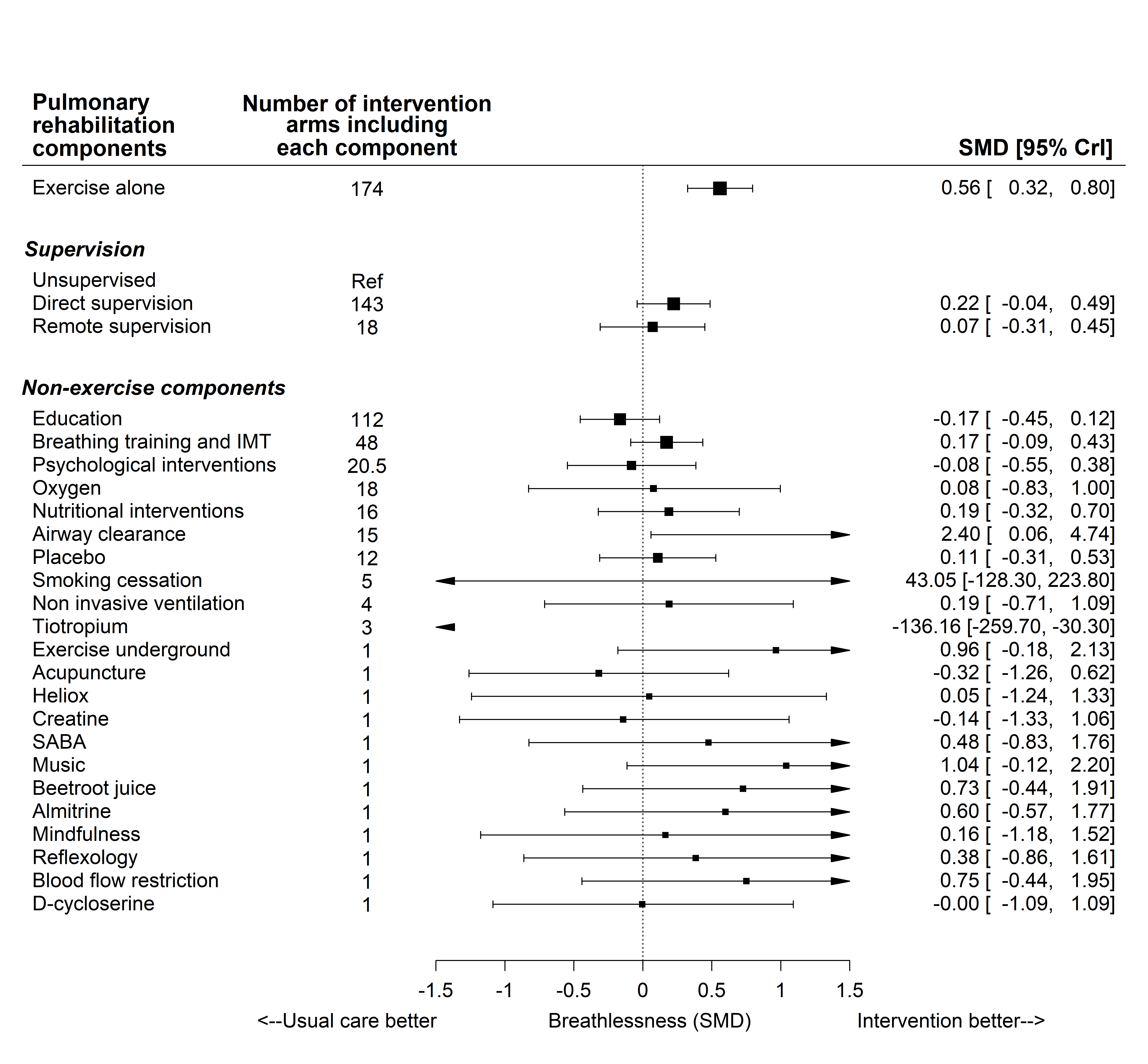
**

**Supplementary figure 41 –** Interim model additive model centred for mean year of publication for outcome of breathlessness

**
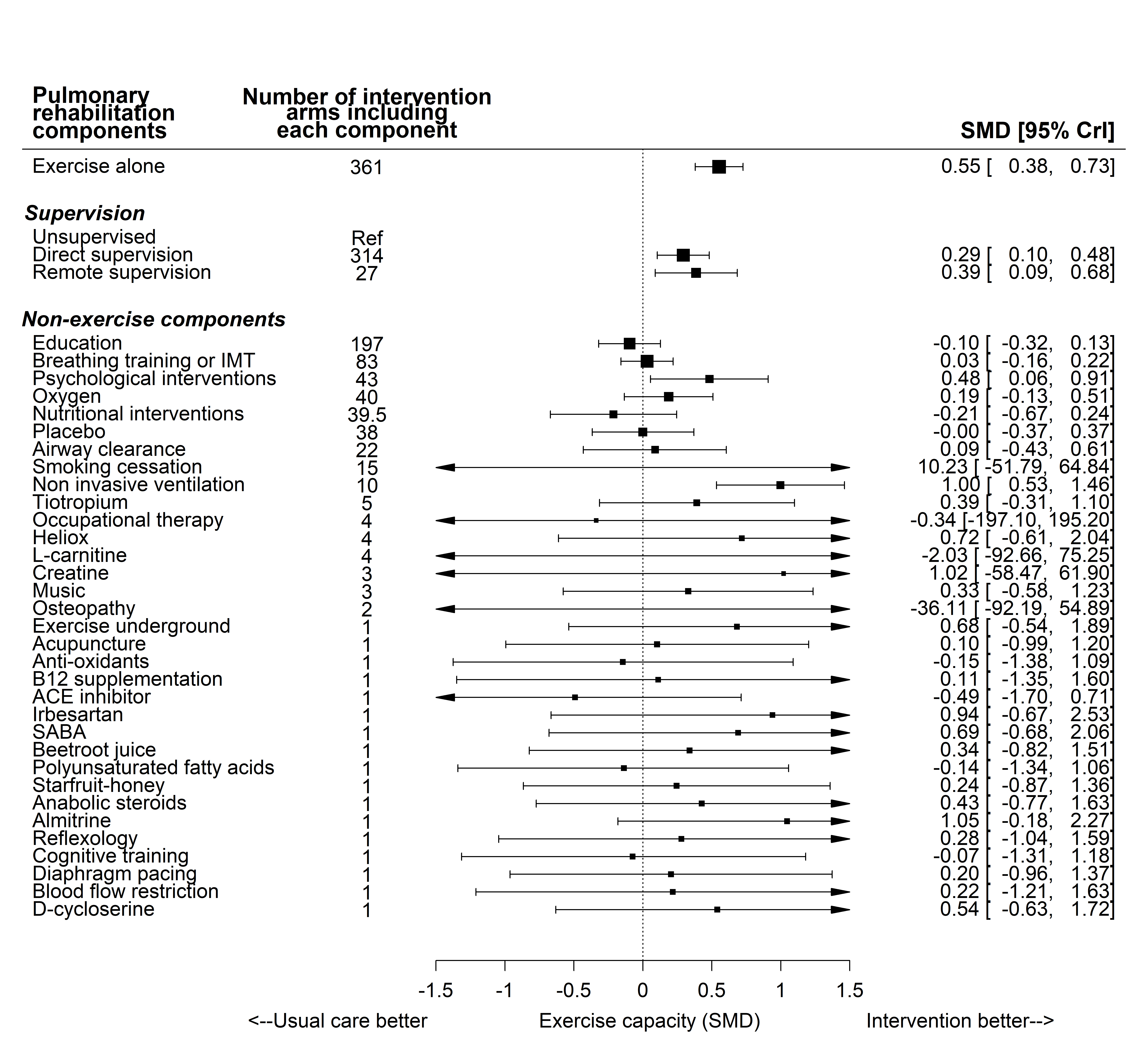
**

**Supplementary figure 42 –** Interim model additive model centred for mean income group of publication country for outcome of exercise capacity

**
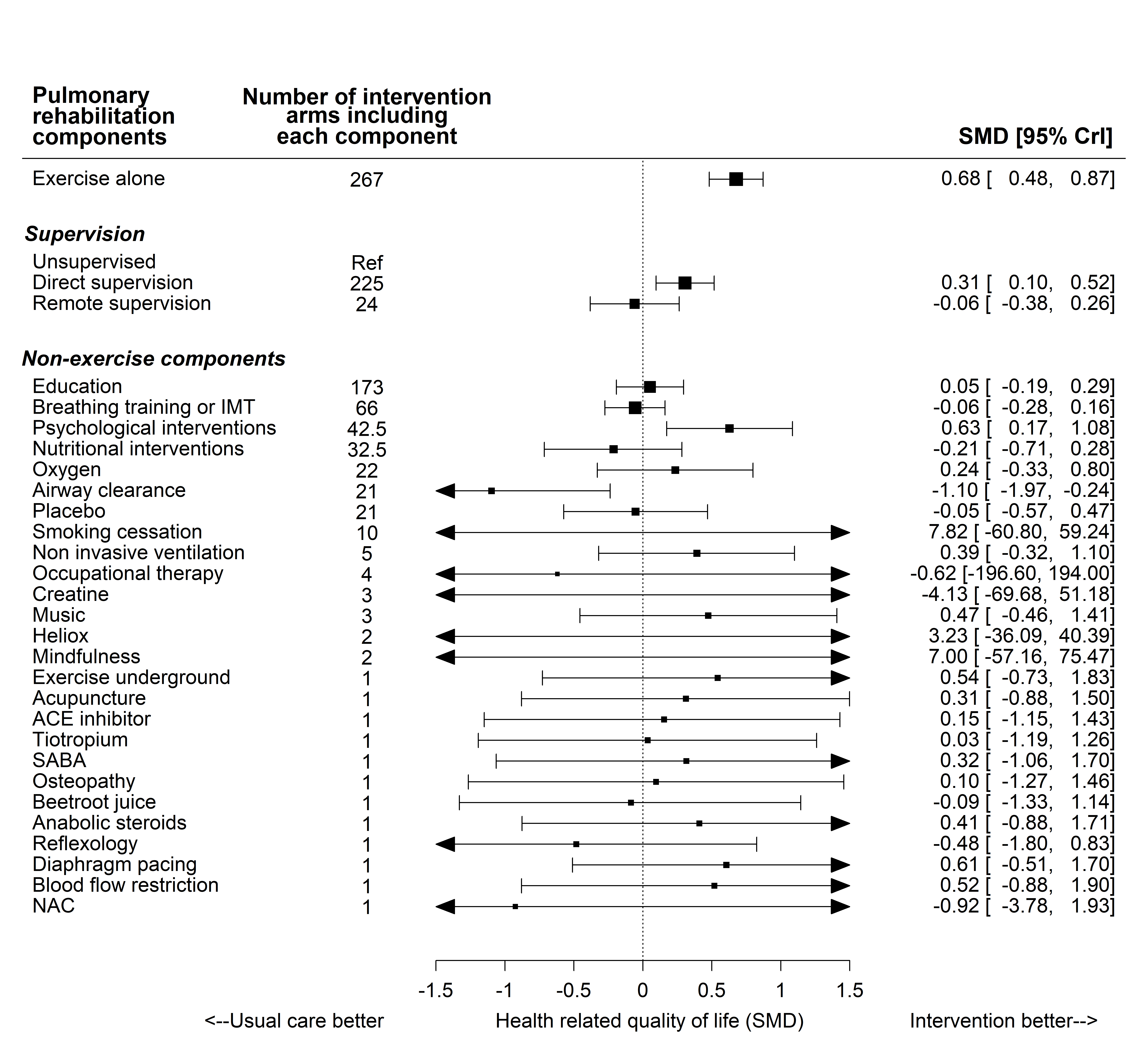
**

**Supplementary figure 43 –** Interim model additive model centred for mean income group of publication country for outcome of quality of life

**
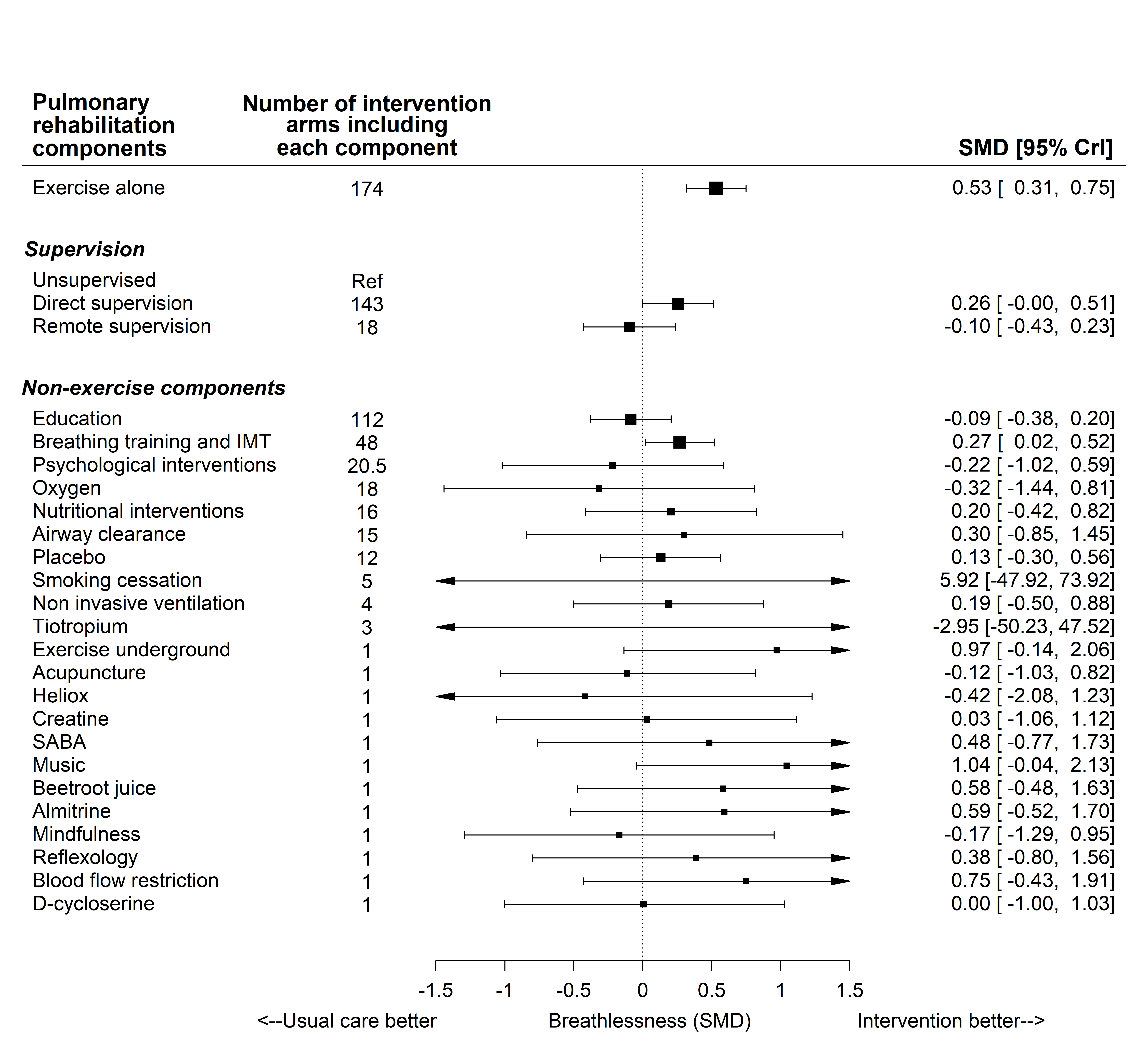
**

**Supplementary figure 44 –** Interim model additive model centred for mean income group of publication country for outcome of breathlessness





**Supplementary figure 45 –** Final model additive model centred for median total number of programme sessions (24) for outcome of exercise capacity

**

**

**Supplementary figure 46 –** Final model additive model centred for median total number of programme sessions (24) for outcome of health-related quality of life

**

**

**Supplementary figure 47 –** Final model additive model centred for median total number of programme sessions (24) for outcome of breathlessness

**

**

**Supplementary figure 48 –** Final model additive model centred for median total programme “dose” (number of programme sessions x duration of sessions) for outcome of exercise capacity (median dose = 24 hours)

**

**

**Supplementary figure 49 –** Final model additive model centred for median total programme “dose” (number of programme sessions x duration of sessions) for outcome of health-related quality of life (median dose = 27 hours)

**

**

**Supplementary figure 50 –** Final model additive model centred for median total programme “dose” (number of programme sessions x duration of sessions) for outcome of breathlessness (median dose = 26.6 hours)

## **Supplementary table 3** – Assessment of model fit after addition of interaction and covariate effects

| Outcome | Model | DIC | Change in DIC | tau | Change in tau | Total residual deviance | Change in total residual deviance |
| --- | --- | --- | --- | --- | --- | --- | --- |
| Exercise capacity | Interim additive model | 3976.83 |  | 3.22 |  | 478.41 |  |
|  | Interim additive model with interactions | 3977.27 | 0.44 | 3.22 | 0.00 | 478.71 | 0.30 |
|  | Interim additive model with FEV1 as covariate | 3981.34 | 4.51 | 3.26 | 0.04 | 478.63 | 0.21 |
|  | Interim additive model with standardised baseline exercise capacity as covariate | 3982.11 | 5.28 | 3.22 | 0.00 | 478.71 | 0.30 |
|  | Interim additive model with age as covariate | 3983.10 | 6.27 | 3.12 | -0.10 | 477.74 | -0.68 |
|  | Interim additive model with sex as covariate | 3980.93 | 4.10 | 3.16 | -0.05 | 477.27 | -1.15 |
|  | Interim additive model with publication year as covariate | 3981.27 | 4.44 | 3.17 | -0.05 | 478.24 | -0.17 |
|  | Interim additive model with programme duration (weeks) as covariate | 3981.07 | 4.24 | 3.15 | -0.07 | 478.12 | -0.29 |
|  | Interim additive model with income of country as covariate | 3977.73 | 0.90 | 3.43 | 0.22 | 478.50 | 0.09 |
|  |  |  |  |  |  |  |  |
| Health related quality of life | Interim additive model | 1640.49 |  | 2.40 |  | 363.67 |  |
|  | Interim additive model with interactions | 1640.41 | -0.08 | 2.43 | 0.02 | 363.41 | -0.26 |
|  | Interim additive model with FEV1 as covariate | 1642.96 | 2.47 | 2.38 | -0.02 | 363.57 | -0.10 |
|  | Interim additive model with standardised baseline exercise capacity as covariate | 1643.07 | 2.58 | 2.48 | 0.08 | 364.63 | 0.96 |
|  | Interim additive model with age as covariate | 1642.97 | 2.48 | 2.42 | 0.01 | 363.44 | -0.24 |
|  | Interim additive model with sex as covariate | 1641.16 | 0.67 | 2.66 | 0.26 | 363.75 | 0.08 |
|  | Interim additive model with publication year as covariate | 1639.48 | -1.01 | 2.69 | 0.28 | 363.00 | -0.68 |
|  | Interim additive model with programme duration (weeks) as covariate | 1642.32 | 1.83 | 2.40 | 0.00 | 363.19 | -0.48 |
|  | Interim additive model with income of country as covariate | 1638.92 | -1.57 | 3.13 | 0.73 | 366.62 | 2.95 |
|  |  |  |  |  |  |  |  |
| Breathlessness | Interim additive model | 280.00 |  | 4.27 |  | 231.57 |  |
|  | Interim additive model with interactions | 279.46 | -0.55 | 4.30 | 0.03 | 231.01 | -0.57 |
|  | Interim additive model with FEV1 as covariate | 283.06 | 3.06 | 3.88 | -0.39 | 230.35 | -1.23 |
|  | Interim additive model with standardised baseline exercise capacity as covariate | 281.61 | 1.60 | 4.27 | 0.01 | 229.76 | -1.82 |
|  | Interim additive model with age as covariate | 281.61 | 1.61 | 4.25 | -0.01 | 230.79 | -0.78 |
|  | Interim additive model with sex as covariate | 283.41 | 3.41 | 4.49 | 0.23 | 232.38 | 0.81 |
|  | Interim additive model with publication year as covariate | 283.95 | 3.95 | 4.25 | -0.02 | 232.43 | 0.86 |
|  | Interim additive model with programme duration (weeks) as covariate | 282.70 | 2.69 | 4.09 | -0.17 | 231.13 | -0.45 |
|  | Interim additive model with income of country as covariate | 281.00 | 1.00 | 4.92 | 0.65 | 232.34 | 0.77 |

DIC: deviance information criterion

## Risk of bias and quality of reporting

**Supplementary figure 51 -** Risk of bias summary for the outcome of exercise capacity.


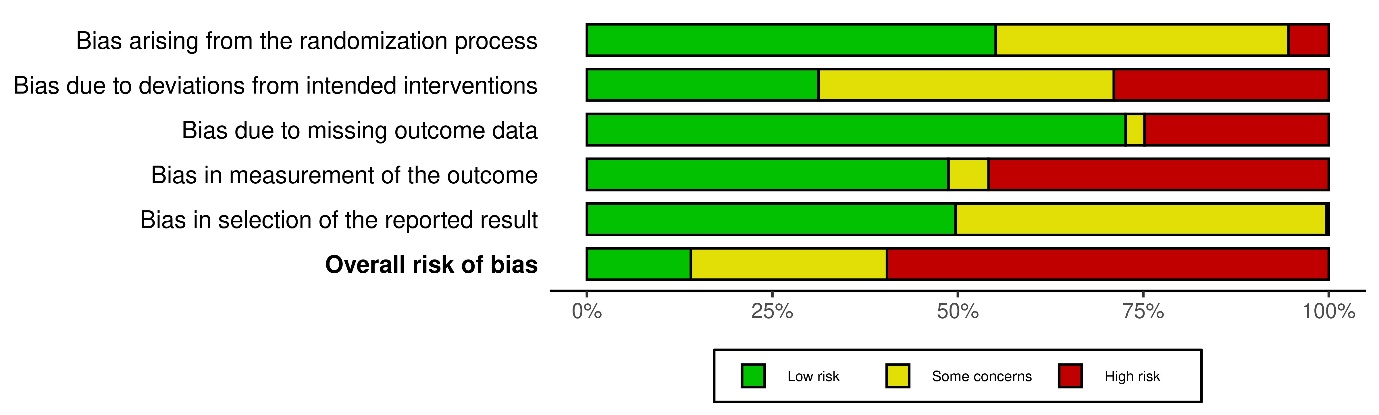


**Supplementary figure 52 -** Risk of bias summary for the outcome of quality of reporting.


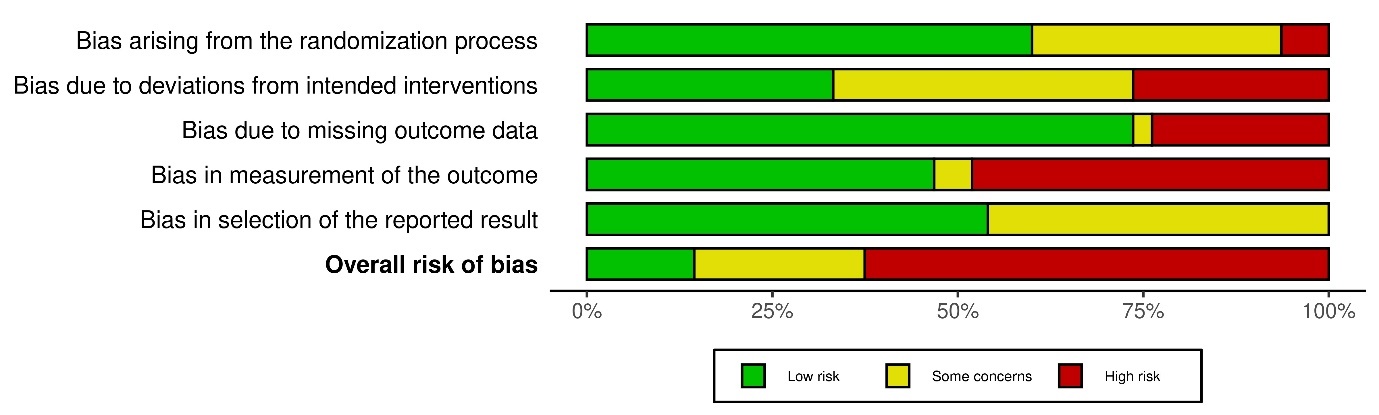


**Supplementary figure 53 -** Risk of bias summary for the outcome of quality of reporting.


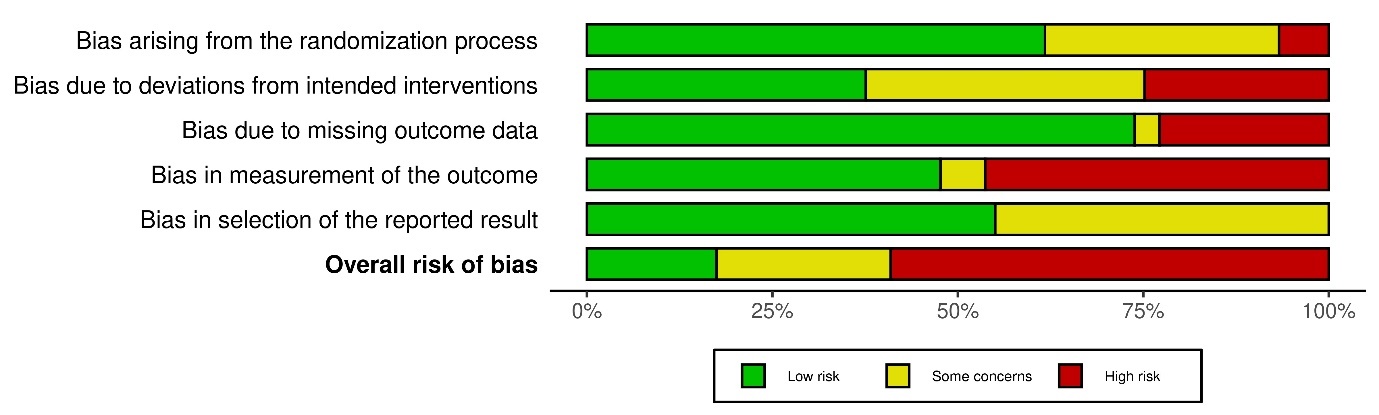


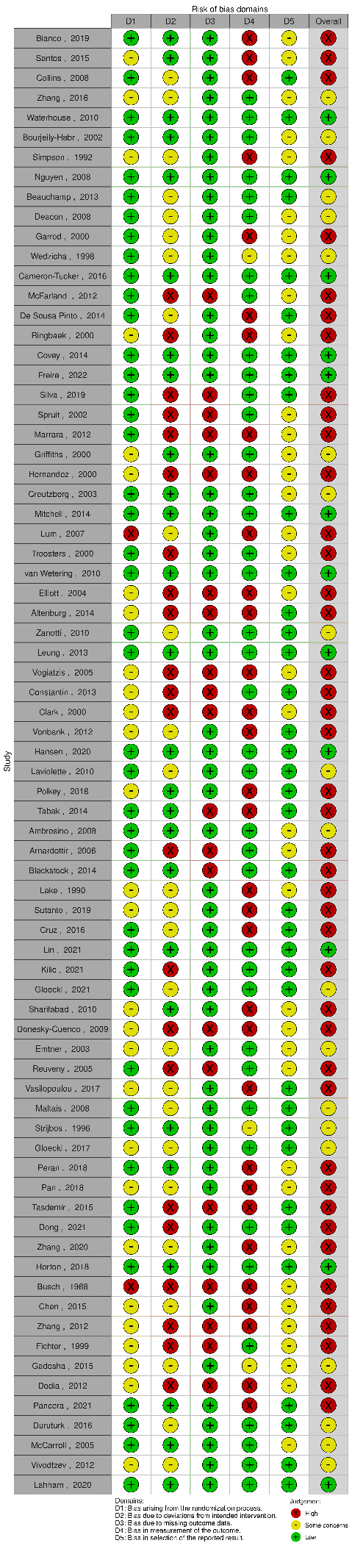

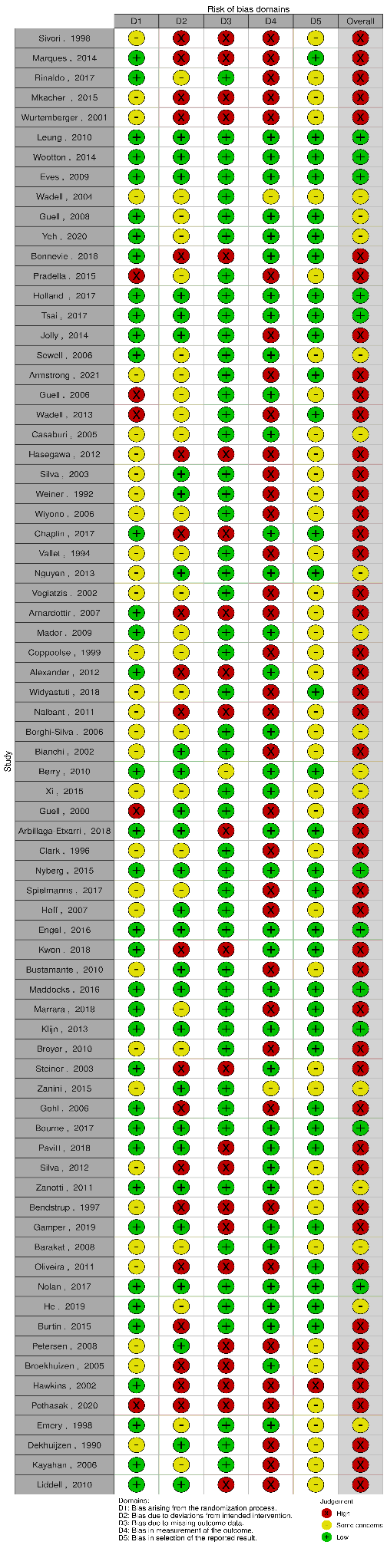
**Supplementary figure 54 -** Risk of bias tables for outcome of exercise capacity




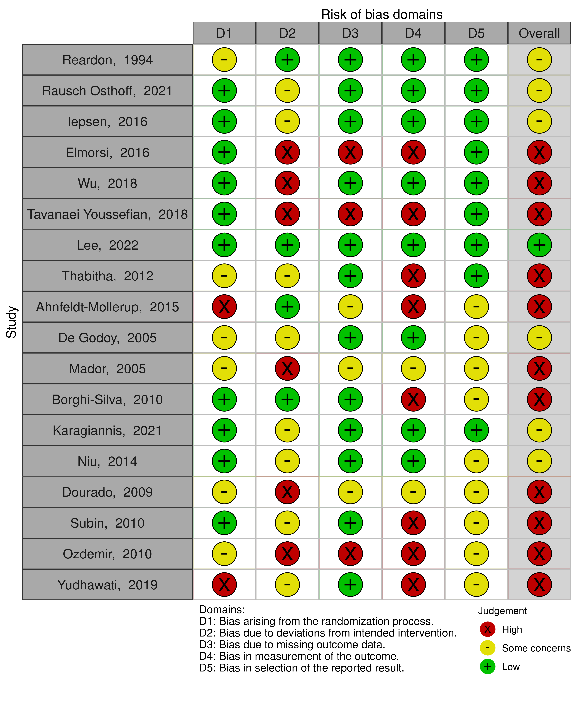

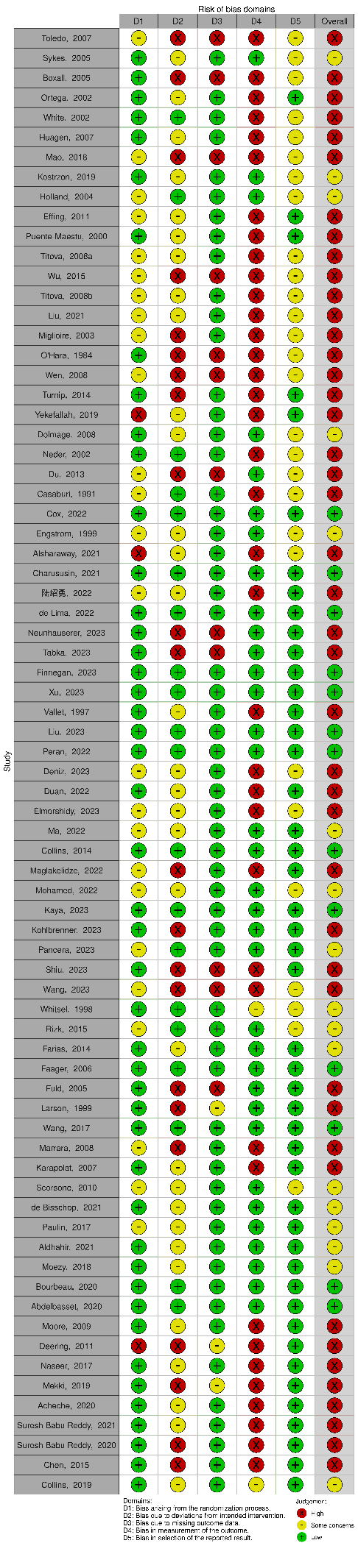


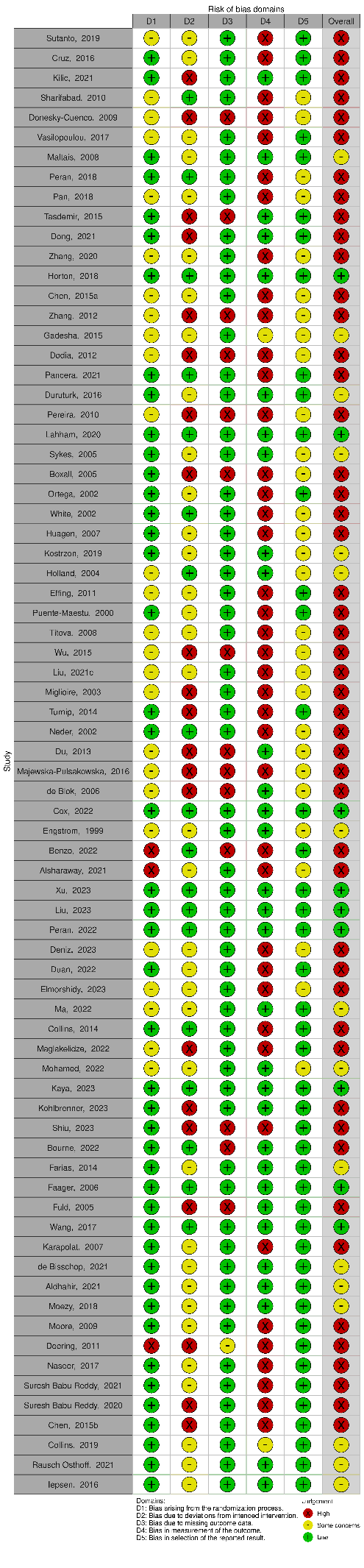

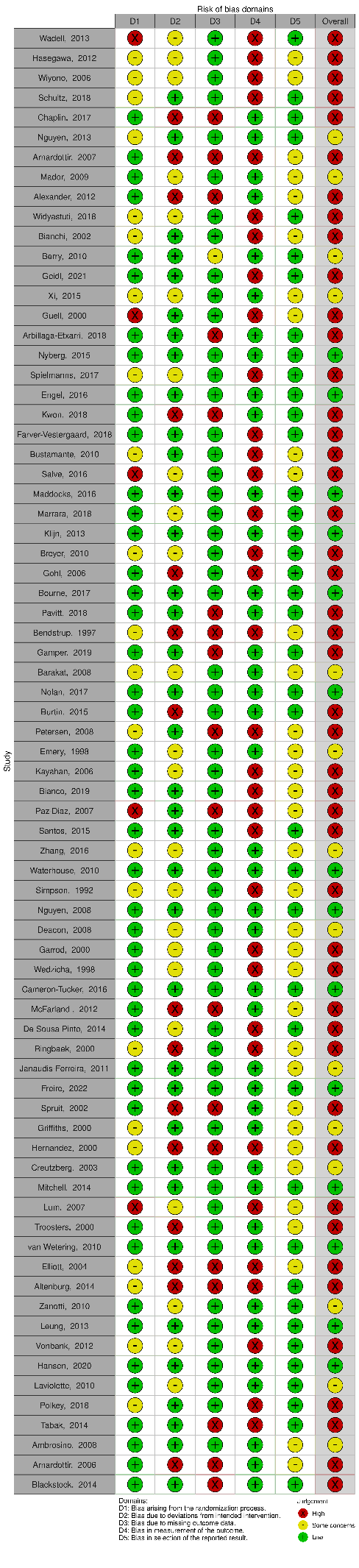

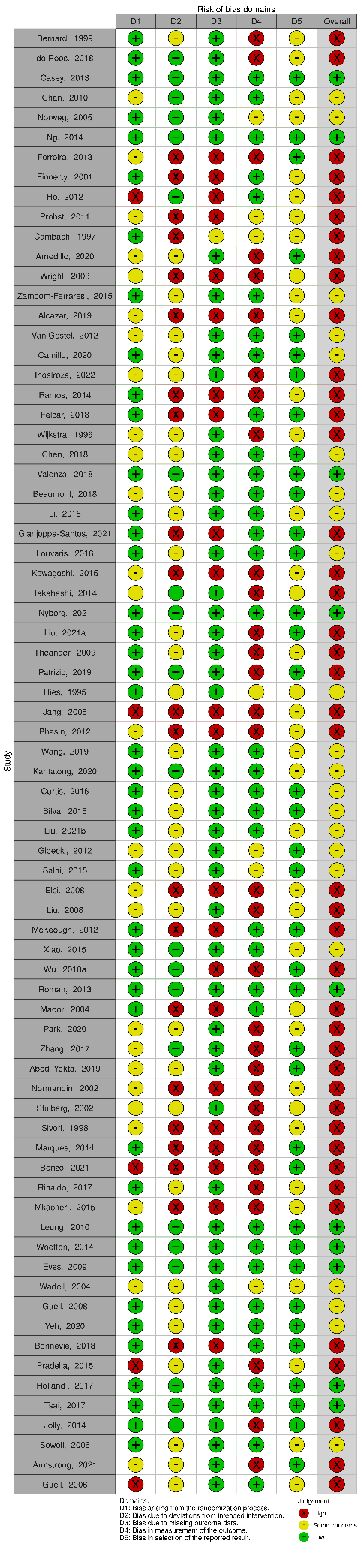
**Supplementary figure 55 -** Risk of bias tables for outcome of health-related quality of life


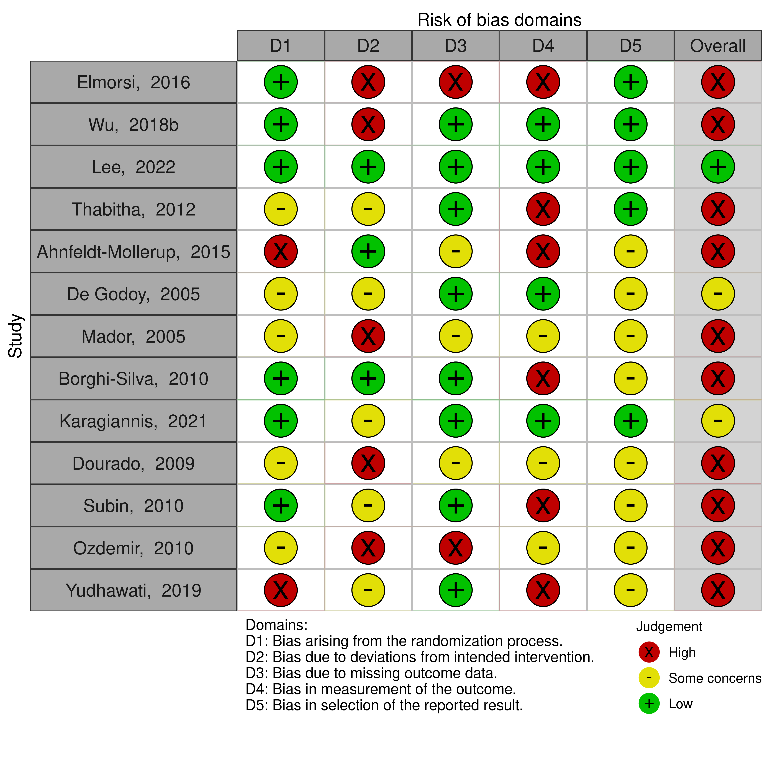


**Supplementary figure 56 -** Risk of bias tables for outcome of breathlessness


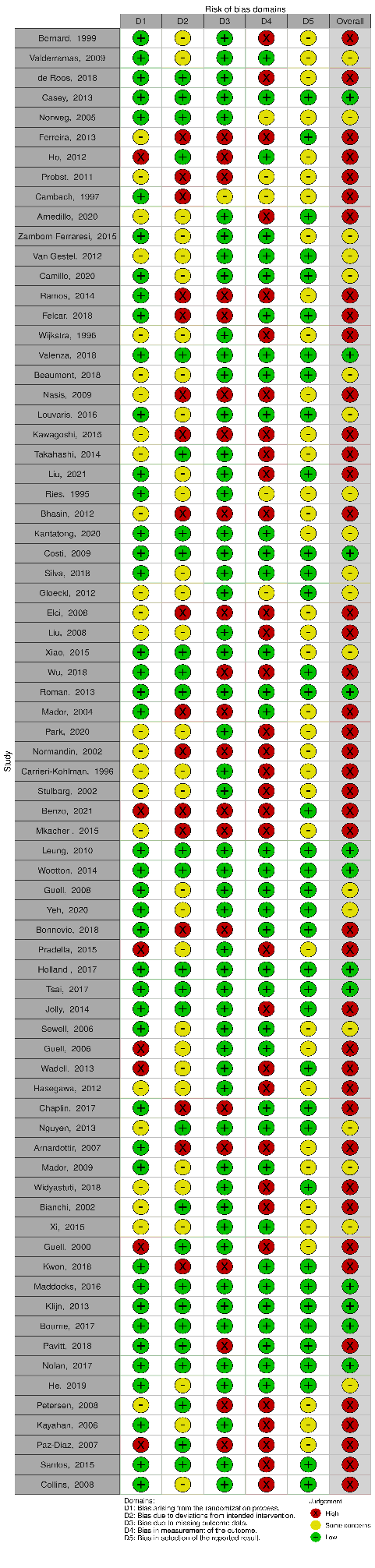

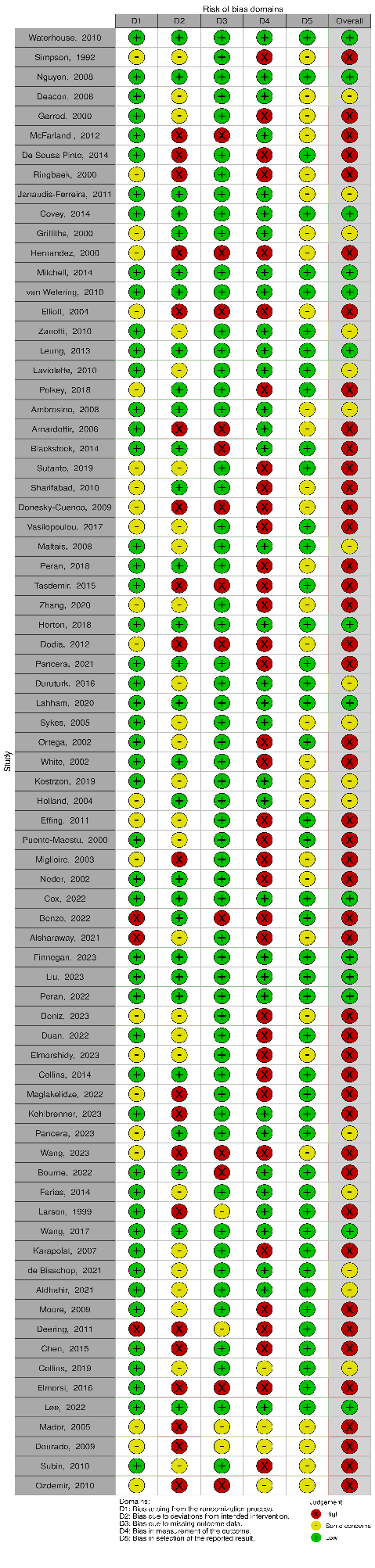


## GRADE assessment of certainty of evidence

| **Supplementary table 4 - Summary of findings:** | | | | |
| --- | --- | --- | --- | --- |
| **Should exercise training be used as part of pulmonary rehabilitation for people with COPD** | | | | |
| **Patient or population: P**ulmonary rehabilitation for COPD  **Setting:** Programmes of a minimum frequency of twice a week for at least 3 weeks  **Intervention:** Exercise training alone (without considering supervision)  **Comparison:** no exercise training | | | | |
| Outcomes | **Anticipated absolute effects^*^** (95% CI) | № of participants (studies) | Certainty of the evidence (GRADE) | Comments |
| Exercise capacity | SMD **0.56 SD higher** (0.38 higher to 0.73 higher) | 14271 (221 RCTs) | ⨁⨁⨁⨁ High^a^ | The exercise training component of pulmonary rehabilitation likely increases exercise capacity. |
| Health related quality of life (QoL) | SMD **0.72 SD higher** (0.50 higher to 0.93 higher) | 13948 (171 RCTs) | ⨁⨁⨁⨁ High | The exercise training component of pulmonary rehabilitation improves health-related quality of life. |
| Breathlessness | SMD **0.54 SD higher** (0.32 higher to 0.76 higher) | 8706 (108 RCTs) | ⨁⨁⨁⨁ High^b^ | The exercise training component of pulmonary rehabilitation may reduce breathlessness slightly. |
| ***The risk in the intervention group** (and its 95% credible interval) is based on the assumed risk in the comparison group and the **relative effect** of the intervention (and its 95% CI).  **CI:** credible interval; **SMD:** standardised mean difference | | | | |
| **GRADE Working Group grades of evidence** **High certainty:** we are very confident that the true effect lies close to that of the estimate of the effect. **Moderate certainty:** we are moderately confident in the effect estimate: the true effect is likely to be close to the estimate of the effect, but there is a possibility that it is substantially different. **Low certainty:** our confidence in the effect estimate is limited: the true effect may be substantially different from the estimate of the effect. **Very low certainty:** we have very little confidence in the effect estimate: the true effect is likely to be substantially different from the estimate of effect. | | | | |

#### Explanations

a. High risk of bias in 129 out of 221 studies. Sensitivity analysis excluding studies at high risk of bias found no change in effect of exercise training on exercise capacity.

b. High risk of bias in 63 out of 108 studies. Sensitivity analysis excluding studies at high risk of bias found no change in effect of exercise training on breathlessness.

| **Supplementary table 5 - Summary of findings:** | | | | |
| --- | --- | --- | --- | --- |
| **Are directly supervised pulmonary rehabilitation programmes better than unsupervised programmes for people with COPD** | | | | |
| **Patient or population:** Pulmonary rehabilitation for COPD  **Setting:** Programmes of a minimum frequency of twice a week for at least 3 weeks  **Intervention:** Direct supervision  **Comparison:** Unsupervised programmes | | | | |
| Outcomes | **Anticipated absolute effects^*^** (95% CI) | № of participants (studies) | Certainty of the evidence (GRADE) | Comments |
| Exercise capacity | SMD **0.41 SD higher** (0.20 higher to 0.63 higher) | 14783 (258 RCTs) | ⨁⨁⨁◯ Moderate^a^ | Direct supervision during pulmonary rehabilitation increases exercise capacity. |
| Health related quality of life (QoL) | SMD **0.43 SD higher** (0.19 higher to 0.68 higher) | 12659 (180 RCTs) | ⨁⨁⨁◯ Moderate^b^ | Direct supervision during pulmonary rehabilitation improves health-related quality of life. |
| Breathlessness | SMD **0.31 SD higher** (0.04 higher to 0.58 higher) | 8259 (119 RCTs) | ⨁⨁⨁⨁ High | Direct supervision during pulmonary rehabilitation reduces breathlessness. |
| ***The risk in the intervention group** (and its 95% credible interval) is based on the assumed risk in the comparison group and the **relative effect** of the intervention (and its 95% CI).  **CI:** credible interval; **SMD:** standardised mean difference | | | | |
| **GRADE Working Group grades of evidence** **High certainty:** we are very confident that the true effect lies close to that of the estimate of the effect. **Moderate certainty:** we are moderately confident in the effect estimate: the true effect is likely to be close to the estimate of the effect, but there is a possibility that it is substantially different. **Low certainty:** our confidence in the effect estimate is limited: the true effect may be substantially different from the estimate of the effect. **Very low certainty:** we have very little confidence in the effect estimate: the true effect is likely to be substantially different from the estimate of effect. | | | | |

#### Explanations

1. High risk of bias in 156 out of 258 studies. Sensitivity analysis excluding studies at high risk of bias found reduced effect
2. High risk of bias in 126 out of 180 studies. Sensitivity analysis excluding studies at high risk of bias found reduced effect
3. Different results in different models

| **Supplementary table 6 - Summary of findings:** | | | | |
| --- | --- | --- | --- | --- |
| **Are remotely supervised pulmonary rehabilitation programmes better than unsupervised programmes for people with COPD** | | | | |
| **Patient or population:** Pulmonary rehabilitation for COPD  **Setting:** Programmes of a minimum frequency of twice a week for at least 3 weeks  **Intervention:**  Remote supervision  **Comparison:** Unsupervised programmes | | | | |
| Outcomes | **Anticipated absolute effects^*^** (95% CI) | № of participants (studies) | Certainty of the evidence (GRADE) | Comments |
| Exercise capacity | SMD **0.4 SD higher** (0.08 higher to 0.73 higher) | 1989 (25 RCTs) | ⨁⨁◯◯ Low ^a,d^ | Remote supervision during pulmonary rehabilitation may increase exercise capacity. |
| Health related quality of life | SMD **0.25 SD higher** (0.10 lower to 0.61 higher) | 2284 (23 RCTs) | ⨁⨁◯◯ Low^b,c,d^ | Remote supervision during pulmonary rehabilitation may improve health-related quality of life. |
| Breathlessness | SMD **0.14 SD higher** (0.18 lower to 0.46 higher) | 1810 (17 RCTs) | ⨁⨁◯◯ Low ^c,d,e^ | Remote supervision during pulmonary rehabilitation may improve breathlessness slightly. |
| ***The risk in the intervention group** (and its 95% credible interval) is based on the assumed risk in the comparison group and the **relative effect** of the intervention (and its 95% CI).  **CI:** credible interval; **SMD:** standardised mean difference | | | | |
| **GRADE Working Group grades of evidence** **High certainty:** we are very confident that the true effect lies close to that of the estimate of the effect. **Moderate certainty:** we are moderately confident in the effect estimate: the true effect is likely to be close to the estimate of the effect, but there is a possibility that it is substantially different. **Low certainty:** our confidence in the effect estimate is limited: the true effect may be substantially different from the estimate of the effect. **Very low certainty:** we have very little confidence in the effect estimate: the true effect is likely to be substantially different from the estimate of effect. | | | | |

#### Explanations

a. High risk of bias in 11 out of 25 studies with reduced effect estimate in a sensitivity analysis excluding studies at high risk of bias.

b. High risk of bias in 12 out of 25 studies, however effect estimate did not change in a sensitivity analysis excluding studies at high risk of bias

c. Different results in different models

d. Imprecise estimate with wide credible intervals

e. High risk of bias in 16 out of 25 studies, however effect estimate did not change in a sensitivity analysis excluding studies at high risk of bias

| **Supplementary table 7 - Summary of findings:** | | | | |
| --- | --- | --- | --- | --- |
| **Should structured education be used alongside exercise training as part of pulmonary rehabilitation for people with COPD** | | | | |
| **Patient or population: P**ulmonary rehabilitation for COPD  **Setting:** Programmes of a minimum frequency of twice a week for at least 3 weeks  **Intervention:** Structured education  **Comparison:** no structured education | | | | |
| Outcomes | **Anticipated absolute effects^*^** (95% CI) | № of participants (studies) | Certainty of the evidence (GRADE) | Comments |
| Exercise capacity | SMD **0.21 SD lower** (0.48 lower to 0.06 higher) | 11026 (147 RCTs) | ⨁⨁⨁◯ Moderate^a^ | Addition of education to pulmonary rehabilitation may reduce improvements in exercise capacity. |
| Health related quality of life (QoL) | SMD **0.08 SD higher** (0.22 lower to 0.37 higher) | 11043 (121 RCTs) | ⨁⨁◯◯ Low^b, c^ | The education component of pulmonary rehabilitation may not impact health-related quality of life. |
| Breathlessness | SMD **0.07 SD higher** (0.37 lower to 0.23 higher) | 7072 (81 RCTs) | ⨁⨁◯◯ Low ^c,d^ | The education component of pulmonary rehabilitation may not impact breathlessness. |
| ***The risk in the intervention group** (and its 95% credible interval) is based on the assumed risk in the comparison group and the **relative effect** of the intervention (and its 95% CI).  **CI:** credible interval; **SMD:** standardised mean difference | | | | |
| **GRADE Working Group grades of evidence** **High certainty:** we are very confident that the true effect lies close to that of the estimate of the effect. **Moderate certainty:** we are moderately confident in the effect estimate: the true effect is likely to be close to the estimate of the effect, but there is a possibility that it is substantially different. **Low certainty:** our confidence in the effect estimate is limited: the true effect may be substantially different from the estimate of the effect. **Very low certainty:** we have very little confidence in the effect estimate: the true effect is likely to be substantially different from the estimate of effect. | | | | |

#### Explanations

a. High risk of bias in 79 out of 147 studies. Increase in effect once studies at high risk of bias removed.

b. High risk of bias in 63 out of 115 studies. Increase in effect once studies at high risk of bias removed.

c. Significant variation in effect across different models reducing confidence in precision.

d. High risk of bias in 44 out of 81 studies. Increase in effect once studies at high risk of bias removed.

| **Supplementary table 8 - Summary of findings:** | | | | |
| --- | --- | --- | --- | --- |
| **Should breathing exercises be used alongside exercise training as part of pulmonary rehabilitation for people with COPD** | | | | |
| **Patient or population: P**ulmonary rehabilitation for COPD  **Setting:** Programmes of a minimum frequency of twice a week for at least 3 weeks  **Intervention:** Breathing exercises  **Comparison:** no breathing exercises | | | | |
| Outcomes | **Anticipated absolute effects^*^** (95% CI) | № of participants (studies) | Certainty of the evidence (GRADE) | Comments |
| Exercise capacity | SMD **0.10 SD lower** (0.35 lower to 0.14 higher) | 4428 (72 RCTs) | ⨁⨁⨁◯ Moderate^a^ | Breathing retraining as a component of pulmonary rehabilitation likely results in little to no difference in exercise capacity. |
| Health related quality of life | SMD **0.01 SD lower** (0.29 lower to 0.28 higher) | 2698 (55 RCTs) | ⨁⨁⨁⨁ High | Breathing retraining as a component of pulmonary rehabilitation may result in little to no difference in health-related quality of life. |
| Breathlessness | SMD **0.26 SD higher** (0.04 lower to 0.56 higher) | 2720 (39 RCTs) | ⨁⨁◯◯ Low^b^ | Breathing retraining as a component of pulmonary rehabilitation may reduce breathlessness slightly. |
| ***The risk in the intervention group** (and its 95% credible interval) is based on the assumed risk in the comparison group and the **relative effect** of the intervention (and its 95% CI).  **CI:** credible interval; **SMD:** standardised mean difference | | | | |
| **GRADE Working Group grades of evidence** **High certainty:** we are very confident that the true effect lies close to that of the estimate of the effect. **Moderate certainty:** we are moderately confident in the effect estimate: the true effect is likely to be close to the estimate of the effect, but there is a possibility that it is substantially different. **Low certainty:** our confidence in the effect estimate is limited: the true effect may be substantially different from the estimate of the effect. **Very low certainty:** we have very little confidence in the effect estimate: the true effect is likely to be substantially different from the estimate of effect. | | | | |

#### Explanations

1. High risk of bias in 44 out of 72 studies and significant change in result when studies at high risk of bias excluded.
2. High risk of bias in 24 out of 39 studies with no effect seen when studies at high risk of bias excluded.

| **Supplementary table 9 - Summary of findings:** | | | | |
| --- | --- | --- | --- | --- |
| **Should inspiratory muscle training be used alongside exercise training as part of pulmonary rehabilitation for people with COPD** | | | | |
| **Patient or population: P**ulmonary rehabilitation for COPD  **Setting:** Programmes of a minimum frequency of twice a week for at least 3 weeks  **Intervention:** Inspiratory muscle training  **Comparison:** no inspiratory muscle training | | | | |
| Outcomes | **Anticipated absolute effects^*^** (95% CI) | № of participants (studies) | Certainty of the evidence (GRADE) | Comments |
| Exercise capacity | SMD **0.59 SD higher** (0.21 higher to 0.97 higher) | 1001 (22 RCTs) | ⨁⨁⨁◯ Moderate^a,b^ | Inspiratory muscle training as a component of pulmonary rehabilitation may improve exercise capacity. |
| Health related quality of life | SMD **0.33 SD higher** (0.14 lower to 0.79 higher) | 1337 (15 RCTs) | ⨁⨁⨁◯ Moderate^b^ | Inspiratory muscle training as a component of pulmonary rehabilitation may improve health-related quality of life. |
| Breathlessness | SMD **0.13 SD higher** (0.29 lower to 0.55 higher) | 675 (12 RCTs) | ⨁⨁◯◯ Low^b,c^ | Inspiratory muscle as a component of pulmonary rehabilitation may result in little to no additional improvement in breathlessness. |
| ***The risk in the intervention group** (and its 95% credible interval) is based on the assumed risk in the comparison group and the **relative effect** of the intervention (and its 95% CI).  **CI:** credible interval; **SMD:** standardised mean difference | | | | |
| **GRADE Working Group grades of evidence** **High certainty:** we are very confident that the true effect lies close to that of the estimate of the effect. **Moderate certainty:** we are moderately confident in the effect estimate: the true effect is likely to be close to the estimate of the effect, but there is a possibility that it is substantially different. **Low certainty:** our confidence in the effect estimate is limited: the true effect may be substantially different from the estimate of the effect. **Very low certainty:** we have very little confidence in the effect estimate: the true effect is likely to be substantially different from the estimate of effect. | | | | |

#### Explanations

a. High risk of bias in 13 out of 22 studies with no significant change in estimate after studies at high risk of bias removed.

b. Imprecise estimate with wide credible intervals

c. High risk of bias in 7 out of 12 studies without significant change in estimate after studies at high risk of bias removed.

| **Supplementary table 10 - Summary of findings:** | | | | |
| --- | --- | --- | --- | --- |
| **Should nutritional interventions be used alongside exercise training as part of pulmonary rehabilitation for people with COPD** | | | | |
| **Patient or population: P**ulmonary rehabilitation for COPD  **Setting:** Programmes of a minimum frequency of twice a week for at least 3 weeks  **Intervention:** Nutritional interventions  **Comparison:** no nutritional interventions | | | | |
| Outcomes | **Anticipated absolute effects^*^** (95% CI) | № of participants (studies) | Certainty of the evidence (GRADE) | Comments |
| Exercise capacity | SMD **0.03 SD lower** (0.41 lower to 0.35 higher) | 2894 (37 RCTs) | ⨁⨁⨁◯ Moderate^a,b^ | Nutritional interventions as a component of pulmonary rehabilitation may result in little to no additional difference in exercise capacity. |
| Health related quality of life | SMD **0.19 SD higher** (0.25 lower to 0.64 higher) | 2320 (24 RCTs) | ⨁⨁◯◯ Low^b^ | Nutritional interventions as a component of pulmonary rehabilitation probably results in little to no additional difference in health-related quality of life. |
| Breathlessness | SMD **0.27 SD higher** (0.25 lower to 0.78 higher) | 1528 (16 RCTs) | ⨁◯◯◯ Very low^b,c^ | The results are very uncertain about the effect of nutritional interventions as a component of pulmonary rehabilitation on breathlessness. |
| ***The risk in the intervention group** (and its 95% credible interval) is based on the assumed risk in the comparison group and the **relative effect** of the intervention (and its 95% CI).  **CI:** credible interval; **SMD:** standardised mean difference | | | | |
| **GRADE Working Group grades of evidence** **High certainty:** we are very confident that the true effect lies close to that of the estimate of the effect. **Moderate certainty:** we are moderately confident in the effect estimate: the true effect is likely to be close to the estimate of the effect, but there is a possibility that it is substantially different. **Low certainty:** our confidence in the effect estimate is limited: the true effect may be substantially different from the estimate of the effect. **Very low certainty:** we have very little confidence in the effect estimate: the true effect is likely to be substantially different from the estimate of effect. | | | | |

#### Explanations

a. High risk of bias in 19 out of 37 studies. Sensitivity analysis excluding studies at high risk of bias found similar effect.

b. Imprecise estimate with very wide credible intervals

c. High risk of bias in 7 out of 16 studies. Sensitivity analysis excluding studies at high risk of bias found reduced effect.

| **Supplementary table 11 - Summary of findings:** | | | | |
| --- | --- | --- | --- | --- |
| **Should psychological interventions be used alongside exercise training as part of pulmonary rehabilitation for people with COPD** | | | | |
| **Patient or population:** Pulmonary rehabilitation for COPD  **Setting:** Programmes of a minimum frequency of twice a week for at least 3 weeks  **Intervention:** Psychological interventions  **Comparison:** no psychological interventions | | | | |
| Outcomes | **Anticipated absolute effects^*^** (95% CI) | № of participants (studies) | Certainty of the evidence (GRADE) | Comments |
| Exercise capacity | SMD **0.37 SD higher** (0.01 higher to 0.73 higher) | 2693 (39 RCTs) | ⨁⨁◯◯ Low^a,b^ | Psychological interventions as a component of pulmonary rehabilitation may result in improvements in exercise capacity. |
| Health related quality of life | SMD **0.54 SD higher** (0.18 higher to 0.91 higher) | 3009 (34 RCTs) | ⨁⨁⨁◯ Moderate^b,c^ | Psychological interventions as a component of pulmonary rehabilitation may improve health related quality of life. |
| Breathlessness | SMD **0.02 SD higher** (0.42 lower to 0.39 higher) | 1737 (18 RCTs) | ⨁⨁◯◯ Low^b,e^ | Psychological interventions as a component of pulmonary rehabilitation may result in little to no additional difference in breathlessness. |
| ***The risk in the intervention group** (and its 95% credible interval) is based on the assumed risk in the comparison group and the **relative effect** of the intervention (and its 95% CI).  **CI:** credible interval; **SMD:** standardised mean difference | | | | |
| **GRADE Working Group grades of evidence** **High certainty:** we are very confident that the true effect lies close to that of the estimate of the effect. **Moderate certainty:** we are moderately confident in the effect estimate: the true effect is likely to be close to the estimate of the effect, but there is a possibility that it is substantially different. **Low certainty:** our confidence in the effect estimate is limited: the true effect may be substantially different from the estimate of the effect. **Very low certainty:** we have very little confidence in the effect estimate: the true effect is likely to be substantially different from the estimate of effect. | | | | |

#### Explanations

a. High risk of bias in 24 out of 39 studies. Sensitivity analysis excluding studies at high risk of bias found an reduced effect on exercise capacity.

b. Imprecise estimate with wide credible intervals

c. High risk of bias in 23 out of 35 studies. Sensitivity analysis excluding studies at high risk of bias found no change in effect.

d. High risk of bias in 10 out of 18 studies. Sensitivity analysis excluding studies at high risk of bias found an increased effect.

| **Supplementary table 12 - Summary of findings:** | | | | |
| --- | --- | --- | --- | --- |
| **Lower limb strength training compared to no strength training in pulmonary rehabilitation for people with COPD** | | | | |
| **Patient or population:** Pulmonary rehabilitation for COPD  **Setting:** Programmes of a minimum frequency of twice a week for at least 3 weeks  **Intervention:** Lower limb strength training  **Comparison:** no strength training | | | | |
| Outcomes | **Anticipated absolute effects^*^** (95% CI) | № of participants (studies) | Certainty of the evidence (GRADE) | Comments |
| Exercise capacity | SMD **0.20 SD higher** (0.10 lower to 0.51 higher) | 5682 (98 RCTs) | ⨁⨁⨁◯ Moderate^a^ | Addition of lower limb strength training as a component of pulmonary rehabilitation may result in small improvements in exercise capacity. |
| Health related quality of life | SMD **0.04 SD higher** (0.33 lower to 0.40 higher) | 5048 (79 RCTs) | ⨁⨁⨁◯ Moderate^b^ | Addition of lower limb strength training as a component of pulmonary rehabilitation may result in no difference in exercise capacity. |
| Breathlessness | SMD **0.12 SD lower** (0.47 lower to 0.24 higher) | 4038 (57 RCTs) | ⨁⨁⨁◯ Moderate^b^ | Addition of lower limb strength training as a component of pulmonary rehabilitation may result in no difference in breathlessness. |
| ***The risk in the intervention group** (and its 95% credible interval) is based on the assumed risk in the comparison group and the **relative effect** of the intervention (and its 95% CI).  **CI:** credible interval; **SMD:** standardised mean difference | | | | |
| **GRADE Working Group grades of evidence** **High certainty:** we are very confident that the true effect lies close to that of the estimate of the effect. **Moderate certainty:** we are moderately confident in the effect estimate: the true effect is likely to be close to the estimate of the effect, but there is a possibility that it is substantially different. **Low certainty:** our confidence in the effect estimate is limited: the true effect may be substantially different from the estimate of the effect. **Very low certainty:** we have very little confidence in the effect estimate: the true effect is likely to be substantially different from the estimate of effect. | | | | |

#### Explanations

1. High risk of bias in 60 out of 198 studies with reduction in effect seen once studies at high risk of bias removed.
2. Imprecise estimate with wide credible interval

| **Supplementary table 13 - Summary of findings:** | | | | | |
| --- | --- | --- | --- | --- | --- |
| **Very high intensity aerobic exercise compared to low intensity aerobic exercise in pulmonary rehabilitation for people with COPD** | | | | | |
| **Patient or population:** Pulmonary rehabilitation for COPD  **Setting:** Programmes of a minimum frequency of twice a week for at least 3 weeks  **Intervention:** Very high intensity aerobic exercise  **Comparison:** Low intensity aerobic exercise | | | | | |
| Outcomes | **Anticipated absolute effects^*^** (95% CI) | | № of participants (studies) | Certainty of the evidence (GRADE) | Comments |
|  | **Risk with low intensity aerobic exercise** | **Risk with very high intensity aerobic exercise** |  |  |  |
| Exercise capacity | mean **0.36** SMD higher (0.1 higher to 0.62 higher) | mean **0.77 SMD higher** (0.44 higher to 1.09 higher) | 1911 (33 RCTs) | ⨁⨁◯◯ Low^a,b^ | The evidence suggests very high intensity aerobic exercise may result in a greater increase in exercise capacity than low intensity exercise. |
| Health related quality of life | mean **0.01** SMD higher (0.3 lower to 0.32 higher) | mean **0.16 SMD higher** (0.25 lower to 0.57 higher) | 1721 (20 RCTs) | ⨁⨁◯◯ Low^b,c^ | Very high intensity aerobic exercise may not result in greater improvements in health-related quality of life than low intensity exercise. |
| Breathlessness | mean **0.11** SMD lower (0.19 lower to 0.41 higher) | mean **0.37 SMD higher** (0.03 lower to 0.71 higher) | 1761 (19 RCTs) | ⨁⨁◯◯ Low^b,d^ | The evidence suggests very high intensity aerobic exercise may result in a greater increase in breathlessness than low intensity exercise. |
| ***The risk in the intervention group** (and its 95% credible interval) is based on the assumed risk in the comparison group and the **relative effect** of the intervention (and its 95% CI).  **CI:** credible interval; **SMD:** standardised mean difference | | | | | |
| **GRADE Working Group grades of evidence** **High certainty:** we are very confident that the true effect lies close to that of the estimate of the effect. **Moderate certainty:** we are moderately confident in the effect estimate: the true effect is likely to be close to the estimate of the effect, but there is a possibility that it is substantially different. **Low certainty:** our confidence in the effect estimate is limited: the true effect may be substantially different from the estimate of the effect. **Very low certainty:** we have very little confidence in the effect estimate: the true effect is likely to be substantially different from the estimate of effect. | | | | | |

#### Explanations

a. High risk of bias in 22 of 32 studies. The effect of high and low intensity training was significantly reduced once studies at risk of bias were removed.

b. Imprecise estimate with wide credible intervals

c. High risk of bias in 11 of 19 studies. The effect of high and moderate intensity aerobic training was greater when restricting to only studies at low risk of bias.

d. High risk of bias in 11 of 18 studies. The effect of very high and low intensity aerobic training was reduced when restricting to only studies at low risk of bias.

| **Supplementary table 14 -** Summary of findings: | | | | |
| --- | --- | --- | --- | --- |
| **Chinese exercise training and yoga compared to no Chinese exercise training and yoga in pulmonary rehabilitation for people with COPD** | | | | |
| **Patient or population:** Pulmonary rehabilitation for COPD  **Setting:** Programmes of a minimum frequency of twice a week for at least 3 weeks  **Intervention:** Chinese exercise training and yoga  **Comparison:** no Chinese exercise training and yoga | | | | |
| Outcomes | **Anticipated absolute effects^*^** (95% CI) | № of participants (studies) | Certainty of the evidence (GRADE) | Comments |
| Exercise capacity | SMD **0.38 SD higher** (0.06 higher to 0.7 higher) | 2123 (22 RCTs) | ⨁⨁⨁◯ Moderate ^a,b^ | Chinese exercise training and yoga likely increases exercise capacity slightly. |
| Health related quality of life | SMD **0.79 SD higher** (0.45 higher to 1.13 higher) | 2448 (22 RCTs) | ⨁⨁⨁⨁ High^a^ | Chinese exercise training and yoga improves health related quality of life. |
| Breathlessness | SMD **0.59 SD higher** (0.20 higher to 0.99 higher) | 985 (9 RCTs) | ⨁⨁⨁◯ Moderate^c^ | Chinese exercise training and yoga increases breathlessness. |
| ***The risk in the intervention group** (and its 95% credible interval) is based on the assumed risk in the comparison group and the **relative effect** of the intervention (and its 95% CI).  **CI:** credible interval; **SMD:** standardised mean difference | | | | |
| **GRADE Working Group grades of evidence** **High certainty:** we are very confident that the true effect lies close to that of the estimate of the effect. **Moderate certainty:** we are moderately confident in the effect estimate: the true effect is likely to be close to the estimate of the effect, but there is a possibility that it is substantially different. **Low certainty:** our confidence in the effect estimate is limited: the true effect may be substantially different from the estimate of the effect. **Very low certainty:** we have very little confidence in the effect estimate: the true effect is likely to be substantially different from the estimate of effect. | | | | |

#### Explanations

a. Few studies at high risk of bias. Increase in effect once studies at high risk of bias removed

b. Imprecise estimate with wide credible intervals

c. Reduced effect once studies at high risk of bias removed

## Sensitivity analyses

Removal of studies in which between 80-99% of participants (n=3) had no effect on the results. Removal of optional components had no impact on the results. Models using a correlation coefficient of 0.5 for imputation of standard deviations had no effect on results. When studies which did not include a practice exercise test at baseline were removed (77% of included studies), the effect of in-person and remote supervision was reduced and the effect of psychological interventions increased. When limiting to studies that performed a measure of peak exercise capacity such as peak workload or the incremental shuttle walk test rather than a functional measure such as the 6-minute walk test, the effect of direct supervision was reduced in interim model but not the final model.

**
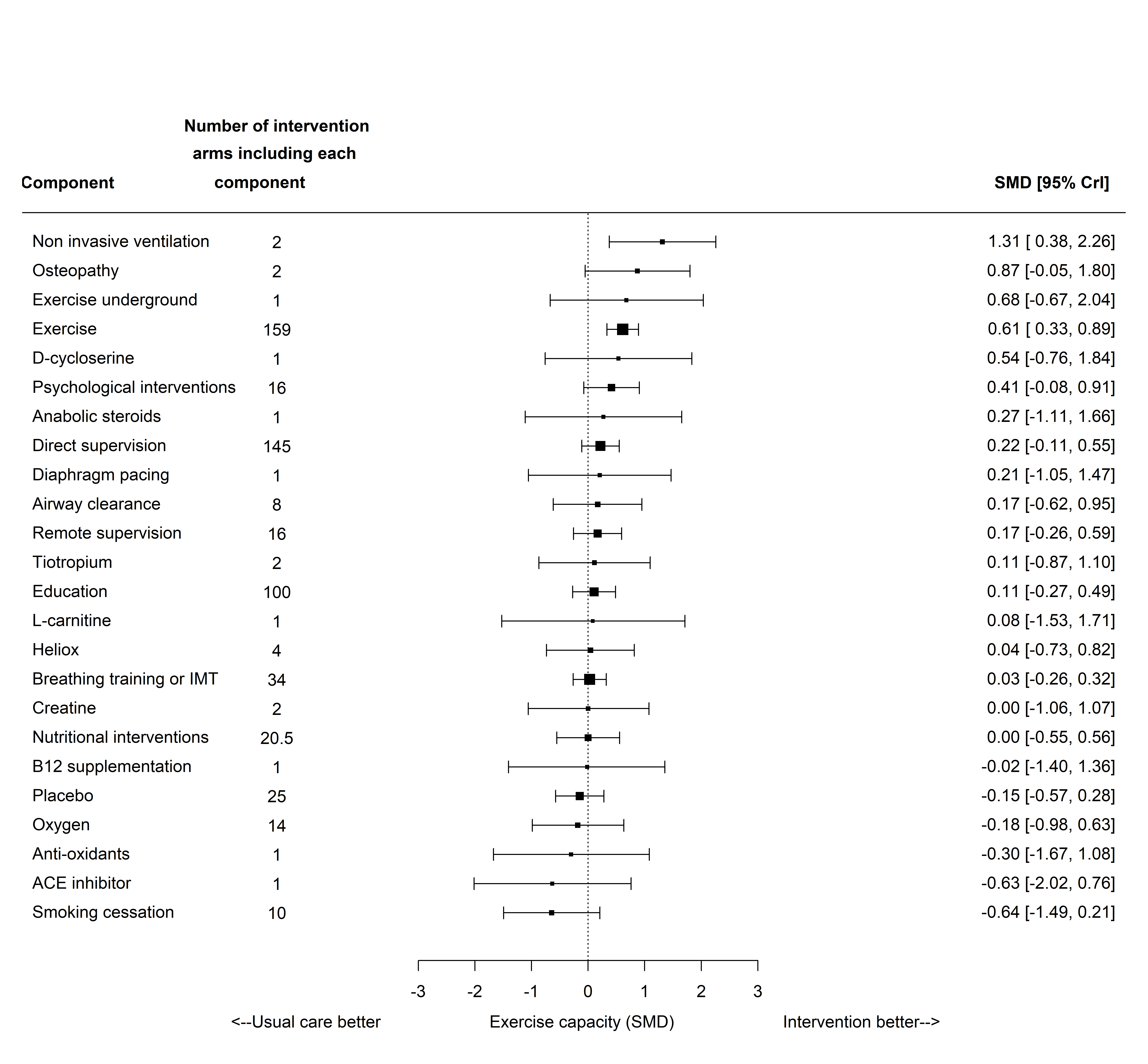
**

**Supplementary figure 57 –** Interim model, exercise capacity, sensitivity analysis, studies at high risk of bias removed

**
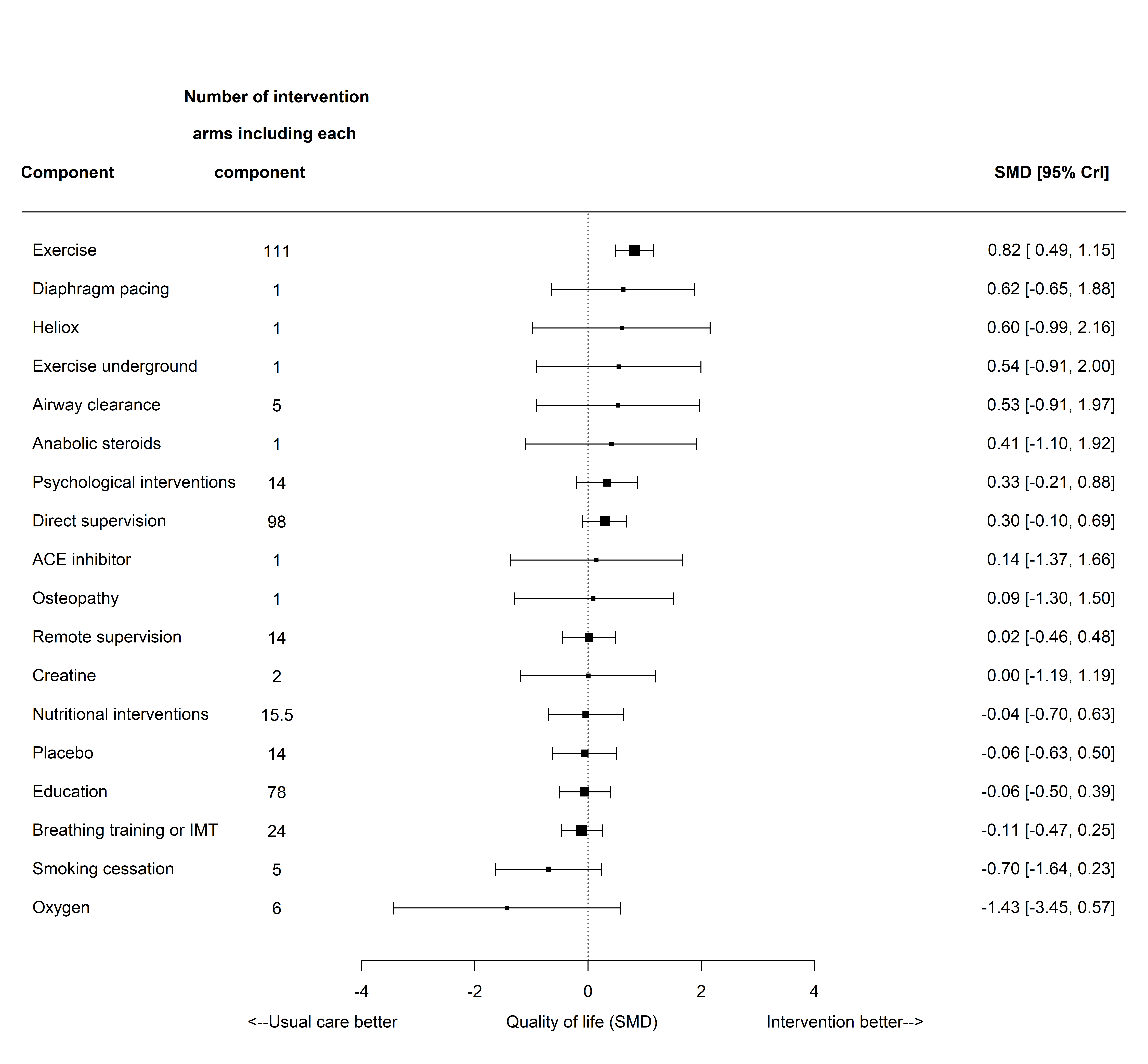
**

**Supplementary figure 58 –** Interim model, quality of life, sensitivity analysis, studies at high risk of bias removed

**
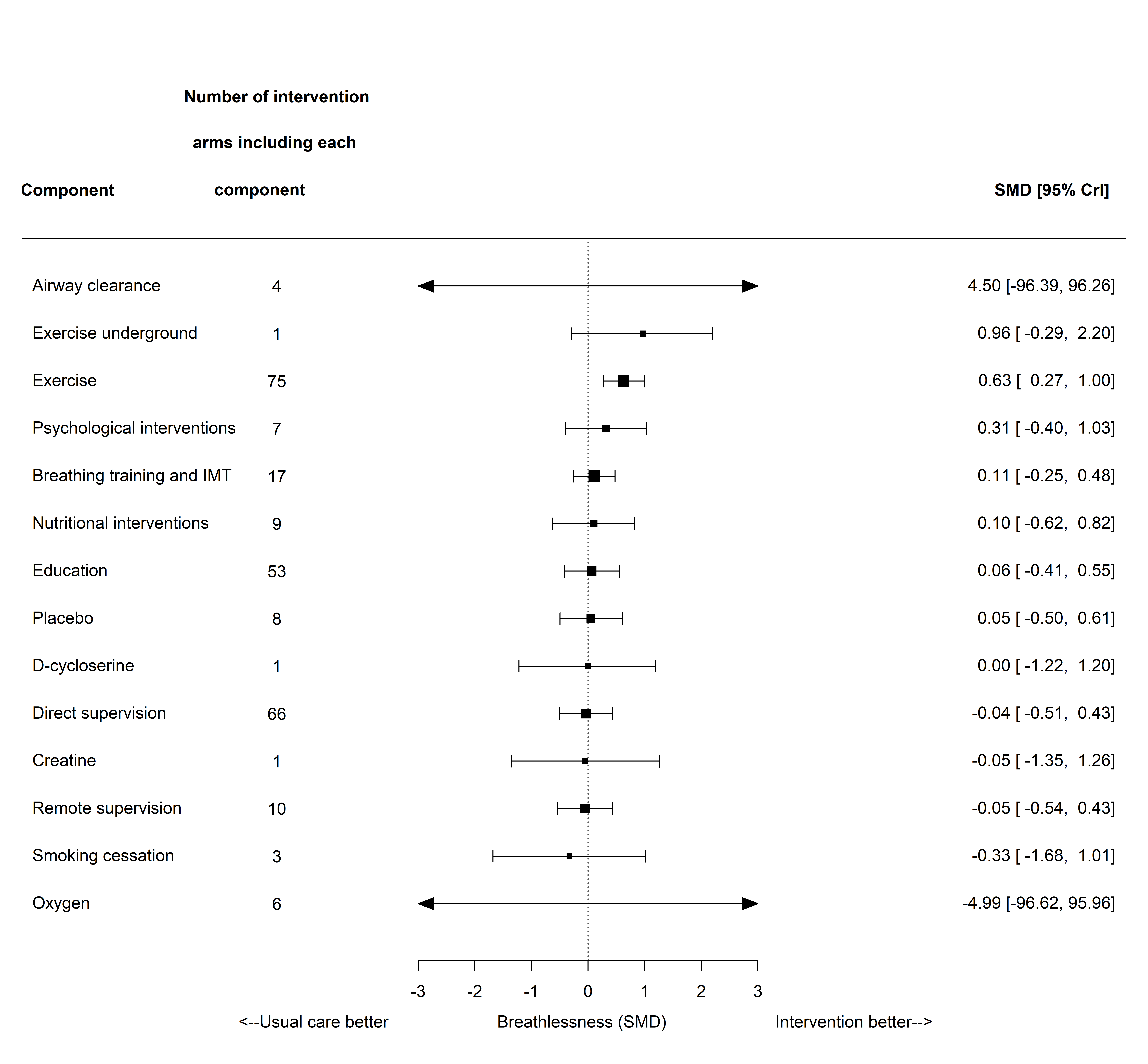
**

**Supplementary figure 59 –** Interim model, breathlessness, sensitivity analysis, studies at high risk of bias removed

**
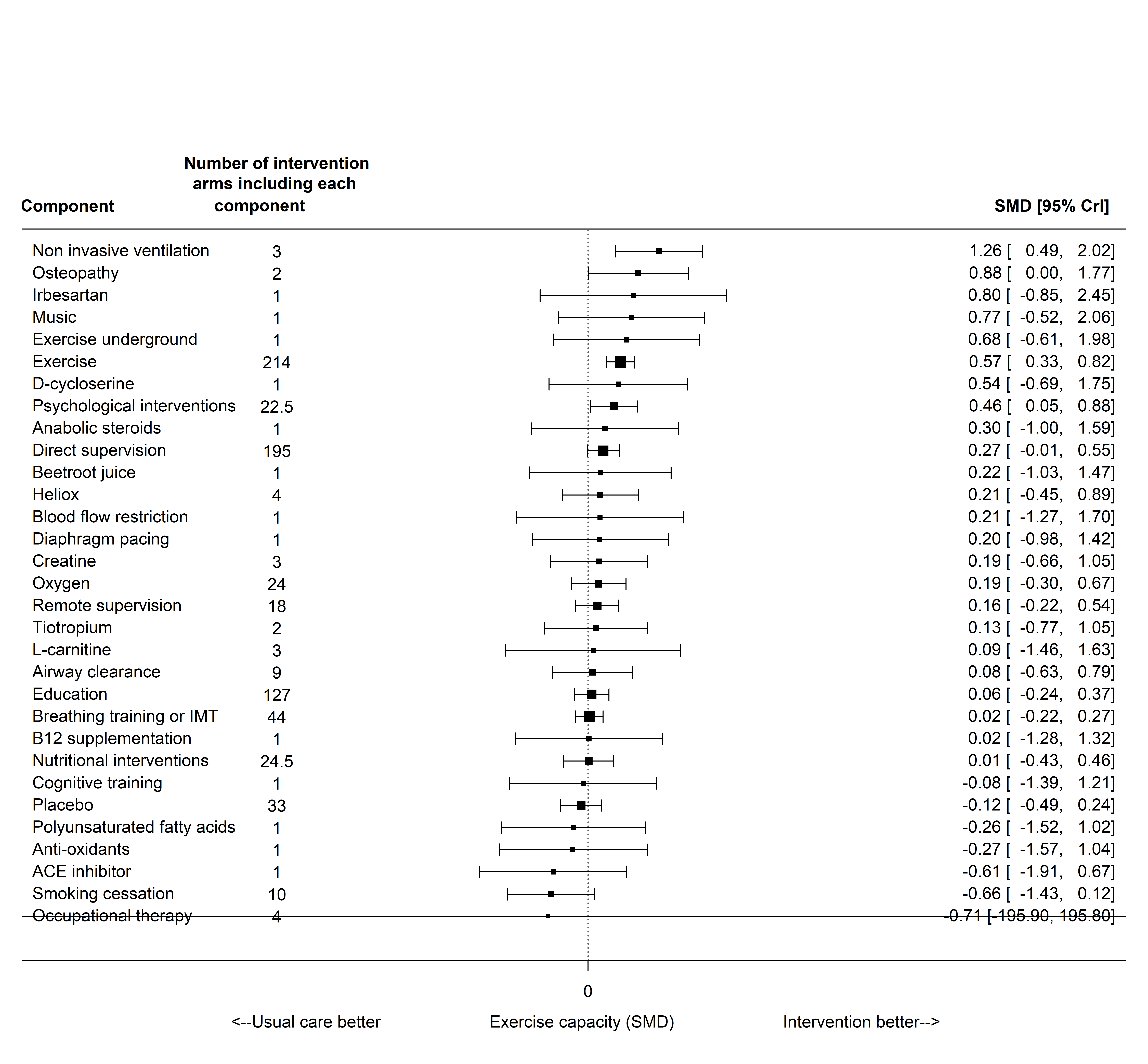
**

**Supplementary figure 60 –** Interim model, exercise capacity, sensitivity analysis, studies without blinding of outcome assessors removed

**
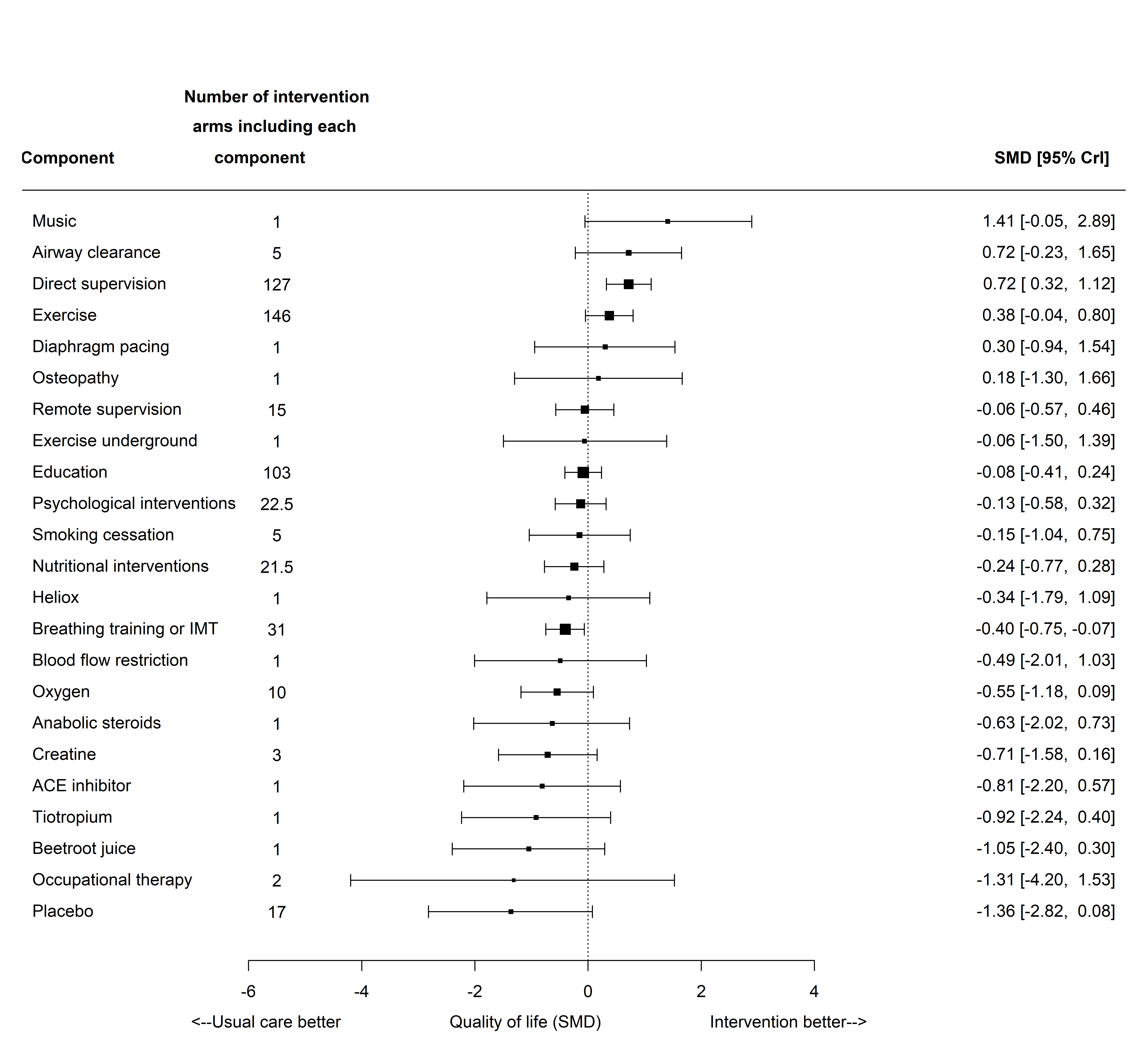
**

**Supplementary figure 61 –** Interim model, quality of life, sensitivity analysis, studies without blinding of outcome assessors removed

**
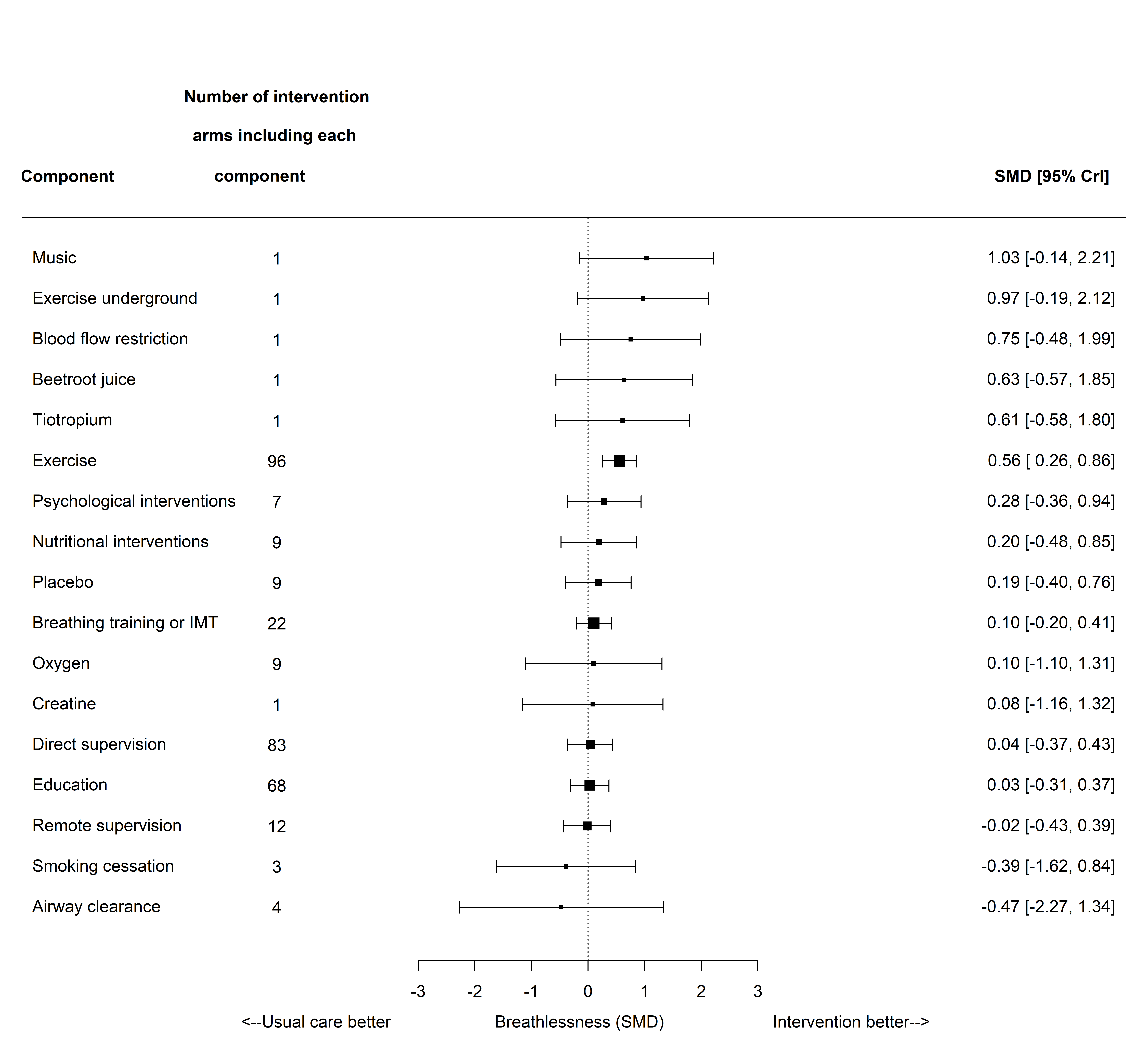
**

**Supplementary figure 62 –** Interim model, breathlessness, sensitivity analysis, studies without blinding of outcome assessors removed

**
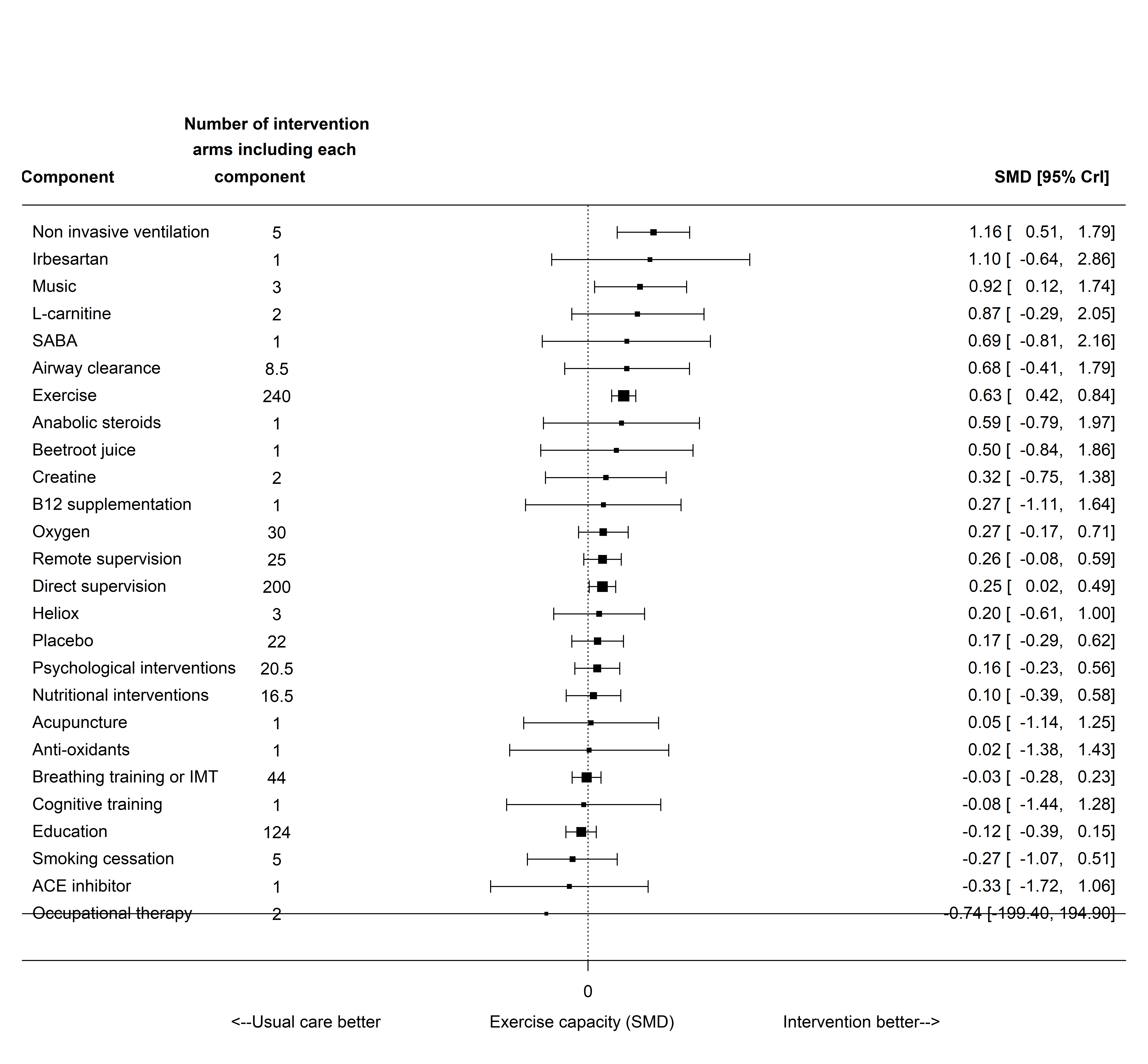
**

**Supplementary figure 63 –** Interim model, exercise capacity, sensitivity analysis, studies with low quality of reporting scores removed

**
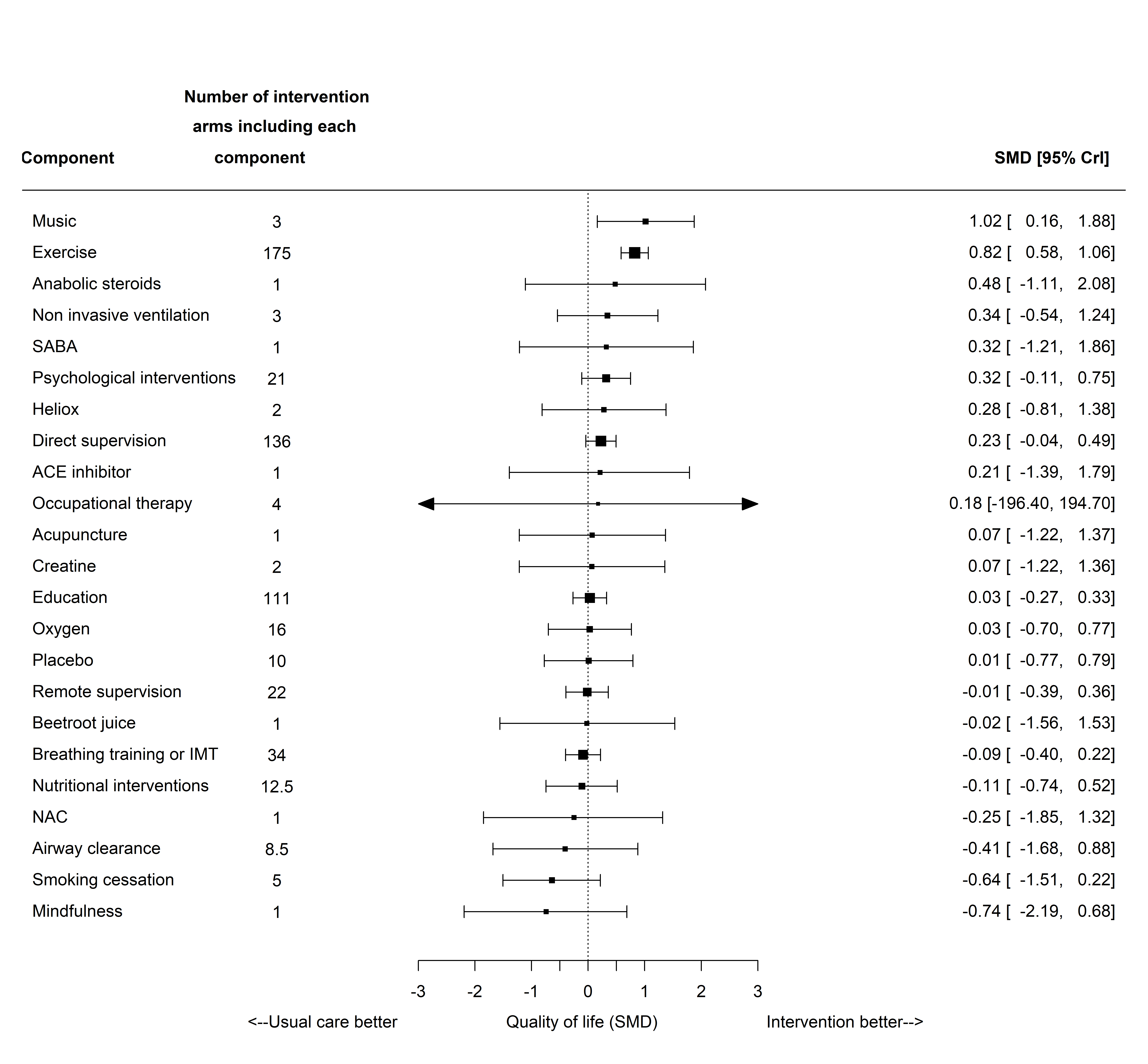
**

**Supplementary figure 64 –** Interim model, quality of life, sensitivity analysis, studies with low quality of reporting scores removed

**
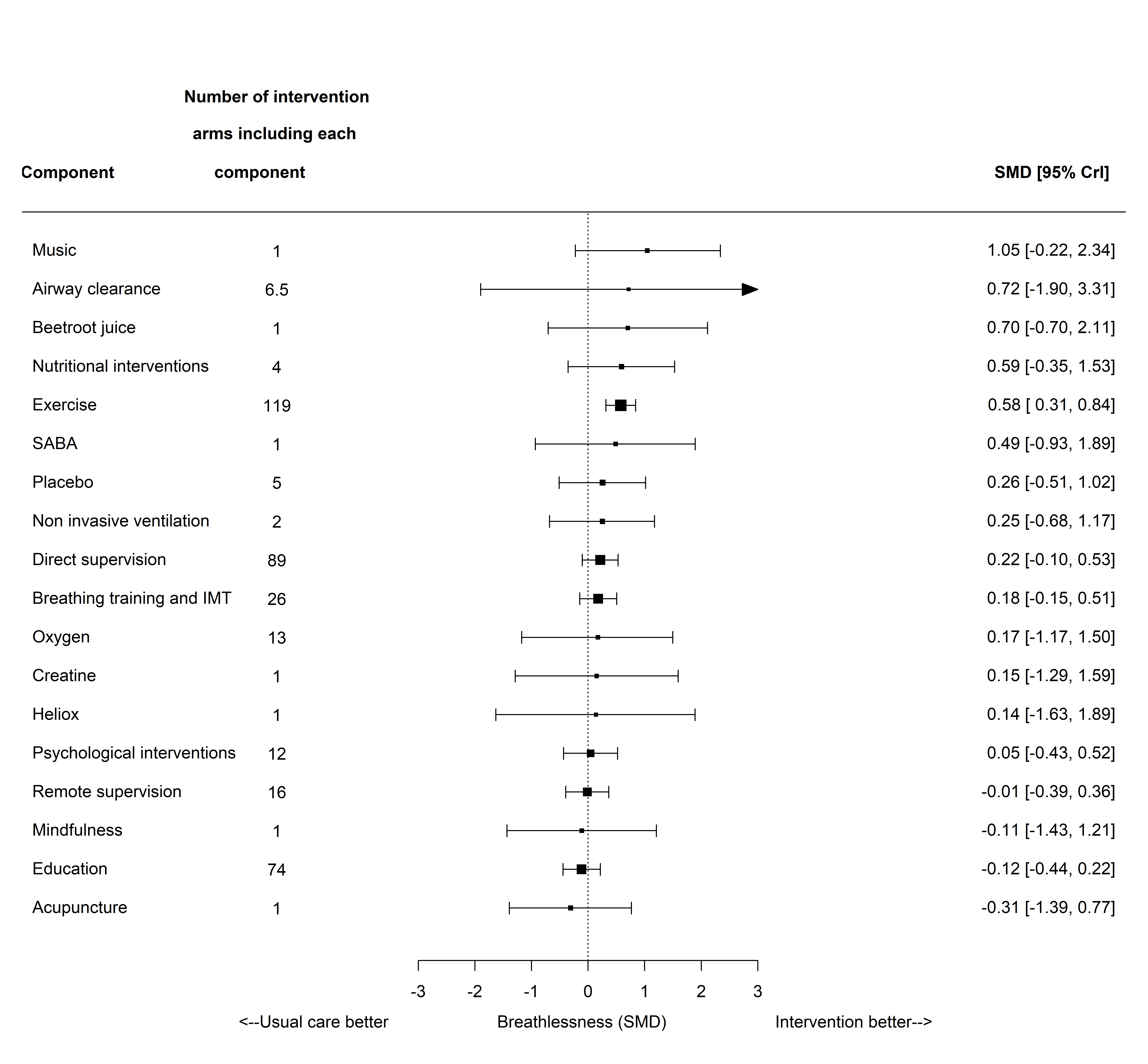
**

**Supplementary figure 65 –** Interim model, breathlessness, sensitivity analysis, studies with low quality of reporting scores removed

**
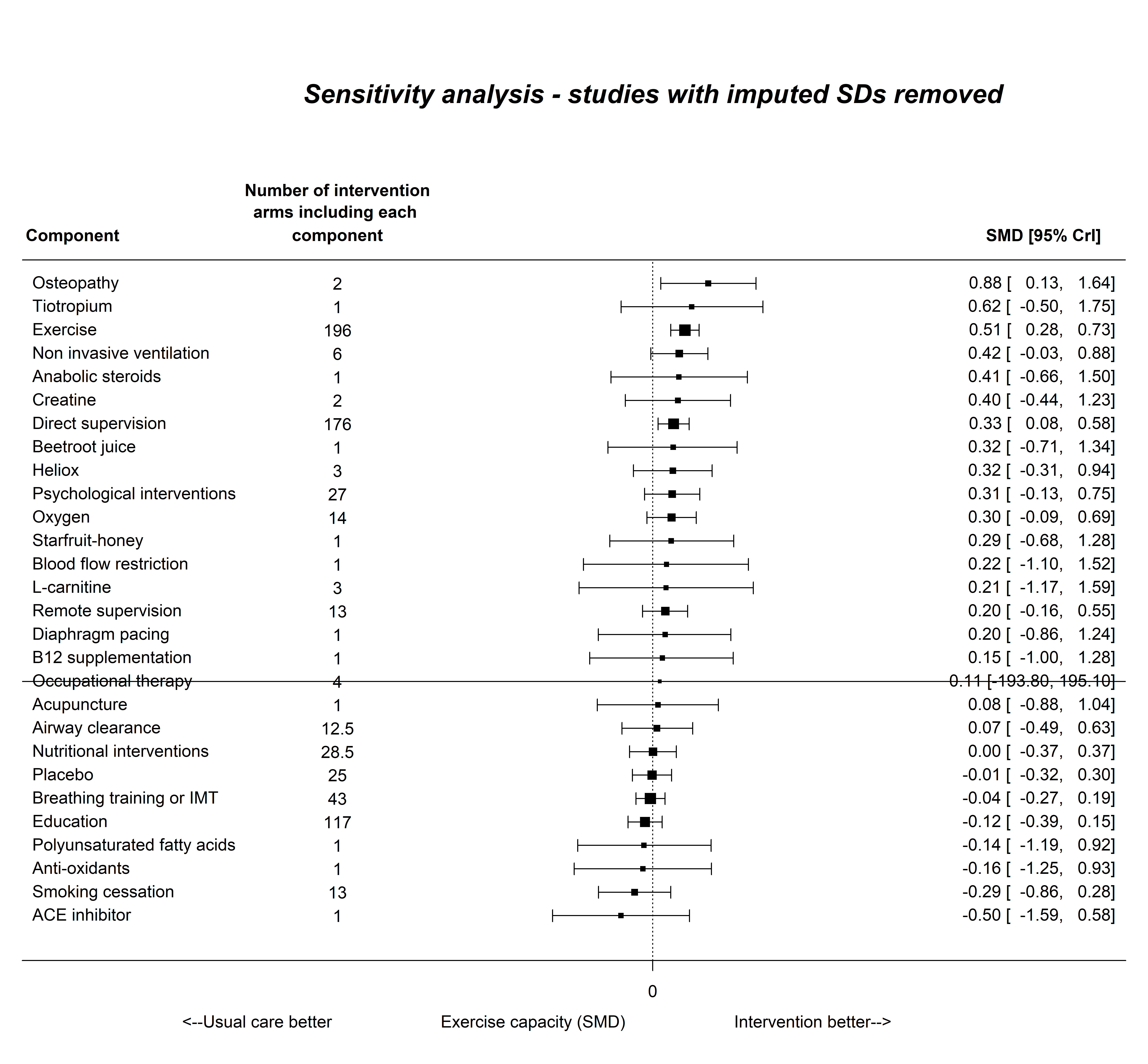
**

**Supplementary figure 66 –** Interim model, exercise capacity, sensitivity analysis, studies with imputed standard deviations removed

**
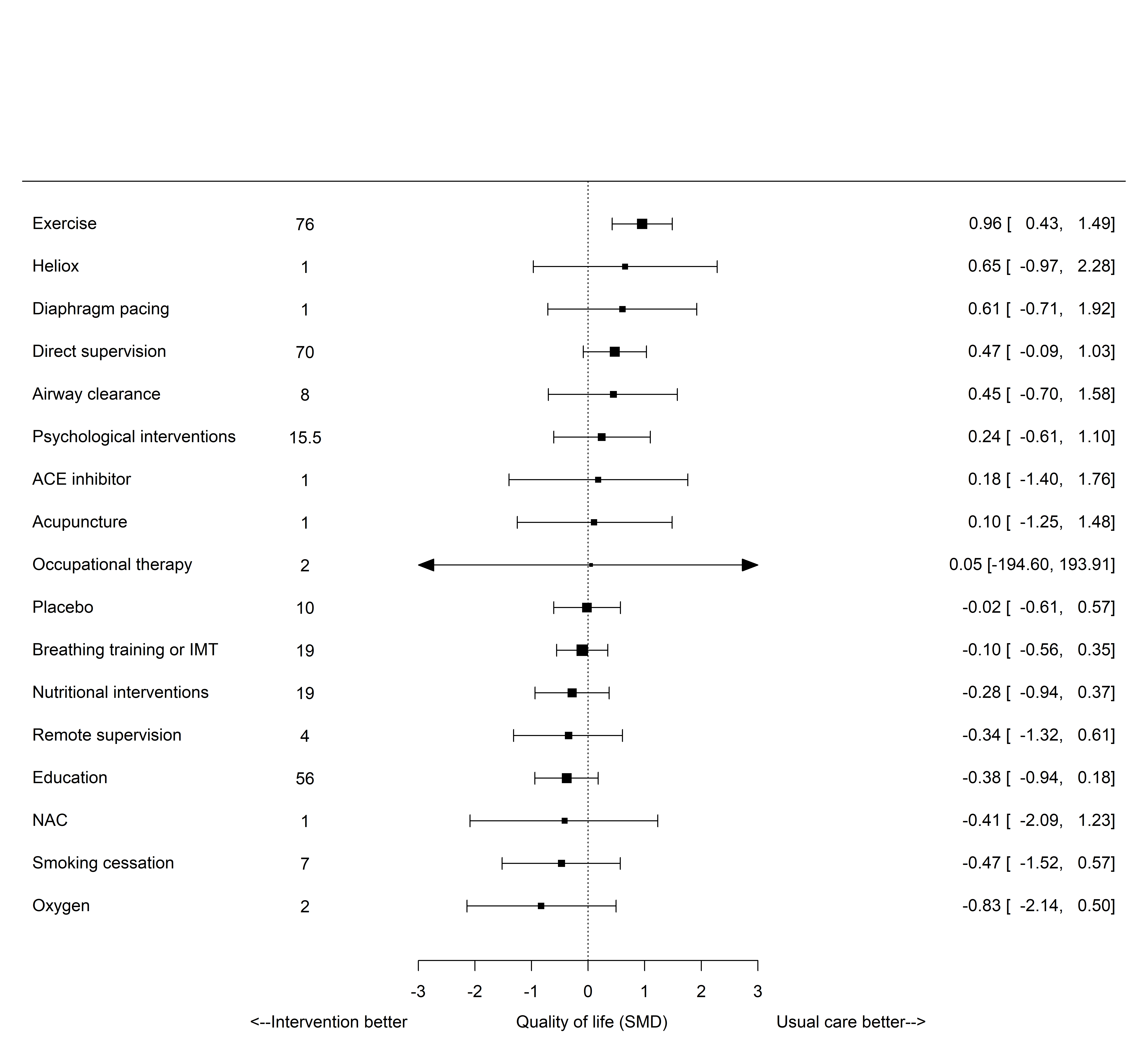
**

**Supplementary figure 67 –** Interim model, quality of life, sensitivity analysis, studies with imputed standard deviations removed

**
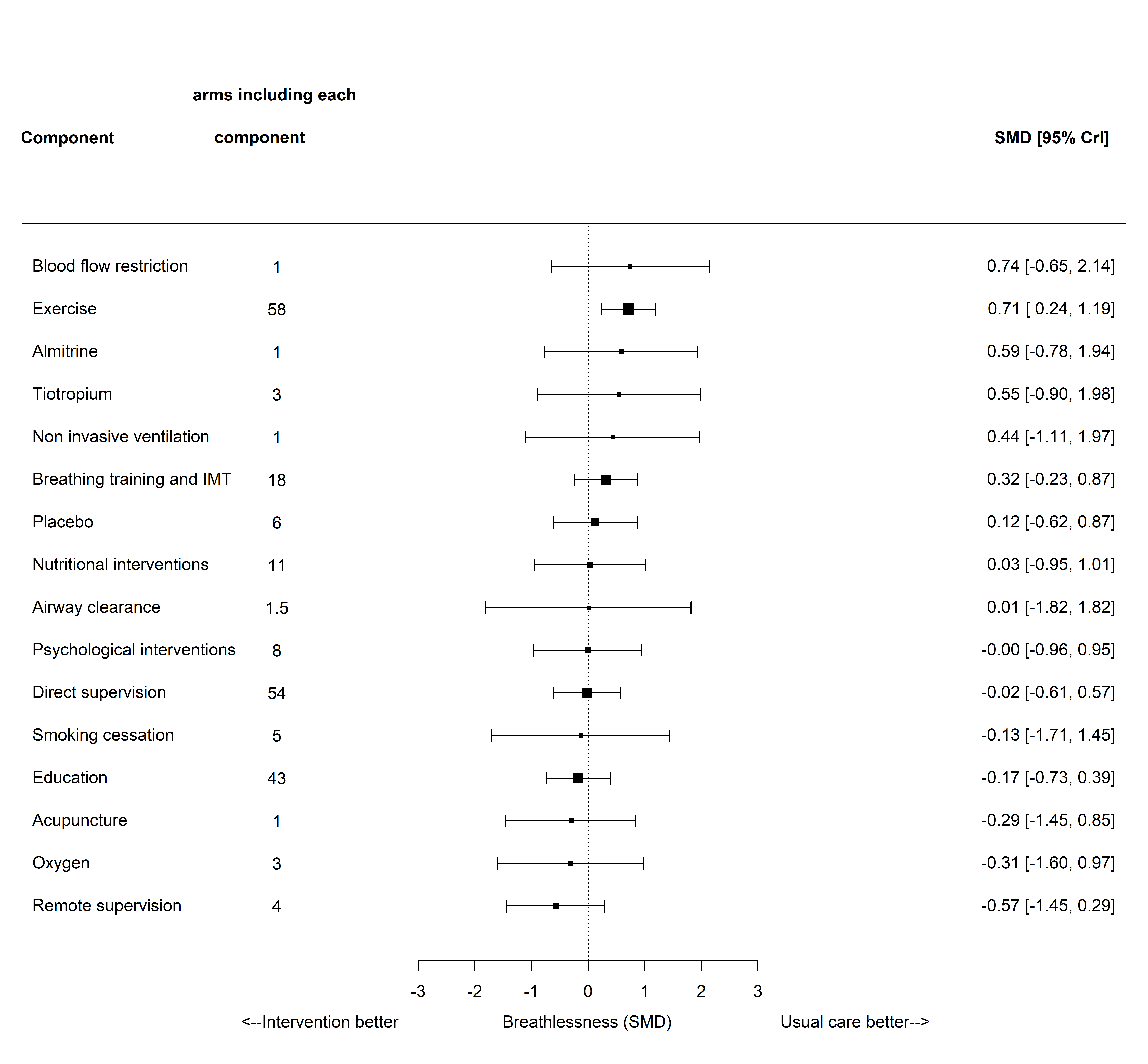
**

**Supplementary figure 68 –** Interim model, breathlessness, sensitivity analysis, studies with imputed standard deviations removed

**
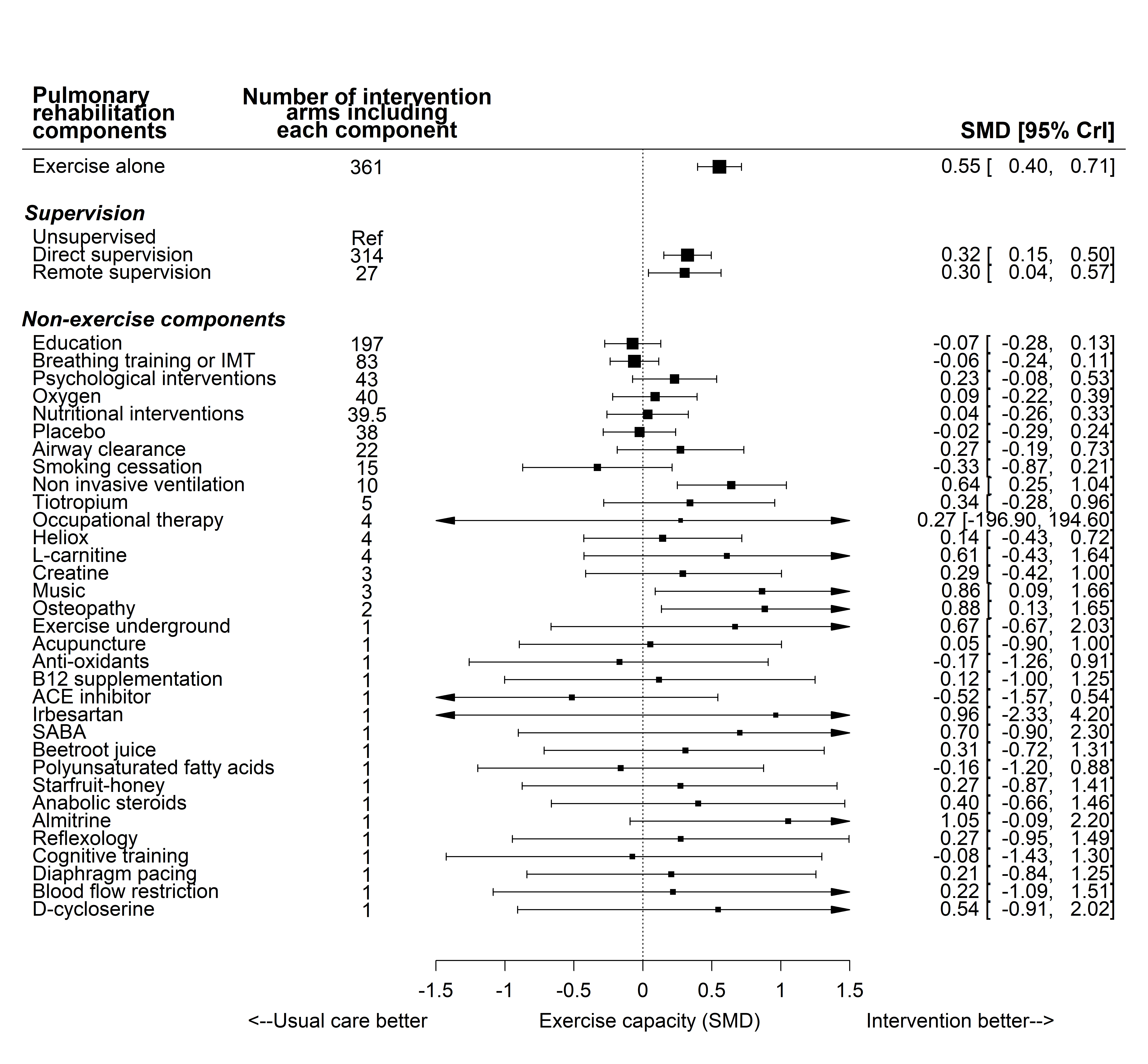
**

**Supplementary figure 69 –** Interim model, exercise capacity, sensitivity analysis, using a correlation coefficient of 0.5 for imputation of standard deviations

**
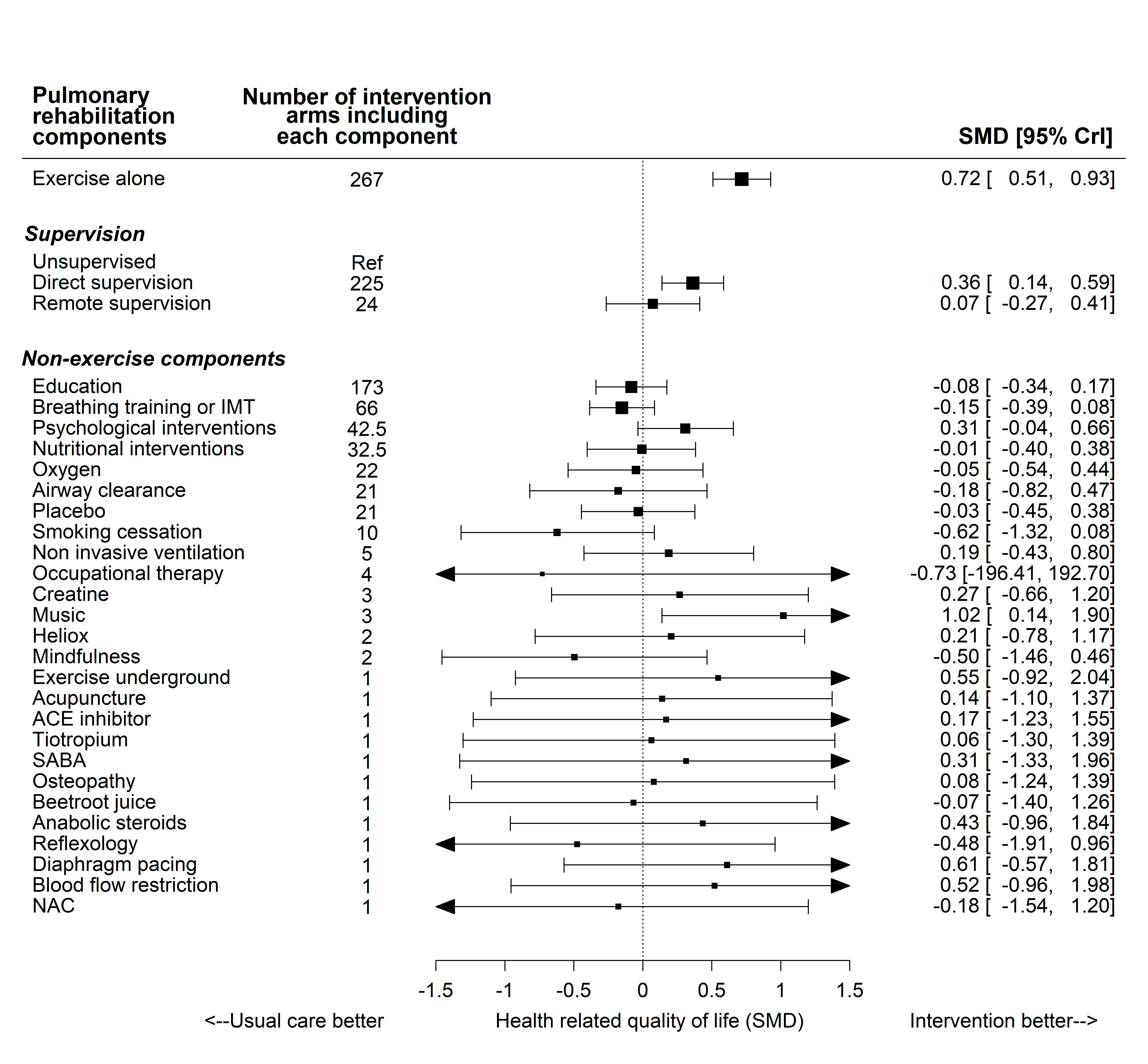
**

**Supplementary figure 70 –** Interim model, quality of life, sensitivity analysis, using a correlation coefficient of 0.5 for imputation of standard deviations

**
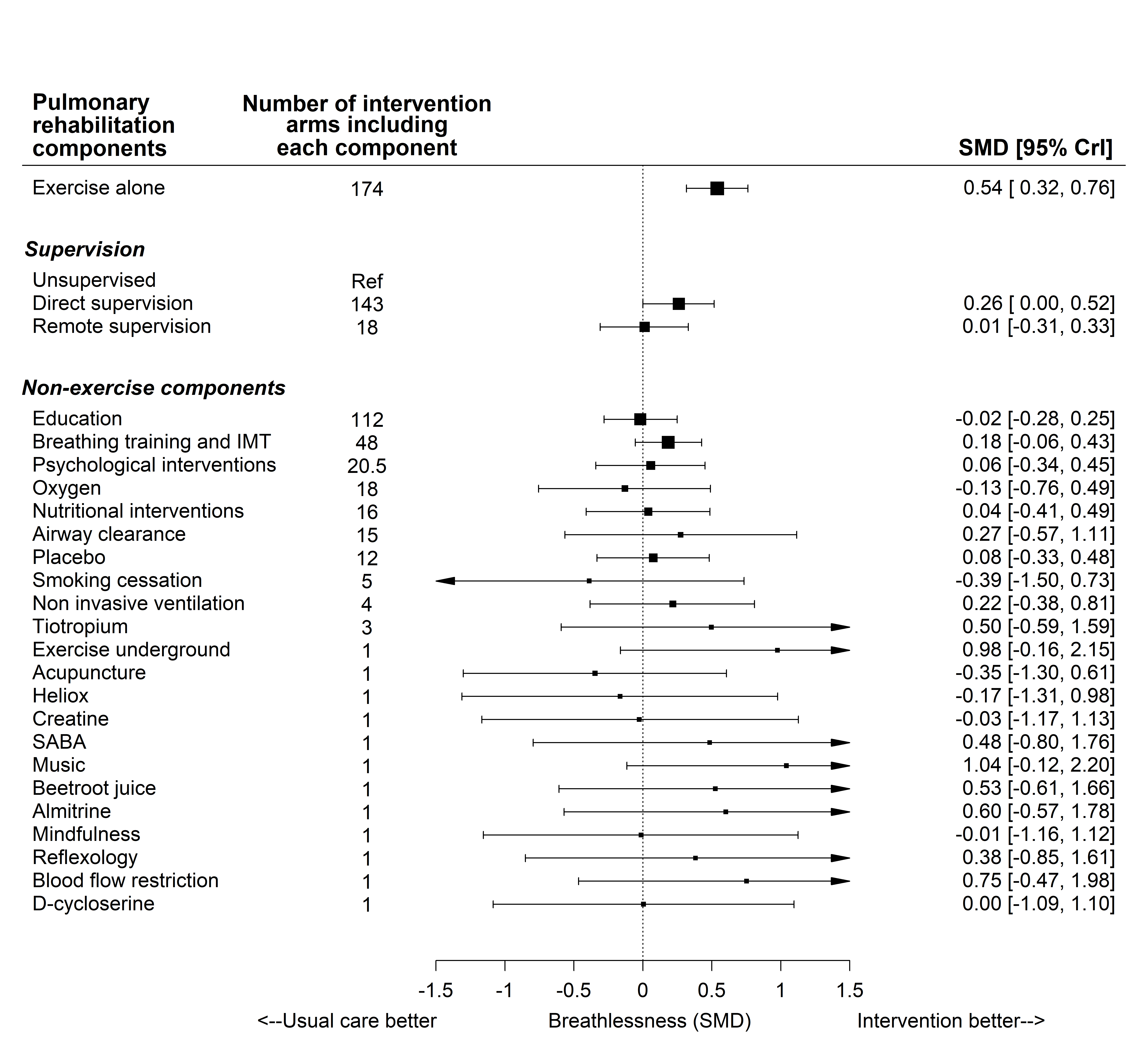
**

**Supplementary figure 71 –** Interim model, breathlessness, sensitivity analysis, using a correlation coefficient of 0.5 for imputation of standard deviations

**
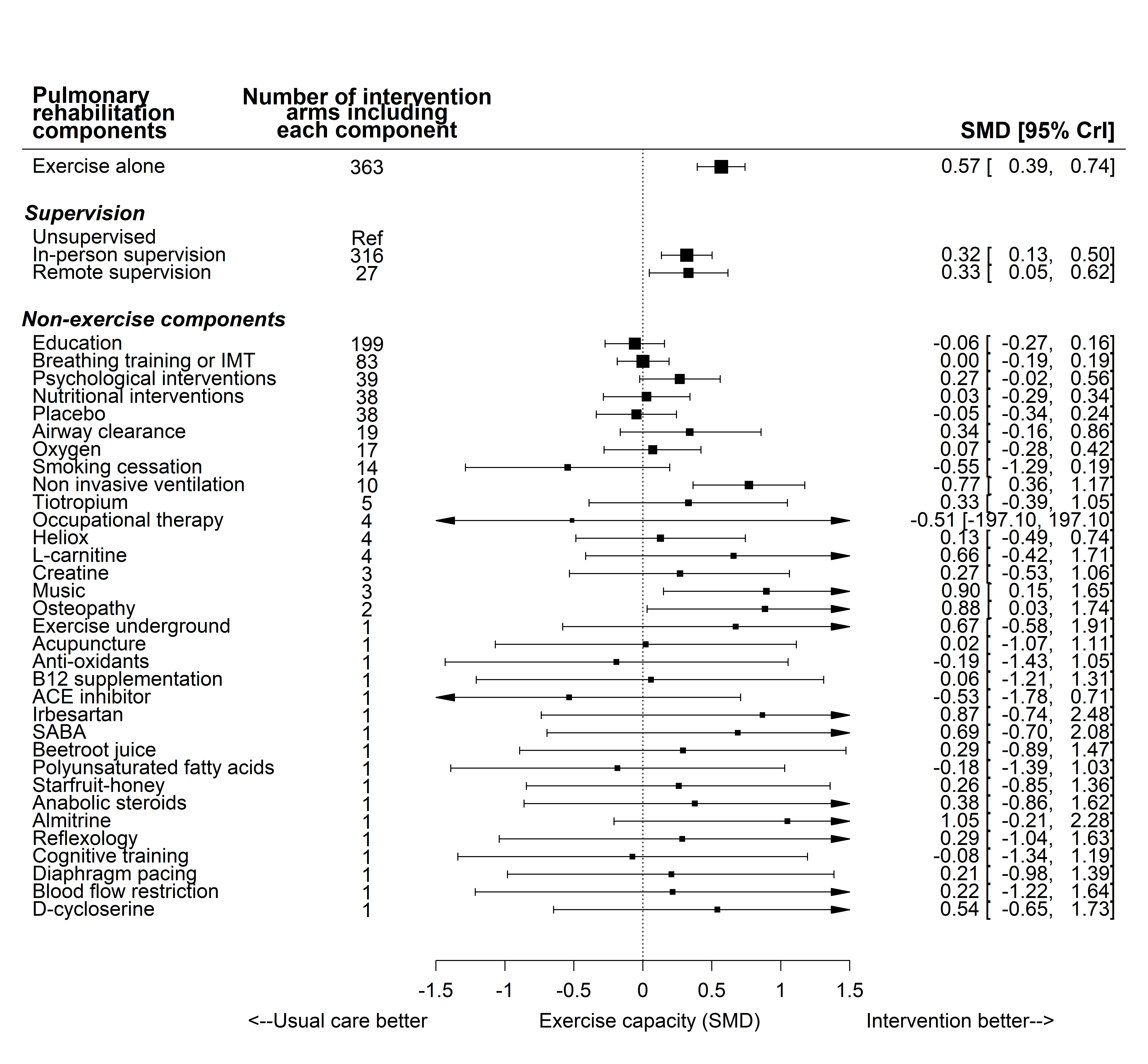
**

**Supplementary figure 72 –** Interim model, exercise capacity, sensitivity analysis, optional components removed

**
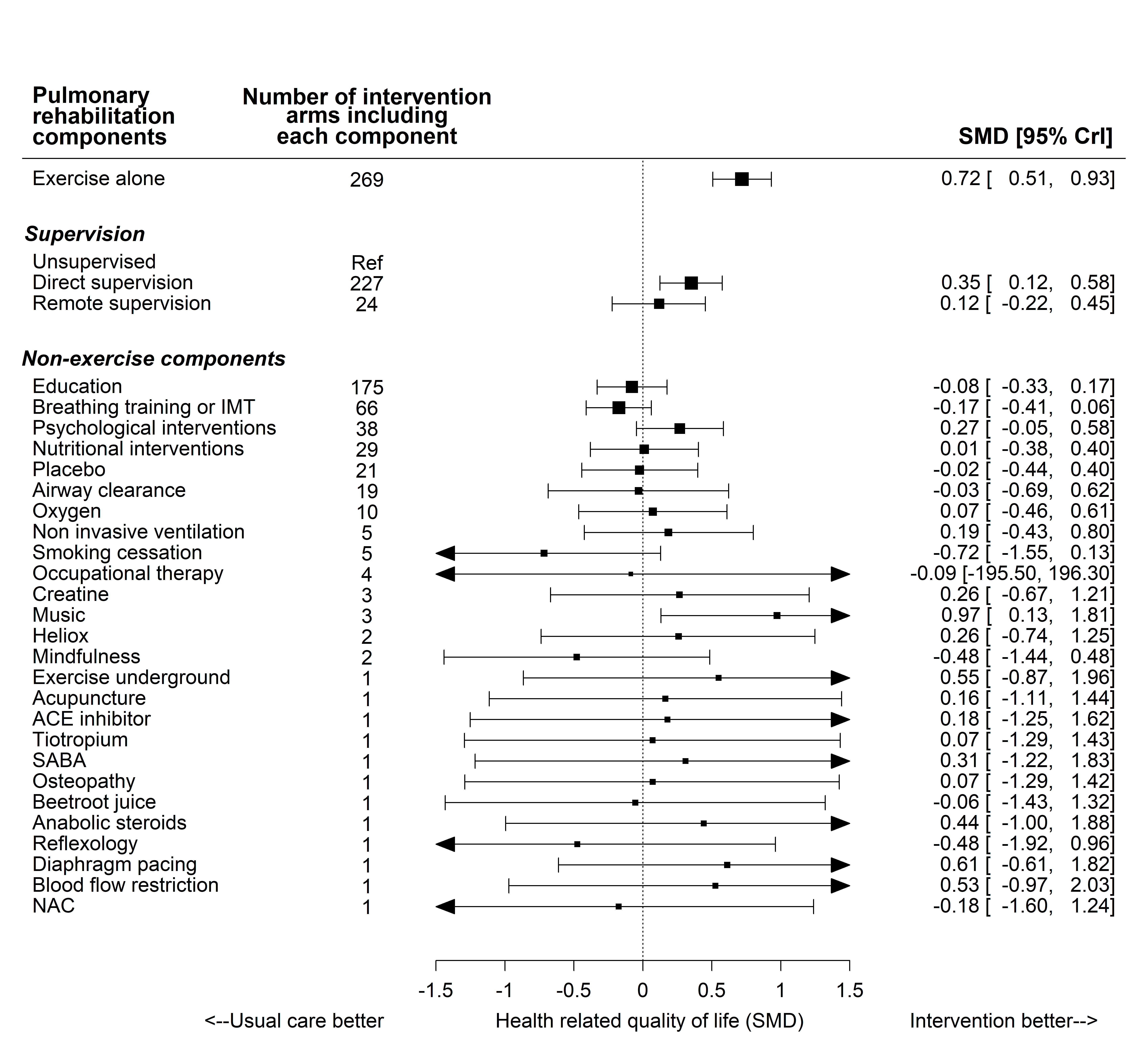
**

**Supplementary figure 73 –** Interim model, quality of life, sensitivity analysis, optional components removed

**
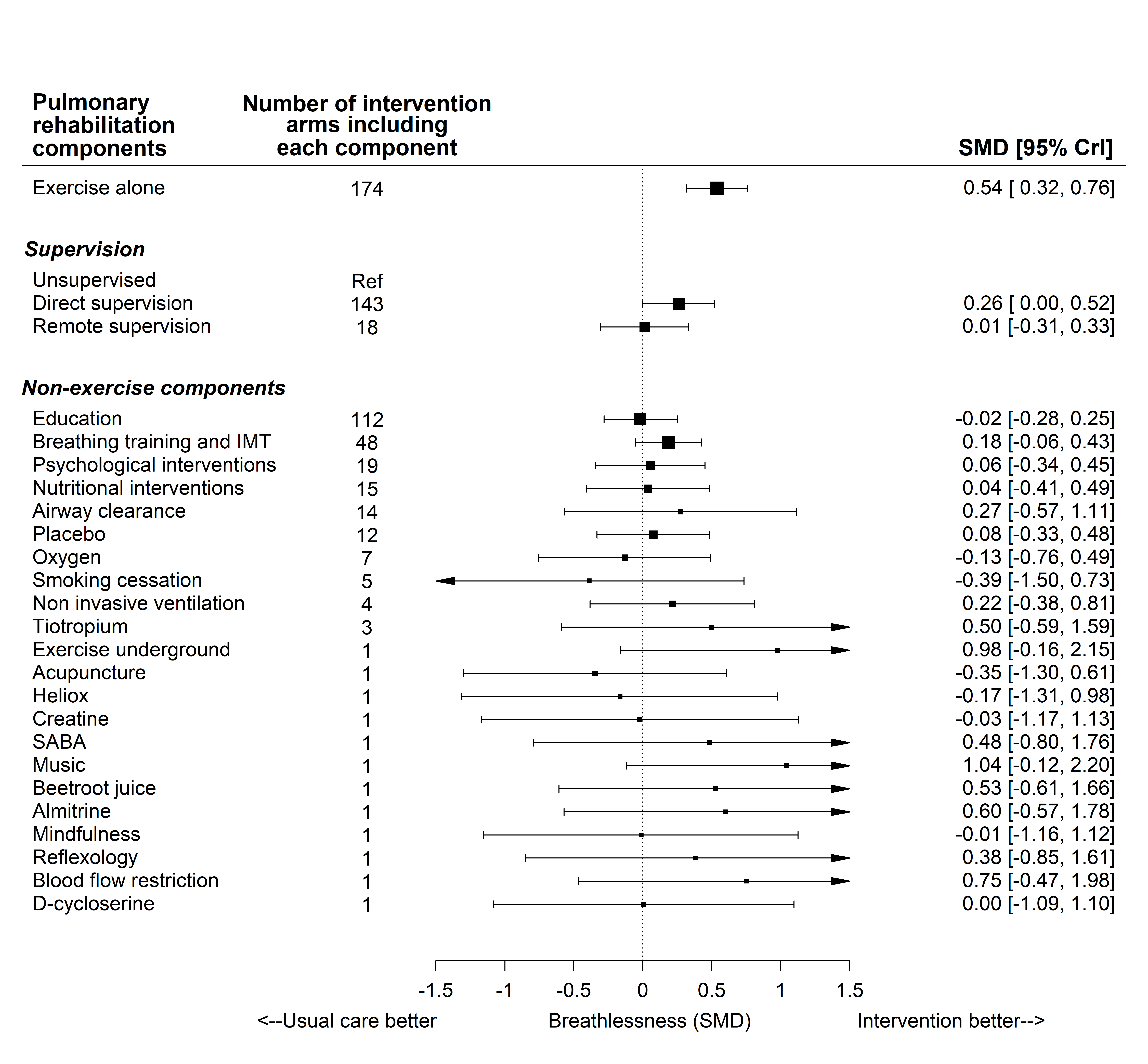
**

**Supplementary figure 74 –** Interim model, breathlessness, sensitivity analysis, optional components removed

**
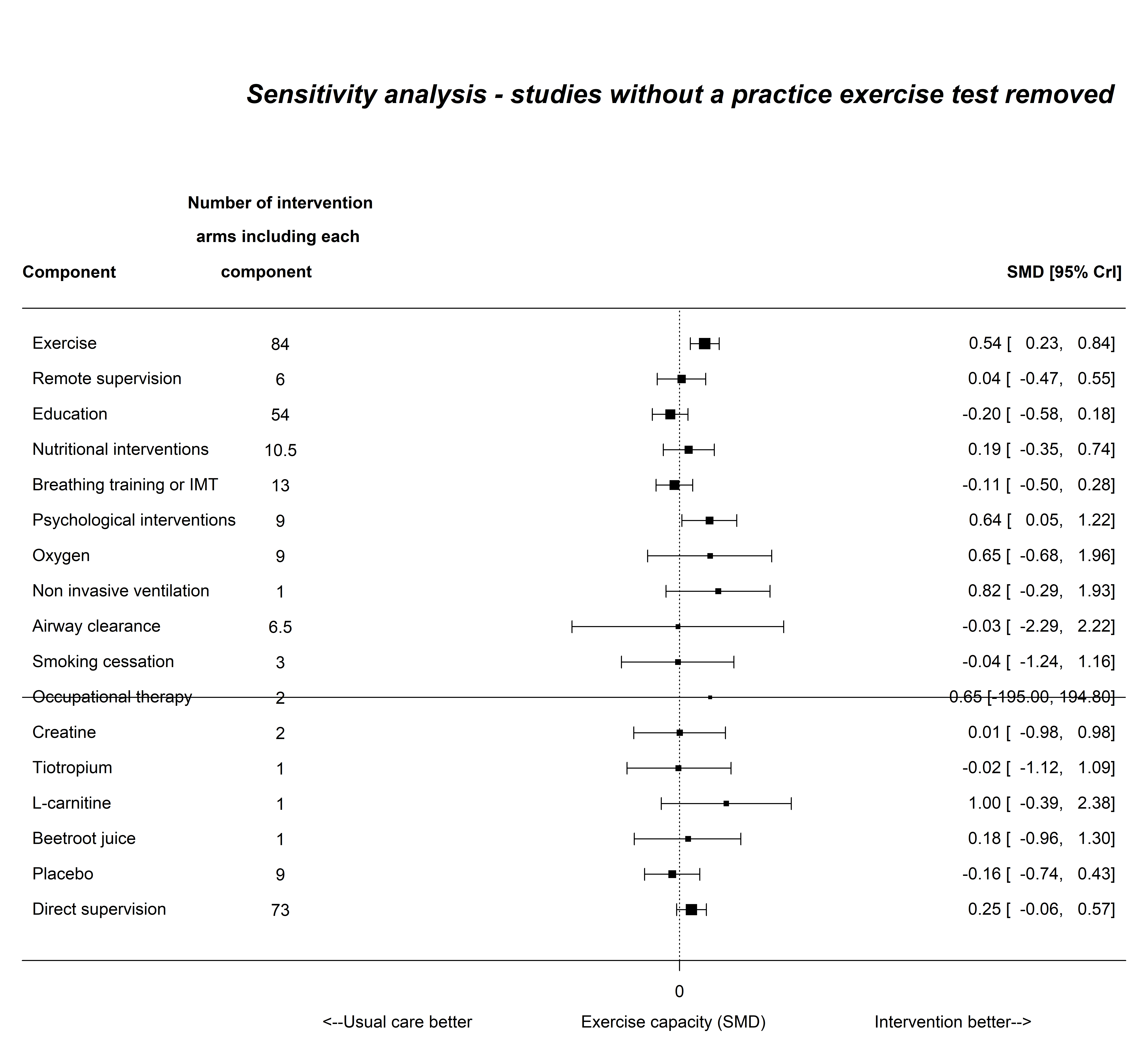
**

**Supplementary figure 75 –** Interim model, exercise capacity, sensitivity analysis, studies without a practice exercise test removed

**
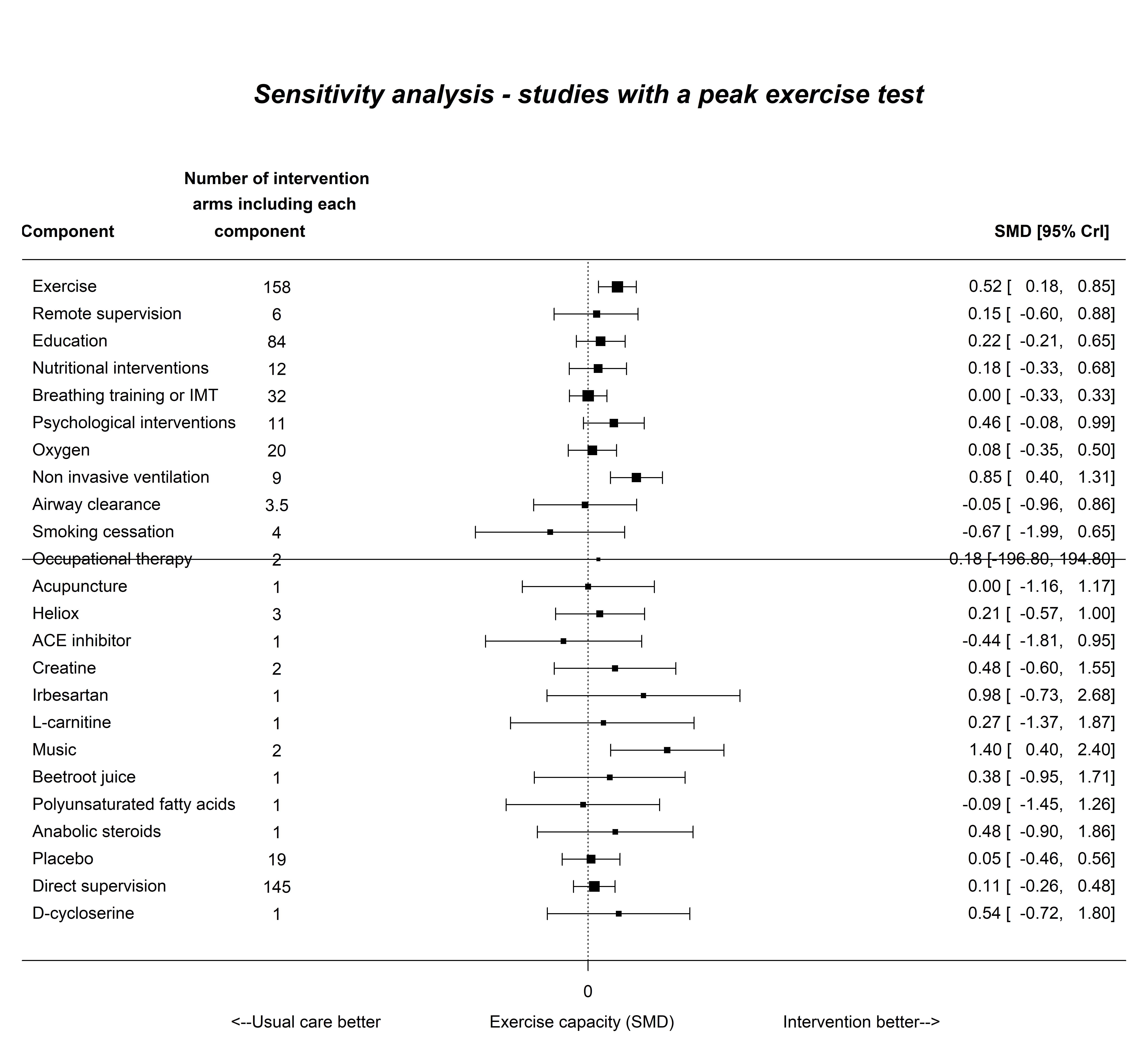
**

**Supplementary figure 76 –** Interim model, exercise capacity, sensitivity analysis, limited to studies measuring peak (rather than functional) exercise capacity

**

**

**Supplementary figure 77 –** Final model, exercise capacity, sensitivity analysis, limited to studies measuring peak exercise capacity

**

**

**Supplementary figure 78 –** Final model, exercise capacity, sensitivity analysis, high risk of bias studies excluded

**

**

**Supplementary figure 79 –** Final model, quality of life, sensitivity analysis, high risk of bias studies excluded

**

**

**Supplementary figure 80 –** Final model, breathlessness, sensitivity analysis, high risk of bias studies excluded

### **Contour adjusted funnel plots**


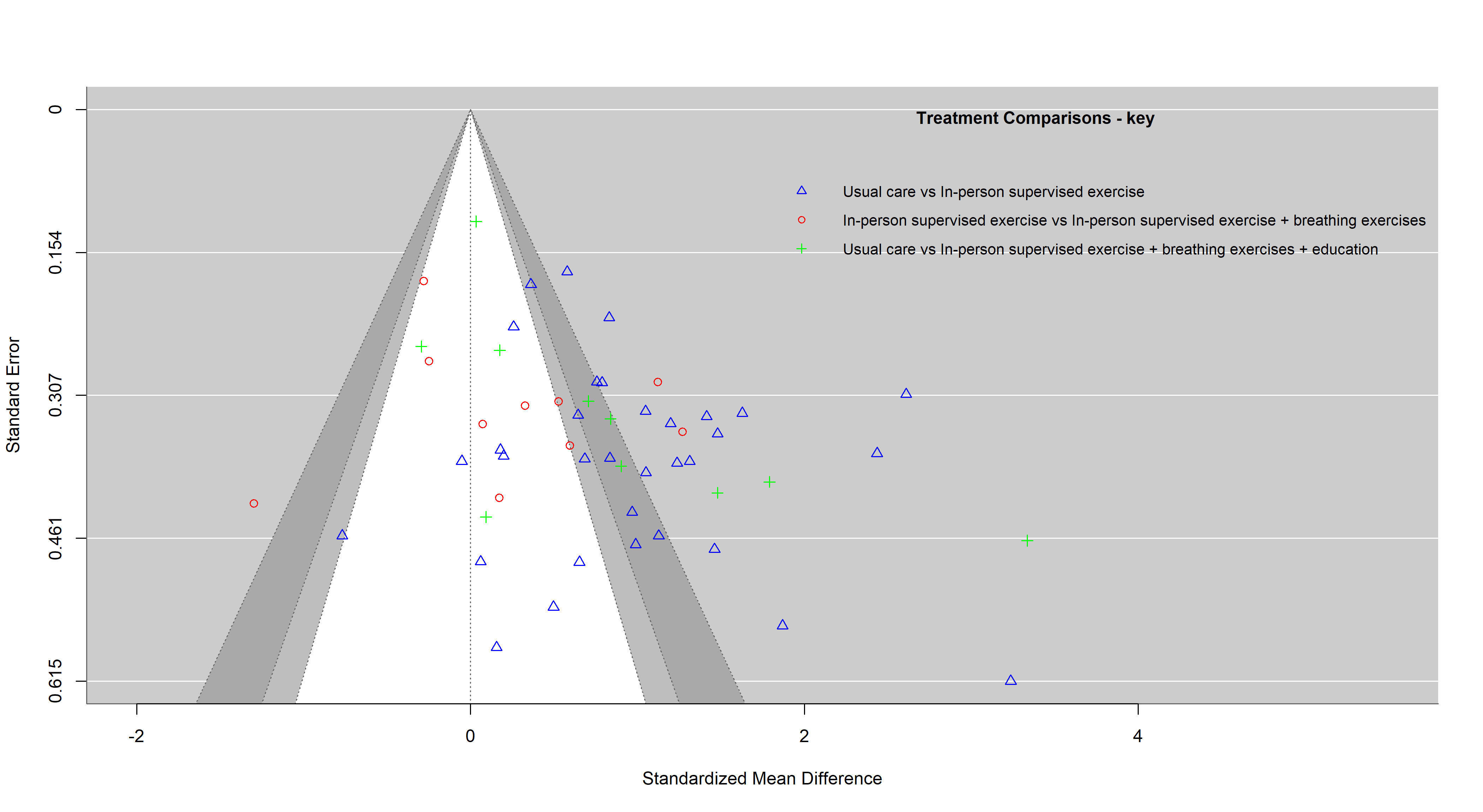


**Supplementary figure 81** – Contour adjusted funnel plot for the outcomes of exercise capacity. Only comparisons for which there were at least 10 included studies as suggested by Sterne et al.[1]


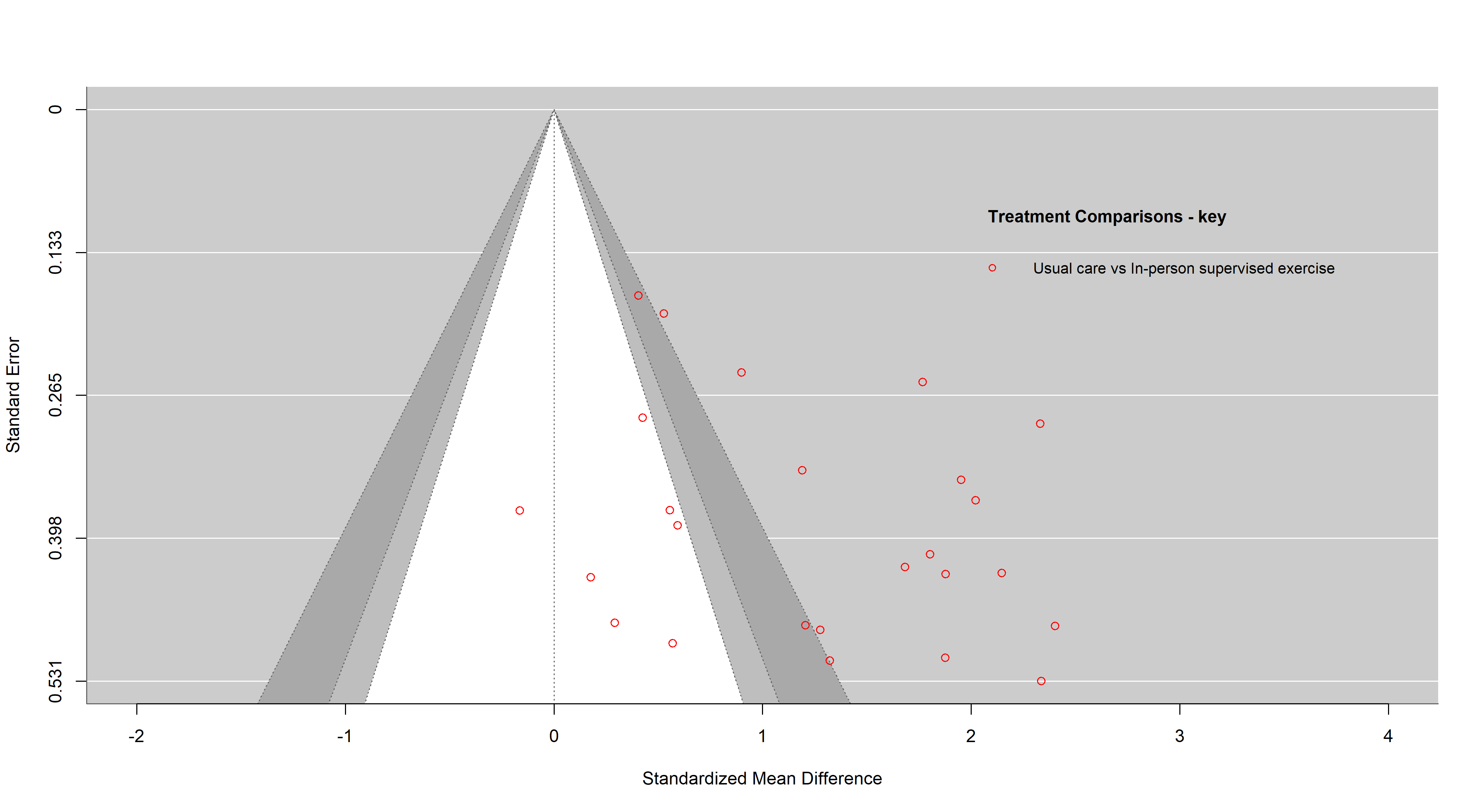


**Supplementary figure 82** – Contour adjusted funnel plot for the outcomes of health related quality of life. Only comparisons for which there were at least 10 included studies as suggested by Sterne et al.[1]


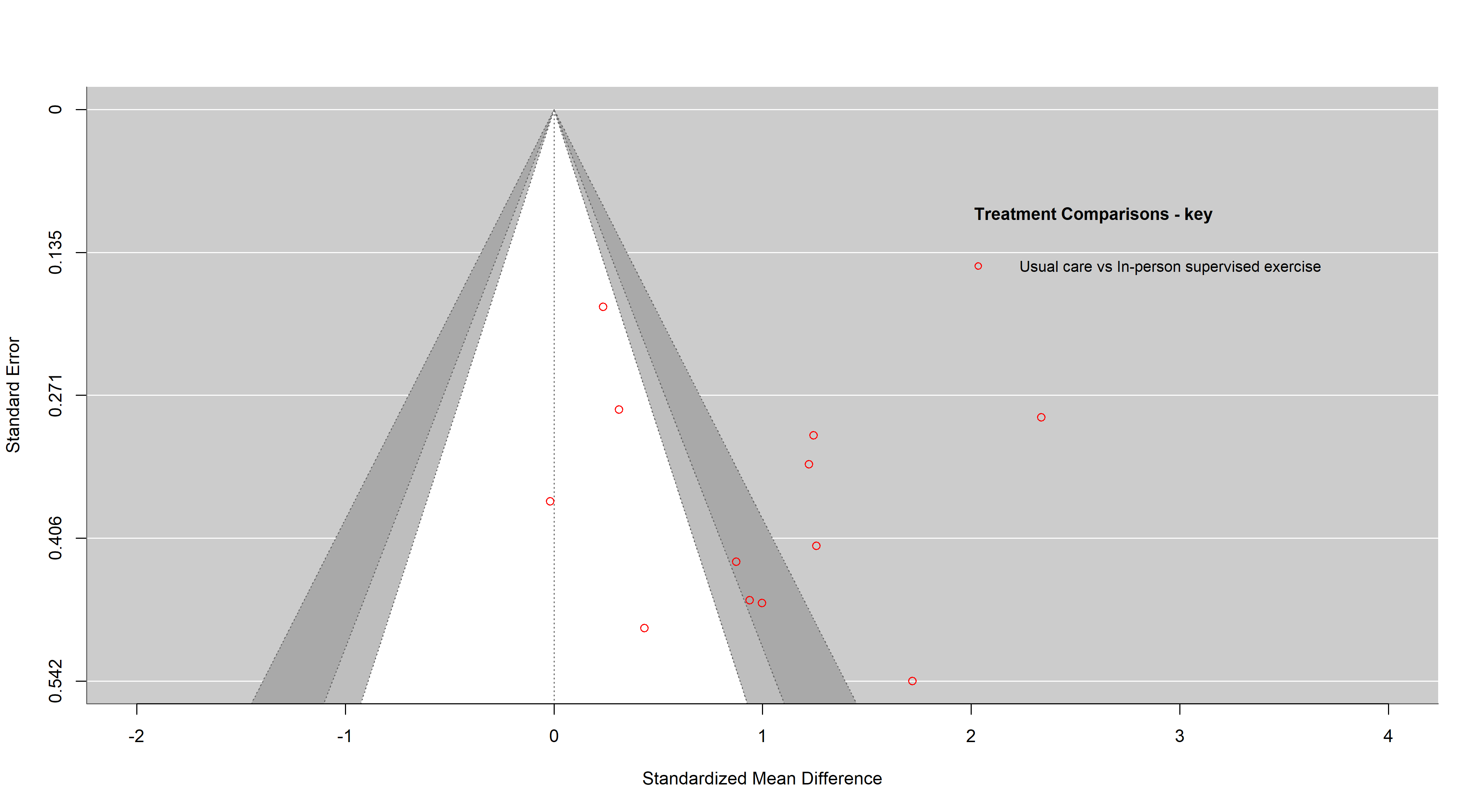


**Supplementary figure 83** – Contour adjusted funnel plot for the outcomes of breathlessness. Only comparisons for which there were at least 10 included studies as suggested by Sterne et al.[1]

## Characteristics of included studies

### **Supplementary table 15 -** Characteristics of interventions in included studies

| Study | Arm 1 components | Arm 2 components | Arm 3 components | Arm 4 components |
| --- | --- | --- | --- | --- |
|  |  |  |  |  |
|  |  |  |  |  |
| Abdelbasset, 2020[2] | Usual care | Supervised high intensity continuous cycling + arm cycling | NA | NA |
| Abedi Yekta, 2019[3] | Supervised moderate continuous walking + supervised moderate continuous cycling + unsupervised low intensity walking | Supervised moderate continuous walking + supervised moderate continuous cycling + unsupervised low intensity walking+ IMT | NA | NA |
| Acheche, 2020[4] | Low intensity interval cycling + upper and lower res | Low intensity interval cycling + NMES (50Hz) + upper and lower res | NA | NA |
| Ahnfeldt-Mollerup, 2015[5] | Supervised high intensity training, modality not specified +supervised strength training, not otherwise specified + unsupervised exercise, not otherwise specified + supervised education | Supervised high intensity training, modality not specified +supervised strength training, not otherwise specified + unsupervised exercise, not otherwise specified + supervised education + protein supplementation | NA | NA |
| Alcazar, 2019[6] | Usual care | supervised very high intensity interval cycling + upper and low moderate strength training | NA | NA |
| Aldhahir, 2021[7] | Supervised cycling and walking intensity not specified + upper and lower strength training + Education + Placebo | Supervised cycling and walking intensity not specified + upper and lower strength training + Education + high protein supplement | NA | NA |
| Alexander, 2012[8] | Supervised arm cycling + supervised low walking + supervised upper strength training (intensity "low" not otherwise specified) | Supervised arm cycling + supervised low walking + supervised upper strength training (intensity "low" not otherwise specified) + harmonica playing | NA | NA |
| Alsharaway, 2021[9] | Supervised education + Psychological counselling + Supervised walking, not otherwise specified + Supervised cycling, not otherwise specified + flexibility + callisthenics, not otherwise specified | Supervised education + Psychological counselling + Supervised walking, not otherwise specified + Supervised cycling, not otherwise specified + flexibility + callisthenics, not otherwise specified + Reflexology | NA | NA |
| Altenburg, 2014[10] | Supervised continuous walking and cycling, not otherwise specified + swimming + psychological (optional) or nutrition (optional) +supervised education | Supervised continuous walking and cycling, not otherwise specified + swimming + psychological (optional) or nutrition (optional) +supervised education +physical activity counselling | NA | NA |
| Ambrosino, 2008[11] | Supervised high continuous walking + supervised education + upper limb strength training, not otherwise specified + placebo | Supervised high continuous walking + supervised education + upper limb strength training, not otherwise specified + tiotropium | NA | NA |
| Aquino, 2016[12] | Moderate intensity continuous walking | Moderate intensity continuous walking + upper and lower strength training | NA | NA |
| Arbillaga-Etxarri, 2018[13] | Unsupervised education | Unsupervised high continuous walking + physical activity counselling + physical activity tracker | NA | NA |
| Armstrong, 2021[14] | Supervised upper and lower limb strength training, not otherwise specified + supervised moderate aerobic training, not otherwise specified + unsupervised moderate aerobic training, not otherwise specified | Supervised upper and lower limb strength training, not otherwise specified + supervised moderate aerobic training, not otherwise specified + unsupervised moderate aerobic training, not otherwise specified + CBT | NA | NA |
| Arnardottir, 2006[15] | Supervised high continuous cycling + flexibility + supervised upper, lower and trunk strength training, moderate + oxygen (optional) | Supervised very high interval cycling + flexibility + supervised upper, lower and trunk strength training, moderate + oxygen (optional) | NA | NA |
| Arnardottir, 2007[16] | Supervised very high interval cycling + supervised upper, lower and trunk moderate strength training + supervised callisthenics | Supervised upper, lower and trunk moderate strength training + supervised callisthenics | NA | NA |
| Arnedillo, 2020[17,18] | Usual care | Supervised high continuous cycling + supervised high continuous walking + upper and lower limb strength training supervised + breathing exercises + Flexibility | Supervised high continuous cycling + supervised high continuous walking + upper and lower limb strength training supervised + breathing exercises + Flexibility + nasal inspiratory restriction device | NA |
| Arslan, 2016[19] | Usual care | Unsupervised walking, not otherwise specified | NA | NA |
| Barakat, 2008[20] | Usual care | Supervised high intensity continuous cycling + supervised education + supervised callisthenics, not otherwise specified + dietary counselling | NA | NA |
| Beauchamp, 2013[21] | Supervised exercise, not otherwise specified + supervised breathing exercises + supervised education + psychological support | Supervised exercise, not otherwise specified + supervised breathing exercises + supervised education + psychological support + balance training | NA | NA |
| Beaumont, 2018[22] | Supervised continuous cycling, intensity not specified + Supervised continuous walking, intensity not specified + supervised upper and lower strength training + supervised education + aerobic gymnastics + smoking cessation + psychological and dietary advice | Supervised continuous cycling, intensity not specified + Supervised continuous walking, intensity not specified + supervised upper and lower strength training + supervised education + aerobic gymnastics + smoking cessation + psychological and dietary advice +IMT | NA | NA |
| Bendstrup, 1997[23] | Usual care | Supervised upper and lower resistance bands + balance training + skipping (optional) + occupational therapy + smoking cessation + supervised education | NA | NA |
| Benzo, 2021[24] | Usual care | Unsupervised low walking + motivational interviewing + Flexibility | NA | NA |
| Benzo, 2022[25] | Unsupervised education | Unsupervised continuous walking, intensity not specified + yoga + physical activity behaviour intervention + flexibility + balance + mindfulness | NA | NA |
| Bernard, 1999[26] | Supervised Very High Intensity Continuous Cycling + supervised breathing exercises + oxygen (optional) | Supervised Very High Intensity Continuous Cycling + supervised breathing exercises + oxygen (optional) + supervised upper and lower strength training, not otherwise specified | NA | NA |
| Berry, 2010[27] | Supervised moderate continuous walking + supervised resistance bands, muscle group not specified + supervised education | Supervised moderate continuous walking + supervised resistance bands, muscle group not specified + supervised education + unsupervised moderate exercise, not otherwise specified | NA | NA |
| Bhasin, 2012[28] | Supervised high continuous walking + supervised breathing exercises + chest physio | Supervised high continuous walking + supervised breathing exercises + chest physio + Breathing exercises | NA | NA |
| Bianchi, 2002[29] | Supervised moderate continuous cycling + supervised upper, lower and trunk strength training, not prescribed + supervised education | Supervised moderate continuous cycling + supervised upper, lower and trunk strength training, intensity not prescribed + supervised education + NIV during exercise | NA | NA |
| Bianco, 2019[30] | Supervised and unsupervised flexibility + supervised upper and lower strength training, intensity not prescribed + stairs + supervised and unsupervised callisthenics+ supervised education + IMT (Respironics, HR 70%max) | Supervised and unsupervised high continuous walking + stairs + arm cycling + supervised continuous cycling, not otherwise specified + supervised upper and lower strength training, intensity not prescribed + supervised education + IMT | NA | NA |
| Bjorgen, 2009[31] | Supervised high intensity interval cycling | One leg interval cycling | NA | NA |
| Bjorgen, 2009[32] | One leg interval cycling | One leg interval cycling + 100% oxygen | NA | NA |
| Blackstock, 2014[33] | Supervised low intensity walking + supervised low intensity cycling + supervised upper and lower limb strength training + oxygen + unsupervised low intensity walking | Supervised low intensity walking + supervised low intensity cycling + supervised upper and lower limb strength training + oxygen + unsupervised low intensity walking + supervised education | NA | NA |
| Bonnevie, 2018[34] | Supervised high continuous cycling + supervised moderate upper and lower strength training + education (optional) + nutrition (optional) + psychological (optional) | Supervised high continuous cycling + supervised moderate upper and lower strength training + education (optional) + nutrition (optional) + psychological (optional) +NMES (35Hz) | NA | NA |
| Borghi-Silva, 2006[35] | High intensity continuous walking + oxygen | Supervised high intensity continuous walking + NIV | NA | NA |
| Borghi-Silva, 2009[36] | Usual care | Supervised High Intensity Continuous Walking + supervised breathing exercises + oxygen (optional) | NA | NA |
| Borghi-Silva, 2010[37] | Supervised very high continuous walking + IMT + Placebo | Supervised very high continuous walking + IMT + L-carnitine | NA | NA |
| Bourbeau, 2020[38] | Supervised high intensity continuous cycling | Eccentric cycling | NA | NA |
| Bourjeily-Habr, 2002[39] | Sham NMES |  | NA | NA |
| Bourne, 2017[40] | Supervised callisthenics + supervised education | unsupervised callisthenics + unsupervised education | NA | NA |
| Bourne, 2022[41] | Usual care | Unsupervised education + Unsupervised very high continuous walking + unsupervised upper and lower strength training, not otherwise specified | NA | NA |
| Boxall, 2005[42] | Usual care | Unsupervised low walking + supervised education + unsupervised, upper strength training, not otherwise specified | NA | NA |
| Breyer, 2010[43] | Supervised education | Supervised Nordic walking + supervised education | NA | NA |
| Broekhuizen, 2005[44] | Supervised continuous cycling, intensity not specified + supervised continuous walking, not otherwise specified + swimming + education + psychology (optional) + OT (optional) + strength training, not otherwise specified + placebo | Supervised continuous cycling, intensity not specified + supervised continuous walking, not otherwise specified + swimming + education + psychology (optional) + OT (optional) + strength training, not otherwise specified + PUFA | NA | NA |
| Bronstad, 2013[45] | Supervised high intensity interval walking | Supervised moderate intensity continuous walking | NA | NA |
| Burtin, 2015[46] | Supervised high continuous walking + supervised high continuous cycling + education + OT (optional) + dietician (optional) + psychology (optional) +arm cycling +supervised upper and lower moderate strength training + stairs | Supervised high continuous walking + supervised high continuous cycling + education + OT (optional) + dietician (optional) + psychology (optional) +arm cycling +supervised upper and lower moderate strength training + stairs + physical activity behavioural intervention | NA | NA |
| Busch, 1988[47] | Usual care | Unsupervised moderate walking OR unsupervised stairs + unsupervised moderate strength training, not otherwise specified + flexibility + breathing exercises | NA | NA |
| Bustamante, 2010[48] | Usual care | Repetitive magnetic stimulation | NA | NA |
| Cambach, 1997[49] | Usual care | Supervised high continuous cycling + breathing retraining + supervised education + stair climbing + swimming (optional) + rowing | NA | NA |
| Cameron-Tucker, 2016[50] | Usual care | Unsupervised low walking + smoking cessation + nutrition + psychosocial wellbeing | NA | NA |
| Camillo, 2020[51] | Supervised high continuous cycling + Supervised low walking + upper and lower strength training + arm cycling + stair climbing + oxygen (optional) | Supervised high continuous cycling + Downhill walking + upper and lower strength training + arm cycling + stair climbing + oxygen (optional) | NA | NA |
| Carrieri-Kohlman, 1996[52] | Supervised low continuous walking | Supervised moderate continuous walking + coaching | NA | NA |
| Casaburi, 1991[53] | Supervised education + supervised high intensity continuous walking + upper limb strength training + oxygen (optional) + Placebo | Supervised education + supervised high intensity continuous walking + upper limb strength training + oxygen (optional) + Tiotropium | NA | NA |
| Casaburi, 2005[54] | Supervised moderate continuous cycling + supervised education | Supervised very high continuous cycling + supervised education | NA | NA |
| Casey, 2013[55] | usual care | Supervised and unsupervised circuit training + unsupervised high continuous walking + supervised education + breathing exercises | NA | NA |
| Chan, 2010[56] | Usual care | Unsupervised low intensity continuous walking + breathing exercises | Supervised and unsupervised tai chi | NA |
| Chaplin, 2017[57] | Supervised continuous moderate walking + unsupervised moderate continuous walking + supervised upper and lower strength training (Borg 13-15) + Supervised moderate continuous cycling + supervised education | Unsupervised very high continuous walking + unsupervised upper and lower strength training + unsupervised education | NA | NA |
| Charususin, 2021[58] | Breathing control + Callisthenics (unsupervised) + unsupervised moderate continuous cycling + unsupervised upper strength training, not otherwise specified + unsupervised moderate continuous walking | Breathing control + water Callisthenics (unsupervised), intensity not stated + unsupervised moderate continuous cycling in water + unsupervised upper strength training in water, not otherwise specified + unsupervised moderate continuous walking in water | NA | NA |
| Chen, 2015[59] | Usual care | Daoyin exercise (supervised and unsupervised) | NA | NA |
| Chen, 2015[60] | Supervised education | Supervised education + unsupervised resistance bands (lower limbs) | NA | NA |
| Chen, 2018[61] | Supervised high continuous cycling | Supervised moderate upper and lower strength training | Supervised high continuous cycling + supervised moderate upper and lower strength training | NA |
| Clark, 1996[62] | Usual care | supervised callisthenics | NA | NA |
| Clark, 2000[63] | Usual care | Supervised moderate upper and lower moderate strength training | NA | NA |
| Collins, 2008[64] | Supervised moderate intensity continuous cycling + supervised low upper strength training + supervised moderate continuous walking | Supervised moderate intensity continuous cycling + Ventilation feedback training + supervised moderate continuous walking + supervised upper limb strength training, low | NA | NA |
| Collins, 2014[65] | Supervised moderate intensity continuous walking + supervised upper limb strength training, not otherwise specified + supplemental oxygen (regardless of sats) | Supervised moderate intensity continuous walking + supervised upper limb strength training, not otherwise specified + supplemental oxygen + breathing retraining | Supervised moderate intensity continuous walking + supervised upper limb strength training + Heliox | NA |
| Collins, 2019[66] | Supervised high intensity continuous walking | Supervised high intensity continuous walking + metronome paced breathing | NA | NA |
| Constantin, 2013[67] | Supervised moderate lower strength training + placebo | Supervised lower moderate strength training + protein/carbohydrate supplement | NA | NA |
| Coppoolse, 1999[68] | Supervised high continuous cycling + supervised callisthenics + supervised education | Supervised very high interval cycling + supervised callisthenics + supervised education | NA | NA |
| Costi, 2009[69] | Supervised moderate continuous cycling + supervised callisthenics + supervised upper and lower strength training, not otherwise specified | Supervised moderate continuous cycling + supervised callisthenics + supervised strength training, not otherwise specified + upper limb resistance | NA | NA |
| Covey, 2012[70] | Supervised chair exercises | Supervised moderate lower limb resistance | NA | NA |
| Covey, 2014[71] | Supervised education + supervised chair exercises | Supervised moderate upper strength training + Self efficacy training | Supervised upper moderate strength training + supervised education | NA |
| Cox, 2022[72] | Supervised low continuous walking + supervised moderate continuous cycling + supervised upper and lower strength training not prescribed + unsupervised low continuous walking + supervised education | supervised moderate continuous cycling + supervised upper and lower strength training not prescribed + unsupervised low continuous walking + supervised education | NA | NA |
| Creutzberg, 2003[73] | Supervised continuous cycling, intensity not specified + supervised continuous walking, intensity not specified + swimming + Placebo | Supervised continuous cycling, intensity not specified + supervised continuous walking, intensity not specified + swimming + Anabolic steroid | NA | NA |
| Cruz, 2016[74] | Supervised continuous walking and cycling, not otherwise specified + swimming + psychological +supervised education + strength training, not otherwise specified + balance | Supervised continuous walking and cycling, not otherwise specified + swimming + psychological +supervised education + strength training, not otherwise specified + balance + physical activity behavioural intervention | NA | NA |
| Curtis, 2016[75] | Education + moderate intensity continuous walking + moderate intensity continuous cycling (supervised) + upper + lower strength training + placebo | Education + moderate intensity continuous walking + moderate intensity continuous cycling (supervised) + upper + lower strength training + ACE inhibitor | NA | NA |
| de Bisschop, 2021[76] | Supervised interval or continuous cycling + low intensity walking + strength training (not specified) + education + breathing retraining + Placebo | Supervised interval or continuous cycling + low intensity walking + strength training (not specified) + education + breathing retraining + BCAA supplement | NA | NA |
| de Blok, 2006[77] | Supervised exercise, not otherwise specified + dietary + supervised education | Supervised exercise, not otherwise specified + dietary + supervised education + physical activity counselling | NA | NA |
| De Godoy, 2005[78] | Supervised moderate continuous walking + Education + breathing exercises + supervised strength training, not otherwise specified + Flexibility | Supervised moderate continuous walking + Education + breathing exercises + supervised strength training, not otherwise specified +psychotherapy + Flexibility | NA | NA |
| de Lima, 2022[79] | Supervised high continuous walking + supervised moderate upper and lower strength training | Supervised high continuous walking + supervised moderate upper and lower strength training + supervised high callisthenics | NA | NA |
| de Roos, 2018[80] | usual care | supervised high continuous cycling + supervised high continuous walking + strength training, not otherwise specified + supervised education + unsupervised low intensity walking | NA | NA |
| De Sousa Pinto, 2014[81] | Usual care | Unsupervised moderate continuous walking + stair climbing + upper and lower limb strength training, not otherwise specified + unsupervised moderate intensity continuous cycling (optional) | NA | NA |
| Deacon, 2008[82] | lower and upper limb and trunk resistance, not otherwise specified + supervised and unsupervised high intensity continuous walking + Placebo | lower and upper limb and trunk resistance, not otherwise specified + supervised and unsupervised high intensity continuous walking + Placebo + Creatine | NA | NA |
| Deering, 2011[83] | Usual care | Supervised continuous walking, intensity not specified + IMT + supervised education | Supervised continuous walking, intensity not specified + IMT + supervised education + Acupuncture | NA |
| Dekhuijzen, 1990[84] | Supervised continuous cycling, not otherwise specified + supervised continuous walking, not otherwise specified + trunk strength training + breathing exercises + supervised education | Supervised continuous cycling, not otherwise specified + supervised continuous walking, not otherwise specified + trunk strength training + breathing exercises + supervised education + IMT | NA | NA |
| Deniz, 2023[85] | Supervised moderate continuous walking + supervised moderate continuous cycling + breathing supervised + supervised upper and lower strength training, not otherwise specified + unsupervised aerobic exercise, not otherwise specified | Supervised moderate continuous walking + supervised moderate continuous cycling + breathing supervised + supervised upper and lower strength training, not otherwise specified + oxygen + unsupervised aerobic training, not otherwise specified | Supervised moderate continuous walking + supervised moderate continuous cycling + breathing supervised + supervised upper and lower strength training, not otherwise specified + oxygen + NIV + unsupervised aerobic not otherwise specified | NA |
| Dodia, 2012[86] | Supervised high continuous walking | Supervised moderate upper strength training | NA | NA |
| Dolmage, 2008[87] | High intensity continuous cycling + breathing training (supervised) + stretching exercises + low continuous walking + upper and lower strength training not prescribed + psychological support + nutritional counselling + OT + education | High intensity continuous cycling (1 leg) + breathing training (supervised) + stretching exercises + low continuous walking + upper and lower strength training not prescribed + psychological support + nutritional counselling + OT + education | NA | NA |
| Donesky-Cuenco, 2009[88] | Usual care | Supervised and unsupervised yoga | NA | NA |
| Dong, 2021[89] | Supervised moderate intensity continuous cycling | Qigong | NA | NA |
| Dourado, 2009[90] | supervised strength training, not otherwise specified | Supervised low walking + supervised upper and lower strength training | NA | NA |
| Du, 2013[91] | Usual care | Unsupervised low walking + breathing exercises | Supervised and unsupervised tai chi | NA |
| Duan, 2022[92] | Usual care | Supervised high continuous cycling | NA | NA |
| Duruturk, 2016[93] | Education | Supervised moderate intensity continuous cycling + Education | Callisthenics + Education | NA |
| Effing, 2011[94] | Supervised education | Supervised education + supervised high continuous walking OR supervised high interval walking + supervised high continuous cycling OR supervised high interval cycling + stairs + supervised moderate upper and lower strength training + unsupervised high continuous walking OR unsupervised high interval walking OR unsupervised high continuous cycling OR unsupervised high interval cycling + unsupervised upper and lower strength training band | NA | NA |
| Elci, 2008[95] | Usual care | Unsupervised low walking + unsupervised upper, lower and trunk strength training, not otherwise specified + nutritional and psychological counselling + supervised education | NA | NA |
| Elliott, 2004[96] | Supervised callisthenics + supervised continuous walking and cycling, not otherwise specified + supervised education | Supervised callisthenics + supervised education | NA | NA |
| Elmorshidy, 2023[97] | Supervised moderate continuous walking + supervised continuous cycling, not otherwise specified (optional) + supervised upper strength training, not otherwise specified + NMES (optional) of quads and diaphragm + IMT + breathing exercises + chest physio + supervised education | Supervised moderate continuous walking + supervised continuous cycling, not otherwise specified (optional) + supervised upper strength training, not otherwise specified + NMES (optional) of quads and diaphragm + IMT + breathing exercises + chest physio + supervised education + oxygen | Supervised moderate continuous walking + supervised continuous cycling, not otherwise specified (optional) + supervised upper strength training, not otherwise specified + NMES (optional) of quads and diaphragm + IMT + breathing exercises + chest physio + supervised education + oxygen +NIV | NA |
| Elmorsi, 2016[98] | Supervised walking not otherwise specified, supervised upper and lower limb res | Supervised walking not otherwise specified, supervised upper and lower limb strength training + IMT | NA | NA |
| Emery, 1998[99] | Usual care | Supervised education | Supervised continuous cycling, not otherwise specified + supervised continuous walking, not otherwise specified, + arm cycling + supervised strength training, not otherwise specified + supervised education + psychology | NA |
| Emtner, 2003[100] | Supervised high continuous cycling + air + supervised education | Supervised high continuous cycling + oxygen + supervised education | NA | NA |
| Engel, 2016[101] | Supervised walking, not otherwise specified + supervised cyling, not otherwise specified + callisthenics, not otherwise specified + supervised strength training, not otherwise specified + rowing, not otherwise specified | Supervised walking, not otherwise specified + supervised cycling, not otherwise specified + callisthenics, not otherwise specified + supervised strength training, not otherwise specified + rowing, not otherwise specified + Soft manipulation (sham osteopathy treatment) | Supervised walking, not otherwise specified + supervised cycling, not otherwise specified + callisthenics, not otherwise specified + supervised strength training, not otherwise specified + rowing, not otherwise specified + Soft manipulation (sham osteopathy treatment) + Osteopathic manipulation | NA |
| Engstrom, 1999[102] | Usual care | Supervised very high cycling + supervised upper limb strength training, not otherwise specified + unsupervised strength training, not otherwise specified + unsupervised flexibility + breathing techniques + OT + dietician + education (supervised) | NA | NA |
| Eves, 2009[103] | Supervised moderate continuous cycling + supervised education + supervised strength training, not otherwise specified + walking, not otherwise specified + air | Supervised moderate continuous cycling + supervised education + supervised strength training, not otherwise specified + walking, not otherwise specified + Helium-hyperoxia | NA | NA |
| Faager, 2006[104] | High intensity continuous cycling + upper limb strength training + upper limb strength training + Education + Placebo | High intensity continuous cycling + upper limb strength training + upper limb strength training + Education + Creatine | NA | NA |
| Fanfa Bordin, 2020[105] | Supervised high continuous cycling + supervised moderate upper and lower strength training | Supervised high continuous cycling + supervised moderate upper and lower strength training + IMT | NA | NA |
| Farias, 2014[106] | Education | Supervised moderate intensity continuous walking + Education + unsupervised moderate intensity continuous walking | NA | NA |
| Farver-Vestergaard, 2018[107] | Supervised exercise, not otherwise specified + supervised education | Supervised exercise, not otherwise specified + supervised education + mindfulness | NA | NA |
| Felcar, 2018[108,109] | supervised low intensity continuous walking + supervised high continuous cycling + supervised education + supervised upper and lower strength training | Low intensity continuous walking in water, supervised high intensity continuous cycling in water + upper and lower strength training in water + supervised education | NA | NA |
| Ferreira, 2013[110] | Supervised very high continuous cycling + air | Supervised very high continuous cycling + oxygen | NA | NA |
| Fichter, 1999[111] | Supervised education + supervised upper and lower limb strength training, intensity not prescribed + supervised walking, not otherwise specified (optional) + supervised stepping, not otherwise specified (optional) + supervised cycling, not otherwise specified (optional) | Supervised education + supervised upper and lower limb strength training, intensity not prescribed + supervised walking, not otherwise specified (optional) + supervised stepping, not otherwise specified (optional) + supervised cycling, not otherwise specified (optional) +d-cycloserine | NA | NA |
| Finnegan, 2023[112] | Supervised education | Supervised education + supervised callisthenics + supervised cycling, not otherwise specified (optional) + supervised walking, not otherwise specified (optional) + unsupervised continuous walking, not otherwise specified + dietary advice + OT + psychological input | NA | NA |
| Finnerty, 2001[113] | Supervised upper and lower strength training, intensity not specified | Supervised upper and lower resistance bands | Supervised upper and lower resistance tubes | NA |
| Freire, 2022[114] | Supervised high intensity continuous cycling + callisthenics + Education + unsupervised home exercise + supervised flexibility + Placebo | Supervised high intensity continuous cycling + callisthenics + Education + unsupervised home exercise + Creatine + Flexibility | NA | NA |
| Fuld, 2005[115] | Supervised high continuous walking + supervised high continuous cycling + stairs + supervised education | Supervised high continuous walking + supervised high continuous cycling + stairs + supervised education + supervised upper strength training, moderate | NA | NA |
| Gadesha, 2015[116] | Usual care | Water callisthenics, high intensity + oxygen (optional) | NA | NA |
| Gallo-Silva, 2019[117] | Supervised high interval cycling | Supervised moderate interval walking | NA | NA |
| Gamper, 2019[118] | supervised education + Supervised upper strength training, not prescribed, lower strength training, not prescribed. + supervised high continuous walking + supervised low cycling + unsupervised exercise, not otherwise specified | supervised education + Supervised upper strength training, not prescribed, lower strength training, not prescribed. + supervised high continuous walking + supervised low cycling + unsupervised exercise, not otherwise specified +NIV | NA | NA |
| Garrod, 2000[119] | Usual care | Supervised moderate continuous walking + supervised strength training, not otherwise specified | NA | NA |
| Gayle, 1988[120] | Supervised aerobic exercise, not otherwise specified + supervised strength training, not otherwise specified + whole body vibration + supervised education + breathing retraining + smoking cessation (optional) + chest physio (optional) + IMT (optional) + NMES (optional) + saline (optional) + psychological intervention (optional) + nutrition (optional) + OT (optional) | Supervised aerobic exercise, not otherwise specified + supervised strength training, not otherwise specified + whole body vibration + supervised education + breathing retraining + smoking cessation (optional) + chest physio (optional) + IMT (optional) + NMES (optional) + saline (optional) + psychological intervention (optional) + nutrition (optional) + OT (optional) + pedometer behavioural intervention | NA | NA |
| Geidl, 2021[121] | Supervised high continuous cycling + supervised low upper and lower strength training | Supervised high continuous cycling + moderate upper and lower strength training | NA | NA |
| Gianjoppe-Santos, 2021[122] | Moderate intensity continuous walking (supervised) | Supervised very high continuous cycling | NA | NA |
| Gimenez, 2000[123] | Supervised high intensity con cycling + Supervised strength training, moderate (limb unclear) + Breathing retraining + education + dietary advice + psychological support | Supervised high intensity con cycling + Supervised strength training (limb unclear) + Breathing retraining + education + dietary advice + psychological support + supervised whole-body vibration | NA | NA |
| Gloeckl, 2012[124] | Supervised high continuous cycling + supervised upper and lower moderate strength training + supervised education + supervised breathing retraining + chest physio + Supervised callisthenics + balance + optional (OT, nutrition, psychology) | Supervised high continuous cycling + supervised upper and lower moderate strength training + supervised education + supervised breathing retraining + chest physio + Supervised callisthenics + WBVT + optional (OT, nutrition, psychology) | NA | NA |
| Gloeckl, 2017[125] | Supervised high continuous cycling + supervised upper and lower moderate strength training + chest physio + supervised education + nutrition + psychological counselling | Supervised high continuous cycling + supervised upper and lower moderate strength training + chest physio + supervised education + nutrition + psychological counselling + vibration | NA | NA |
| Gloeckl, 2021[126] | Usual care | Supervised moderate continuous cycling + Unsupervised moderate continuous walking + upper and lowe limb strength training, not otherwise specified + Education + supervised very high intensity interval cycling + stairs + Flexibility | NA | NA |
| Gohl, 2006[127] | Supervised high continuous walking + supervised high continuous cycling + supervised high strength training, limb not specified + dietary counselling + smoking cessation + supervised education | Supervised high continuous walking + supervised high continuous cycling + supervised high strength training, limb not specified + dietary counselling + smoking cessation + supervised education | NA | NA |
| Gouzi, 2019[128] | Usual care | Supervised very high continuous walking + supervised very high continuous cycling + stairs + supervised upper strength training, intensity not prescribed + unsupervised aerobic training, not otherwise specified + supervised education + psychological support + nutritional support | NA | NA |
| Griffiths, 2000[129] | Supervised high continuous cycling + supervised education + IMT + upper limb strength training (supervised), not prescribed | Unsupervised continuous walking, intensity not specified + supervised education + IMT + upper limb strength training, not prescribed (unsupervised) +stairs | NA | NA |
| Guell, 2000[130] | Usual care | Supervised moderate continuous cycling + supervised education + supervised breathing retraining + chest physio (optional) | NA | NA |
| Guell, 2006[131] | Usual care | Supervised moderate continuous cycling + breathing retraining + unsupervised low walking + chest physio (optional) + supervised education + unsupervised cycling, not otherwise specified (optional) | NA | NA |
| Guell, 2008[132] | Supervised education +supervised high continuous cycling or walking (optional) + Supervised high interval cycling or walking (optional) + supervised upper and lower strength training, low + smoking cessation (optional) + dietary (optional) + supervised breathing exercises | Education + supervised upper and lower moderate strength training | NA | NA |
| Hansen, 2020[133,134] | IMT, not otherwise specified + unsupervised lower strength training, not otherwise specified + unsupervised high continuous walking | IMT, not otherwise specified + unsupervised lower strength training, not otherwise specified + unsupervised high continuous walking + SABA | NA | NA |
| Hasegawa, 2012[135] | Supervised high continuous cycling | Supervised high continuous cycling +NIV | NA | NA |
| Haugen, 2007[136] | Supervised low intensity cycling + education + supervised interval walking, not otherwise specified + supervised strength training, not otherwise specified | Supervised moderate intensity continuous cycling + education | Supervised high intensity continuous cycling + education | NA |
| Hawkins, 2002[137] | Usual care | Unsupervised high continuous walking | NA | NA |
| He, 2019[138] | Unsupervised education + unsupervised low intensity walking | unsupervised high intensity continuous walking + unsupervised education + music | NA | NA |
| Hernandez, 2000 | Usual care | Supervised lower limb strength training, high intensity | NA | NA |
| Ho, 2012[140] | Supervised high continuous walking OR supervised high continuous cycling + stairs + unsupervised exercise, not otherwise specified + sham training (pegboard test) | Supervised high continuous walking OR supervised high continuous cycling + stairs + unsupervised exercise, not otherwise specified + supervised upper moderate resistance | NA | NA |
| Hoff, 2007[141] | Supervised high continuous walking + supervised cycling, not otherwise specified + stair climbing + supervised upper strength training + supervised education | Supervised education + unsupervised high continuous walking + unsupervised upper and lower strength training | NA | NA |
| Holland, 2004[142] | Supervised high intensity continuous walking + Education + unsupervised high intensity continuous walking + upper and lower limb strength training, moderate (supervised) + upper and lower limb strength training, moderate (unsupervised) | Unsupervised high intensity continuous walking + unsupervised upper and lower limb strength training, moderate + unsupervised education | NA | NA |
| Holland, 2017[143–145] | Supervised continuous walking, not otherwise specified + unsupervised continuous walking, not otherwise specified + swimming + supervised education + psychosocial support | Supervised continuous walking, not otherwise specified + unsupervised continuous walking, not otherwise specified + swimming + supervised education + psychosocial support | NA | NA |
| Horton, 2018[146] | Supervised high intensity continuous walking or cycling | Supervised upper and lower strength training, low | NA | NA |
| Iepsen, 2016[147] | Moderate intensity interval cycling | Eccentric cycling | NA | NA |
| Inostroza, 2022[148] | Supervised low walking + supervised upper and lower strength training (moderate) + supervised breathing + supervised education | Supervised low walking + supervised lower strength training (moderate) + supervised breathing + supervised education + supervised flexibility | NA | NA |
| Janaudis-Ferreira, 2011[149] | Supervised education | Unsupervised low walking + breathing exercises + IMT + supervised education + upper limb resistance bands | NA | NA |
| Jang, 2006[150] | Supervised education + smoking cessation + supervised moderate continuous walking + unsupervised moderate continuous walking | Supervised education + smoking cessation + supervised moderate continuous walking + Heliox + unsupervised moderate continuous walking | Supervised education + smoking cessation + supervised moderate continuous walking + NIV +unsupervised moderate continuous walking | NA |
| Johnson, 2002[151] | Supervised high continuous cycling + supervised upper limb strength training (intensity not prescribed) + unsupervised callisthenics | Unsupervised high continuous walking + unsupervised upper limb strength training (intensity not prescribed) + unsupervised callisthenics | NA | NA |
| Jolly, 2014[152] | Usual care | Tai Chi (supervised and unsupervised) | NA | NA |
| Kantatong, 2020[153] | Supervised moderate interval cycling + upper and lower strength training, moderate + oxygen (optional) | Supervised moderate interval cycling + upper and lower strength training, moderate + arm cycling + oxygen (optional) | NA | NA |
| Karagiannis, 2021[154] | Usual care | Supervised moderate continuous walking + Education + upper and lower limb strength training, not otherwise specified + breathing exercises + respiratory physio (optional) | NA | NA |
| Karapolat, 2007[155] | Supervised education + IMT + unsupervised moderate continuous walking, + callisthenics + breathing exercises | Supervised education + IMT + unsupervised moderate continuous walking + callisthenics + breathing exercises + physical activity counselling/pedometer | NA | NA |
| Kawagoshi, 2015[156] | Unsupervised breathing techniques + unsupervised aerobic training, not otherwise specified | Unsupervised breathing techniques + unsupervised aerobic training, not otherwise specified + creative dance | NA | NA |
| Kaya, 2023[157] | Usual care | Supervised education + chest physio + breathing retraining + upper limb strength training, not otherwise specified + supervised continuous cycling not otherwise specified | NA | NA |
| Kayahan, 2006[158] | Supervised circuit training + diaphragm exercises | Unsupervised circuit training + unsupervised diaphragm exercises | NA | NA |
| Kilic, 2021[159] | Low continuous walking + supervised upper and lower moderate strength training + supervised education + breathing retraining + chest physio + psychosocial support + supervised moderate continuous cycling + oxygen (optional) | supervised upper and lower low strength training + supervised education + breathing retraining + chest physio + psychosocial support + oxygen (optional) + supervised high interval cycling + periodisation | NA | NA |
| Klijn, 2013[160] | Supervised high continuous cycling + supervised education + supervised moderate upper and lower strength training | Supervised high continuous cycling + supervised education + supervised moderate upper strength training + supervised low lower strength training + BFR | NA | NA |
| Kohlbrenner, 2023[161] | Usual care | Supervised high lower strength training | NA | NA |
| Kongsgaard, 2004[162] | Supervised continuous cycling, not otherwise specified + supervised strength training, not otherwise specified + supervised callisthenics + breathing exercises + supervised education | Supervised continuous cycling, not otherwise specified + supervised strength training, not otherwise specified + supervised callisthenics + breathing exercises + supervised education + underground | NA | NA |
| Kostrzon, 2019[163] | Usual care | Unsupervised high continuous walking + unsupervised strength training, not otherwise specified | NA | NA |
| Kwon, 2018[164] | Usual care | Unsupervised high continuous walking + unsupervised upper and lower strength training, not otherwise specified | NA | NA |
| Lahham, 2020[165] | Usual care | Arm cycling + supervised upper limb strength training, not otherwise specified | Supervised continuous walking, not otherwise specified | 0 |
| Lake, 1990[166] | Education | Supervised and unsupervised moderate intensity interval cycling | Supervised and unsupervised moderate interval cycling +IMT | NA |
| Larson, 1999[167] | Supervised Very High Intensity Continuous Cycling +Education + supervised upper and lower limb strength training + placebo | Supervised Very High Intensity Continuous Cycling +Education + supervised upper and lower limb strength training + whey protein | NA | NA |
| Laviolette, 2010[168] | Usual care | Walking or cycling, not otherwise specified + upper limb strength, not otherwise specified + flexibility | NA | NA |
| Lee, 2022[169] | Supervised high continuous walking | Supervised high continuous cycling | NA | NA |
| Leung, 2010[170] | Usual care | Supervised and unsupervised tai chi | NA | NA |
| Leung, 2013[171] | Education, not otherwise specified | Liuzijue exercise + Education, not otherwise specified | NA | NA |
| Li, 2018[172] | Supervised continuous walking, high + supervised callisthenics, high + supervised education + unsupervised exercise, not otherwise specified | Supervised continuous walking, high + supervised callisthenics, high + supervised education + unsupervised exercise, not otherwise specified | NA | NA |
| Liddell, 2010[173] | Usual care | Unsupervised continuous walking, not otherwise specified + supervised education + unsupervised breathing exercises | NA | NA |
| Lin, 2021[174] | Breathing exercises + Supervised high intensity continuous walking + chest physio | Breathing exercises + Supervised high intensity continuous walking + chest physio + Flexibility | NA | NA |
| Liu, 2008[175] | Usual care | Supervised Liuzijue | Water based Liuzijue | NA |
| Liu, 2021[176] | High intensity continuous walking (unsupervised) | High intensity continuous walking (unsupervised) + music | NA | NA |
| Liu, 2021[177,178] | Usual care | Supervised education + unsupervised aerobic not otherwise specified + psychology + nutrition | NA | NA |
| Liu, 2021[179] | Supervised education | Supervised continuous cycling, intensity not prescribed + supervised education | Tai chi + supervised education | Tai chi + supervised education + supervised continuous cycling, intensity not prescribed |
| Liu, 2023[180] | Usual care | Supervised very high intensity interval cycling + supervised upper + lower limb strength training + breathing retraining + dietary advice + education | NA | NA |
| Louvaris, 2016[181] | Usual care | Supervised breathing exercises + supervised upper limb strength training, not otherwise specified + stairs | NA | NA |
| Lum, 2007[182] | Supervised education | Supervised low intensity callisthenics + supervised breathing + chest physio + supervised education + unsupervised low callisthenics + unsupervised breathing | NA | NA |
| Ma, 2022[183] | Sham NMES | HF NMES | NA | NA |
| Maddocks, 2016[184] | Supervised moderate continuous walking + callisthenics + Education | Supervised moderate continuous walking + callisthenics + Education + Hyperpnea training | NA | NA |
| Mador, 2004[185] | Supervised moderate continuous cycling + supervised moderate continuous walking + supervised education | Supervised education + supervised moderate continuous walking + supervised moderate continuous cycling + supervised upper and lower strength training | NA | NA |
| Mador, 2005[186] | Supervised moderate continuous cycling +supervised high continuous walking + supervised education + supervised callisthenics | Supervised high interval cycling +supervised very high interval walking + supervised education + supervised callisthenics | NA | NA |
| Mador, 2009[187] | Usual care | Supervised high continuous walking + supervised upper and lower strength training, not otherwise specified + supervised education + unsupervised aerobic training, not otherwise specified | NA | NA |
| Maglakelidze, 2022[188] | Usual care | Supervised continuous cycling, not otherwise specified | Supervised continuous cycling, not otherwise specified +IMT | NA |
| Majewska-Pulsakowska, 2016[189] | Supervised very high continuous cycling + oxygen (optional) + supervised strength training, not otherwise specified + supervised education | Unsupervised high continuous cycling + oxygen (optional) + unsupervised strength training, not otherwise specified + supervised education | NA | NA |
| Maltais, 2008[190] | Supervised high continuous cycling OR supervised high continuous walking OR arm cycling + rowing | Supervised high continuous cycling OR supervised high continuous walking OR arm cycling + rowing + supervised trunk strength training + supervised lower resistance bands | NA | NA |
| Mao, 2018[191] | Supervised low intensity walking + supervised upper and lower strength training + balance training + education + psychological support | Supervised low intensity walking + supervised upper and lower strength training + balance training + education + psychological support + family involvement | NA | NA |
| Marques, 2014[192] | Supervised very high intensity continuous cycling + upper limb strength training, not otherwise specified + supervised low intensity walking + Placebo | Supervised very high intensity continuous cycling + upper limb strength training, not otherwise specified + supervised low intensity walking+ irbesartan | NA | NA |
| Marquis, 2008[193] | Supervised high continuous walking + respiratory physio +Flexibility | Supervised upper limb strength training + respiratory physio + Flexibility | NA | NA |
| Marrara, 2008[194] | Supervised high continuous walking + oxygen (optional) | Supervised high continuous walking + oxygen (optional) +NIV | NA | NA |
| Marrara, 2012[195] | Chest physio + breathing exercises + flexibility | Chest physio + breathing exercises + flexibility + supervised high intensity continuous walking | NA | NA |
| Marrara, 2018[196] | Supervised low walking + supervised low cycling + stairs + arm cycling + supervised strength training, not otherwise specified | Supervised low walking + supervised low cycling + stairs + arm cycling + supervised strength training, not otherwise specified + chest wall vibration | Supervised low walking + supervised low cycling + stairs + arm cycling + supervised strength training, not otherwise specified + PEP (Acapella) | NA |
| McCarroll, 2005[197] | Supervised moderate interval walking + low cycling | Supervised callisthenics + balance training | NA | NA |
| McFarland, 2012[198] | Supervised high intensity continuous cycling and high intensity continuous walking + supervised lower limb strength training, moderate | Supervised high intensity continuous cycling and high intensity continuous walking + supervised lower limb strength training, moderate + arm cycling | Supervised high intensity continuous cycling and high intensity continuous walking + supervised lower limb strength training, moderate + supervised upper limb strength training, moderate | Supervised high intensity continuous cycling and high intensity continuous walking + supervised lower limb strength training, moderate + supervised upper limb strength training, moderate + arm cycling |
| McKeough, 2012[199] | Low intensity interval cycling + upper and lower strength training (low) | Low intensity interval cycling + NMES HF | NA | NA |
| Mekki, 2019[200] | Supervised moderate continuous cycling + supervised moderate continuous walking + arm cycling + stairs + supervised upper strength training, not otherwise specified + oxygen (optional) + chest physio + breathing exercises + unsupervised aerobic exercise not otherwise specified | Supervised moderate continuous cycling + supervised moderate continuous walking + arm cycling + stairs + supervised upper strength training, not otherwise specified + oxygen (optional) + chest physio + breathing exercises + unsupervised aerobic exercise not otherwise specified +physical activity intervention | Supervised moderate continuous cycling + supervised moderate continuous walking + arm cycling + stairs + supervised upper strength training, not otherwise specified + oxygen (optional) + chest physio + breathing exercises + unsupervised aerobic exercise not otherwise specified +supervised education | NA |
| Miglioire, 2003[201] | Usual care | Unsupervised education + Unsupervised very high continuous walking + unsupervised upper and lower strength training, not otherwise specified | NA | NA |
| Mitchell, 2014[202] | Unsupervised exercise, not otherwise specified + breathing exercises + supervised education | Unsupervised exercise, not otherwise specified + breathing exercises + supervised education + balance training | NA | NA |
| Mkacher, 2015[203,204] | Supervised moderate intensity continuous walking | Downhill walking | NA | NA |
| Moezy, 2018[205] | Usual care | supervised low intensity continuous walking OR supervised low continuous cycling + supervised low callisthenics + supervised education | NA | NA |
| Mohamed, 2022[206] | Unsupervised education | Unsupervised education + unsupervised callisthenics, high | NA | NA |
| Moore, 2009[207] | Usual care | Supervised moderate continuous walking + upper and lower limb strength training (supervised) + breathing exercises | Supervised moderate continuous walking + supervised circuit training | NA |
| Nakamura, 2008[208] | Usual care | Supervised moderate continuous walking + breathing exercises | NA | NA |
| Nalbant, 2011[209] | Supervised education + counselling + unsupervised home exercise, not specified | Supervised education + counselling+ supervised cycling not otherwise specified, supervised walking not otherwise specified +Flexibility | NA | NA |
| Naseer, 2017[210] | Supervised High intensity continuous cycling + breathing techniques + dietary advice + oxygen | Supervised very High intensity interval cycling + breathing techniques + dietary advice + oxygen | NA | NA |
| Nasis, 2009[211] | Usual care | NMES 50Hz | NA | NA |
| Neder, 2002[212] | Supervised high intensity interval training + Supervised moderate upper and lower strength training + air | Supervised high intensity interval training + Supervised moderate upper and lower strength training + oxygen | NA | NA |
| Neunhauserer, 2023[213] | Supervised continuous walking and cycling, intensity not specified + unsupervised walking, not otherwise specified + unsupervised resistance bands + Education | Supervised continuous walking and cycling, intensity not specified + unsupervised walking, not otherwise specified + unsupervised Thera-Band + Education + Tai chi (supervised and unsupervised) | NA | NA |
| Ng, 2014[214] | Supervised education | Unsupervised moderate continuous walking (optional) + unsupervised continuous moderate cycling (optional) + unsupervised swimming (optional) + unsupervised upper limb strength training (intensity not clear) + supervised education | Unsupervised moderate continuous walking (optional) + unsupervised continuous moderate cycling (optional) + unsupervised swimming (optional) + unsupervised upper limb strength training (intensity not clear) + unsupervised education | NA |
| Nguyen, 2008[215] | Unsupervised moderate continuous walking (optional) + unsupervised continuous moderate cycling (optional) + unsupervised swimming (optional) + unsupervised upper limb strength training (intensity not clear) + supervised education | Unsupervised moderate continuous walking (optional) + unsupervised continuous moderate cycling (optional) + unsupervised swimming (optional) + unsupervised upper limb strength training (intensity not clear) + unsupervised education | NA | NA |
| Nguyen, 2013[216] | Usual care | High intensity continuous cycling + Education | NA | NA |
| Ninot, 2011[217] | Usual care | Supervised and unsupervised Tai Chi | NA | NA |
| Niu, 2014[218] | Supervised education + supervised high continuous walking + supervised moderate continuous cycling + supervised moderate upper and lower strength training + unsupervised aerobic exercise, not otherwise specified | Supervised education + supervised high continuous walking + supervised moderate continuous cycling + supervised moderate upper and lower strength training + unsupervised aerobic exercise, not otherwise specified +physical activity intervention | NA | NA |
| Nolan, 2017[219] | Supervised callisthenics + Supervised education | Supervised education + supervised very high continuous walking + supervised very high continuous cycling | NA | NA |
| Normandin, 2002[220] | Supervised moderate continuous walking + upper limb strength training, not otherwise specified + oxygen (optional) + chest physio | Supervised moderate continuous walking + upper limb strength training, not otherwise specified + oxygen (optional) + chest physio + behavioural intervention + breathing pattern retraining using visual feedback | Supervised moderate continuous walking + upper limb strength training, not otherwise specified + oxygen (optional) + chest physio + supervised education | NA |
| Norweg, 2005[221] | Supervised upper and lower limb resistance bands | Supervised upper and lower limb resistance bands - 1 leg | NA | NA |
| Nyberg, 2015[222] | Supervised education | Supervised Upper limb resistance bands, supervised lower limb resistance bands | NA | NA |
| Nyberg, 2021[223] | Unsupervised backpack wearing, low intensity | Unsupervised backpack + unsupervised upper and lower strength training, not otherwise specified | NA | NA |
| O’Hara, 1984[224] | Usual care | Supervised low walking + oxygen (optional) + supervised moderate upper and lower strength training | unsupervised low walking + oxygen (optional) + unsupervised moderate upper and lower strength training | NA |
| Oliveira, 2011[225] | Supervised upper and lower limb strength training | Supervised high continuous cycling | Supervised high continuous cycling + supervised upper and lower strength training | NA |
| Ortega, 2002[226] | Usual care | Water based callisthenics, intensity not stated | NA | NA |
| Ozdemir, 2010[227] | Supervised education | Supervised tai chi | NA | NA |
| Pan, 2018[228] | Supervised moderate continuous cycling + supervised moderate strength training, limb not specified + chest physio | Supervised moderate continuous cycling + supervised moderate strength training, limb not specified + chest physio + sham chest vibration | Supervised moderate continuous cycling + supervised moderate strength training, limb not specified + chest physio + chest wall vibration | NA |
| Pancera, 2021[229] | Supervised moderate continuous cycling + supervised moderate upper and lower strength training + chest physio (optional) + education supervised + nutritional support + psychological | Supervised moderate continuous cycling + supervised moderate upper strength training + chest physio (optional) + education supervised + nutritional support + psychological + downhill walking + ECC resistance | NA | NA |
| Pancera, 2023[230] | Unsupervised upper and lower resistance bands + physical activity intervention + Supervised education | Unsupervised upper and lower resistance bands + physical activity intervention + Supervised education | NA | NA |
| Park, 2020[231] | Low intensity walking + supervised lower and upper limb strength training + Bubble PEP | Low intensity walking + supervised lower and upper limb strength training + expiratory flow accelerator | NA | NA |
| Patrizio, 2019[232] | Placebo | Supervised high intensity continuous cycle + strength training upper limb + Placebo | Supervised high intensity continuous cycling + strength training lower limbs + B12 | NA |
| Paulin, 2017[233] | Supervised moderate aerobic exercise, not otherwise specified + unsupervised moderate aerobic exercise, not otherwise specified + supervised strength training (intensity or limb not specified) + supervised education + placebo | Supervised moderate aerobic exercise, not otherwise specified + unsupervised moderate aerobic exercise, not otherwise specified + supervised strength training (intensity or limb not specified) + supervised education + beetroot juice | NA | NA |
| Pavitt, 2018[234] | Usual care | Supervised high continuous walking + supervised high continuous cycling + supervised upper strength training, intensity not stated + breathing exercises | NA | NA |
| Paz-Diaz, 2007[235] | Supervised low walking + supervised high cycling + supervised lower moderate strength training + supervised education + supervised callisthenics + supervised quads NMES 50Hz | Supervised low walking + supervised high cycling + supervised lower moderate strength training + supervised education + supervised callisthenics + supervised quads NMES 50Hz + triceps NMES | NA | NA |
| Peran, 2018[236] | Supervised continuous walking, not otherwise specified + supervised upper and lower strength training, not otherwise specified + supervised education + supervised callisthenics + smoking cessation + psychological (optional) + nutrition (optional) + supervised high continuous cycling | Supervised continuous walking, not otherwise specified + supervised upper and lower strength training, not otherwise specified + supervised education + supervised callisthenics + smoking cessation + psychological (optional) + nutrition (optional) + NMES (high, quads) | NA | NA |
| Peran, 2022[237] | Supervised high continuous cycling | Supervised high continuous cycling + supervised upper and lower moderate strength training + breathing control | NA | NA |
| Pereira, 2010[238] | Supervised education | Supervised very high continuous walking + supervised continuous cycling, intensity not specified + flexibility + supervised education + unsupervised very high continuous walking | NA | NA |
| Petersen, 2008[239] | Usual care | Supervised vibration | NA | NA |
| Pleguezuelos, 2013[240] | Supervised moderate continuous walking and cycling + supervised education + stairs + rowing + supervised moderate upper and lower strength training | Supervised tai chi | NA | NA |
| Polkey, 2018[241] | Usual care | Unsupervised high continuous walking | Unsupervised high continuous walking + Star Fruit-Honey Product | NA |
| Pothasak, 2020[242] | Usual care | Unsupervised low intensity walking + stair climbing + unsupervised upper limb strength training | NA | NA |
| Pradella, 2015[243] | Supervised callisthenics + breathing exercises | Low intensity continuous walking, supervised high intensity continuous cycling + upper and lower strength training + oxygen (optional) | NA | NA |
| Probst, 2011[244] | moderate intensity overground walking | supervised high intensity treadmill walking | NA | NA |
| Puente-Maestu, 2000[245] | supervised upper and lower limb resistance | Upper and lower limb training with resistance bands (supervised) | NA | NA |
| Ramos, 2014[246] | Supervised high intensity continuous walking or cycling + upper and lower strength training + Nordic walking | Supervised high intensity continuous walking or cycling + upper and lower strength training + Physical activity counselling (Motivational interviewing) + Nordic walking | NA | NA |
| Rausch Osthoff, 2021[247] | Unsupervised home exercise, modality and intensity unclear | moderate continuous cycling + moderate continuous walking + upper limb strength training, not otherwise specified + education +IMT + stairs + Breathing retraining | NA | NA |
| Reardon, 1994[248] | Supervised high continuous walking + oxygen (optional) | Supervised high continuous walking + oxygen (optional) + NIV | NA | NA |
| Reuveny, 2005[249] | Supervised education | Supervised education + chest physio + breathing exercises + oxygen (optional) + psychosocial support + Supervised moderate intensity walking + unsupervised moderate intensity walking + unsupervised upper limb strength training | NA | NA |
| Ries, 1995[250] | Flexibility + balance + Nordic walking + circuit training +music | Supervised moderate continuous walking + supervised moderate continuous cycling + supervised arm cycling + supervised upper, lower and trunk moderate strength training + flexibility + balance | NA | NA |
| Rinaldo, 2017[251] | Usual care | Supervised high intensity walking + flexibility + supervised upper, lower and trunk strength training, not otherwise specified + stairs + supervised education + nutritional counselling + OT + unsupervised resistance bands, not otherwise specified | NA | NA |
| Ringbaek, 2000[252] | Supervised Very High Intensity Continuous Cycling | Supervised High Intensity Continuous Cycling | Supervised Very High intensity interval cycling | NA |
| Rizk, 2015[253] | Usual care | Supervised education + breathing exercises + supervised callisthenics | NA | NA |
| Roman, 2013[254] | Supervised upper and lower strength training + occupational therapy + dietary counselling + supervised education +oxygen (optional) | Supervised vibration + occupational therapy + dietary counselling + supervised education +oxygen (optional) | NA | NA |
| Salhi, 2015[255] | Placebo | Unsupervised low intensity walking + N-acetylcysteine | NA | NA |
| Salve, 2016[256] | Supervised very high continuous cycling + supervised upper and lower moderate strength training + supervised flexibility + supervised education | Supervised high continuous cycling + supervised upper and lower moderate strength training + supervised flexibility + supervised education | NA | NA |
| Santos, 2015[257] | Supervised education + aerobic training, not otherwise specified + supervised strength training not otherwise specified + vibration training + chest physio +smoking cessation (optional) + saline (optional) + psychological therapy (optional) + nutrition (optional) + OT (optional) + Sham IMT | Supervised education + aerobic training, not otherwise specified + supervised strength training not otherwise specified + vibration training + chest physio +smoking cessation (optional) + saline (optional) + psychological therapy (optional) + nutrition (optional) + OT (optional) +IMT | NA | NA |
| Schultz, 2018[258] | Supervised high intensity continuous cycling + air | Supervised high intensity continuous cycling + supplemental oxygen | Supervised high intensity continuous cycling + Heliox | NA |
| Scorsone, 2010[259] | Supervised high intensity continuous walking + Education + unsupervised high intensity continuous walking + upper and lower limb strength training (supervised), moderate + upper and lower limb strength training (unsupervised) + unsupervised education | Supervised high intensity continuous walking + Education + unsupervised high intensity continuous walking + upper and lower limb strength training (supervised), moderate + upper and lower limb strength training (unsupervised) + unsupervised education | NA | NA |
| Sewell, 2006[260] | Supervised exercise, not otherwise specified + supervised education + psychological support + writing about emotionally neutral subject (sham) | Supervised exercise, not otherwise specified + supervised education + psychological support + Written disclosure therapy | NA | NA |
| Sharifabad, 2010[261] | Supervised education | Supervised aerobic training, not otherwise specified + supervised education + chest physio + supervised breathing+ psychological + nutrition | NA | NA |
| Shui, 2023[262] | Supervised callisthenics + IMT + Flexibility | Supervised callisthenics + IMT + supervised upper limb strength training + Flexibility | NA | NA |
| Silva, 2003[263] | Supervised very high continuous walking + supervised callisthenics + Placebo | Supervised very high continuous walking + supervised callisthenics + L-carnitine | NA | NA |
| Silva, 2012[264] | Supervised low intensity walking + chest physio + L-carnitine | Supervised low intensity walking + chest physio + L-carnitine + IMT | NA | NA |
| Silva, 2018[265] | Supervised upper and lower strength training, intensity not specified | Supervised upper and lower strength training tubes | NA | NA |
| Silva, 2019[266] | Usual care | Supervised upper and lower strength training, moderate | NA | NA |
| Simpson, 1992[267] | Supervised high intensity continuous cycling + oxygen (optional) | Supervised high continuous cycling + upper limb strength training (supervised) | NA | NA |
| Sivori, 1998[268] | Usual care | Supervised callisthenics + upper limb strength training + supervised high continuous cycling | Water callisthenics (high intensity) + upper limb resistance | NA |
| Spielmanns, 2015[269] | Supervised high intensity interval training + air | Supervised high intensity interval training + oxygen | NA | NA |
| Spielmanns, 2017[270] | Supervised whole body vibration | Supervised Breathing retraining and relaxation, calisthenics | NA | NA |
| Spruit, 2002[271] | Supervised moderate continuous cycling, supervised low walking + arm cycling +stairs | Supervised upper and lower moderate strength training +stairs | NA | NA |
| Steiner, 2003[272] | Supervised high intensity continuous walking + flexibility training + unsupervised high continuous walking + supervised education | Supervised high intensity continuous walking + flexibility training + unsupervised high continuous walking + supervised education + macronutrient supplement | NA | NA |
| Strijbos, 1996[273] | Usual Care | Supervised walking + supervised stair climbing + supervised high intensity cycling + unsupervised walking + unsupervised stair climbing + breathing & relaxation techniques + optional bronchial clearance techniques | Supervised walking + supervised stair climbing + supervised high intensity cycling + unsupervised walking + unsupervised stair climbing + breathing & relaxation techniques + optional bronchial clearance techniques | NA |
| Stulbarg, 2002[274–277] | Supervised education + unsupervised high intensity continuous walking | Supervised education + unsupervised high intensity continuous walking + nurse coaching | NA | NA |
| Subin, 2010[278] | upper limb strength training + breathing exercises | Supervised continuous walking, intensity unclear + breathing exercises | Supervised continuous walking, intensity not specified + upper limb strength training + breathing exercises | NA |
| Sunil Kumar, 2020[279] | Supervised low intensity walking | Stair climbing | NA | NA |
| Suresh Babu Reddy, 2020[280] | Supervised high intensity continuous walking + supervised upper and lower strength training, not otherwise specified + breathing exercises + education + psychological support | Supervised high intensity continuous walking + supervised upper and lower strength training, not otherwise specified + breathing exercises + education + psychological support + Balance training | NA | NA |
| Suresh Babu Reddy, 2021[281] | Supervised high intensity continuous walking + supervised upper and lower strength training, not otherwise specified + breathing exercises + education + psychological support | Supervised high intensity continuous walking + supervised upper and lower strength training, not otherwise specified + breathing exercises + education + psychological support + Balance training | NA | NA |
| Sutanto, 2019[282] | Supervised high continuous cycling | Supervised high continuous cycling + Wii fit | NA | NA |
| Sykes, 2005[283] | Supervised moderate continuous cycling + supervised moderate upper strength training + supervised education | Supervised moderate continuous cycling + supervised moderate upper strength training + supervised education + IMT | NA | NA |
| Tabak, 2014[284] | Usual care | Unsupervised exercise, not otherwise specified + physical activity coaching + unsupervised education | NA | NA |
| Tabka, 2023[285] | Supervised low cycling + supervised education | Supervised low cycling + supervised education + cognitive training | NA | NA |
| Takahashi, 2014[286] | Supervised education | Supervised education + unsupervised callisthenics | NA | NA |
| Tasdemir, 2015[287] | Supervised high continuous cycling + supervised high continuous walking + supervised low lower strength training + supervised upper strength training, not otherwise specified + supervised education + nutrition + psychological + Sham NMES | Supervised high continuous cycling +supervised high continuous walking + supervised low lower strength training + supervised upper strength training, not otherwise specified + supervised education + nutrition + psychological + HF NMES | NA | NA |
| Tavanaei Youssefian, 2018[288] | Breathing exercises + Supervised moderate continuous cycling and walking + supervised upper limb strength training, not otherwise specified | Breathing exercises +unsupervised walking, intensity not stated + upper limb strength training (unsupervised), not otherwise specified | NA | NA |
| Thabitha, 2012[289] | Usual care | supervised upper and lower limb strength training | NA | NA |
| Theander, 2009[290] | Usual care | Supervised continuous cycling, intensity not specified + supervised upper and lower strength training, not otherwise specified + unsupervised upper resistance bands + dietary advice + occupational therapy + Supervised education + Unsupervised low intensity walking | NA | NA |
| Titova, 2008[291] | Supervised continuous walking, not otherwise specified + supervised breathing exercises + supervised education + nutrition + smoking cessation+ tiotropium | Supervised continuous walking, not otherwise specified + supervised breathing exercises + supervised education + nutrition + smoking cessation + tiotropium + almitrine | NA | NA |
| Titova, 2008[292] | Supervised continuous walking, not otherwise specified + supervised breathing exercises + supervised education + nutrition + smoking cessation | Supervised continuous walking, not otherwise specified + supervised breathing exercises + supervised education + nutrition + smoking cessation + tiotropium | NA | NA |
| Toledo, 2007[293] | Supervised very high continuous walking | Supervised very high continuous walking + NIV | NA | NA |
| Tounsi, 2021[294] | Supervised low intensity continuous walking | Supervised low intensity continuous walking + IMT | NA | NA |
| Troosters, 2000[295] | Usual care | supervised high continuous cycling + supervised low walking + stairs + supervised upper and lower moderate strength training + oxygen (optional) | NA | NA |
| Tsai, 2017[296] | Usual care | Supervised high intensity continuous walking + supervised high intensity continuous cycling + supervised lower limb strength training | NA | NA |
| Turnip, 2014[297] | Supervised continuous cycling, intensity not specified | Supervised continuous walking, intensity not specified | NA | NA |
| Valderramas, 2009[298] | Supervised upper and lower strength training, not otherwise specified + High intensity continuous walking + normal saline + Flexibility | Supervised upper and lower strength training not otherwise specified + High intensity continuous walking + hypertonic saline + Flexibility | NA | NA |
| Valenza, 2018[299] | usual care | NMES (50Hz) + breathing exercises | NA | NA |
| Vallet, 1994[300] | Supervised moderate intensity interval training + Education + Chest physio | High intensity interval cycling (supervised) + Education + Chest physio | NA | NA |
| Vallet, 1997[301] | breathing exercises + supervised strength training, not otherwise specified | Supervised high continuous walking + breathing exercises + supervised strength training, not otherwise specified | NA | NA |
| Van Gestel, 2012[302] | Supervised low intensity cycling + arm cycling + upper and lower resistance | Supervised low intensity cycling + arm cycling + supervised upper and lower strength training, moderate + controlled breathing training | NA | NA |
| van Wetering, 2010[303] | Usual care | Supervised and unsupervised continuous walking, not otherwise specified + supervised and unsupervised continuous cycling, not otherwise specified + supervised and unsupervised callisthenics + supervised education + smoking cessation + nutrition | NA | NA |
| Varga, 2006[304] | Supervised very high intensity continuous cycling | Supervised very high intensity interval cycling | NA | NA |
| Vasilopoulou, 2017[305] | Usual care | Supervised breathing exercises + supervised education + dietary advice + supervised very high interval cycling + supervised upper and lower strength training, not otherwise specified | NA | NA |
| Vivodtzev, 2012[306] | Unsupervised sham NMES | Unsupervised HF NMES | NA | NA |
| Vogiatzis, 2002[307] | Supervised moderate continuous cycling + breathing control (supervised) + chest clearance + dietary advice + supervised education + psychological support | Supervised very high interval cycling + breathing control (supervised) + chest clearance + dietary advice + supervised education + psychological support | NA | NA |
| Vogiatzis, 2005[308] | Supervised breathing exercises + chest physio + supervised education + dietary advice + psychological support + supervised high continuous cycling + oxygen (optional) | Supervised breathing exercises + chest physio + supervised education + dietary advice + psychological support + supervised very high interval cycling + oxygen (optional) | NA | NA |
| Vonbank, 2012[309] | Supervised moderate continuous cycling | Supervised moderate upper, lower and trunk strength training | Supervised moderate upper, lower and trunk strength training + supervised moderate continuous cycling | NA |
| Wada, 2016[310] | Supervised high intensity continuous walking + Sham flexibility training | Supervised high continuous walking + breathing exercises | NA | NA |
| Wadell, 2004[311] | Supervised callisthenics + supervised upper, lower and torso strength training + music | Supervised callisthenics in water, high + supervised upper, lower and torso strength training in water + music | NA | NA |
| Wadell, 2013[312] | Unsupervised education | Supervised moderate continuous walking + supervised moderate continuous cycling + supervised education + arm cycling + upper and lower strength training (supervised) + flexibility training | NA | NA |
| Wang, 2017[313] | Low intensity walking | Supervised high intensity interval cycling | Supervised high intensity interval cycling +IMT | NA |
| Wang, 2019[314] | Usual care | Tai Chi (supervised and unsupervised) | NA | NA |
| Wang, 2023[315] | Supervised education + dietary + supervised moderate continuous cycling + supervised moderate arm cycling + smoking cessation | Supervised education + dietary + supervised high continuous cycling + supervised high arm cycling + smoking cessation | Supervised education + dietary + supervised very high continuous cycling + supervised very high arm cycling + smoking cessation | NA |
| Wanke, 1994[316] | Supervised high continuous cycling | Supervised high continuous cycling + IMT | NA | NA |
| Waterhouse, 2010[317] | Supervised interval walking, not otherwise specified + callisthenics supervised + supervised education | Supervised interval walking, not otherwise specified + callisthenics supervised + supervised education | NA | NA |
| Wedzicha, 1998[318,319] | Supervised education | Supervised education + supervised moderate continuous walking + low cycling + supervised callisthenics + unsupervised exercise, not otherwise specified | NA | NA |
| Weiner, 1992[320] | Supervised education | Supervised education + supervised moderate continuous walking + supervised callisthenics + unsupervised moderate continuous walking | NA | NA |
| Wen, 2008[321] | Usual care | Supervised moderate continuous cycling + rowing + supervised upper, lower and trunk strength training + sham IMT | Supervised moderate continuous cycling + rowing + supervised upper, lower and trunk strength training + IMT | NA |
| White, 2002[322] | Supervised moderate continuous cycling + unsupervised moderate walking + stairs (optional) | Supervised high continuous cycling + unsupervised high continuous walking + stairs (optional) | NA | NA |
| Whitsel, 1998[323] | Unsupervised low walking + unsupervised upper and lower strength training not otherwise specified + steps + unsupervised education | Supervised high continuous walking +steps + upper limb resistance bands + supervised lower strength training, not otherwise specified + supervised education | NA | NA |
| Widyastuti, 2018[324] | Supervised education + Supervised moderate aerobic training, not otherwise specified + supervised upper and lower strength training, not prescribed | Supervised education + Supervised moderate aerobic training, not otherwise specified + supervised upper and lower strength training, not prescribed + nutritional supplement | NA | NA |
| Wijkstra, 1996[325] | High intensity continuous walking + low intensity unsupervised walking | unsupervised moderate continuous walking | NA | NA |
| Wittmann, 2007[326,327] | Usual care | Unsupervised breathing retraining + supervised education + IMT + unsupervised flexibility training + unsupervised high continuous cycling + supervised high continuous cycling | NA | NA |
| Wiyono, 2006[328] | Supervised exercise, not otherwise specified + breathing exercises + psychological + nutrition + smoking cessation | Supervised exercise, not otherwise specified + breathing exercises + psychological + nutrition + smoking cessation + supervised education | NA | NA |
| Wootton, 2014[329,330] | Usual care | Supervised continuous cycling, intensity not specified +education + chest physio | NA | NA |
| Wright, 2003[331] | Usual care | Supervised high intensity continuous walking | NA | NA |
| Wu, 2015[332] | Usual care | Supervised upper, lower and core resistance | NA | NA |
| Wu, 2018[333] | Usual care | Liuzijue exercise (supervised and unsupervised) | Liuzijue exercise + upper and lower limbs resistance bands | NA |
| Wu, 2018[334] | Usual care | Unsupervised moderate aerobic exercise, not otherwise specified | NA | NA |
| Wurtemberger, 2001[335] | Usual care | Supervised very high continuous cycling | NA | NA |
| Xi, 2015[336] | Supervised high continuous cycling + education + psychological intervention (optional) | Supervised upper and lower strength training + education + psychological intervention (optional) | Supervised high continuous cycling + education + psychological intervention (optional) + supervised upper and lower strength training | NA |
| Xiao, 2015[337] | Supervised education | Breathing exercises (unsupervised) + unsupervised upper and lower limb strength training (not prescribed) | NA | NA |
| Xu, 2023[338] | Breathing retraining (supervised) + Unsupervised continuous walking, not otherwise specified | Unsupervised continuous walking, not otherwise specified + Unsupervised Liuzijue | NA | NA |
| Yeh, 2020[339] | Supervised moderate continuous cycling + education + nutrition + psychological | Supervised moderate continuous cycling + supervised high upper and lower strength training + education + nutrition + psychological | Supervised moderate continuous cycling + supervised high upper and lower strength training + education + nutrition + psychological + diaphragm pacing | NA |
| Yekefallah, 2019[340] | Supervised education | Tai Chi (supervised and unsupervised) | NA | NA |
| Yudhawati, 2019[341] | Usual care | Supervised upper limb strength training, not otherwise specified | NA | NA |
| Zambom-Ferraresi, 2015[342] | Unsupervised education (brochure) | Supervised Yoga + supplemental oxygen | NA | NA |
| Zanini, 2015[343] | Usual care | Supervised moderate continuous cycling + supervised upper and lower strength training | Supervised upper and lower limb resistance | NA |
| Zanotti, 2011[344] | Supervised low walking + Education + Arm cycling OR upper limb strength training (intensity not specified) + airway clearance (optional) + breathing retraining (optional) + IMT (optional) | Supervised low walking + Education + Arm cycling OR upper limb strength training (intensity not specified) + airway clearance (optional) + breathing retraining (optional) + IMT (optional) + supervised upper and lower moderate resistance | NA | NA |
| Zanotti, 2012[345] | Low intensity continuous walking + Education + psychological counselling (optional) + nutrition (optional) + sham NMES | Low intensity continuous walking + Education + psychological counselling (optional) + nutrition (optional) +NMES | NA | NA |
| Zhang, 2012[346] | Supervised high continuous cycling + arm cycling + supervised education + psychological counselling + nutrition + soft manipulation (sham osteopathy treatment) | Supervised high continuous cycling + arm cycling + supervised education + psychological counselling + nutrition + osteopathy | NA | NA |
| Zhang, 2016[347] | Supervised education | Supervised education + unsupervised daoyin | NA | NA |
| Zhang, 2017[348] | Usual care | Unsupervised low walking + breathing exercise | Supervised and unsupervised qigong | NA |
| Zhang, 2020[349] | Usual care | Unsupervised continuous walking, not otherwise specified + breathing exercises + nutrition + psychological (optional) | NA | NA |
| Zhuang, 2023[350] | Usual care | Unsupervised tai chi + unsupervised breathing exercises | NA | NA |
| 陆绍勇, 2022[351] | Supervised education | Supervised education + unsupervised qigong | NA | NA |

### **Supplementary table 16 -** Demographics of cohorts in included studies.

| Study | Total participants | Age (years) | Percentage of males | Ethnicity | Religion | Socioeconomic group | BMI (kg/m2) | GOLDI% | GOLDII% | GOLDIII% | GOLDIV% | FEV1 (L) | FEV1 (%predicted) |
| --- | --- | --- | --- | --- | --- | --- | --- | --- | --- | --- | --- | --- | --- |
| Gouzi, 2019[128] | 57 | 62.26 | 49% | U | U | U | 25.14 | U | U | U | U | U | 59.28 |
| Rinaldo, 2017[251] | 24 | 66.20 | 100% | U | U | U | 29.15 | U | U | U | U | U | 66.15 |
| Rizk, 2015[253,352] | 35 | 67.31 | 40% | U | U | U | 27.89 | U | U | U | U | 1.44 | 60.24 |
| Paulin, 2017[233] | 24 | 59.93 | 46% | U | U | U | 25.97 | 0% | 13% | 67% | 21% | 1.12 | 38.30 |
| Deering, 2011[83] | 60 | 67.29 | 52% | U | U | U | 27.64 | 10% | 30% | 42% | 17% | U | 47.74 |
| Borghi-Silva, 2010[37] | 24 | 67.50 | 64% | U | U | U | 24.50 | 0% | 39% | U | U | 0.80 | 33.50 |
| Bernard, 1999[26] | 45 | 65.27 | 78% | U | U | U | 26.16 | U | U | U | U | 1.12 | 42.47 |
| Borghi-Silva, 2009[36] | 34 | 67.00 | 63% | U | U | U | 24.59 | U | U | U | U | 0.78 | 33.82 |
| Bronstad, 2013[45] | 17 | 64.84 | 71% | U | U | U | 25.03 | U | U | U | U | 1.72 | 52.78 |
| Bjorgen, 2009[31] | 19 | 61.37 | 37% | U | U | U | 24.63 | 0% | 0% | 100% | 0% | 1.21 | 42.66 |
| Bjorgen, 2009[32] | 12 | 61.00 | 42% | U | U | U | 25.58 | 0% | 0% | 100% | 0% | 1.17 | 39.67 |
| Louvaris, 2016[181] | 128 | 65.67 | 81% | U | U | U | 27.40 | 4% | 42% | 32% | 22% | U | 47.49 |
| Patrizio, 2019[232] | 20 | 72.50 | U | U | U | U | U | U | U | U | U | 1.78 | U |
| Curtis, 2016[75] | 65 | 67.05 | 52% | U | U | U | 25.52 | U | U | U | U | 1.21 | 49.98 |
| Mkacher, 2015[203] | 62 | 62.20 | 100% | U | U | U | 25.80 | U | U | U | U | U | 39.80 |
| Yeh, 2020[339] | 81 | 68.43 | 66% | 81% White, 12% Black, 8% Other | U | 57% Annual income <US$35K | U | U | U | U | U | U | 57.33 |
| Armstrong, 2021[14] | 48 | 72.00 | 60% | U | U | U | 27.15 | U | U | U | U | 1.24 | 50.50 |
| Wiyono, 2006[328] | 56 | 65.80 | 93% | U | U | U | 19.96 | 38% | 63% | 0% | 0% | 1.18 | U |
| Widyastuti, 2018[324] | 36 | 64.75 | 86% | U | U | 62% ‘Low education’ | 21.30 | 14% | 47% | 28% | 11% | 1.00 | U |
| Gohl, 2006[127] | 19 | 62.83 | 68% | U | U | U | 27.11 | U | U | U | U | U | 53.54 |
| He, 2019[138] | 203 | 65.27 | 57% | U | U | U | 21.14 | 0% | 41% | 59% | 0% | U | 48.97 |
| Collins, 2008[64] | 42 | 66.57 | 97% | U | U | U | 29.03 | U | U | U | U | 1.35 | 41.95 |
| De Sousa Pinto, 2014[81] | 41 | 70.22 | 95% | U | U | 90% Primary school education or less | 26.18 | 0% | 0% | 46% | 54% | 0.90 | 33.94 |
| Zanotti, 2012[345] | 83 | 61.78 | 72% | U | U | U | 24.60 | U | U | U | U | U | 59.85 |
| Laviolette, 2010[168] | 22 | 65.04 | 64% | U | U | U | 28.34 | U | U | U | U | 1.26 | 50.18 |
| Blackstock, 2014[33] | 267 | 72.21 | 60% | U | U | U | 27.56 | U | U | U | U | 1.35 | 57.42 |
| Kilic, 2021[159] | 58 | 69.19 | 90% | U | U | U | U | 5% | 55% | 19% | 21% | U | 54.14 |
| Strijbos, 1996[273] | 45 | 61.40 | 84% | U | U | U | U | U | U | U | U | 1.23 | 42.80 |
| Dong, 2021[89] | 20 | 64.55 | U | U | U | U | 23.75 | U | U | U | U | U | U |
| Horton, 2018[146] | 224 | 67.59 | 67% | U | U | U | 27.36 | U | U | U | U | 1.20 | 43.21 |
| Duruturk, 2016[93] | 42 | 62.00 | 83% | U | U | U | 26.86 | 0% | U | U | 0% | 1.70 | 59.61 |
| Ortega, 2002[226] | 47 | 64.21 | 87% | U | U | U | U | U | U | U | U | 1.07 | 38.26 |
| Puente-Maestu, 2000[245] | 41 | 64.42 | 100% | U | U | U | 25.59 | U | U | U | U | 1.09 | 40.51 |
| Turnip, 2014[297] | 44 | U | 95% | U | U | U | U | 14% | 48% | 39% | 0% | U | U |
| Vallet, 1997[301] | 24 | 57.00 | 75% | U | U | U | 25.17 | U | U | U | U | 1.78 | 58.50 |
| Collins, 2014[65] | 103 | 68.98 | 95% | U | U | U | 29.01 | U | U | U | U | 1.29 | 43.96 |
| Farias, 2014[106] | 34 | 67.38 | 50% | U | U | U | 27.31 | U | U | U | U | U | 53.67 |
| Faager, 2006[104] | 23 | 65.70 | 43% | U | U | U | 23.70 | U | U | U | U | U | 43.13 |
| Fuld, 2005[115] | 38 | 62.75 | 61% | U | U | U | 23.78 | 0% | U | U | U | 1.13 | 45.40 |
| Larson, 1999[167] | 40 | 65.50 | 66% | U | U | U | 26.00 | U | U | U | U | U | 48.70 |
| Wang, 2017[313] | 81 | 70.21 | 80% | U | U | U | 21.67 | U | U | U | U | 1.31 | 52.78 |
| Marrara, 2008[194] | 16 | 69.00 | 100% | U | U | U | 23.50 | U | U | U | U | U | 46.00 |
| Karapolat, 2007[155] | 49 | 65.77 | 88% | U | U | U | U | 6% | 57% | 18% | 0% | U | 25.00 |
| Scorsone, 2010[259] | 30 | 67.33 | 77% | U | U | U | 27.00 | U | U | U | U | U | 48.67 |
| de Bisschop, 2021[76] | 54 | 64.90 | 67% | U | U | U | 28.33 | U | U | U | U | 1.57 | 58.60 |
| Aldhahir, 2021[7] | 44 | 72.50 | 64% | U | U | U | 23.50 | U | U | U | U | 1.40 | 55.50 |
| Moezy, 2018[205] | 30 | 65.60 | 80% | U | U | 27% higher than high school diploma | 24.16 | U | U | U | U | U | 59.58 |
| Bourbeau, 2020[38] | 24 | 66.71 | 100% | U | U | U | 25.39 | 0% | 16% | 65% | 19% | 1.09 | 37.11 |
| Abdelbasset, 2020[2] | 40 | 71.30 | 73% | U | U | U | 34.71 | 0% | 100% | 0% | 0% | U | 63.80 |
| Moore, 2009[207] | 20 | 70.25 | 50% | U | U | U | U | U | U | U | U | 0.95 | U |
| Naseer, 2017[210] | 30 | 54.10 | 100% | U | U | U | 23.99 | U | U | U | U | U | 46.57 |
| Mekki, 2019[200] | 45 | 59.56 | 100% | U | U | U | 25.60 | U | U | U | U | 1.71 | 57.44 |
| Acheche, 2020[4] | 42 | 62.52 | 100% | U | U | U | 24.59 | U | U | U | U | 1.71 | 54.45 |
| Suresh Babu Reddy, 2021[281] | 128 | 52.32 | 80% | U | U | U | U | 0% | 100% | 0% | 0% | U | 68.36 |
| Suresh Babu Reddy, 2020[280] | 20 | 55.20 | 75% | U | U | U | U | 100% | 0% | 0% | 0% | U | U |
| Chen, 2015[60] | 60 | 55.90 | 58% | U | U | U | U | 0% | 28% | 72% | 0% | U | 35.02 |
| Collins, 2019[66] | 119 | 66.00 | 95% | U | U | U | 29.64 | 0% | U | U | U | 1.29 | 44.00 |
| Reardon, 1994[248] | 20 | 66.20 | 50% | U | U | U | U | 0% | 10% | 55% | 35% | 0.87 | 34.00 |
| Rausch Osthoff, 2021[247] | 42 | 68.21 | 50% | U | U | U | 25.81 | 0% | U | U | U | 1.18 | 48.39 |
| Iepsen, 2016[147] | 30 | 63.00 | 43% | U | U | U | 27.00 | U | U | U | U | 1.60 | 56.00 |
| Elmorsi, 2016[98] | 40 | 56.45 | 100% | U | U | U | 25.23 | 0% | 30% | 43% | 28% | U | 39.91 |
| Wu, 2018[334] | 50 | 65.64 | 82% | U | U | U | 22.76 | 0% | 74% | 26% | 0% | 1.49 | 54.32 |
| Tavanaei Youssefian, 2018[288] | 60 | U | 27% | U | U | 27% illiterate | 25.00 | 7% | 35% | 58% | 0% | U | U |
| Lee, 2022[169] | 17 | 69.29 | 94% | U | U | U | 24.62 | U | U | U | U | 1.60 | U |
| Thabitha, 2012[289] | 30 | U | U | U | U | U | U | U | U | U | U | U | U |
| Ahnfeldt-Mollerup, 2015[5] | 53 | 68.42 | 43% | U | U | U | 23.82 | 4% | 43% | 36% | 38% | U | U |
| De Godoy, 2005[78] | 33 | 62.58 | 73% | 93% White | U | 55% had monthy income greater than three minimum wages | 22.58 | U | U | U | U | 0.89 | 33.42 |
| Mador, 2005[186] | 29 | 70.28 | U | U | U | U | 27.62 | U | U | U | U | 1.47 | 44.57 |
| Karagiannis, 2021[154] | 36 | 67.50 | 100% | U | U | U | 24.55 | U | U | U | U | 1.20 | 46.30 |
| Niu, 2014[218] | 40 | 60.50 | 93% | U | U | U | 21.48 | 0% | U | U | U | 1.20 | 42.80 |
| Dourado, 2009[90] | 24 | 63.52 | 79% | U | U | U | 25.07 | U | U | U | U | 1.25 | 58.54 |
| Subin, 2010[278] | 27 | 58.74 | 100% | U | U | U | 19.56 | 0% | U | U | 0% | U | 44.67 |
| Ozdemir, 2010[227] | 50 | 62.50 | 100% | U | U | U | 26.15 | 0% | U | U | 0% | U | 54.30 |
| Yudhawati, 2019[341] | 30 | 64.87 | 100% | U | U | U | 22.01 | 7% | 27% | 30% | 37% | 0.98 | 42.20 |
| Valderramas, 2009[298] | 68 | 67.50 | 60% | U | U | U | 26.30 | U | U | U | U | 1.29 | 47.00 |
| de Roos, 2018[80] | 45 | 70.25 | 30% | U | U | U | 27.67 | 0% | 100% | 0% | 0% | U | 66.40 |
| Casey, 2013[55] | 350 | 68.60 | 64% | U | U | 15% in work | U | 0% | 72% | 28% | 0% | 1.50 | 58.63 |
| Chan, 2010[56,353,354] | 206 | 72.95 | 91% | U | 41% had religious beliefs | 59% lowest income bracket | 21.73 | 16% | 42% | 43% | 0% | 0.90 | 53.84 |
| Norweg, 2005[221] | 33 | 74.20 | 33% | 98% White, 2% African American | U | 22% employed, remainder retired. 84% had a college education and 17% had completed high school | 26.04 | U | U | U | U | 1.23 | 55.00 |
| Sunil Kumar, 2020[279] | 60 | U | U | U | U | U | U | 0% | 100% | 0% | 0% | U | U |
| Ng, 2014[214] | 192 | 74.14 | 91% | U | U | 74% primary school education or less | 22.78 | 20% | 41% | 30% | 9% | 1.17 | 59.82 |
| Ferreira, 2013[110] | 32 | 63.16 | 63% | U | U | U | 27.63 | 0% | U | U | U | U | 42.40 |
| Finnerty, 2001[113] | 65 | 69.51 | 68% | U | U | U | 24.87 | U | U | U | U | 1.02 | 41.20 |
| Ho, 2012[140] | 40 | 74.00 | 5% | U | U | U | 24.15 | 22% | 39% | 34% | 5% | 1.30 | 60.90 |
| Varga, 2006[304] | 39 | 63.62 | 77% | U | U | U | 25.56 | U | U | U | 0% | 1.59 | 56.67 |
| Probst, 2011[244] | 40 | 66.00 | 53% | U | U | U | 26.50 | U | U | U | U | U | 39.50 |
| Cambach, 1997[49] | 23 | 62.00 | 57% | U | U | U | U | U | U | U | U | 1.60 | 59.00 |
| Arnedillo, 2020[17] | 16 | 68.49 | 100% | U | U | U | 27.28 | U | U | U | U | 1.64 | 49.67 |
| Wright, 2003[331] | 28 | 55.70 | 43% | U | U | U | U | 0% | U | U | 0% | U | 59.00 |
| Marquis, 2008[193] | 16 | 68.88 | 50% | U | U | U | 26.91 | U | U | U | U | 1.03 | 45.88 |
| Nakamura, 2008[208] | 33 | 68.92 | U | U | U | U | 21.89 | 0% | U | U | U | 1.40 | 51.49 |
| Wada, 2016[310] | 28 | 62.50 | 54% | U | U | U | 25.80 | 0% | U | U | 0% | U | 44.25 |
| Wanke, 1994[316] | 42 | 56.00 | 52% | U | U | U | 25.65 | U | U | U | U | 1.33 | 46.00 |
| Zambom-Ferraresi, 2015[342] | 36 | 68.22 | 96% | U | U | U | 28.19 | 0% | U | U | 0% | U | 44.79 |
| Aquino, 2016[12] | 28 | 67.21 | 100% | U | U | U | U | U | U | U | U | U | 68.42 |
| Alcazar, 2019[6] | 29 | 78.79 | 83% | U | U | U | 30.71 | U | U | U | U | U | 53.24 |
| Van Gestel, 2012[302] | 40 | 66.15 | 43% | U | U | U | 25.40 | 10% | 45% | 40% | 5% | 1.21 | 45.67 |
| Camillo, 2020[51] | 44 | 62.00 | 64% | U | U | U | 25.00 | U | U | U | U | U | 50.00 |
| Inostroza, 2022[148] | 20 | 69.65 | 50% | U | U | U | 28.60 | 0% | 100% | 0% | 0% | 1.79 | 70.90 |
| Ramos, 2014[246] | 34 | 66.50 | 71% | U | U | U | U | U | U | U | U | 1.20 | U |
| Felcar, 2018[108] | 36 | 68.56 | 64% | U | U | U | 26.00 | 3% | 47% | 33% | 17% | U | 47.11 |
| Wijkstra, 1996[325] | 43 | 63.30 | 86% | U | U | U | U | 0% | U | U | U | 1.20 | 44.35 |
| Chen, 2018[61] | 47 | 67.13 | 79% | U | U | Education: 47% Below junior high. 36% moderate to severe self perceived economic burden | 24.00 | 0% | U | U | U | U | 54.70 |
| Valenza, 2018[299] | 36 | 68.97 | 67% | U | U | U | 26.93 | U | U | U | U | U | 40.91 |
| Beaumont, 2018[22] | 149 | 64.06 | 63% | U | U | U | 25.44 | 0% | 0% | 46% | 54% | U | 35.29 |
| Nasis, 2009[211] | 42 | 65.50 | 79% | U | U | U | 25.05 | 0% | 33% | 36% | 31% | 1.10 | 42.15 |
| Li, 2018[172] | 36 | 66.00 | 78% | U | U | U | 23.00 | 0% | 64% | 36% | 0% | U | 57.08 |
| Gianjoppe-Santos, 2021[122] | 31 | 68.97 | 77% | U | U | U | 25.58 | 0% | U | U | U | 1.23 | 48.45 |
| Kawagoshi, 2015[156] | 27 | 74.56 | 89% | U | U | U | 21.87 | U | U | U | U | 1.46 | 59.44 |
| Takahashi, 2014[286] | 67 | 72.45 | 100% | U | U | U | 20.03 | 0% | U | U | U | 1.31 | 45.76 |
| Nyberg, 2021[223] | 33 | 66.00 | 70% | U | U | U | 25.10 | 0% | 0% | 76% | 24% | 1.00 | 39.00 |
| Spielmanns, 2015[269] | 36 | 64.53 | U | U | U | U | 24.94 | 0% | U | U | U | 1.34 | 43.53 |
| Liu, 2021[176] | 55 | 68.05 | 80% | U | U | U | 27.54 | 0% | 75% | 25% | 0% | U | 47.35 |
| Theander, 2009[290] | 26 | 64.92 | 50% | U | U | U | 24.68 | 0% | U | U | U | U | 33.59 |
| Ries, 1995[250] | 119 | 62.59 | 73% | U | U | U | U | U | U | U | U | 1.23 | U |
| Jang, 2006[150] | 36 | U | 100% | U | 56% "Religious" | 39% employed, 89% low income (<1,000,000 Won), 56% ≤Elementary school education | U | 0% | U | U | 0% | U | 48.66 |
| Tounsi, 2021[294] | 32 | 62.50 | 100% | U | U | U | 23.27 | 0% | 9% | 66% | 25% | U | 38.00 |
| Wang, 2019[314] | 50 | 67.84 | 88% | U | U | U | U | 0% | 24% | 46% | 30% | 1.18 | 58.86 |
| Kantatong, 2020[153] | 50 | 63.58 | 68% | U | U | U | U | 66% | 34% | 0% | 0% | 1.31 | 68.29 |
| Johnson, 2002[151] | 32 | 69.25 | 59% | U | U | U | U | 0% | 0% | U | U | U | 32.07 |
| Costi, 2009[69] | 50 | 69.50 | 66% | U | U | U | 26.00 | 0% | U | U | 0% | 1.02 | 40.90 |
| Silva, 2018[265] | 51 | 67.56 | 41% | U | U | U | 27.31 | 24% | 27% | 37% | 12% | 1.25 | 55.88 |
| Gallo-Silva, 2019[117] | 19 | 66.39 | 100% | U | U | U | 24.38 | U | U | U | U | 1.56 | 47.85 |
| Liu, 2021[177] | 45 | 65.36 | 73% | U | U | U | 23.21 | 16% | 62% | 18% | 4% | U | 57.68 |
| Gloeckl, 2012[124] | 82 | 64.49 | 51% | U | U | U | 25.46 | 0% | 0% | U | U | U | 38.51 |
| Salhi, 2015[255] | 62 | 60.50 | 71% | U | U | U | 24.50 | 0% | U | U | U | 1.10 | 38.50 |
| Pleguezuelos, 2013[240] | 51 | 69.82 | 100% | U | U | U | 26.15 | 0% | 0% | U | U | 1.05 | 34.60 |
| Elci, 2008[95] | 78 | 59.18 | 85% | U | U | U | U | 77% | 31% | 51% | 10% | U | 47.03 |
| Liu, 2008[175] | 48 | 72.10 | 100% | U | U | U | 23.20 | U | U | U | U | 0.98 | 45.60 |
| Xiao, 2015[337] | 126 | 71.55 | 93% | U | U | U | 19.45 | 0% | U | U | U | U | 41.10 |
| Wu, 2018[333] | 94 | 64.17 | 85% | U | U | U | 22.84 | 0% | 27% | 52% | 21% | 1.12 | U |
| Roman, 2013[254] | 71 | 64.17 | 82% | U | U | 26% in work | 28.66 | 0% | 100% | 0% | 0% | U | 19.97 |
| Mador, 2004[185] | 24 | 70.75 | U | U | U | U | 27.55 | U | U | U | U | 1.38 | 41.83 |
| Gimenez, 2000[123] | 13 | 49.13 | 100% | U | U | U | 26.28 | 0% | U | U | U | 1.60 | U |
| Park, 2020[231] | 42 | 67.88 | 79% | U | U | 38% Low income, 57% High school education or higher | U | U | U | 79% | 0% | U | 65.02 |
| Zhang, 2017[348] | 461 | 63.41 | 65% | U | U | 69% Elementary education only | 23.43 | 0% | 47% | 38% | 15% | 1.21 | 47.88 |
| Normandin, 2002[220] | 40 | 68.00 | 53% | U | U | U | 27.00 | U | U | U | U | U | 49.50 |
| Carrieri-Kohlman, 1996[52] | 51 | 66.94 | 49% | U | U | Mean 15 years of education | U | 0% | U | U | U | 0.91 | 37.88 |
| Stulbarg, 2002[274,276] | 60 | 65.98 | 46% | U | U | U | 25.64 | 0% | U | U | U | 1.04 | 43.18 |
| Sivori, 1998[268] | 28 | 64.57 | 82% | U | U | U | 24.03 | 0% | 0% | U | U | 1.01 | 36.11 |
| Marques, 2014[192] | 42 | 67.42 | 67% | U | U | 38% primary school education only | 28.01 | 38% | 38% | U | U | 1.76 | 70.48 |
| Wurtemberger, 2001[335] | 37 | 65.06 | 54% | U | U | U | 27.32 | 0% | U | U | U | 1.47 | 57.16 |
| Leung, 2010[170] | 36 | 71.50 | 69% | U | U | U | 26.50 | U | U | U | U | U | 54.50 |
| Wootton, 2014[329] | 143 | 68.66 | 59% | U | U | U | 25.67 | 0% | 43% | 44% | 12% | 1.15 | 43.00 |
| Eves, 2009[103] | 38 | 65.50 | 61% | U | U | U | 26.62 | 0% | U | U | U | 1.36 | U |
| Wadell, 2004[311] | 30 | 65.00 | 30% | U | U | U | 28.05 | 0% | U | U | U | 1.29 | 54.50 |
| Guell, 2008[132] | 51 | 64.46 | 100% | U | U | U | U | 0% | 0% | 100% | 0% | 1.12 | 38.18 |
| Bonnevie, 2018[34] | 51 | 59.20 | 86% | U | U | U | 23.50 | 0% | 10% | 51% | 39% | 1.00 | 35.00 |
| Pradella, 2015[243] | 44 | 63.39 | 82% | U | U | U | 25.71 | 5% | 32% | 39% | 14% | 1.30 | 47.34 |
| Holland, 2017[143] | 166 | 69.00 | 60% | U | U | U | 28.48 | U | U | U | U | 1.28 | 50.45 |
| Tsai, 2017[296] | 36 | 73.94 | 50% | U | U | 67% currently using a computer | 28.00 | 31% | 33% | 36% | 0% | 1.35 | 63.78 |
| Jolly, 2014[152] | 50 | 66.50 | 56% | U | U | U | 25.45 | 0% | U | U | 0% | 1.13 | 46.50 |
| Sewell, 2006[260] | 100 | 70.08 | 56% | U | U | U | 28.37 | U | U | U | U | 1.14 | 49.05 |
| Guell, 2006[131] | 35 | 64.46 | 94% | U | U | U | 25.63 | 0% | U | U | U | U | 34.91 |
| Wadell, 2013[312] | 48 | 66.83 | 56% | U | U | U | 27.98 | U | U | U | U | 1.20 | 48.00 |
| Casaburi, 2005[54] | 108 | 66.60 | 56% | U | U | U | 25.90 | 0% | U | U | U | 0.88 | 34.40 |
| Hasegawa, 2012[135] | 21 | 70.66 | U | U | U | U | 22.17 | 0% | 62% | 29% | 10% | U | 58.51 |
| Silva, 2003[263] | 20 | 66.70 | 70% | U | U | U | 23.80 | 0% | U | U | U | 1.10 | U |
| Weiner, 1992[320] | 36 | 64.63 | 50% | U | U | U | U | 0% | 3% | 58% | 39% | U | 35.23 |
| Chaplin, 2017[57] | 103 | 66.25 | 69% | U | U | U | 28.61 | 21% | 37% | 24% | 17% | 1.54 | 56.83 |
| Vallet, 1994[300] | 20 | 58.90 | U | U | U | U | 25.11 | 0% | U | U | U | 1.79 | U |
| Nguyen, 2013[216] | 125 | 68.70 | 54% | U | U | Education: 44% college or more. 75% unemployed or retired | 27.30 | U | U | U | U | U | 51.10 |
| Vogiatzis, 2002[307] | 36 | 68.00 | 83% | U | U | U | 27.60 | 0% | U | U | U | 1.15 | 44.90 |
| Arnardottir, 2007[16] | 60 | 64.47 | 15% | U | U | U | 23.78 | 0% | U | U | U | 0.85 | 33.40 |
| Mador, 2009[187] | 41 | 71.81 | U | U | U | U | 28.74 | U | U | U | U | 1.47 | 43.19 |
| Coppoolse, 1999[68] | 19 | 65.11 | 100% | U | U | U | 24.89 | 0% | U | U | 0% | U | 36.83 |
| Alexander, 2012[8] | 25 | 70.04 | U | U | U | U | 29.42 | U | U | U | U | U | 41.12 |
| Nalbant, 2011[209] | 21 | 70.62 | 100% | U | U | U | 22.46 | 0% | 29% | 71% | 0% | 1.28 | 44.14 |
| Borghi-Silva, 2006[35] | 16 | 67.00 | 63% | U | U | U | 22.50 | 0% | 0% | U | U | 0.86 | U |
| Bianchi, 2002[29] | 33 | 64.45 | 100% | U | U | U | 25.89 | 36% | 24% | 39% | 0% | 1.32 | 44.25 |
| Berry, 2010[27] | 176 | 66.00 | 54% | U | U | U | U | 7% | 44% | 36% | 13% | 1.47 | 51.76 |
| Xi, 2015[336] | 60 | 74.20 | 75% | U | U | U | U | 0% | 13% | 35% | 52% | 1.43 | 42.62 |
| Guell, 2000[130] | 60 | 65.00 | 100% | U | U | U | U | 0% | U | U | U | U | 35.00 |
| Arbillaga-Etxarri, 2018[13] | 280 | 69.00 | 87% | U | U | 72% low socioecomonic status | 28.25 | 10% | 53% | 31% | 6% | U | 57.00 |
| Clark, 1996[62] | 48 | 57.00 | U | U | U | U | 25.00 | U | U | U | U | 1.63 | 61.00 |
| Nyberg, 2015[222] | 40 | 68.50 | 52% | U | U | U | 25.50 | 41% | 34% | 5% | 21% | 1.55 | 57.00 |
| Spielmanns, 2017[270] | 27 | 69.48 | 52% | U | U | U | 28.84 | U | U | U | U | 1.50 | 57.70 |
| Hoff, 2007[141] | 12 | 61.70 | 67% | U | U | U | 26.46 | 0% | U | U | U | 1.08 | 36.20 |
| Engel, 2016[101] | 33 | 65.50 | 30% | U | U | U | U | U | U | U | U | 1.57 | U |
| Kwon, 2018[164] | 85 | 64.35 | 82% | U | U | U | 23.48 | 24% | 53% | 13% | 5% | 1.49 | 57.15 |
| Bustamante, 2010[48] | 18 | 61.44 | 100% | 100% Caucasian | U | U | 26.65 | 0% | 0% | U | U | U | 33.11 |
| Maddocks, 2016[184] | 52 | 69.48 | 40% | U | U | U | 26.79 | 0% | 0% | 44% | 56% | 0.81 | 30.75 |
| Marrara, 2018[196] | 43 | 68.00 | 81% | U | U | U | 23.67 | 0% | 18% | 52% | 29% | 1.05 | 40.08 |
| Klijn, 2013[160] | 110 | 61.00 | 35% | U | U | U | 25.50 | 0% | 0% | 57% | 43% | U | 35.50 |
| Breyer, 2010[43] | 60 | 60.30 | 45% | U | U | U | 26.20 | 0% | 45% | 61% | 32% | U | 46.30 |
| Steiner, 2003[272] | 85 | 67.01 | 62% | U | U | U | 23.70 | U | U | U | U | 0.87 | 34.55 |
| Zanini, 2015[343] | 60 | 70.50 | 82% | U | U | U | 25.50 | U | U | U | U | U | 48.50 |
| Bourne, 2017[40] | 90 | 69.76 | 66% | U | U | U | U | 22% | 43% | 27% | 8% | 1.64 | 58.72 |
| Pavitt, 2018[234] | 122 | 68.93 | 43% | U | U | U | 26.47 | 0% | 48% | 36% | 16% | 1.15 | 50.34 |
| Silva, 2012[264] | 14 | U | U | U | U | U | 25.83 | U | U | U | U | U | U |
| Zanotti, 2011[344] | 20 | 63.85 | 75% | U | U | U | 18.05 | 0% | 0% | 100% | 0% | U | 26.95 |
| Bendstrup, 1997[23] | 32 | 64.50 | 56% | U | U | U | U | 0% | U | U | U | 1.03 | U |
| Gamper, 2019[118] | 16 | 64.30 | 63% | U | U | U | U | 0% | 0% | 63% | 38% | U | U |
| Barakat, 2008[20] | 71 | 64.80 | 84% | U | U | U | 24.90 | U | U | U | U | U | 42.60 |
| Oliveira, 2011[225] | 85 | 69.23 | 76% | U | U | U | 24.46 | 9% | 28% | 34% | 28% | 1.20 | 46.50 |
| Nolan, 2017[219] | 152 | 68.00 | 72% | U | U | U | 28.10 | U | U | U | U | 1.39 | 50.50 |
| Burtin, 2015[46] | 80 | 66.50 | 83% | U | U | U | 25.50 | U | U | U | U | U | 45.50 |
| Petersen, 2008[239] | 19 | 66.00 | 33% | U | U | U | 25.00 | 0% | U | U | U | U | 31.00 |
| Broekhuizen, 2005[44] | 102 | 63.00 | 70% | U | U | U | 22.30 | 0% | U | U | U | U | 37.00 |
| Hawkins, 2002[137] | 19 | 67.05 | 89% | U | U | U | 24.53 | U | U | U | U | 0.78 | 26.95 |
| Pothasak, 2020[242] | 40 | 64.18 | 58% | U | U | U | 21.39 | U | U | U | U | 1.48 | 65.36 |
| Emery, 1998[99] | 79 | 66.60 | 47% | U | U | 28% less than high school education | U | U | U | U | U | 1.14 | 42.00 |
| Dekhuijzen, 1990[84] | 40 | 59.00 | 75% | U | U | U | U | 0% | U | U | U | 1.49 | 49.30 |
| Kayahan, 2006[158] | 45 | 65.82 | 87% | U | U | U | U | 4% | 58% | 38% | 0% | 1.44 | 56.56 |
| Liddell, 2010[173] | 30 | 69.00 | 67% | U | U | U | U | U | U | U | U | U | 51.00 |
| Bianco, 2019[30] | 30 | 70.07 | 93% | U | U | U | 27.98 | U | U | U | U | 1.33 | 51.13 |
| Santos, 2015[257] | 34 | 67.10 | 79% | U | U | 27% Elementary education only | 26.75 | U | U | U | U | 1.50 | 54.90 |
| Zhang, 2016[347] | 130 | 63.46 | 77% | U | U | U | U | 25% | 65% | 10% | 0% | 1.68 | 58.20 |
| Waterhouse, 2010[317] | 240 | 68.92 | 52% | U | U | U | 25.40 | U | U | U | U | 1.10 | 46.82 |
| Bourjeily-Habr, 2002[39] | 18 | 60.00 | 56% | U | U | U | 26.65 | 0% | U | U | U | U | 38.15 |
| Simpson, 1992[267] | 28 | 71.50 | 54% | U | U | U | 24.69 | U | U | U | U | 0.99 | 39.35 |
| Nguyen, 2008[215] | 39 | 69.50 | 56% | U | U | 54% college education or higher. 28% employed | 28.50 | U | U | U | U | U | 49.60 |
| Deacon, 2008[82] | 80 | 67.97 | 63% | U | U | U | 26.84 | U | U | U | U | 1.10 | 44.05 |
| Garrod, 2000[119] | 45 | 64.96 | 62% | U | U | U | U | U | U | U | U | 0.93 | 34.13 |
| Wedzicha, 1998[318] | 110 | 70.51 | 51% | U | U | U | U | 0% | U | U | U | 0.98 | 37.48 |
| Cameron-Tucker, 2016[50] | 65 | 69.00 | 45% | U | U | Mean 10 years of education | 27.00 | 6% | 34% | 37% | 12% | U | U |
| McFarland, 2012[198] | 24 | 74.03 | 54% | U | U | U | U | U | U | U | U | 0.75 | U |
| Ringbaek, 2000[252] | 45 | 63.11 | 16% | U | U | U | 25.17 | U | U | U | U | U | 47.07 |
| Covey, 2014[71] | 75 | 68.00 | 89% | U | U | U | 28.62 | 0% | U | U | U | U | 40.53 |
| Freire, 2022[114] | 48 | 67.75 | 65% | U | U | U | 25.63 | 8% | 44% | 35% | 13% | 1.35 | 48.98 |
| Silva, 2019[266] | 28 | 64.00 | 68% | U | U | U | 27.50 | 0% | 36% | 54% | 11% | U | 48.00 |
| Spruit, 2002[271] | 30 | 63.47 | 87% | U | U | U | 25.07 | U | U | U | U | U | 40.53 |
| Marrara, 2012[195] | 36 | 69.58 | 100% | U | U | U | 23.05 | 0% | 53% | 47% | 0% | 1.20 | 47.21 |
| Griffiths, 2000[129] | 200 | 68.25 | 60% | U | U | U | 25.25 | 0% | U | U | U | 0.90 | 39.55 |
| Hernandez, 2000 | 37 | 63.75 | U | U | U | U | U | 0% | U | U | U | U | 40.92 |
| Creutzberg, 2003[73] | 63 | 66.48 | 100% | U | U | U | 21.54 | 0% | U | U | U | U | 35.62 |
| Mitchell, 2014[202] | 184 | 69.00 | 58% | 98% White British | U | U | 27.55 | 8% | 60% | 23% | 9% | 1.45 | 57.88 |
| Lum, 2007[182] | 75 | 80.83 | 49% | U | U | U | U | U | U | U | U | U | 44.29 |
| Troosters, 2000[295] | 70 | 56.70 | 87% | U | U | U | 24.47 | 0% | U | U | U | U | 41.94 |
| van Wetering, 2010[303] | 199 | 66.53 | 71% | U | U | U | 26.68 | 0% | 69% | 31% | 0% | U | 58.97 |
| Elliott, 2004[96] | 43 | 66.18 | 53% | U | U | U | 26.47 | 0% | U | U | 0% | 0.96 | 45.13 |
| Altenburg, 2014[10] | 61 | 54.00 | 59% | U | U | U | 26.40 | 5% | 31% | 33% | 31% | 1.24 | 43.00 |
| Leung, 2013[171] | 42 | 73.00 | 64% | 100% Caucasian | U | U | 27.40 | U | U | U | U | 1.40 | 59.00 |
| Vogiatzis, 2005[308] | 19 | 65.42 | 84% | U | U | U | 26.37 | 0% | 0% | U | U | 1.10 | 41.63 |
| Constantin, 2013[67] | 59 | 67.98 | 56% | U | U | U | 26.10 | 0% | 0% | U | U | 1.11 | 46.83 |
| Clark, 2000[63] | 43 | 49.00 | 58% | U | U | U | 26.00 | U | U | U | U | 2.33 | 77.00 |
| Vonbank, 2012[309] | 36 | 60.20 | 69% | U | U | U | 27.40 | 11% | 39% | 44% | 6% | 1.80 | 55.80 |
| Hansen, 2020[133] | 134 | 68.30 | 45% | U | U | U | 25.70 | 0% | 0% | 61% | 39% | U | 33.10 |
| Polkey, 2018[241] | 120 | U | U | U | U | U | 20.70 | 0% | U | U | U | 1.21 | 47.90 |
| Tabak, 2014[284] | 24 | 63.45 | 50% | U | U | 30% employed | 26.75 | U | U | U | U | U | 43.00 |
| Ambrosino, 2008[11] | 209 | 67.40 | 84% | U | U | U | 26.60 | 0% | U | U | U | 1.10 | 41.40 |
| Arnardottir, 2006[15] | 42 | 66.57 | 50% | U | U | U | 22.90 | 0% | U | U | U | 1.00 | 37.52 |
| Lake, 1990[166] | 26 | 66.42 | 85% | U | U | U | U | U | U | U | U | 0.88 | U |
| Sutanto, 2019[282] | 20 | 65.35 | 95% | U | U | U | 19.85 | 0% | 65% | 35% | 0% | U | 55.75 |
| Cruz, 2016[74] | 32 | 66.45 | 84% | U | U | U | 29.45 | 38% | 31% | U | U | 1.95 | 66.95 |
| Lin, 2021[174] | 78 | 72.24 | 97% | U | 34% Buddhist, 41% Taoist, 25% Other | 75% unemployed, 35% Elementary school or lower | 24.38 | 27% | 44% | 29% | 0% | U | 64.65 |
| Gloeckl, 2021[126] | 48 | 65.50 | U | U | U | U | 25.65 | 0% | 0% | U | U | U | 37.10 |
| Sharifabad, 2010[261] | 66 | 71.50 | 45% | U | U | U | U | U | U | U | U | 1.03 | U |
| Donesky-Cuenco, 2009[88] | 29 | 69.87 | 28% | 79% Caucasian | U | 13% High school or less | U | U | U | U | U | 1.14 | 47.68 |
| Emtner, 2003[100] | 29 | 66.52 | 62% | U | U | U | 25.29 | 0% | 0% | U | U | 1.01 | 36.55 |
| Reuveny, 2005[249] | 19 | 63.47 | U | U | U | U | U | 0% | 0% | U | U | 0.96 | 32.53 |
| Vasilopoulou, 2017[305] | 147 | 65.88 | 81% | U | U | U | 27.29 | 0% | U | U | U | 1.46 | 51.06 |
| Maltais, 2008[190] | 252 | 66.00 | 56% | U | U | U | 27.50 | 0% | 36% | 50% | 13% | 1.11 | 44.50 |
| Gloeckl, 2017[125] | 74 | 64.00 | 68% | U | U | U | 25.40 | 0% | 0% | U | U | U | 30.10 |
| Peran, 2018[236] | 11 | 63.09 | 55% | U | U | U | 26.15 | 0% | 0% | U | U | U | 33.36 |
| Pan, 2018[228] | 41 | U | 68% | U | U | U | U | U | U | U | 0% | 1.51 | 52.42 |
| Tasdemir, 2015[287] | 27 | 62.51 | 89% | U | U | U | 26.29 | 0% | 33% | 33% | 33% | 1.10 | 36.00 |
| Zhang, 2020[349] | 260 | 64.15 | 87% | U | U | 25% secondary education or above | 21.80 | 14% | 24% | 35% | 27% | U | 53.40 |
| Busch, 1988[47] | 14 | 65.10 | 79% | U | U | U | U | U | U | U | U | 0.77 | 26.25 |
| Chen, 2015[59] | 45 | 59.92 | 62% | U | U | U | U | 0% | 53% | 33% | 13% | 1.79 | 55.66 |
| Zhang, 2012[346] | 90 | 62.54 | 58% | U | U | U | U | U | U | U | U | 1.34 | 51.80 |
| Fichter, 1999[111] | 10 | 58.50 | 100% | U | U | U | 23.35 | U | U | U | U | 1.45 | 43.20 |
| Gadesha, 2015[116] | 36 | 58.06 | 94% | U | U | U | U | 0% | 0% | U | U | U | U |
| Dodia, 2012[86] | 40 | 40.03 | 70% | U | U | U | U | 0% | U | U | 0% | U | U |
| Pancera, 2021[229] | 40 | 69.29 | 69% | U | U | U | 24.01 | 0% | 19% | 39% | 41% | 1.01 | 39.86 |
| McCarroll, 2005[197] | 36 | 72.50 | 46% | U | U | U | 27.31 | 0% | U | U | U | 0.91 | U |
| Vivodtzev, 2012[306] | 20 | 69.20 | 65% | U | U | U | 21.00 | 0% | 0% | U | U | U | 32.40 |
| Lahham, 2020[165] | 58 | 67.50 | 59% | U | U | U | 28.00 | 100% | 0% | 0% | 0% | 2.55 | 91.00 |
| Toledo, 2007[293] | 18 | 67.20 | U | U | U | U | 26.85 | 0% | U | U | U | 0.75 | 33.50 |
| Sykes, 2005[283] | 37 | 73.27 | 84% | U | U | U | 19.37 | 0% | U | U | U | U | 42.00 |
| Boxall, 2005[42] | 46 | 76.70 | 57% | U | U | U | U | U | U | U | U | 0.84 | 39.10 |
| White, 2002[322] | 103 | 67.00 | 69% | U | U | U | U | 0% | 0% | U | U | 0.83 | 26.89 |
| Haugen, 2007[136] | 96 | 60.25 | 64% | U | U | U | 26.91 | 0% | U | U | U | 1.44 | 48.63 |
| Mao, 2018[191] | 40 | 69.85 | 65% | U | U | U | 23.90 | U | U | U | U | 0.86 | 50.05 |
| Kostrzon, 2019[163] | 42 | 63.50 | 57% | U | U | U | 27.04 | 10% | 62% | 24% | 5% | U | 56.07 |
| Holland, 2004[142] | 38 | 67.78 | 63% | U | U | U | 23.49 | 0% | 0% | U | U | 0.99 | 36.56 |
| Effing, 2011[94] | 153 | 63.40 | 58% | U | U | U | 26.45 | 0% | U | U | U | 1.42 | 50.05 |
| Titova, 2008[291] | 39 | 56.38 | 82% | U | U | U | U | 0% | U | U | 0% | 1.31 | 42.12 |
| Wu, 2015[332] | 30 | 60.50 | 100% | U | U | U | 23.22 | 0% | 0% | U | U | U | 38.00 |
| Titova, 2008[292] | 43 | U | U | U | U | U | U | 0% | U | U | U | U | U |
| Liu, 2021[179] | 358 | 64.89 | 56% | U | U | U | 23.84 | 39% | 41% | 20% | 0% | 1.31 | U |
| Miglioire, 2003[201] | 33 | 73.78 | 38% | U | U | 32% less than 9 years education | 25.87 | U | U | U | U | 1.14 | 52.12 |
| O’Hara, 1984[224] | 14 | 57.45 | 79% | U | U | U | U | U | U | U | U | 1.15 | U |
| Wen, 2008[321] | 32 | 67.53 | U | U | U | U | 21.68 | 0% | 44% | 56% | 0% | 1.20 | 48.13 |
| Yekefallah, 2019[340] | 50 | 60.75 | 72% | U | U | 30% illiterate | 25.10 | 0% | U | U | U | U | U |
| Dolmage, 2008[87] | 18 | 62.50 | 44% | U | U | U | 24.24 | 0% | 11% | 17% | 72% | 0.90 | 38.50 |
| Neder, 2002[212] | 15 | 65.96 | 60% | U | U | U | 25.12 | 0% | 0% | U | U | 0.95 | 38.60 |
| Du, 2013[91] | 112 | 64.01 | 63% | U | U | U | 22.91 | 29% | 71% | 0% | 0% | U | 73.54 |
| Casaburi, 1991[53] | 19 | 51.11 | 100% | U | U | U | 25.14 | 0% | U | U | U | 1.82 | 56.00 |
| Cox, 2022[72] | 96 | 67.50 | 46% | U | U | U | 28.00 | U | U | U | U | 1.55 | 60.99 |
| Engstrom, 1999[102] | 50 | 66.42 | 52% | U | U | U | 22.96 | 0% | 0% | U | U | U | 32.47 |
| Alsharaway, 2021[9] | 35 | 66.76 | 80% | U | U | U | 24.26 | U | U | U | U | U | 31.84 |
| Charususin, 2021[58] | 14 | 67.50 | 71% | U | U | U | 23.40 | U | U | U | U | U | 56.40 |
| 陆绍勇, 2022[351] | 60 | 63.36 | 65% | U | U | U | 23.85 | U | U | U | U | 2.17 | U |
| de Lima, 2022[79] | 51 | 68.35 | 49% | U | U | U | 25.93 | U | U | U | U | 1.26 | 53.72 |
| Neunhauserer, 2023[213] | 28 | 63.60 | 75% | U | U | U | 26.90 | 0% | U | U | 0% | U | 45.90 |
| Tabka, 2023[285] | 39 | 65.25 | 100% | 100% Tunisian | U | Mean 9 years of education | 23.84 | U | U | U | U | 1.35 | 45.24 |
| Finnegan, 2023[112] | 71 | 71.24 | 75% | U | U | U | 27.11 | U | U | U | U | U | 58.93 |
| Xu, 2023[338] | 82 | U | U | U | U | U | U | U | U | U | U | U | U |
| Liu, 2023[180] | 102 | 63.74 | 67% | U | U | U | 23.61 | 0% | U | U | 0% | 1.48 | 54.30 |
| Peran, 2022[237] | 100 | 65.25 | 61% | U | U | U | 25.10 | U | U | U | U | 0.78 | 30.16 |
| Deniz, 2023[85] | 35 | 43.36 | 100% | U | U | U | 15.87 | U | U | U | U | 0.73 | 24.87 |
| Duan, 2022[92] | 90 | 72.47 | U | 100% Asian | U | U | 21.88 | U | U | U | U | U | 42.12 |
| Elmorshidy, 2023[97] | 75 | 41.33 | 100% | U | U | U | 14.53 | U | U | U | U | U | 23.65 |
| Ma, 2022[183] | 72 | 69.58 | 57% | U | U | 60% secondary school or above | U | U | U | U | U | U | U |
| Maglakelidze, 2022[188] | 38 | 65.00 | 90% | 93% Georgian | U | 70% High school education or higher. 33% in work | U | 22% | 62% | 13% | 3% | U | U |
| Mohamed, 2022[206] | 40 | 59.85 | 100% | U | U | U | 26.33 | U | U | U | U | 1.08 | 63.55 |
| Kaya, 2023[157] | 24 | 64.96 | 83% | U | U | U | 25.54 | U | U | U | U | U | 43.25 |
| Kohlbrenner, 2023[161] | 30 | 64.00 | 57% | U | U | U | U | 3% | 30% | 47% | 20% | 1.40 | 47.00 |
| Pancera, 2023[230] | 30 | 68.30 | 50% | U | U | U | 23.03 | 0% | 23% | 47% | 30% | U | 44.37 |
| Shui, 2023[262] | 37 | 70.34 | 84% | U | U | U | 21.14 | U | U | U | U | U | 41.35 |
| Wang, 2023[315] | 60 | 42.54 | 78% | U | U | U | U | U | U | U | U | U | U |
| Whitsel, 1998[323] | 8 | 65.80 | 38% | U | U | U | 26.20 | U | U | U | U | U | U |
|  | 38 | 66 | 61% | U | U | U | 25.53 | U | U | U | U | 1.53 | 57.00 |
| McKeough, 2012[199] | 29 | 52 | 59% | U | U | U | 27.50 | 0% | 44% | 55% | 0% | U | U |
| Abedi Yekta, 2019[3] | 38 | 63 | 84% | U | U | U | 25.47 | U | U | U | U | 1.61 | 55.05 |
| Ninot, 2011[217] | 36 | 68 | 100% | U | U | U | 24.55 | U | U | U | U | 1.20 | 46.30 |
| Arslan, 2016[19] | 65 | 57 | 26% | U | U | U | U | 0% | U | U | 0% | U | U |
| Bhasin, 2012[28] | 40 | U | U | U | U | U | U | U | U | U | U | U | U |
| Benzo, 2021[24] | 146 | 69 | 49% | U | U | 51% post secondary education | U | U | U | U | U | U | 42.84 |
| Schultz, 2018[258] | 602 | 58 | 65% | U | U | U | 26.75 | 0% | 49% | 40% | 11% | 1.52 | 50.25 |
| Geidl, 2021[121] | 327 | 58 | 69% | U | U | U | 27.59 | 9% | 45% | 35% | 9% | U | 53.51 |
| Farver-Vestergaard, 2018[107] | 84 | 67 | 43% | U | U | 75% less than 2 years of further education. 90% retired. 5% employed | 25.75 | 0% | 0% | U | U | U | 27.74 |
| Salve, 2016[256] | 94 | 59 | 86% | U | U | U | 18.96 | 18% | 11% | 65% | 13% | U | U |
| Paz-Diaz, 2007[235] | 19 | 67 | 89% | U | U | U | 24.53 | U | U | U | U | 0.78 | 26.95 |
| Beauchamp, 2013[21] | 24 | 64 | 75% | U | U | U | 22.89 | U | U | U | U | 0.79 | 31.67 |
| Janaudis-Ferreira, 2011[149] | 39 | 70 | 38% | U | U | U | 25.68 | U | U | U | U | U | 37.82 |
| Fanfa Bordin, 2020[105] | 36 | 67 | 58% | U | U | U | 26.74 | 0% | U | U | U | 0.80 | 35.00 |
| Covey, 2012[70] | 20 | 65 | 55% | U | U | U | 25.35 | 0% | U | U | U | U | 38.50 |
| Kongsgaard, 2004[162] | 64 | 71 | 81% | U | U | U | 28.14 | 0% | 50% | 39% | 11% | U | 57.80 |
| Pereira, 2010[238] | 13 | 72 | 100% | U | U | 55% secondary school level | 25.49 | U | U | U | U | 1.48 | 57.85 |
|  | 50 | 64 | 100% | U | U | U | 26.50 | 0% | 66% | 34% | 0% | 1.31 | 51.95 |
| Wittmann, 2007[326] | 184 | 54 | 80% | U | U | U | U | U | U | U | U | 1.82 | U |
| Gayle, 1988[120] | 15 | U | U | U | U | U | U | U | U | U | U | U | U |
| Majewska-Pulsakowska, 2016[189] | 35 | 63 | 66% | U | U | U | 28.27 | 0% | U | U | 0% | 1.67 | 61.18 |
| de Blok, 2006[77] | 16 | 64 | 43% | U | U | U | 28.75 | 5% | 38% | 39% | 19% | 1.34 | 47.50 |
| Benzo, 2022[25] | 375 | 69 | 43% | 97% "Not Hispanic or Latino" | U | 32% had income of $30,000 or less | 28.84 | U | U | U | U | U | 44.69 |
| Bourne, 2022[41] | 193 | 70 | 54% | 90.7% White | U | 71% Retired | 27.50 | 20% | 57% | 16% | 3% | 1.70 | 66.92 |
| Zhuang, 2023[350] | 36 | 66 | 81% | U | U | 47% education < 12 years | 23.34 | 0% | 17% | 36% | 47% | U | U |

###

### **Supplementary table 17 –** Description of individual components in included studies

| Component | Interim model | Final model | Frequency (non-optional) | Frequency (optional) | Total frequency |
| --- | --- | --- | --- | --- | --- |
| Usual care |  |  | 92 | 0 | 92 |
| Supervised very high Intensity continuous walking | Exercise | Very high intensity aerobic exercise | 9 | 0 | 9 |
| Unsupervised very high intensity continuous walking | Exercise | Very high intensity aerobic exercise | 4 | 0 | 4 |
| Supervised very high intensity continuous cycling | Exercise | Very high intensity aerobic exercise | 20 | 0 | 20 |
| Supervised very high intensity interval walking | Exercise | Very high intensity aerobic exercise | 1 | 0 | 1 |
| Supervised very high intensity interval cycling | Exercise | Very high intensity aerobic exercise | 12 | 0 | 12 |
| Supervised high intensity continuous walking | Exercise | High intensity aerobic exercise | 68 | 8 | 76 |
| Supervised high intensity interval walking | Exercise | High intensity aerobic exercise | 1 | 2 | 3 |
| Unsupervised high intensity continuous walking | Exercise | High intensity aerobic exercise | 26 | 1 | 27 |
| Supervised high intensity continuous cycling | Exercise | High intensity aerobic exercise | 91 | 8 | 99 |
| Supervised high intensity interval cycling | Exercise | High intensity aerobic exercise | 10 | 2 | 12 |
| Unsupervised high Intensity continuous cycling | Exercise | High intensity aerobic exercise | 3 | 1 | 4 |
| Unsupervised high intensity interval cycling | Exercise | High intensity aerobic exercise | 0 | 1 | 1 |
| Unsupervised high intensity interval walking | Exercise | High intensity aerobic exercise | 0 | 1 | 1 |
| Stair climbing, high intensity | Exercise | High intensity aerobic exercise | 7 | 2 | 9 |
| Supervised circuit training/callisthenics, high intensity | Exercise | High intensity aerobic exercise | 5 | 0 | 5 |
| Unsupervised circuit training/callisthenics, high intensity | Exercise | High intensity aerobic exercise | 2 | 0 | 2 |
| Supervised high intensity aerobic exercise, not otherwise specified | Exercise | High intensity aerobic exercise | 2 | 0 | 2 |
| Supervised high intensity cycling in water | Exercise | High intensity aerobic exercise | 1 | 0 | 1 |
| Creative dance, high intensity | Exercise | High intensity aerobic exercise | 1 | 0 | 1 |
| Supervised rowing, high intensity | Exercise | High intensity aerobic exercise | 1 | 2 | 3 |
| Water callisthenics, high intensity | Exercise | High intensity aerobic exercise | 3 | 0 | 3 |
| Supervised moderate intensity continuous walking | Exercise | Moderate intensity aerobic exercise | 55 | 0 | 55 |
| Supervised moderate intensity interval walking | Exercise | Moderate intensity aerobic exercise | 2 | 0 | 2 |
| Unsupervised moderate intensity continuous walking | Exercise | Moderate intensity aerobic exercise | 17 | 3 | 20 |
| Supervised moderate intensity continuous cycling | Exercise | Moderate intensity aerobic exercise | 54 | 0 | 54 |
| Supervised moderate intensity interval cycling | Exercise | Moderate intensity aerobic exercise | 4 | 0 | 4 |
| Unsupervised moderate intensity continuous cycling | Exercise | Moderate intensity aerobic exercise | 1 | 5 | 6 |
| Unsupervised moderate intensity interval cycling | Exercise | Moderate intensity aerobic exercise | 4 | 0 | 4 |
| Stair climbing, moderate intensity | Exercise | Moderate intensity aerobic exercise | 1 | 1 | 2 |
| Supervised circuit training/callisthenics, moderate intensity | Exercise | Moderate intensity aerobic exercise | 3 | 0 | 3 |
| Unsupervised circuit training/callisthenics, moderate intensity | Exercise | Moderate intensity aerobic exercise | 3 | 0 | 3 |
| Supervised continuous moderate intensity cycling in water | Exercise | Moderate intensity aerobic exercise | 1 | 0 | 1 |
| Supervised continuous moderate intensity walking in water | Exercise | Moderate intensity aerobic exercise | 1 | 0 | 1 |
| Supervised moderate intensity aerobic exercise, not otherwise specified | Exercise | Moderate intensity aerobic exercise | 6 | 0 | 6 |
| Unsupervised moderate aerobic exercise, not otherwise specified | Exercise | Moderate intensity aerobic exercise | 6 | 0 | 6 |
| Supervised low intensity walking | Exercise | Low intensity aerobic exercise | 41 | 1 | 42 |
| Unsupervised low intensity walking | Exercise | Low intensity aerobic exercise | 24 | 0 | 24 |
| Supervised low intensity cycling | Exercise | Low intensity aerobic exercise | 18 | 1 | 19 |
| Stair climbing, low intensity | Exercise | Low intensity aerobic exercise | 7 | 0 | 7 |
| Supervised rowing, low intensity | Exercise | Low intensity aerobic exercise | 3 | 0 | 3 |
| Supervised circuit training/callisthenics, low intensity | Exercise | Low intensity aerobic exercise | 7 | 0 | 7 |
| Unsupervised circuit training/callisthenics, low intensity | Exercise | Low intensity aerobic exercise | 3 | 0 | 3 |
| Supervised chair exercises | Exercise | Low intensity aerobic exercise | 2 | 0 | 2 |
| Supervised low intensity walking in water | Exercise | Low intensity aerobic exercise | 1 | 0 | 1 |
| Unsupervised low intensity aerobic exercise, not otherwise specified | Exercise | Low intensity aerobic exercise | 1 | 0 | 1 |
| Supervised continuous cycling, intensity not specified | Exercise | Aerobic training, intensity not specified or not prescribed | 39 | 11 | 50 |
| Supervised interval cycling, intensity not specified | Exercise | Aerobic training, intensity not specified or not prescribed | 0 | 2 | 2 |
| Supervised continuous walking, intensity not specified | Exercise | Aerobic training, intensity not specified or not prescribed | 44 | 6 | 50 |
| Supervised interval walking, intensity not specified | Exercise | Aerobic training, intensity not specified or not prescribed | 4 | 0 | 4 |
| Unsupervised continuous walking, intensity not specified | Exercise | Aerobic training, intensity not specified or not prescribed | 15 | 0 | 15 |
| Supervised circuit training/callisthenics, intensity not specified or not prescribed | Exercise | Aerobic training, intensity not specified or not prescribed | 43 | 0 | 43 |
| Unsupervised circuit training/callisthenics, intensity not specified or not prescribed | Exercise | Aerobic training, intensity not specified or not prescribed | 6 | 0 | 6 |
| Stair climbing, intensity not specified or not prescribed | Exercise | Aerobic training, intensity not specified or not prescribed | 21 | 2 | 23 |
| Supervised aerobic exercise, not otherwise specified | Exercise | Aerobic training, intensity not specified or not prescribed | 7 | 0 | 7 |
| Water based callisthenics, intensity not specified | Exercise | Aerobic training, intensity not specified or not prescribed | 2 | 0 | 2 |
| Skipping, intensity not specified | Exercise | Aerobic training, intensity not specified or not prescribed | 1 | 0 | 1 |
| Unsupervised continuous cycling, intensity not specified | Exercise | Aerobic training, intensity not specified or not prescribed | 2 | 1 | 3 |
| Supervised rowing, intensity not specified | Exercise | Aerobic training, intensity not specified or not prescribed | 3 | 0 | 3 |
| Swimming, intensity not specified | Exercise | Aerobic training, intensity not specified or not prescribed | 8 | 4 | 12 |
| Unsupervised backpack wearing, low intensity | Exercise | Aerobic training, intensity not specified or not prescribed | 2 | 0 | 2 |
| Video game directed exercises; intensity not prescribed | Exercise | Aerobic training, intensity not specified or not prescribed | 1 | 0 | 1 |
| Unsupervised aerobic exercise, not otherwise specified | Exercise | Aerobic training, intensity not specified or not prescribed | 13 | 0 | 13 |
| Supervised lower limb strength training, low intensity | Exercise | Lower limb strength training | 12 | 0 | 12 |
| Supervised lower limb strength training, moderate intensity | Exercise | Lower limb strength training | 87 | 0 | 87 |
| Supervised lower limb strength training, high intensity | Exercise | Lower limb strength training | 4 | 0 | 4 |
| Supervised lower limb strength training, intensity not specified or prescribed | Exercise | Lower limb strength training | 57 | 0 | 57 |
| Unsupervised lower limb strength training, moderate intensity | Exercise | Lower limb strength training | 6 | 0 | 6 |
| Unsupervised lower limb strength training, intensity not specified or prescribed | Exercise | Lower limb strength training | 13 | 0 | 13 |
| Lower limb resistance bands | Exercise | Lower limb strength training | 7 | 0 | 7 |
| Supervised lower limb strength training in water, moderate intensity | Exercise | Lower limb strength training | 1 | 0 | 1 |
| Supervised lower limb strength training in water, intensity not specified | Exercise | Lower limb strength training | 1 | 0 | 1 |
| Unsupervised lower limb resistance bands | Exercise | Lower limb strength training | 5 | 0 | 5 |
| Supervised lower limb resistance bands - 1 leg, moderate | Exercise | Lower limb strength training | 1 | 0 | 1 |
| Supervised lower limb resistance tubes (not bands) | Exercise | Lower limb strength training | 2 | 0 | 2 |
| Supervised upper limb strength training, moderate intensity | Exercise | Upper limb strength training | 95 | 0 | 95 |
| Supervised upper limb strength training, high intensity | Exercise | Upper limb strength training | 2 | 0 | 2 |
| Supervised upper limb strength training, low intensity | Exercise | Upper limb strength training | 14 | 0 | 14 |
| Supervised upper limb strength training, intensity not specified or prescribed | Exercise | Upper limb strength training | 93 | 2 | 95 |
| Unsupervised upper limb strength training, moderate | Exercise | Upper limb strength training | 6 | 0 | 6 |
| Unsupervised upper limb strength training, intensity not specified or prescribed | Exercise | Upper limb strength training | 14 | 0 | 14 |
| Upper limb resistance bands | Exercise | Upper limb strength training | 8 | 0 | 8 |
| Supervised upper limb strength training in water, moderate intensity | Exercise | Upper limb strength training | 1 | 0 | 1 |
| Unsupervised upper limb resistance bands | Exercise | Upper limb strength training | 6 | 0 | 6 |
| Supervised upper limb resistance bands - 1 arm, moderate intensity | Exercise | Upper limb strength training | 1 | 0 | 1 |
| Supervised upper limb resistance tubes (not bands) | Exercise | Upper limb strength training | 2 | 0 | 2 |
| Supervised upper limb strength training in water, intensity not specified | Exercise | Upper limb strength training | 2 | 0 | 1 |
| Unsupervised trunk strength training | Exercise | Core strength training | 1 | 0 | 1 |
| Supervised trunk strength training | Exercise | Core strength training | 19 | 0 | 19 |
| Supervised trunk strength training in water | Exercise | Core strength training | 1 | 0 | 1 |
| Supervised strength training, limb not specified, moderate intensity | Exercise | Strength training (limb not specified) | 6 | 0 | 6 |
| Supervised strength training, limb not specified, high intensity | Exercise | Strength training (limb not specified) | 2 | 0 | 2 |
| Unsupervised moderate strength training, limb not specified | Exercise | Strength training (limb not specified) | 1 | 0 | 1 |
| Unsupervised strength training (intensity and limb not specified) | Exercise | Strength training (limb not specified) | 6 | 0 | 6 |
| Supervised strength training, not otherwise specified | Exercise | Strength training (limb not specified) | 34 | 0 | 34 |
| Resistance bands, muscle group not specified | Exercise | Strength training (limb not specified) | 5 | 0 | 3 |
| One leg continuous cycling | Exercise | Partition training | 1 | 0 | 1 |
| One leg interval cycling | Exercise | Partition training | 4 | 0 | 4 |
| Arm cycling | Exercise | Arm cycling | 30 | 4 | 34 |
| Balance training | Exercise | Balance training | 14 | 0 | 14 |
| Supervised whole body vibration | Exercise | Whole body vibration | 10 | 0 | 10 |
| Supervised flexibility training | Exercise | Flexibility training | 37 | 0 | 37 |
| Unsupervised flexibility training | Exercise | Flexibility training | 6 | 0 | 6 |
| Supervised Nordic walking | Exercise | Nordic walking | 4 | 0 | 4 |
| Unsupervised Nordic walking | Exercise | Nordic walking | 1 | 0 | 1 |
| Periodisation of training | Exercise | Periodisation of training | 1 | 0 | 1 |
| High frequency neuroelectrical stimulation of quadriceps | Exercise | Non-volitional exercise | 12 | 0 | 12 |
| Low frequency neuroelectrical stimulation of quadriceps | Exercise | Non-volitional exercise | 1 | 0 | 1 |
| Repetitive magnetic stimulation | Exercise | Non-volitional exercise | 1 | 0 | 1 |
| Neuroelectrical stimulation of quadriceps, intensity not specified | Exercise | Non-volitional exercise | 0 | 3 | 3 |
| Neuroelectrical stimulation of diaphragm | Exercise | Non-volitional exercise | 0 | 3 | 3 |
| High frequency neuroelectrical stimulation of triceps | Exercise | Non-volitional exercise | 1 | 0 | 1 |
| Neuromuscular electrical stimulation, not otherwise specified | Exercise | Non-volitional exercise | 0 | 2 | 2 |
| Eccentric strength training of the legs | Exercise | Eccentric exercise | 1 | 0 | 1 |
| Eccentric cycling | Exercise | Eccentric exercise | 3 | 0 | 3 |
| Downhill walking | Exercise | Eccentric exercise | 3 | 0 | 3 |
| Supervised Qigong | Exercise | Asian and Chinese exercise training | 3 | 0 | 3 |
| Daoyin exercise | Exercise | Asian and Chinese exercise training | 1 | 0 | 1 |
| Liuzijue exercise | Exercise | Asian and Chinese exercise training | 4 | 0 | 4 |
| Unsupervised daoyin exercise | Exercise | Asian and Chinese exercise training | 2 | 0 | 2 |
| Unsupervised Liuzijue exercise | Exercise | Asian and Chinese exercise training | 4 | 0 | 4 |
| Unsupervised Taichi | Exercise | Asian and Chinese exercise training | 9 | 0 | 9 |
| Unsupervised yoga | Exercise | Asian and Chinese exercise training | 2 | 0 | 2 |
| Unsupervised Qigong | Exercise | Asian and Chinese exercise training | 1 | 0 | 1 |
| Tai Chi | Exercise | Asian and Chinese exercise training | 12 | 0 | 12 |
| Yoga | Exercise | Asian and Chinese exercise training | 2 | 0 | 2 |
| Water based Liuzijue | Exercise | Asian and Chinese exercise training | 1 | 0 | 1 |
| Supervised exercise, not otherwise specified | Exercise | Exercise, not otherwise specified | 10 | 0 | 10 |
| Unsupervised exercise, not specified | Exercise | Exercise, not otherwise specified | 17 | 0 | 17 |
| Sham chest vibration | Placebo | Placebo | 1 | 0 | 1 |
| Writing about an emotionally neutral subject (sham) | Placebo | Placebo | 1 | 0 | 1 |
| Sham NMES | Placebo | Placebo | 5 | 0 | 5 |
| Supplemental air | Placebo | Placebo | 6 | 0 | 6 |
| Sham flexibility training | Placebo | Placebo | 2 | 0 | 2 |
| Sham IMT | Placebo | Placebo | 2 | 0 | 2 |
| Soft manipulation (sham osteopathy treatment) | Placebo | Placebo | 3 | 0 | 3 |
| Placebo | Placebo | Placebo | 20 | 0 | 20 |
| Dietary counselling | Nutritional intervention | Nutritional intervention | 52 | 17 | 69 |
| Whey protein | Nutritional intervention | Nutritional intervention | 1 | 0 | 1 |
| BCAA supplementation | Nutritional intervention | Nutritional intervention | 1 | 0 | 1 |
| High protein supplement | Nutritional intervention | Nutritional intervention | 3 | 0 | 3 |
| Macronutrient supplement drink | Nutritional intervention | Nutritional intervention | 1 | 0 | 1 |
| High calorie drink | Nutritional intervention | Nutritional intervention | 1 | 0 | 1 |
| Smoking cessation | Smoking cessation | Smoking cessation | 23 | 5 | 28 |
| Supervised education | Education | Education | 278 | 2 | 280 |
| Unsupervised education | Education | Education | 20 | 0 | 20 |
| Education, unclear if unsupervised | Education | Education | 3 | 0 | 3 |
| Behavioural intervention | Psychological intervention | Psychological intervention | 4 | 0 | 4 |
| Cognitive Behavioural Therapy | Psychological intervention | Psychological intervention | 0 | 4 | 4 |
| Physical activity behaviour modification intervention | Psychological intervention | Psychological intervention | 14 | 0 | 14 |
| Psychological counselling | Psychological intervention | Psychological intervention | 52 | 22 | 74 |
| Family involvement | Psychological intervention | Psychological intervention | 1 | 0 | 1 |
| Nurse coaching | Psychological intervention | Psychological intervention | 2 | 0 | 2 |
| Self-efficacy intervention | Psychological intervention | Psychological intervention | 1 | 0 | 1 |
| Written disclosure therapy | Psychological intervention | Psychological intervention | 1 | 0 | 1 |
| Occupational therapy | Occupational therapy | Occupational therapy | 9 | 8 | 17 |
| Exercise performed underground | Exercise performed underground | Exercise performed underground | 1 | 0 | 1 |
| Mindfulness | Mindfulness | Mindfulness | 2 | 0 | 2 |
| Reflexology | Reflexology | Reflexology | 1 | 0 | 1 |
| Cognitive training | Cognitive training | Cognitive training | 1 | 0 | 1 |
| Supervised breathing exercises/ retraining | Breathing training | Breathing training | 98 | 2 | 100 |
| Unsupervised breathing exercises/ retraining | Breathing training | Breathing training | 16 | 0 | 16 |
| Ventilation feedback training | Breathing training | Breathing training | 3 | 0 | 3 |
| Hyperpnea training | Breathing training | Breathing training | 1 | 0 | 1 |
| Harmonica playing | Breathing training | Breathing training | 1 | 0 | 1 |
| Nasal inspiratory restriction device | Breathing training | Breathing training | 1 | 0 | 1 |
| Inspiratory muscle training | Breathing training | Inspiratory muscle training | 35 | 5 | 40 |
| Supplemental oxygen (targeted to maintain saturations) | Oxygen | Oxygen | 6 | 40 | 46 |
| Supplemental oxygen (hyperoxia) | Oxygen | Oxygen | 13 | 0 | 13 |
| NIV during exercise | NIV | NIV | 9 | 0 | 9 |
| Domiciliary NIV | NIV | NIV | 1 | 0 | 1 |
| Vibratory PEP (Acapella) | Airway clearance | Airway clearance | 1 | 0 | 1 |
| Airway clearance techniques (Bubble PEP) | Airway clearance | Airway clearance | 2 | 0 | 2 |
| Generic chest physio/ airway clearance | Airway clearance | Airway clearance | 39 | 12 | 51 |
| Chest wall vibration during exercise | Airway clearance | Airway clearance | 2 | 0 | 2 |
| Acupuncture | Acupuncture | Acupuncture | 1 | 0 | 1 |
| Heliox | Heliox | Heliox | 4 | 0 | 4 |
| Music during exercise | Music | Music | 5 | 0 | 5 |
| Osteopathic manipulation | Osteopathic manipulation | Osteopathic manipulation | 2 | 0 | 2 |
| Diaphragm pacing | Diaphragm pacing | Diaphragm pacing | 1 | 0 | 1 |
| Blood flow restriction | Blood flow restriction | Blood flow restriction | 1 | 0 | 1 |
| Antioxidants | Antioxidants | Antioxidants | 1 | 0 | 1 |
| B12 | B12 | B12 | 1 | 0 | 1 |
| ACE inhibitor | ACE inhibitor | ACE inhibitor | 1 | 0 | 1 |
| Creatine | Creatine | Creatine | 3 | 0 | 3 |
| Normal saline | Normal saline | Normal saline | 1 | 4 | 5 |
| Hypertonic saline | Hypertonic saline | Hypertonic saline | 1 | 0 | 1 |
| Irbesartan | Irbesartan | Irbesartan | 1 | 0 | 1 |
| Tiotropium | Tiotropium | Tiotropium | 5 | 0 | 5 |
| Short acting beta agonist | Short acting beta agonist | Short acting beta agonist | 1 | 0 | 1 |
| L-carnitine | L-carnitine | L-carnitine | 4 | 0 | 4 |
| N-acetylcysteine | N-acetylcysteine | N-acetylcysteine | 1 | 0 | 1 |
| Beetroot juice | Beetroot juice | Beetroot juice | 1 | 0 | 1 |
| Polyunsaturated fatty acids | Polyunsaturated fatty acids | Polyunsaturated fatty acids | 1 | 0 | 1 |
| Star fruit-honey Product | Star fruit-honey Product | Star fruit-honey Product | 1 | 0 | 1 |
| Anabolic steroids | Anabolic steroids | Anabolic steroids | 1 | 0 | 1 |
| Almitrine | Almitrine | Almitrine | 1 | 0 | 1 |
| D-cycloserine | D-cycloserine | D-cycloserine | 1 | 0 | 1 |

### **Supplementary figure 84–** PRISMA flow chart for updated search conducted in May 2025 prior to publication

Studies from this updated search not included in the analysis.

### **Supplementary table 18 –** Included studies in updated review May 2025 (not included in analysis)

| He[355] | 2025 | Comparing penetrating needles and non-penetrating needles with electrical stimulation combined with exercise training for relief of dyspnea and improving exercise tolerance in chronic obstructive pulmonary disease patients: A single-blind randomized controlled trial. |
| --- | --- | --- |
| Zhu[356] | 2025 | Effects of Mawangdui exercise intervention on the pulmonary function, physical fitness and quality of life in stable chronic obstructive pulmonary disease patients: A randomised controlled trial. |
| Jin[357] | 2025 | Effect of neuromuscular electrical stimulation combined with respiratory rehabilitation training on pulmonary rehabilitation in patients with chronic obstructive pulmonary disease. |
| Phantayuth[358] | 2024 | Effectiveness of a 12-week combining tai chi and yoga program on pulmonary function and functional fitness in COPD patients. |
| Chen[359] | 2024 | Effect of Baduanjin exercise on health and functional status in patients with chronic obstructive pulmonary disease: a community-based, cluster-randomized controlled trial. |
| Cui[360] | 2024 | Effects and long-term outcomes of endurance versus resistance training as an adjunct to standard medication in patients with stable COPD: a multicenter randomized trial. |
| Viana[361] | 2023 | Melatonin supplementation enhances pulmonary rehabilitation outcomes in COPD: a randomized, double-blind, placebo-controlled study. |
| Wang[362] | 2025 | Effectiveness of high-intensity interval training in rehabilitation nursing for mild-to-moderate stable COPD patients: a randomized controlled clinical trial. |
| Nolasco[363] | 2025 | Combined Physical Exercise in Pulmonary Rehabilitation Does Not Alter Endothelial Function and Vascular Structure in Chronic Obstructive Pulmonary Disease: A Randomized Clinical Trial. |
| Engel[364] | 2024 | The Long-Term Benefit of Exercise With and Without Manual Therapy for Mild Chronic Obstructive Pulmonary Disease: A Randomized Controlled Trial. |
| Jiang[365] | 2023 | Effects of pulmonary-based Qigong exercise in stable patients with chronic obstructive pulmonary disease: a randomized controlled trial. |
| Ito[366] | 2025 | Effect of exercise training on modulating the TH17/TREG imbalance in individuals with severe COPD: a randomized controlled trial |
| Sevasta[367] | 2025 | Short term effects of MET programme in patients diagnosed with Chronic Obstructive Pulmonary Disease: a randomised controlled trial. |
| Gallo-Silva[368] | 2024 | Effect of water exercise on the respiratory function and functional capacity of patients with COPD: a randomized controlled trial. |
| Zong[369] | 2023 | Effects of whey protein complex combined with low-intensity exercise in elderly inpatients with COPD at a stable stage |
| Williams[370] | 2023 | Pulmonary Rehabilitation with and without a Cognitive Behavioral Intervention for Breathlessness in People Living with Chronic Obstructive Pulmonary Disease: randomized Controlled Trial |
| Lee[371] | 2024 | Participant-selected music listening during pulmonary rehabilitation in people with chronic obstructive pulmonary disease: a randomised controlled trial |
| da Silva[372] | 2024 | Responders COPD patients to two different home-based rehabilitation programs: a blind, randomized, and controlled clinical trial |
| Chen[373] | 2025 | Combined Effect of 12 Weeks Baduanjin and Tri-Ball Respiratory Training as a Home-Based Pulmonary Rehabilitation in Subjects With Moderate Chronic Obstructive Pulmonary Disease: A Multi-Center Randomized Controlled Trial. |
| Harvey-Dunstan[374] | 2025 | The Responsiveness of Exercise Tests in COPD: a Randomized Controlled Trial |
| Atac[375] | 2024 | The Impact of Different Telerehabilitation Methods on Peripheral Muscle Strength and Aerobic Capacity in COPD Patients: a Randomized Controlled Trial |
| Karaca[376] | 2024 | Effects of body awareness therapy on balance and fear of falling in patients with chronic obstructive pulmonary disease: a randomized controlled trial. |
| Tsui[377] | 2025 | Benefits of chest wall mobilization on respiratory efficiency and functional exercise capacity in people with severe chronic obstructive pulmonary disease (COPD): A randomized controlled trial. |
| Bishop[378] | 2025 | Effect of pulmonary rehabilitation duration on exercise capacity and health-related quality of life in people with chronic obstructive pulmonary disease (PuRe Duration Trial): A randomized controlled equivalence trial. |
| Kohlbrenner[379] | 2024 | Hybrid Virtual Coaching and Telemonitoring in COPD Management: The CAir Randomised Controlled Study. |
| Jiang[380] | 2024 | Effectiveness of patient decision aid supported shared decision-making intervention in in-person and virtual hybrid pulmonary rehabilitation in older adults with chronic obstructive pulmonary disease: A pilot randomized controlled trial. |
| Kochamat[381] | 2024 | The effects of a symptom management program on symptom experience and physical function in Thai adults with chronic obstructive pulmonary disease: A single-blind randomized controlled trial study. |
| Gloeckl[382] | 2025 | Smartphone application-based pulmonary rehabilitation in COPD: a multicentre randomised controlled trial. |
| Zhang[383] | 2024 | Efficacy of online pulmonary rehabilitation management among community dwelling patients with stable chronic obstructive pulmonary disease |
| Rath[384] | 2025 | Optimizing pulmonary rehabilitation: the role of structured exercise interventions in enhancing functional and physiological outcomes in chronic obstructive pulmonary disease |
| Na[385] | 2023 | Influences of aerobic exercise associated with the pharmacological treatment of COPD |
| Thokchom[386] | 2021 | A Clinical Study to Evaluate the Effects of Yogic Intervention on Pulmonary Functions, Inflammatory Marker and Health Status in Patients of Chronic Obstructive Pulmonary Disease |
| Dua[387] | 2024 | Effectiveness of Telemedicine Interventions in Chronic Obstructive Pulmonary Disease (COPD) Management: A Randomized Controlled Trial Comparing Yoga Therapy and Pulmonary Rehabilitation Over Three Months. |
| Zhang[388] | 2025 | Research on the effect of home-based pulmonary rehabilitation in patients with moderate chronic obstructive pulmonary disease |
| Wu[389] | 2024 | Efficacy of remote home-monitored Baduanjin in patients with chronic obstructive pulmonary disease |

#

# Example WinBugs models

### Additive model

# Within groups standard deviation

# s_within = ( ( ((n1-1)*sd1^2) + ((n2-1)*sd2^2) ) / (n1+n2-2) )^0.5

model{

for(i in 1:Ntrials){ # LOOP THROUGH STUDIES

w[i,1] <- 0 # adjustment for multi-arm trials is zero for control arm

delta[i,1] <- 0 # treatment effect is zero for control arm

mu[i] ~ dnorm(0,.0001) # vague priors for all trial baselines

for (k in 1:na[i]){

prec[i,k] <- n[i,k]/pow(sd[i,k],2) # set precisions

var[i,k] <- 1/prec[i,k] # set variances

y[i,k] ~ dnorm(phi[i,k], prec[i,k]) # normal likelihood

phi[i,k] <- theta[i,k]*s_within[i] # theta is standardised mean

theta[i,k] <- mu[i] + delta[i,k] # model for linear predictor, delta is SMD

# deviance contribution

dev[i,k] <- (y[i,k]-phi[i,k])*(y[i,k]-phi[i,k])/var[i,k]

}

# summed residual deviance contribution for this trial

resdev[i] <- sum(dev[i,1:na[i]])

for (k in 2:na[i]){ # LOOP THROUGH ARMS

# trial-specific RE distributions

delta[i,k] ~ dnorm(md[i,k], taud[i,k])

# mean of treatment effect distributions, with multi-arm trial correction

A[i,k] <- d[2]*c1[i, k] + d[3]*c2[i, k] + d[4]*c3[i, k] + d[5]*c4[i, k]

B[i,k] <- d[6]*c5[i, k] + d[7]*c6[i, k] + d[8]*c7[i, k] + d[9]*c8[i, k]

C[i,k] <- d[10]*c9[i, k] + d[11]*c10[i, k] + d[12]*c11[i, k] + d[13]*c12[i, k]

D[i,k] <- d[14]*c13[i, k] + d[15]*c14[i, k]+ d[16]*c15[i, k]+ d[17]*c16[i, k]

E[i,k] <- d[18]*c17[i, k] + d[19]*c18[i, k]+ d[20]*c19[i, k]+ d[21]*c20[i, k]

F[i,k] <- d[22]*c21[i, k] + d[23]*c22[i, k]+ d[24]*c23[i, k]+ d[25]*c24[i, k]

G[i,k] <- d[26]*c25[i, k] + d[27]*c26[i, k]+ d[28]*c27[i, k]+ d[29]*c28[i, k]

H[i,k] <- d[30]*c29[i, k] + d[31]*c30[i, k]+ d[32]*c31[i, k]+ d[33]*c32[i, k]

I[i,k] <- d[34]*c33[i, k] + d[35]*c34[i, k]+ d[36]*c35[i, k] + d[37]*c36[i, k]

mdA[i,k] <- A[i,k] + B[i,k] + C[i,k] + D[i,k]+ E[i,k] + F[i,k] + G[i,k] + H[i,k] + I[i,k]

Aa[i,k] <- d[2]*c1[i, 1] + d[3]*c2[i, 1] + d[4]*c3[i, 1] + d[5]*c4[i, 1]

Bb[i,k] <- d[6]*c5[i, 1] + d[7]*c6[i, 1] + d[8]*c7[i, 1] + d[9]*c8[i, 1]

Cc[i,k] <- d[10]*c9[i, 1] + d[11]*c10[i, 1] + d[12]*c11[i, 1] + d[13]*c12[i, 1]

Dd[i,k] <- d[14]*c13[i, 1] + d[15]*c14[i, 1]+ d[16]*c15[i, 1]+ d[17]*c16[i, 1]

Ee[i,k] <- d[18]*c17[i, 1] + d[19]*c18[i, 1]+ d[20]*c19[i, 1]+ d[21]*c20[i, 1]

Ff[i,k] <- d[22]*c21[i, 1] + d[23]*c22[i, 1]+ d[24]*c23[i, 1]+ d[25]*c24[i, 1]

Gg[i,k] <- d[26]*c25[i, 1] + d[27]*c26[i, 1]+ d[28]*c27[i, 1]+ d[29]*c28[i, 1]

Hh[i,k] <- d[30]*c29[i, 1] + d[31]*c30[i, 1]+ d[32]*c31[i, 1]+ d[33]*c32[i, 1]

Ii[i,k] <- d[34]*c33[i, 1] + d[35]*c34[i, 1]+ d[36]*c35[i, 1] + d[37]*c36[i, 1]

mdB[i,k] <- Aa[i,k] + Bb[i,k] + Cc[i,k] +Dd[i,k] + Ee[i,k] + Ff[i,k] +Gg[i,k] +Hh[i,k] +Ii[i,k]

md[i,k] <- mdA[i,k] - mdB[i,k] + sw[i,k]

# precision of RE distributions (with multi-arm trial correction)

taud[i,k] <- tau *2*(k-1)/k

#adjustment, multi-arm RCTs

w[i,k] <- delta[i,k] - mdA[i, k] + mdB[i,k]

# cumulative adjustment for multi-arm trials

sw[i,k] <-sum(w[i,1:k-1])/(k-1)

}

}

totresdev <- sum(resdev[]) #Total Residual Deviance

d[1]<-0 # treatment effect is zero for control arm

# vague priors for component effects

for (k in 2:nt){

d[k] ~ dnorm(0,.0001)

}

sdbt ~ dunif(0,10) # vague prior for for between-trial SD

tau <- pow(sdbt,-2) # between-trial precision = (1/between-trial variance)

}

### Interaction model

# Within groups standard deviation

# s_within = ( ( ((n1-1)*sd1^2) + ((n2-1)*sd2^2) ) / (n1+n2-2) )^0.5

model{

for(i in 1:Ntrials){ # LOOP THROUGH STUDIES

w[i,1] <- 0 # adjustment for multi-arm trials is zero for control arm

delta[i,1] <- 0 # treatment effect is zero for control arm

mu[i] ~ dnorm(0,.0001) # vague priors for all trial baselines

for (k in 1:na[i]){

prec[i,k] <- n[i,k]/pow(sd[i,k],2) # set precisions

var[i,k] <- 1/prec[i,k] # set variances

y[i,k] ~ dnorm(phi[i,k], prec[i,k]) # normal likelihood

phi[i,k] <- theta[i,k]*s_within[i] # theta is standardised mean

theta[i,k] <- mu[i] + delta[i,k] # model for linear predictor, delta is SMD

# deviance contribution

dev[i,k] <- (y[i,k]-phi[i,k])*(y[i,k]-phi[i,k])/var[i,k]

}

# summed residual deviance contribution for this trial

resdev[i] <- sum(dev[i,1:na[i]])

for (k in 2:na[i]){ # LOOP THROUGH ARMS

# trial-specific RE distributions

delta[i,k] ~ dnorm(md[i,k], taud[i,k])

# mean of treatment effect distributions, with multi-arm trial correction

A[i,k] <- d[2]*c1[i, k] + d[3]*c2[i, k] + d[4]*c3[i, k] + d[5]*c4[i, k]

B[i,k] <- d[6]*c5[i, k] + d[7]*c6[i, k] + d[8]*c7[i, k] + d[9]*c8[i, k]

C[i,k] <- d[10]*c9[i, k] + d[11]*c10[i, k] + d[12]*c11[i, k] + d[13]*c12[i, k]

D[i,k] <- d[14]*c13[i, k] + d[15]*c14[i, k]+ d[16]*c15[i, k]+ d[17]*c16[i, k]

E[i,k] <- d[18]*c17[i, k] + d[19]*c18[i, k]+ d[20]*c19[i, k]+ d[21]*c20[i, k]

F[i,k] <- d[22]*c21[i, k] + d[23]*c22[i, k]+ d[24]*c23[i, k]+ d[25]*c24[i, k]

G[i,k] <- d[26]*c25[i, k] + d[27]*c26[i, k]+ d[28]*c27[i, k]+ d[29]*c28[i, k]

H[i,k] <- d[30]*c29[i, k] + d[31]*c30[i, k]+ d[32]*c31[i, k]+ d[33]*c32[i, k]

I[i,k] <- d[34]*c33[i, k] + d[35]*c34[i, k]+ d[36]*c35[i, k] + d[37]*c36[i, k] + d[38]*c1[i, k]*c3[i, k] + d[39]*c1[i, k]*c31[i, k]

mdA[i,k] <- A[i,k] + B[i,k] + C[i,k] + D[i,k]+ E[i,k] + F[i,k] + G[i,k] + H[i,k] + I[i,k]

Aa[i,k] <- d[2]*c1[i, 1] + d[3]*c2[i, 1] + d[4]*c3[i, 1] + d[5]*c4[i, 1]

Bb[i,k] <- d[6]*c5[i, 1] + d[7]*c6[i, 1] + d[8]*c7[i, 1] + d[9]*c8[i, 1]

Cc[i,k] <- d[10]*c9[i, 1] + d[11]*c10[i, 1] + d[12]*c11[i, 1] + d[13]*c12[i, 1]

Dd[i,k] <- d[14]*c13[i, 1] + d[15]*c14[i, 1]+ d[16]*c15[i, 1]+ d[17]*c16[i, 1]

Ee[i,k] <- d[18]*c17[i, 1] + d[19]*c18[i, 1]+ d[20]*c19[i, 1]+ d[21]*c20[i, 1]

Ff[i,k] <- d[22]*c21[i, 1] + d[23]*c22[i, 1]+ d[24]*c23[i, 1]+ d[25]*c24[i, 1]

Gg[i,k] <- d[26]*c25[i, 1] + d[27]*c26[i, 1]+ d[28]*c27[i, 1]+ d[29]*c28[i, 1]

Hh[i,k] <- d[30]*c29[i, 1] + d[31]*c30[i, 1]+ d[32]*c31[i, 1]+ d[33]*c32[i, 1]

Ii[i,k] <- d[34]*c33[i, 1] + d[35]*c34[i, 1]+ d[36]*c35[i, 1] + d[37]*c36[i, 1] + d[38]*c1[i, 1]*c3[i, 1] + d[39]*c1[i, 1]*c31[i, 1]

mdB[i,k] <- Aa[i,k] + Bb[i,k] + Cc[i,k] +Dd[i,k] + Ee[i,k] + Ff[i,k] +Gg[i,k] +Hh[i,k] +Ii[i,k]

md[i,k] <- mdA[i,k] - mdB[i,k] + sw[i,k]

# precision of RE distributions (with multi-arm trial correction)

taud[i,k] <- tau *2*(k-1)/k

#adjustment, multi-arm RCTs

w[i,k] <- delta[i,k] - mdA[i, k] + mdB[i,k]

# cumulative adjustment for multi-arm trials

sw[i,k] <-sum(w[i,1:k-1])/(k-1)

}

}

totresdev <- sum(resdev[]) #Total Residual Deviance

d[1]<-0 # treatment effect is zero for control arm

# vague priors for component effects

for (k in 2:nt){

d[k] ~ dnorm(0,.0001)

}

sdbt ~ dunif(0,10) # vague prior for for between-trial SD

tau <- pow(sdbt,-2) # between-trial precision = (1/between-trial variance)

}

### Example covariate model – outcome at baseline

# Within groups standard deviation

# s_within = ( ( ((n1-1)*sd1^2) + ((n2-1)*sd2^2) ) / (n1+n2-2) )^0.5

model{

for(i in 1:Ntrials){ # LOOP THROUGH STUDIES

w[i,1] <- 0 # adjustment for multi-arm trials is zero for control arm

delta[i,1] <- 0 # treatment effect is zero for control arm

mu[i] ~ dnorm(0,.0001) # vague priors for all trial baselines

Base[i] ~ dnorm(zeta, x.phi)

for (k in 1:na[i]){

prec[i,k] <- n[i,k]/pow(sd[i,k],2) # set precisions

var[i,k] <- 1/prec[i,k] # set variances

y[i,k] ~ dnorm(phi[i,k], prec[i,k]) # normal likelihood

phi[i,k] <- theta[i,k]*s_within[i] # theta is standardised mean

theta[i,k] <- mu[i] + delta[i,k] # model for linear predictor, delta is SMD

# deviance contribution

dev[i,k] <- (y[i,k]-phi[i,k])*(y[i,k]-phi[i,k])/var[i,k]

}

# summed residual deviance contribution for this trial

resdev[i] <- sum(dev[i,1:na[i]])

for (k in 2:na[i]){ # LOOP THROUGH ARMS

# trial-specific RE distributions

delta[i,k] ~ dnorm(md[i,k], taud[i,k])

# mean of treatment effect distributions, with multi-arm trial correction

A[i,k] <- d[2]*c1[i, k] + d[3]*c2[i, k] + d[4]*c3[i, k] + d[5]*c4[i, k]

B[i,k] <- d[6]*c5[i, k] + d[7]*c6[i, k] + d[8]*c7[i, k] + d[9]*c8[i, k]

C[i,k] <- d[10]*c9[i, k] + d[11]*c10[i, k] + d[12]*c11[i, k] + d[13]*c12[i, k]

D[i,k] <- d[14]*c13[i, k] + d[15]*c14[i, k]+ d[16]*c15[i, k]+ d[17]*c16[i, k]

E[i,k] <- d[18]*c17[i, k] + d[19]*c18[i, k]+ d[20]*c19[i, k]+ d[21]*c20[i, k]

F[i,k] <- d[22]*c21[i, k] + d[23]*c22[i, k]+ d[24]*c23[i, k]+ d[25]*c24[i, k]

G[i,k] <- d[26]*c25[i, k] + d[27]*c26[i, k]+ d[28]*c27[i, k]+ d[29]*c28[i, k]

H[i,k] <- d[30]*c29[i, k] + d[31]*c30[i, k]+ d[32]*c31[i, k]+ d[33]*c32[i, k]

I[i,k] <- d[34]*c33[i, k] + d[35]*c34[i, k]+ d[36]*c35[i, k] + d[37]*c36[i, k]

mdA[i,k] <- A[i,k] + B[i,k] + C[i,k] + D[i,k]+ E[i,k] + F[i,k] + G[i,k] + H[i,k] + I[i,k]

Aa[i,k] <- d[2]*c1[i, 1] + d[3]*c2[i, 1] + d[4]*c3[i, 1] + d[5]*c4[i, 1]

Bb[i,k] <- d[6]*c5[i, 1] + d[7]*c6[i, 1] + d[8]*c7[i, 1] + d[9]*c8[i, 1]

Cc[i,k] <- d[10]*c9[i, 1] + d[11]*c10[i, 1] + d[12]*c11[i, 1] + d[13]*c12[i, 1]

Dd[i,k] <- d[14]*c13[i, 1] + d[15]*c14[i, 1]+ d[16]*c15[i, 1]+ d[17]*c16[i, 1]

Ee[i,k] <- d[18]*c17[i, 1] + d[19]*c18[i, 1]+ d[20]*c19[i, 1]+ d[21]*c20[i, 1]

Ff[i,k] <- d[22]*c21[i, 1] + d[23]*c22[i, 1]+ d[24]*c23[i, 1]+ d[25]*c24[i, 1]

Gg[i,k] <- d[26]*c25[i, 1] + d[27]*c26[i, 1]+ d[28]*c27[i, 1]+ d[29]*c28[i, 1]

Hh[i,k] <- d[30]*c29[i, 1] + d[31]*c30[i, 1]+ d[32]*c31[i, 1]+ d[33]*c32[i, 1]

Ii[i,k] <- d[34]*c33[i, 1] + d[35]*c34[i, 1]+ d[36]*c35[i, 1] + d[37]*c36[i, 1]

mdB[i,k] <- Aa[i,k] + Bb[i,k] + Cc[i,k] +Dd[i,k] + Ee[i,k] + Ff[i,k] +Gg[i,k] +Hh[i,k] +Ii[i,k]

md[i,k] <- mdA[i,k] - mdB[i,k] + sw[i,k] + covar[i,k]

# precision of RE distributions (with multi-arm trial correction)

taud[i,k] <- tau *2*(k-1)/k

#adjustment, multi-arm RCTs

w[i,k] <- delta[i,k] - mdA[i, k] + mdB[i,k] + covar[i,k]

# cumulative adjustment for multi-arm trials

sw[i,k] <-sum(w[i,1:k-1])/(k-1)

# Covariate adjustment

covar1[i,k] <- beta1[2]*c1[i, k] + beta1[3]*c2[i, k] + beta1[4]*c3[i, k] + beta1[5]*c4[i, k] + beta1[6]*c5[i, k]

+ beta1[7]*c6[i, k]+ beta1[8]*c7[i, k]+ beta1[9]*c8[i, k] + beta1[10]*c9[i, k]+ beta1[11]*c10[i, k]+ beta1[12]*c11[i, k]

+ beta1[15]*c14[i, k]+ beta1[19]*c18[i, k]+ beta1[21]*c20[i, k]+ beta1[23]*c22[i, k]+ beta1[24]*c23[i, k]+ beta1[25]*c24[i, k]

+ beta1[31]*c30[i, k]+ beta1[32]*c31[i, k]

covar[i,k] <- (covar1[i,k] -(beta1[2]*c1[i, 1] + beta1[3]*c2[i, 1] + beta1[4]*c3[i, 1] +

beta1[5]*c4[i, 1] + beta1[6]*c5[i, 1] + beta1[7]*c6[i, 1]+ beta1[8]*c7[i, 1]+ beta1[9]*c8[i, 1]+ beta1[10]*c9[i, 1]

+ beta1[11]*c10[i, 1]+ beta1[12]*c11[i, 1]+ beta1[15]*c14[i, 1]+ beta1[19]*c18[i, 1]+ beta1[21]*c20[i, 1]+ beta1[23]*c22[i, 1]

+ beta1[24]*c23[i, 1]+ beta1[25]*c24[i, 1]+ beta1[31]*c30[i, 1]+ beta1[32]*c31[i, 1]))*Base[i]

}

}

totresdev <- sum(resdev[]) #Total Residual Deviance

d[1]<-0 # treatment effect is zero for control arm

beta1[1] <- 0 # covariate effect is zero for reference treatment

# vague priors for component effects

for (k in 2:nt){

d[k] ~ dnorm(0,.0001)

beta1[k] ~ dnorm(0, 0.0001) # independent covariate effect

}

sdbt ~ dunif(0,10) # vague prior for for between-trial SD

tau <- pow(sdbt,-2) # between-trial precision = (1/between-trial variance)

zeta ~ dnorm(0, 0.001)

x.phi ~ dunif(0, 10)

temp ~ dnorm(zeta, x.phi)

}

# References

1 Sterne JAC, Sutton AJ, Ioannidis JPA, *et al.* Recommendations for examining and interpreting funnel plot asymmetry in meta-analyses of randomised controlled trials. *BMJ* 2011;**343**. doi:10.1136/BMJ.D4002

2 Abdelbasset WK, Alrawaili SM, Moawd SA, *et al.* Effect of 12-week endurance exercise on obese elderly patients with COPD: a randomized trial. *J Adv Pharm Educ Res* 2020;**10**:100‐106.www.japer.in (accessed 1 May 2024).

3 Abedi Yekta AH, Poursaeid Esfahani M, Salehi S, *et al.* Assessment of the Effects of Inspiratory Muscle Training (IMT) and Aerobic Training on the Quality of Life of Patients with Chronic Obstructive Pulmonary Disease. *Tanaffos* 2019;**18**:223–9.http://ovidsp.ovid.com/ovidweb.cgi?T=JS&PAGE=reference&D=pmnm4&NEWS=N&AN=32411262

4 Acheche A, Mekki M, Paillard T, *et al.* The Effect of Adding Neuromuscular Electrical Stimulation with Endurance and Resistance Training on Exercise Capacity and Balance in Patients with Chronic Obstructive Pulmonary Disease: A Randomized Controlled Trial. *Can Respir J* 2020;**2020**:9826084. doi:10.1155/2020/9826084

5 Ahnfeldt-Mollerup P, Hey H, Johansen C, *et al.* The effect of protein supplementation on quality of life, physical function, and muscle strength in patients with chronic obstructive pulmonary disease. *Eur J Phys Rehabil Med* 2015;**51**:447–56. doi:R33Y9999N00A140381 [pii]

6 Alcazar J, Losa-Reyna J, Rodriguez-Lopez C, *et al.* Effects of concurrent exercise training on muscle dysfunction and systemic oxidative stress in older people with COPD. *Scand J Med Sci Sports* 2019;**29**:1591–603.http://ovidsp.ovid.com/ovidweb.cgi?T=JS&PAGE=reference&D=med16&NEWS=N&AN=31169924 %25[20190624//

7 Aldhahir AM, Aldabayan YS, Alqahtani JS, *et al.* A double-blind randomised controlled trial of protein supplementation to enhance exercise capacity in COPD during pulmonary rehabilitation: a pilot study. *ERJ open Res* 2021;**7**. doi:10.1183/23120541.00077-2021

8 Alexander JL, Wagner CL, Alex, *et al.* Is harmonica playing an effective adjunct therapy to pulmonary rehabilitation? *Rehabil Nurs* 2012;**37**:207–12. doi:10.1002/rnj.33

9 Alsharaway LA, Elyazed TIA, Helmy NA. Effect of new reflexology maneuver on lung function and functional ability of chronic obstructive pulmonary disease patients in pulmonary rehabilitation program: A randomized control trial. *Fizjoterapia Pol* 2021;**21**:160–5.http://www.fizjoterapiapolska.pl/ http://ovidsp.ovid.com/ovidweb.cgi?T=JS&PAGE=reference&D=emca&NEWS=N&AN=2016767121

10 Altenburg WA, Ten Hacken NHT, Bossenbroek L, *et al.* Short- and long-term effects of a physical activity counselling programme in COPD: A randomized controlled trial. *Respir Med* 2015;**109**:112–21. doi:10.1016/j.rmed.2014.10.020

11 Ambrosino N, Foglio K, Balzano G, *et al.* Tiotropium and exercise training in COPD patients: effects on dyspnea and exercise tolerance. *Int J Chron Obstruct Pulmon Dis* 2008;**3**:771–80.

12 Aquino G, Iuliano E, di Cagno A, *et al.* Effects of combined training vs aerobic training on cognitive functions in COPD: a randomized controlled trial. *Int J Chron Obstruct Pulmon Dis* 2016;**11**:711–8.http://ovidsp.ovid.com/ovidweb.cgi?T=JS&PAGE=reference&D=med13&NEWS=N&AN=27110107 %25[20160404//

13 Arbillaga-Etxarri A, Gimeno-Santos E, Barberan-Garcia A, *et al.* Long-term efficacy and effectiveness of a behavioural and community-based exercise intervention (Urban Training) to increase physical activity in patients with COPD: a randomised controlled trial. *Eur Respir J* 2018;**52**.http://ovidsp.ovid.com/ovidweb.cgi?T=JS&PAGE=reference&D=med15&NEWS=N&AN=30166322 %25[20181018//

14 Armstrong M, Hume E, McNeillie L, *et al.* Behavioural modification interventions alongside pulmonary rehabilitation improve COPD patients’ experiences of physical activity. *Respir Med* 2021;**180**:106353. doi:10.1016/J.RMED.2021.106353

15 Arnardottir RH, Sorensen S, Ringqvist I, *et al.* Two different training programmes for patients with COPD: A randomised study with 1-year follow-up. *Respir Med* 2006;**100**:130–9. doi:10.1016/j.rmed.2005.03.043

16 Arnardottir RH, Boman G, Larsson K, *et al.* Interval training compared with continuous training in patients with COPD. *Respir Med* 2007;**101**:1196–204. doi:10.1016/j.rmed.2006.11.004

17 Arnedillo A, Gonzalez-Montesinos JL, Fern, *et al.* Effects of a Rehabilitation Programme with a Nasal Inspiratory Restriction Device on Exercise Capacity and Quality of Life in COPD. *Int J Environ Res Public Health* 2020;**17**.http://ovidsp.ovid.com/ovidweb.cgi?T=JS&PAGE=reference&D=med17&NEWS=N&AN=32456097 %25[20200522//

18 Gonzalez-Montesinos JL, Fern, ez-Santos JR, *et al.* Effects of a Rehabilitation Programme Using a Nasal Inspiratory Restriction Device in COPD. *Int J Environ Res Public Health* 2021;**18**.http://ovidsp.ovid.com/ovidweb.cgi?T=JS&PAGE=reference&D=med19&NEWS=N&AN=33921105 %25[20210415//

19 Arslan S, Oztunc G. The Effects of a Walking Exercise Program on Fatigue in the Person with COPD. *Rehabil Nurs* 2016;**41**:303–12.http://ovidsp.ovid.com/ovidweb.cgi?T=JS&PAGE=reference&D=med13&NEWS=N&AN=25772587 %25[20150314//

20 Barakat S, Michele G, George P, *et al.* Outpatient pulmonary rehabilitation in patients with chronic obstructive pulmonary disease. *Int J Chron Obstruct Pulmon Dis* 2008;**3**:155–62.

21 Beauchamp MK, Janaudis-Ferreira T, Parreira V, *et al.* A randomized controlled trial of balance training during pulmonary rehabilitation for individuals with COPD. *Chest* 2013;**144**:1803–10. doi:10.1378/chest.13-1093

22 Beaumont M, Mialon P, Le Ber C, *et al.* Effects of inspiratory muscle training on dyspnoea in severe COPD patients during pulmonary rehabilitation: controlled randomised trial. *Eur Respir J* 2018;**51**.http://ovidsp.ovid.com/ovidweb.cgi?T=JS&PAGE=reference&D=med15&NEWS=N&AN=29371379 %25[20180125//

23 Bendstrup KE, Ingemann Jensen J, Holm S, *et al.* Out-patient rehabilitation improves activities of daily living, quality of life and exercise tolerance in chronic obstructive pulmonary disease. *Eur Respir J* 1997;**10**:2801–6.

24 Benzo RP, Ridgeway J, Hoult JP, *et al.* Feasibility of a Health Coaching and Home-Based Rehabilitation Intervention With Remote Monitoring for COPD. *Respir Care* 2021;**66**:960–71. doi:https://dx.doi.org/10.4187/respcare.08580

25 Benzo R, Hoult J, McEvoy C, *et al.* Promoting Chronic Obstructive Pulmonary Disease Wellness through Remote Monitoring and Health Coaching: A Clinical Trial. *Ann Am Thorac Soc* 2022;**19**:1808–17. doi:https://dx.doi.org/10.1513/AnnalsATS.202203-214OC

26 Bernard S, Whittom F, Leblanc P, *et al.* Aerobic and strength training in patients with chronic obstructive pulmonary disease. *Am J Respir Crit Care Med* 1999;**159**:896–901. doi:10.1164/ajrccm.159.3.9807034

27 Berry MJ, Rejeski WJ, Miller ME, *et al.* A lifestyle activity intervention in patients with chronic obstructive pulmonary disease. *Respir Med* 2010;**104**:829–39. doi:10.1016/j.rmed.2010.02.015

28 Bhasin P, An, a Subramanian S. Effects of Short Term Pulmonary Rehabilitation with or without Respiratory Muscle Stretch Gymnastics on Quality of Life in Patients with Chronic Obstructive Pulmonary Disease. *Indian J Physiother Occup Ther* 2012;**6**:187–90.https://search.ebscohost.com/login.aspx?direct=true&AuthType=sso&db=cin20&AN=82959667&site=ehost-live&custid=ns124898

29 Bianchi L, Foglio K, Porta R, *et al.* Lack of additional effect of adjunct of assisted ventilation to pulmonary rehabilitation in mild COPD patients. *Respir Med* 2002;**96**:359–67. doi:10.1053/rmed.2001.1287

30 Bianco JP, Sciriha A, Lungaro-Mifsud S, *et al.* Pulmonary rehabilitation for chronic obstructive pulmonary disease: Effects of a high versus low intensity programme. *Int J Ther Rehabil* 2019;**26**:ijtr20180047.http://www.ijtr.co.uk/ http://ovidsp.ovid.com/ovidweb.cgi?T=JS&PAGE=reference&D=emca3&NEWS=N&AN=630503913

31 Bjørgen S, Hoff J, Husby VS, *et al.* Aerobic high intensity one and two legs interval cycling in chronic obstructive pulmonary disease: the sum of the parts is greater than the whole. *Eur J Appl Physiol* 2009;**106**:501–7. doi:10.1007/S00421-009-1038-1

32 Bjorgen S, Helgerud J, Husby V, *et al.* Aerobic high intensity one-legged interval cycling improves peak oxygen uptake in chronic obstructive pulmonary disease patients; no additional effect from hyperoxia. *Int J Sports Med* 2009;**30**:872–8. doi:10.1055/s-0029-1238292

33 Blackstock FC, Webster KE, McDonald CF, *et al.* Comparable improvements achieved in chronic obstructive pulmonary disease through pulmonary rehabilitation with and without a structured educational intervention: a randomized controlled trial. *Respirology* 2014;**19**:193–202. doi:10.1111/resp.12203

34 Bonnevie T, Gravier FE, Debeaumont D, *et al.* Home-based Neuromuscular Electrical Stimulation as an Add-on to Pulmonary Rehabilitation Does Not Provide Further Benefits in Patients With Chronic Obstructive Pulmonary Disease: A Multicenter Randomized Trial. *Arch Phys Med Rehabil* 2018;**99**:1462–70. doi:10.1016/j.apmr.2018.01.024

35 Borghi-Silva A, Baldissera V, Sampaio LMMM, *et al.* L-carnitine as an ergogenic aid for patients with chronic obstructive pulmonary disease submitted to whole-body and respiratory muscle training programs. *Brazilian J Med Biol Res* 2006;**39**:465–74. doi:10.1590/S0100-879X2006000400006

36 Borghi-Silva A, Arena R, Castello V, *et al.* Aerobic exercise training improves autonomic nervous control in patients with COPD. *Respir Med* 2009;**103**:1503–10. doi:10.1016/j.rmed.2009.04.015

37 Borghi-Silva A, Mendes RGG, Toledo AC, *et al.* Adjuncts to physical training of patients with severe COPD: oxygen or noninvasive ventilation? *Respir Care* 2010;**55**:885–94. doi:10.1016/S0002-8703(03)00499-X

38 Bourbeau J, De Sousa Sena R, Taivassalo T, *et al.* Eccentric versus conventional cycle training to improve muscle strength in advanced COPD: A randomized clinical trial. *Respir Physiol Neurobiol* 2020;**276**:103414. doi:10.1016/j.resp.2020.103414

39 Bourjeily-Habr G, Rochester CL, Palermo F, *et al.* Randomised controlled trial of transcutaneous electrical muscle stimulation of the lower extremities in patients with chronic obstructive pulmonary disease. *Thorax* 2002;**57**:1045–9.

40 Bourne S, Devos R, North M, *et al.* Online versus face-to-face pulmonary rehabilitation for patients with chronic obstructive pulmonary disease: randomised controlled trial. *BMJ Open* 2017;**7**:e014580. doi:10.1136/bmjopen-2016-014580

41 Bourne C, Houchen-Wolloff L, Patel P, *et al.* Self-management programme of activity coping and education-SPACE for COPD(C)-in primary care: a pragmatic randomised trial. *BMJ open Respir Res* 2022;**9**. doi:https://dx.doi.org/10.1136/bmjresp-2022-001443

42 Boxall A-M, Barclay L, Sayers A, *et al.* Managing chronic obstructive pulmonary disease in the community. A randomized controlled trial of home-based pulmonary rehabilitation for elderly housebound patients. *J Cardiopulm Rehabil* 2005;**25**:378–85.

43 Breyer M-K, Breyer-Kohansal R, Funk G-C, *et al.* Nordic walking improves daily physical activities in COPD: a randomised controlled trial. *Respir Res* 2010;**11**:112. doi:10.1186/1465-9921-11-112

44 Broekhuizen R, Wouters EFM, Creutzberg EC, *et al.* Polyunsaturated fatty acids improve exercise capacity in chronic obstructive pulmonary disease. *Thorax* 2005;**60**:376–82. doi:10.1136/thx.2004.030858

45 Brønstad E, Tjonna AE, Rognmo Ø, *et al.* Aerobic exercise training improves right- and left ventricular systolic function in patients with COPD. *COPD J Chronic Obstr Pulm Dis* 2013;**10**:300–6. doi:10.3109/15412555.2012.745843

46 Burtin C, Langer D, Van Remoortel H, *et al.* Physical activity counselling during pulmonary rehabilitation in patients with COPD: A randomised controlled trial. *PLoS One* 2015;**10**:e0144989. doi:10.1371/journal.pone.0144989

47 Busch AJ, McClements JD. Effects of a supervised home exercise program on patients with severe chronic obstructive pulmonary disease. *Phys Ther* 1988;**68**:469–74.

48 Bustamante V, Lopez de Santa Maria E, Gorostiza A, *et al.* Muscle training with repetitive magnetic stimulation of the quadriceps in severe COPD patients. *Respir Med* 2010;**104**:237–45. doi:10.1016/j.rmed.2009.10.001

49 Cambach W, Chadwick-Straver R V, Wagenaar RC, *et al.* The effects of a community-based pulmonary rehabilitation programme on exercise tolerance and quality of life: a randomized controlled trial. *Eur Respir J* 1997;**10**:104–13.

50 Cameron-Tucker HL, Wood-Baker R, Joseph L, *et al.* A randomized controlled trial of telephone-mentoring with home-based walking preceding rehabilitation in COPD. *Int J Chron Obstruct Pulmon Dis* 2016;**11**:1991–2000.http://ovidsp.ovid.com/ovidweb.cgi?T=JS&PAGE=reference&D=med13&NEWS=N&AN=27601892 %25[20160825//

51 Camillo CA, Osadnik CR, Burtin C, *et al.* Effects of downhill walking in pulmonary rehabilitation for patients with COPD: a randomised controlled trial. *Eur Respir J* 2020;**56**.http://ovidsp.ovid.com/ovidweb.cgi?T=JS&PAGE=reference&D=med17&NEWS=N&AN=32444407 %25[20200917//

52 Carrieri-Kohlman V, Gormley JM, Douglas MK, *et al.* Exercise training decreases dyspnea and the distress and anxiety associated with it. Monitoring alone may be as effective as coaching. *Chest* 1996;**110**:1526–35.http://ovidsp.ovid.com/ovidweb.cgi?T=JS&PAGE=reference&D=med4&NEWS=N&AN=8989072

53 Casaburi R, Patessio A, Ioli F, *et al.* Reductions in Exercise Lactic Acidosis and Ventilation as a Result of Exercise Training in Patients with Obstructive Lung Disease. *Am Rev Respir Dis* 1991;**143**:9–18. doi:10.1164/ajrccm/143.1.9

54 Casaburi R, Kukafka D, Cooper CB, *et al.* Improvement in exercise tolerance with the combination of tiotropium and pulmonary rehabilitation in patients with COPD. *Chest* 2005;**127**:809–17. doi:10.1378/chest.127.3.809

55 Casey D, Murphy K, Devane D, *et al.* The effectiveness of a structured education pulmonary rehabilitation programme for improving the health status of people with moderate and severe chronic obstructive pulmonary disease in primary care: the PRINCE cluster randomised trial. *Thorax* 2013;**68**:922–8. doi:10.1136/thoraxjnl-2012-203103

56 Chan AWK, Lee A, Suen LKP, *et al.* Effectiveness of a Tai chi Qigong program in promoting health-related quality of life and perceived social support in chronic obstructive pulmonary disease clients. *Qual Life Res* 2010;**19**:653–64. doi:10.1007/s11136-010-9632-6

57 Chaplin E, Hewitt S, Apps L, *et al.* Interactive web-based pulmonary rehabilitation programme: a randomised controlled feasibility trial. *BMJ Open* 2017;**7**:e013682. doi:10.1136/BMJOPEN-2016-013682

58 Charususin N, Sricharoenchai T, Pongpanit K, *et al.* Beneficial Effect of Water-Based Exercise Training on Exercise Capacity in COPD Patients-a Pilot Study. *Front Rehabil Sci* 2021;**2**:728973. doi:https://dx.doi.org/10.3389/fresc.2021.728973

59 Chen W, Lai X, Xie S. Comparison of effects of endurance training and strength training on exercise endurance and quality of life of patients with chronic obstructive pulmonary disease. *Chinese J Rehabil Med* 2015;**30**:152–7.http://www.oriprobe.com/journals/zgkfyxzz.html http://ovidsp.ovid.com/ovidweb.cgi?T=JS&PAGE=reference&D=emca3&NEWS=N&AN=603847926

60 Chen Q, Shi MY, Zhang W, *et al.* Effect of breathing and Daoyin exercises on the quality of life in patients with chronic obstructive pulmonary disease. *J Acupunct Tuina Sci* 2015;**13**:353–60.http://www.springer.com/west/home?SGWID=4-102-70-173667206-0&changeHeader=true http://ovidsp.ovid.com/ovidweb.cgi?T=JS&PAGE=reference&D=emca3&NEWS=N&AN=607149778

61 Chen Y, Niu M, Zhang X, *et al.* Effects of home-based lower limb resistance training on muscle strength and functional status in stable Chronic obstructive pulmonary disease patients. *J Clin Nurs* 2018;**27**:e1022–37.http://ovidsp.ovid.com/ovidweb.cgi?T=JS&PAGE=reference&D=med15&NEWS=N&AN=29076609 %25[20180206//

62 Clark CJ, Cochrane L, Mackay E. Low intensity peripheral muscle conditioning improves exercise tolerance and breathlessness in COPD. *Eur Respir J* 1996;**9**:2590–6. doi:10.1183/09031936.96.09122590

63 Clark CJ, Cochrane LM, Mackay E, *et al.* Skeletal muscle strength and endurance in patients with mild COPD and the effects of weight training. *Eur Respir J* 2000;**15**:92–7.http://ovidsp.ovid.com/ovidweb.cgi?T=JS&PAGE=reference&D=med4&NEWS=N&AN=10678627

64 Collins EG, Langbein WE, Fehr L, *et al.* Can ventilation-feedback training augment exercise tolerance in patients with chronic obstructive pulmonary disease? *Am J Respir Crit Care Med* 2008;**177**:844–52. doi:10.1164/rccm.200703-477OC

65 Collins EG, Jelinek C, O’Connell S, *et al.* Contrasting breathing retraining and helium-oxygen during pulmonary rehabilitation in COPD: a randomized clinical trial. *Respir Med* 2014;**108**:297–306. doi:10.1016/j.rmed.2013.10.023

66 Collins EG, Jelinek C, O’Connell S, *et al.* The Effect of Breathing Retraining Using Metronome-Based Acoustic Feedback on Exercise Endurance in COPD: A Randomized Trial. *Lung* 2019;**197**:181–8.http://ovidsp.ovid.com/ovidweb.cgi?T=JS&PAGE=reference&D=med16&NEWS=N&AN=30739217 %25[20190209//

67 Constantin D, Menon MK, Houchen-Wolloff L, *et al.* Skeletal muscle molecular responses to resistance training and dietary supplementation in COPD. *Thorax* 2013;**68**:625–33. doi:10.1136/thoraxjnl-2012-202764

68 Coppoolse R, Schols AM, Baarends EM, *et al.* Interval versus continuous training in patients with severe COPD: a randomized clinical trial. *Eur Respir J* 1999;**14**:258–63.

69 Costi S, Crisafulli E, Antoni FD, *et al.* Effects of unsupported upper extremity exercise training in patients with COPD: a randomized clinical trial. *Chest* 2009;**136**:387–95. doi:10.1378/chest.09-0165

70 Covey MK, McAuley E, Kapella MC, *et al.* Upper-body resistance training and self-efficacy enhancement in COPD. *J Pulm Respir Med* 2012;:1.https://www.cochranelibrary.com/central/doi/10.1002/central/CN-01616817/full

71 Covey MK, Collins EG, Reynertson SI, *et al.* Resistance training as a preconditioning strategy for enhancing aerobic exercise training outcomes in COPD. *Respir Med* 2014;**108**:1141–52. doi:10.1016/j.rmed.2014.06.001

72 Cox NS, McDonald CF, Mahal A, *et al.* Telerehabilitation for chronic respiratory disease: a randomised controlled equivalence trial. *Thorax* 2022;**77**:643–51. doi:https://dx.doi.org/10.1136/thoraxjnl-2021-216934

73 Creutzberg EC, Wouters EFM, Mostert R, *et al.* A role for anabolic steroids in the rehabilitation of patients with COPD? A double-blind, placebo-controlled, randomized trial. *Chest* 2003;**124**:1733–42.http://www.ncbi.nlm.nih.gov/pubmed/14605042 (accessed 4 Jan 2017).

74 Cruz J, Brooks D, Marques A. Walk2Bactive: A randomised controlled trial of a physical activity-focused behavioural intervention beyond pulmonary rehabilitation in chronic obstructive pulmonary disease. *Chron Respir Dis* 2016;**13**:57–66. doi:10.1177/1479972315619574

75 Curtis KJ, Meyrick VM, Mehta B, *et al.* Angiotensin-Converting Enzyme Inhibition as an Adjunct to Pulmonary Rehabilitation in Chronic Obstructive Pulmonary Disease. *Am J Respir Crit Care Med* 2016;**194**:1349–57. doi:10.1164/RCCM.201601-0094OC

76 de Bisschop C, Caron F, Ingrand P, *et al.* Does branched-chain amino acid supplementation improve pulmonary rehabilitation effect in COPD? *Respir Med* 2021;**189**:106642. doi:10.1016/J.RMED.2021.106642

77 de Blok et al. BM, de Blok BMJ, de Greef MHG, *et al.* The effects of a lifestyle physical activity counseling program with feedback of a pedometer during pulmonary rehabilitation in patients with COPD: a pilot study. *Patient Educ Couns* 2006;**61**:48–55. doi:10.1016/j.pec.2005.02.005

78 De Godoy D V, De Godoy RF, Junior BB, *et al.* The effect of psychotherapy provided as part of a pulmonary rehabilitation program for the treatment of patients with chronic obstructive pulmonary disease. *J Bras Pneumol* 2005;**31**:499‐505.https://www.cochranelibrary.com/central/doi/10.1002/central/CN-00591997/full

79 de Lima F, Marçal Camillo CA, Grigoletto I, *et al.* Combining functional exercises with exercise training in COPD: a randomized controlled trial. *Physiother Theory Pract* 2022;**40**:1–10. doi:https://dx.doi.org/10.1080/09593985.2022.2148146

80 de Roos P, Lucas C, Strijbos JH, *et al.* Effectiveness of a combined exercise training and home-based walking programme on physical activity compared with standard medical care in moderate COPD: a randomised controlled trial. *Physiotherapy* 2018;**104**:116–21.http://ovidsp.ovid.com/ovidweb.cgi?T=JS&PAGE=reference&D=med15&NEWS=N&AN=28802772 %25[20170714//

81 de Sousa Pinto JM, Martin-Nogueras AM, Calvo-Arenillas JI, *et al.* Clinical benefits of home-based pulmonary rehabilitation in patients with chronic obstructive pulmonary disease. *J Cardiopulm Rehabil Prev* 2014;**34**:355–9. doi:10.1097/HCR.0000000000000061

82 Deacon SJ, Vincent EE, Greenhaff PL, *et al.* Randomized controlled trial of dietary creatine as an adjunct therapy to physical training in chronic obstructive pulmonary disease. *Am J Respir Crit Care Med* 2008;**178**:233–9. doi:10.1164/rccm.200710-1508OC

83 Deering BM, Fullen B, Egan C, *et al.* Acupuncture as an adjunct to pulmonary rehabilitation. *J Cardiopulm Rehabil Prev* 2011;**31**:392–9. doi:10.1097/HCR.0b013e31822f0f61

84 Dekhuijzen PNR, Beek MML, Folgering HTM, *et al.* Psychological changes during pulmonary rehabilitation and target-flow inspiratory muscle training in COPD patients with a ventilatory limitation during exercise. *Int J Rehabil Res* 1990;**13**:109–17. doi:10.1097/00004356-199006000-00002

85 Deniz S, Tuncel S, Gurgun A, *et al.* Adding Non-Invasive Positive Pressure Ventilation to Supplemental Oxygen During Exercise Training in Severe Chronic Obstructive Pulmonary Disease: A Randomized Controlled Study. *Thorac Res Pract* 2023;**24**:262–9. doi:https://dx.doi.org/10.5152/ThoracResPract.2023.23040

86 Dodia B, Gaikwad P, Shetye J, *et al.* Effect of unsupported upper limb endurance training versus lower limb endurance training in patients with chronic obstructive pulmonary disease. *Indian J Physiother Occup Ther* 2012;**6**:38–43.http://www.indianjournals.com/ijor.aspx?target=ijor:ijpot&volume=6&issue=4&article=008&type=pdf http://ovidsp.ovid.com/ovidweb.cgi?T=JS&PAGE=reference&D=emca2&NEWS=N&AN=368958883

87 Dolmage TE, Goldstein RS. Effects of one-legged exercise training of patients with COPD. *Chest* 2008;**133**:370–6. doi:10.1378/chest.07-1423

88 Donesky-Cuenco D, Nguyen HQ, Paul S, *et al.* Yoga therapy decreases dyspnea-related distress and improves functional performance in people with chronic obstructive pulmonary disease: a pilot study. *J Altern Complement Med* 2009;**15**:225–34. doi:10.1089/acm.2008.0389

89 Dong X, Wang X, Jia N, *et al.* A comparison between Qigong exercise and cycle ergometer exercise for the rehabilitation of chronic obstructive pulmonary disease: A pilot randomized controlled trial (CONSORT). *Medicine (Baltimore)* 2021;**100**:E26010. doi:10.1097/MD.0000000000026010

90 Dourado VZ, Tanni SE, Antunes LCO, *et al.* Effect of three exercise programs on patients with chronic obstructive pulmonary disease. *Brazilian J Med Biol Res = Rev Bras Pesqui medicas e Biol* 2009;**42**:263–71.

91 Du S, Ding D, Wang C, *et al.* The effect of tai chi chuan on the exercise tolerance and lung function among COPD patients. *Chinese J Rehabil Med* 2013;**28**:374‐376.https://www.cochranelibrary.com/central/doi/10.1002/central/CN-00991791/full

92 Duan W, Zeng D, Huang J, *et al.* Effect of modified Total Body Recumbent Stepper training on exercise capacity and thioredoxin in COPD: a randomized clinical trial. *Sci Rep* 2022;**12**:11139. doi:https://dx.doi.org/10.1038/s41598-022-15466-2

93 Duruturk N, Arıkan H, Ulubay G, *et al.* A comparison of calisthenic and cycle exercise training in chronic obstructive pulmonary disease patients: a randomized controlled trial. *Expert Rev Respir Med* 2016;**10**:99–108. doi:10.1586/17476348.2015.1126419

94 Effing T, Zielhuis G, Kerstjens H, *et al.* Community based physiotherapeutic exercise in COPD self-management: a randomised controlled trial. *Respir Med* 2011;**105**:418–26. doi:10.1016/j.rmed.2010.09.017

95 Elci A, Borekci S, Ovayolu N, *et al.* The efficacy and applicability of a pulmonary rehabilitation programme for patients with COPD in a secondary-care community hospital. *Respirology* 2008;**13**:703–7. doi:10.1111/j.1440-1843.2008.01327.x

96 Elliott M, Watson C, Wilkinson E, *et al.* Short- and long-term hospital and community exercise programmes for patients with chronic obstructive pulmonary disease. *Respirology* 2004;**9**:345–51. doi:10.1111/j.1440-1843.2004.00595.x

97 Elmorshidy BES, Elkholy MGA, Elsaadany HM, *et al.* Effect of pulmonary rehabilitation programme including either O2 inhalation or noninvasive ventilation in patients with chronic obstructive pulmonary disease. *Can J Respir Ther CJRT = Rev Can la Ther Respir RCTR* 2023;**59**:45–51. doi:https://dx.doi.org/10.29390/cjrt-2022-051

98 Elmorsi AS, Eldesoky ME, Mohsen MAA, *et al.* Effect of inspiratory muscle training on exercise performance and quality of life in patients with chronic obstructive pulmonary disease. *Egypt J Chest Dis Tuberc* 2016;**65**:41–6.http://www.elsevier.com/journals/egyptian-journal-of-chest-disease-and-tuberculosis/0422-7638 http://ovidsp.ovid.com/ovidweb.cgi?T=JS&PAGE=reference&D=emed17&NEWS=N&AN=607924692

99 Emery CF, Schein RL, Hauck ER, *et al.* Psychological and cognitive outcomes of a randomized trial of exercise among patients with chronic obstructive pulmonary disease. *Health Psychol* 1998;**17**:232–40. doi:10.1037//0278-6133.17.3.232

100 Emtner M, Porszasz J, Burns M, *et al.* Benefits of supplemental oxygen in exercise training in nonhypoxemic chronic obstructive pulmonary disease patients. *Am J Respir Crit Care Med* 2003;**168**:1034–42. doi:10.1164/rccm.200212-1525OC

101 Engel RM, Gonski P, Beath K, *et al.* Medium term effects of including manual therapy in a pulmonary rehabilitation program for chronic obstructive pulmonary disease (COPD): a randomized controlled pilot trial. *J Man Manip Ther* 2016;**24**:80–9.http://www.tandfonline.com/loi/yjmt20#.VwHawE1f1Qs http://ovidsp.ovid.com/ovidweb.cgi?T=JS&PAGE=reference&D=emed17&NEWS=N&AN=611771713

102 Engstrom CP, Persson LO, Larsson S, *et al.* Long-term effects of a pulmonary rehabilitation programme in outpatients with chronic obstructive pulmonary disease: a randomized controlled study. *Scand J Rehabil Med* 1999;**31**:207–13.http://ovidsp.ovid.com/ovidweb.cgi?T=JS&PAGE=reference&D=med4&NEWS=N&AN=10599897

103 Eves ND, Sandmeyer LC, Wong EY, *et al.* Helium-hyperoxia: a novel intervention to improve the benefits of pulmonary rehabilitation for patients with COPD. *Chest* 2009;**135**:609–18. doi:10.1378/chest.08-1517

104 Faager G, Soderlund K, Skold CM, *et al.* Creatine supplementation and physical training in patients with COPD: a double blind, placebo-controlled study. *Int J Chron Obstruct Pulmon Dis* 2006;**1**:445–53.

105 Fanfa Bordin D, Machado Cardoso D, Wagner LE, *et al.* Sternocleidomastoid muscle activation following inspiratory muscle training in patients with chronic obstructive pulmonary disease: a randomized clinical trial. *Fisioter e Pesqui* 2020;**27**:133–9.https://search.ebscohost.com/login.aspx?direct=true&AuthType=sso&db=cin20&AN=145169853&site=ehost-live&custid=ns124898

106 Farias CC, Resqueti V, Dias FAL, *et al.* Costs and benefits of Pulmonary Rehabilitation in Chronic Obstructive Pulmonary Disease: A randomized controlled trial. *Brazilian J Phys Ther* 2014;**18**:165–73. doi:10.1590/S1413-35552012005000151

107 Farver-Vestergaard I, O’Toole MS, O’Connor M, *et al.* Mindfulness-based cognitive therapy in COPD: a cluster randomised controlled trial. *Eur Respir J* 2018;**51**.http://ovidsp.ovid.com/ovidweb.cgi?T=JS&PAGE=reference&D=med15&NEWS=N&AN=29386337 %25[20180131//

108 Felcar JM, Probst VS, de Carvalho DR, *et al.* Effects of exercise training in water and on land in patients with COPD: a randomised clinical trial. *Physiotherapy* 2018;**104**:408–16.http://ovidsp.ovid.com/ovidweb.cgi?T=JS&PAGE=reference&D=med15&NEWS=N&AN=30477678 %25[20180301//

109 de Castro LA, Felcar JM, de Carvalho DR, *et al.* Effects of land- and water-based exercise programmes on postural balance in individuals with COPD: additional results from a randomised clinical trial. *Physiotherapy* 2020;**107**:58–65.http://ovidsp.ovid.com/ovidweb.cgi?T=JS&PAGE=reference&D=med17&NEWS=N&AN=32026836 %25[20190806//

110 Ferreira G, Araujo Z, Silva I, *et al.* Effectiveness of low-intensity aquatic exercise on COPD: A randomized clinical trial. *Eur Respir J* 2013;**42**.http://erj.ersjournals.com/content/42/Suppl_57/P3564.full.pdf+html?sid=b46d140c-0813-4257-8be8-c9c4c5664db0 http://ovidsp.ovid.com/ovidweb.cgi?T=JS&PAGE=reference&D=emed14&NEWS=N&AN=71840765

111 Fichter J, Fleckenstein J, Stahl C, *et al.* [Effect of oxygen (FI02: 0.35) on the aerobic capacity in patients with COPD]. *Einfluss von Sauerst (FIO2 035) auf die aerobe Belastbarkeit bei Patienten mit COPD* 1999;**53**:121–6.http://ovidsp.ovid.com/ovidweb.cgi?T=JS&PAGE=reference&D=med4&NEWS=N&AN=10226473

112 Finnegan SL, Harrison OK, Booth S, *et al.* The effect of d-cycloserine on brain processing of breathlessness over pulmonary rehabilitation: an experimental medicine study. *ERJ open Res* 2023;**9**. doi:https://dx.doi.org/10.1183/23120541.00479-2022

113 Finnerty JP, Keeping I, Bullough I, *et al.* The effectiveness of outpatient pulmonary rehabilitation in chronic lung disease: a randomized controlled trial. *Chest* 2001;**119**:1705–10.

114 Freire APCF, Marcal Camillo CA, de Alencar Silva BS, *et al.* Resistance training using different elastic components offers similar gains on muscle strength to weight machine equipment in Individuals with COPD: A randomized controlled trial. *Physiother Theory Pract* 2022;**38**:14–27.http://ovidsp.ovid.com/ovidweb.cgi?T=JS&PAGE=reference&D=medl&NEWS=N&AN=31975638 %25[20200124//

115 Fuld JP, Kilduff LP, Neder JA, *et al.* Creatine supplementation during pulmonary rehabilitation in chronic obstructive pulmonary disease. *Thorax* 2005;**60**:531–7. doi:10.1136/thx.2004.030452

116 Gadesha A, Bhise A. Effect of unsupported upper extremity exercise training on symptoms & quality of life in patients with COPD. *Indian J Physiother Occup Ther* 2015;**9**:146–51.http://indianjournals.com/ijor.aspx?target=ijor:ijpot&volume=9&issue=4&article=028&type=pdf http://ovidsp.ovid.com/ovidweb.cgi?T=JS&PAGE=reference&D=emca3&NEWS=N&AN=606282048

117 Gallo-Silva B, Cerezer-Silva V, Ferreira DG, *et al.* Effects of Water-Based Aerobic Interval Training in Patients With COPD: A RANDOMIZED CONTROLLED TRIAL. *J Cardiopulm Rehabil Prev* 2019;**39**:105–11.http://ovidsp.ovid.com/ovidweb.cgi?T=JS&PAGE=reference&D=med16&NEWS=N&AN=30720640

118 Gamper E, Schmidt U, Bansi J, *et al.* Outdoor Walking Training Compared To Cycle Ergometer Training in Severe COPD: A Randomized Controlled Feasibility Trial. *COPD* 2019;**16**:37–44.http://ovidsp.ovid.com/ovidweb.cgi?T=JS&PAGE=reference&D=med16&NEWS=N&AN=31056954 %25[20190506//

119 Garrod R, Mikelsons C, Paul EA, *et al.* Randomized controlled trial of domiciliary noninvasive positive pressure ventilation and physical training in severe chronic obstructive pulmonary disease. *Am J Respir Crit Care Med* 2000;**162**:1335–41. doi:10.1164/ajrccm.162.4.9912029

120 Gayle RC, Spitler DL, Karper WB, *et al.* Psychological changes in exercising COPD patients. *Int J Rehabil Res* 1988;**11**:335‐342.https://www.cochranelibrary.com/central/doi/10.1002/central/CN-00493670/full

121 Geidl W, Carl J, Schuler M, *et al.* Long-Term Benefits of Adding a Pedometer to Pulmonary Rehabilitation for COPD: The Randomized Controlled STAR Trial. *Int J Chron Obstruct Pulmon Dis* 2021;**16**:1977–88.http://ovidsp.ovid.com/ovidweb.cgi?T=JS&PAGE=reference&D=med19&NEWS=N&AN=34239299 %25[20210702//

122 Gianjoppe-Santos J, Barusso-Gruninger M, Pires Di Lorenzo VA. Effects of low and high resistance training intensities on clinical outcomes in patients with COPD - a randomized trial. *Physiother Theory Pract* 2022;**38**:1–12. doi:https://dx.doi.org/10.1080/09593985.2021.1929616

123 Gimenez M, Servera E, Vergara P, *et al.* Endurance training in patients with chronic obstructive pulmonary disease: a comparison of high versus moderate intensity. *Arch Phys Med Rehabil* 2000;**81**:102–9.

124 Gloeckl R, Heinzelmann I, Baeuerle S, *et al.* Effects of whole body vibration in patients with chronic obstructive pulmonary disease--a randomized controlled trial. *Respir Med* 2012;**106**:75–83. doi:10.1016/j.rmed.2011.10.021

125 Gloeckl R, Jarosch I, Bengsch U, *et al.* What’s the secret behind the benefits of whole-body vibration training in patients with COPD? A randomized, controlled trial. *Respir Med* 2017;**126**:17–24.http://ovidsp.ovid.com/ovidweb.cgi?T=JS&PAGE=reference&D=med14&NEWS=N&AN=28427544 %25[20170314//

126 Gloeckl R, Heinzelmann I, Kenn K. Whole body vibration training in patients with COPD. *Chron Respir Dis* 2015;**12**:212–21. doi:10.1177/1479972315583049

127 Göhl O, Linz H, Schönleben T, *et al.* Effekte eines multimodularen ambulanten trainingsprogramms für patienten mit COPD. *Pneumologie* 2006;**60**:529–36. doi:10.1055/s-2006-944235

128 Gouzi F, Maury J, Heraud N, *et al.* Additional Effects of Nutritional Antioxidant Supplementation on Peripheral Muscle during Pulmonary Rehabilitation in COPD Patients: A Randomized Controlled Trial. *Oxid Med Cell Longev* 2019;**2019**:5496346. doi:10.1155/2019/5496346

129 Griffiths TL, Burr ML, Campbell IA, *et al.* Results at 1 year of outpatient multidisciplinary pulmonary rehabilitation: a randomised controlled trial. *Lancet (London, England)* 2000;**355**:362–8.

130 Guell R, Casan P, Belda J, *et al.* Long-term effects of outpatient rehabilitation of COPD: A randomized trial. *Chest* 2000;**117**:976–83.

131 Guell R, Resqueti V, Sangenis M, *et al.* Impact of pulmonary rehabilitation on psychosocial morbidity in patients with severe COPD. *Chest* 2006;**129**:899–904. doi:10.1378/chest.129.4.899

132 Guell MR, Lucas de P, Galdiz JB, *et al.* Home versus hospital-based pulmonary rehabilitation for patients with chronic obstructive pulmonary disease: a Spanish multicenter trial. *Arch Bronconeumol* 2008;**44**:512‐518. doi:10.1157/13126830

133 Hansen H, Bieler T, Beyer N, *et al.* Supervised pulmonary tele-rehabilitation versus pulmonary rehabilitation in severe COPD: a randomised multicentre trial. *Thorax* 2020;**75**:413–21. doi:10.1136/thoraxjnl-2019-214246

134 Godtfredsen N, Frølich A, Bieler T, *et al.* 12-months follow-up of pulmonary tele-rehabilitation versus standard pulmonary rehabilitation: A multicentre randomised clinical trial in patients with severe COPD. *Respir Med* 2020;**172**:106129. doi:10.1016/J.RMED.2020.106129

135 Hasegawa M, Dobashi K, Horie T, *et al.* Influence of inhaled procaterol on pulmonary rehabilitation in chronic obstructive pulmonary disease. *Respir Investig* 2012;**50**:135–9.http://ovidsp.ovid.com/ovidweb.cgi?T=JS&PAGE=reference&D=emed13&NEWS=N&AN=366182195

136 Haugen TS, Stavem K. Rehabilitation in a warm versus a colder climate in chronic obstructive pulmonary disease: a randomized study. *J Cardiopulm Rehabil Prev* 2007;**27**:50–6.

137 Hawkins P, Johnson LC, Nikoletou D, *et al.* Proportional assist ventilation as an aid to exercise training in severe chronic obstructive pulmonary disease. *Thorax* 2002;**57**:853–9. doi:10.1136/thorax.57.10.853

138 He GX, Li N, Ren L, *et al.* Benefits of different intensities of pulmonary rehabilitation for patients with moderate-to-severe COPD according to the GOLD stage: a prospective, multicenter, single-blinded, randomized, controlled trial. *Int J Chron Obstruct Pulmon Dis* 2019;**14**:2291–304. doi:10.2147/COPD.S214836

139 Hernandez MTE, Rubio TM, Ortega Ruiz F, *et al.* Results of a home-based training program for patients with COPD. *Chest* 2000;**118**:106–14. doi:10.1378/chest.118.1.106

140 Ho C-F, Maa S-H, Shyu Y-IL, *et al.* Effectiveness of paced walking to music at home for patients with COPD. *COPD* 2012;**9**:447–57. doi:10.3109/15412555.2012.685664

141 Hoff J, Tjknna AE, Steinshamn S, *et al.* Maximal Strength Training of the Legs in COPD : A Therapy for Mechanical Inefficiency. *Med Sci Sport Exerc* 2007;**39**:220–6. doi:10.1249/01.mss.0000246989.48729.39

142 Holland A, Hill CJ, Nehez E, *et al.* Does unsupported upper limb exercise training improve symptoms and quality of life for patients with chronic obstructive pulmonary disease? *J Cardiopulm Rehabil* 2004;**24**:422–7.http://ovidsp.ovid.com/ovidweb.cgi?T=JS&PAGE=reference&D=med5&NEWS=N&AN=15632778

143 Holland AE, Mahal A, Hill CJ, *et al.* Home-based rehabilitation for COPD using minimal resources: a randomised, controlled equivalence trial. *Thorax* 2017;**72**:57–65. doi:10.1136/THORAXJNL-2016-208514

144 Wageck B, Cox NS, McDonald CF, *et al.* The Impact of COPD Exacerbations in the Year Following Pulmonary Rehabilitation: Secondary Analysis of a Randomised Controlled Trial. *Int J Chron Obstruct Pulmon Dis* 2020;**15**:3423–31.http://ovidsp.ovid.com/ovidweb.cgi?T=JS&PAGE=reference&D=med18&NEWS=N&AN=33408472 %25[20201231//

145 Burge AT, Palarea-Albaladejo J, Holland A, *et al.* The Impact of Pulmonary Rehabilitation on 24-Hour Movement Behavior in People With Chronic Obstructive Pulmonary Disease: New Insights From a Compositional Perspective. *J Phys Act Health* 2021;**18**:13–20.http://ovidsp.ovid.com/ovidweb.cgi?T=JS&PAGE=reference&D=med18&NEWS=N&AN=33307537 %25[20201211//

146 Horton EJ, Mitchell KE, Johnson-Warrington V, *et al.* Comparison of a structured home-based rehabilitation programme with conventional supervised pulmonary rehabilitation: A randomised non-inferiority trial. *Thorax* 2018;**73**:29–36. doi:10.1136/thoraxjnl-2016-208506

147 Iepsen UW, Munch GDW, Rugbjerg M, *et al.* Effect of endurance versus resistance training on quadriceps muscle dysfunction in COPD: A pilot study. *Int J COPD* 2016;**11**:2659–69. doi:10.2147/COPD.S114351

148 Inostroza M, Valdes O, Tapia G, *et al.* Effects of eccentric vs concentric cycling training on patients with moderate COPD. *Eur J Appl Physiol* 2022;**122**:489–502. doi:https://dx.doi.org/10.1007/s00421-021-04850-x

149 Janaudis-Ferreira T, Hill K, Goldstein RS, *et al.* Resistance arm training in patients with COPD: A Randomized Controlled Trial. *Chest* 2011;**139**:151–8. doi:10.1378/chest.10-1292

150 Jang HJ, Jung YK. The effects of self-efficacy promoting pulmonary rehabilitation program in out-patients with chronic obstructive pulmonary disease. *Tuberc Respir Dis (Seoul)* 2006;**61**:533–46.http://www.e-trd.org/index.php?body=current http://ovidsp.ovid.com/ovidweb.cgi?T=JS&PAGE=reference&D=emed9&NEWS=N&AN=46464931

151 Johnson JE, Gavin DJ, Adams-Dramiga S. Effects of training with heliox and noninvasive positive pressure ventilation on exercise ability in patients with severe COPD. *Chest* 2002;**122**:464–72. doi:10.1378/chest.122.2.464

152 Jolly E, Sívori M, Villarreal S, *et al.* [Home-based versus ambulatory hospital-based training in COPD]. *Med* 2014;**74**:293–300. doi:10.1021/la8010458

153 Kantatong T, Panpanich R, Deesomchok A, *et al.* Effects of the tai chi qigong programme on functional capacity, and lung function in chronic obstructive pulmonary disease patients: A ramdomised controlled trial. *J Tradit Complement Med* 2020;**10**:354–9.http://ovidsp.ovid.com/ovidweb.cgi?T=JS&PAGE=reference&D=pmnm5&NEWS=N&AN=32695652 %25[20190401//

154 Karagiannis C, Savva C, Korakakis V, *et al.* Effect of Strength Versus Strength and Endurance Upper Limb Exercise Training in Patients With Chronic Obstructive Pulmonary Disease: A RANDOMIZED CLINICAL TRIAL. *J Cardiopulm Rehabil Prev* 2021;**41**:426–31.http://ovidsp.ovid.com/ovidweb.cgi?T=JS&PAGE=reference&D=medl&NEWS=N&AN=34117184

155 Karapolat H, Atasever A, Atamaz F, *et al.* Do the benefits gained using a short-term pulmonary rehabilitation program remain in COPD patients after participation? *Lung* 2007;**185**:221–5. doi:10.1007/s00408-007-9011-4

156 Kawagoshi A, Kiyokawa N, Sugawara K, *et al.* Effects of low-intensity exercise and home-based pulmonary rehabilitation with pedometer feedback on physical activity in elderly patients with chronic obstructive pulmonary disease. *Respir Med* 2015;**109**:364–71. doi:10.1016/j.rmed.2015.01.008

157 Kaya M, Gurses HN, Ucgun H, *et al.* Effects of creative dance on functional capacity, pulmonary function, balance, and cognition in COPD patients: A randomized controlled trial. *Heart Lung* 2023;**58**:13–20. doi:https://dx.doi.org/10.1016/j.hrtlng.2022.10.017

158 Kayahana B, Karapolatb H, Atyntopraka E, *et al.* Psychological outcomes of an outpatient pulmonary rehabilitation program in patients with chronic obstructive pulmonary disease. *Respir Med* 2006;**100**:1050–7.http://ovidsp.ovid.com/ovidweb.cgi?T=JS&PAGE=reference&D=med6&NEWS=N&AN=16253496 %25[20051025//

159 Kilic B, Cicek HS, Avci MZ. Comparing the effects of self-management and hospital-based pulmonary rehabilitation programs in COPD patients. *Niger J Clin Pract* 2021;**24**:362–8. doi:10.4103/njcp.njcp_165_20

160 Klijn P, Van Stel H, Van Keimpema T, *et al.* Non-linear exercise training is the preferred training method in patients with severe COPD. *Eur Respir J* 2011;**38**.http://erj.ersjournals.com/content/38/Suppl_55/1454 http://ovidsp.ovid.com/ovidweb.cgi?T=JS&PAGE=reference&D=emed12&NEWS=N&AN=72116555

161 Kohlbrenner D, Kuhn M, Manettas A, *et al.* Low-load blood-flow restriction strength training in patients with COPD: a randomised controlled pilot study. *Swiss Med Wkly* 2023;**153**:132S.https://www.smw.ch/index.php/smw/article/view/3340/5703 http://ovidsp.ovid.com/ovidweb.cgi?T=JS&PAGE=reference&D=emexb&NEWS=N&AN=641943437

162 Kongsgaard M, Backer V, Jorgensen K, *et al.* Heavy resistance training increases muscle size, strength and physical function in elderly male COPD-patients--a pilot study. *Respir Med* 2004;**98**:1000–7.

163 Kostrzon M, Sliwka A, Wloch T, *et al.* Subterranean Pulmonary Rehabilitation in Chronic Obstructive Pulmonary Disease. *Adv Exp Med Biol* 2019;**1176**:35–46.http://ovidsp.ovid.com/ovidweb.cgi?T=JS&PAGE=reference&D=med16&NEWS=N&AN=30980315

164 Kwon H, Lee S, Jung EJ, *et al.* An mHealth Management Platform for Patients with Chronic Obstructive Pulmonary Disease (efil breath): Randomized Controlled Trial. *JMIR mHealth uHealth* 2018;**6**:e10502.http://ovidsp.ovid.com/ovidweb.cgi?T=JS&PAGE=reference&D=pmnm4&NEWS=N&AN=30143475 %25[20180824//

165 Lahham A, McDonald CF, Moore R, *et al.* The impact of home-based pulmonary rehabilitation on people with mild chronic obstructive pulmonary disease: A randomised controlled trial. *Clin Respir J* 2020;**14**:335–44.http://ovidsp.ovid.com/ovidweb.cgi?T=JS&PAGE=reference&D=med17&NEWS=N&AN=31880078 %25[20200111//

166 Lake FR, Henderson K, Briffa T, *et al.* Upper-limb and lower-limb exercise training in patients with chronic airflow obstruction. *Chest* 1990;**97**:1077–82. doi:10.1378/chest.97.5.1077

167 Larson JL, Covey MK, Wirtz SE, *et al.* Cycle ergometer and inspiratory muscle training in chronic obstructive pulmonary disease. *Am J Respir Crit Care Med* 1999;**160**:500–7. doi:10.1164/ajrccm.160.2.9804067

168 Laviolette L, Lands LC, Dauletbaev N, *et al.* Combined effect of dietary supplementation with pressurized whey and exercise training in chronic obstructive pulmonary disease: a randomized, controlled, double-blind pilot study. *J Med Food* 2010;**13**:589–98. doi:10.1089/jmf.2009.0142

169 Lee SW, Park JJ, Lyu YR, *et al.* The effect of lung-conduction exercise in chronic obstructive pulmonary disease Randomized, assessor-blind, multicenter pilot trial. *Medicine (Baltimore)* 2022;**101**:e28629. doi:https://dx.doi.org/10.1097/MD.0000000000028629

170 Leung RWM, Alison JA, McKeough ZJ, *et al.* Ground walk training improves functional exercise capacity more than cycle training in people with chronic obstructive pulmonary disease (COPD): a randomised trial. *J Physiother* 2010;**56**:105–12.

171 Leung RWM, McKeough ZJ, Peters MJ, *et al.* Short-form Sun-style t’ai chi as an exercise training modality in people with COPD. *Eur Respir J* 2013;**41**:1051–7. doi:10.1183/09031936.00036912

172 Li P, Liu J, Lu Y, *et al.* Effects of long-term home-based Liuzijue exercise combined with clinical guidance in elderly patients with chronic obstructive pulmonary disease. *Clin Interv Aging* 2018;**13**:1391–9.http://ovidsp.ovid.com/ovidweb.cgi?T=JS&PAGE=reference&D=med15&NEWS=N&AN=30122911 %25[20180803//

173 Liddell F, Webber J. Pulmonary rehabilitation for chronic obstructive pulmonary disease: a pilot study evaluating a once-weekly versus twice-weekly supervised programme. *Physiotherapy* 2010;**96**:68–74. doi:10.1016/j.physio.2009.04.007

174 Lin F-L, Yeh M-L. Walking and mindfulness improve the exercise capacity of patients with chronic obstructive pulmonary disease: A randomised controlled trial. *Clin Rehabil* 2021;**35**:1117–25.http://ovidsp.ovid.com/ovidweb.cgi?T=JS&PAGE=reference&D=med19&NEWS=N&AN=33706570 %25[20210311//

175 Liu W-T, Wang C-H, Lin H-C, *et al.* Efficacy of a cell phone-based exercise programme for COPD. *Eur Respir J* 2008;**32**:651–9. doi:10.1183/09031936.00104407

176 Liu K, Yu X, Cui X, *et al.* Effects of Proprioceptive Neuromuscular Facilitation Stretching Combined with Aerobic Training on Pulmonary Function in COPD Patients: A Randomized Controlled Trial. *Int J Chron Obstruct Pulmon Dis* 2021;**16**:969–77.http://ovidsp.ovid.com/ovidweb.cgi?T=JS&PAGE=reference&D=med19&NEWS=N&AN=33880021 %25[20210413//

177 Liu X, Wu W, Li N, *et al.* Effects of water-based Liuzijue exercise on peak exercise capacity, functional exercise capacity, and quality of life in people with COPD. *Clin Respir J* 2021;**15**:956–66.http://ovidsp.ovid.com/ovidweb.cgi?T=JS&PAGE=reference&D=med19&NEWS=N&AN=33998778 %25[20210614//

178 Wu W, Liu X, Liu J, *et al.* Effectiveness of water-based Liuzijue exercise on respiratory muscle strength and peripheral skeletal muscle function in patients with COPD. *Int J Chron Obstruct Pulmon Dis* 2018;**13**:1713–26.http://ovidsp.ovid.com/ovidweb.cgi?T=JS&PAGE=reference&D=med15&NEWS=N&AN=29872289 %25[20180525//

179 Liu J, Sun CH, Zhao Y, *et al.* [The application value of chronic disease self-management project in promoting pulmonary rehabilitation of patients with chronic obstructive pulmonary disease]. *Zhonghua Lao Dong Wei Sheng Zhi Ye Bing Za Zhi* 2021;**39**:37–40.http://ovidsp.ovid.com/ovidweb.cgi?T=JS&PAGE=reference&D=med18&NEWS=N&AN=33535338

180 Liu W, Liu X-MM, Huang Y-LL, *et al.* Tai Chi as a complementary exercise for pulmonary rehabilitation in chronic obstructive pulmonary disease: A randomised controlled trial. *Complement Ther Med* 2023;**78**:102977. doi:https://dx.doi.org/10.1016/j.ctim.2023.102977

181 Louvaris Z, Spetsioti S, Kortianou EA, *et al.* Interval training induces clinically meaningful effects in daily activity levels in COPD. *Eur Respir J* 2016;**48**:567–70. doi:10.1183/13993003.00679-2016

182 Lum CM, Woo J, Yeung F, *et al.* Semi-supervised, domiciliary pulmonary rehabilitation programme: A controlled clinical trial. *Hong Kong Med J* 2007;**13**:42–5.http://www.hkmj.org/system/files/hkm0710sp5p42.pdf http://ovidsp.ovid.com/ovidweb.cgi?T=JS&PAGE=reference&D=emed10&NEWS=N&AN=610342985

183 Ma Y, Chen Y, Zhang N, *et al.* Efficacy and safety of pulmonary rehabilitation training on lung function, quality of life, and T cell immune function in patients with stable chronic obstructive pulmonary disease: a randomized controlled trial. *Ann Palliat Med* 2022;**11**:1774–85. doi:https://dx.doi.org/10.21037/apm-22-451

184 Maddocks M, Nolan CM, Man WDC, *et al.* Neuromuscular electrical stimulation to improve exercise capacity in patients with severe COPD: a randomised double-blind, placebo-controlled trial. *Lancet Respir Med* 2016;**4**:27–36.http://ovidsp.ovid.com/ovidweb.cgi?T=JS&PAGE=reference&D=med13&NEWS=N&AN=26701362 %25[20151215//

185 Mador MJ, Bozkanat E, Aggarwal A, *et al.* Endurance and strength training in patients with COPD. *Chest* 2004;**125**:2036–45.

186 Mador MJ, Deniz O, Aggarwal A, *et al.* Effect of respiratory muscle endurance training in patients with COPD undergoing pulmonary rehabilitation. *Chest* 2005;**128**:1216–24. doi:10.1378/chest.128.3.1216

187 Mador MJ, Krawza M, Alhajhusian A, *et al.* Interval training versus continuous training in patients with chronic obstructive pulmonary disease. *J Cardiopulm Rehabil Prev* 2009;**29**:126–32. doi:10.1097/HCR.0b013e31819a024f

188 Maglakelidze M, Kurua I, Maglakelidze N, *et al.* Feasibility of a pulmonary rehabilitation programme for patients with symptomatic chronic obstructive pulmonary disease in Georgia: a single-site, randomised controlled trial from the Breathe Well Group. *BMJ Open* 2022;**12**:e056902. doi:https://dx.doi.org/10.1136/bmjopen-2021-056902

189 Majewska-Pulsakowska M, Wytrychowski K, Rożek-Piechura K. The role of inspiratory muscle training in the process of rehabilitation of patients with chronic obstructive pulmonary disease. In: *Advances in Experimental Medicine and Biology*. 2016. 47–51. doi:10.1007/5584_2015_194

190 Maltais F, Bourbeau J, Shapiro S, *et al.* Effects of home-based pulmonary rehabilitation in patients with chronic obstructive pulmonary disease: a randomized trial. *Ann Intern Med* 2008;**149**:869–78.

191 Mao L, Lu G, Wang L. Effects of combined aerobic and low-intensity resistance training on pulmonary function and motor ability in the old patients with COPD. *Chinese J Rehabil Med* 2018;**33**:928–33.http://www.rehabi.com.cn/ http://ovidsp.ovid.com/ovidweb.cgi?T=JS&PAGE=reference&D=emca3&NEWS=N&AN=623820330

192 Marques A, Jacome C, Cruz J, *et al.* Family-based pulmonary rehabilitation in COPD: A randomized controlled trial. *Eur Respir J* 2014;**44**.http://erj.ersjournals.com/content/44/Suppl_58/418.abstract?sid=f6e5253b-c4fb-4163-a830-ff6e2095f2ca http://ovidsp.ovid.com/ovidweb.cgi?T=JS&PAGE=reference&D=emed15&NEWS=N&AN=71848361

193 Marquis K, Maltais F, Lacasse Y, *et al.* Effects of aerobic exercise training and irbesartan on blood pressure and heart rate variability in patients with chronic obstructive pulmonary disease. *Can Respir J* 2008;**15**:355–60.

194 Marrara KT, Marino DM, de Held PA, *et al.* Different physical therapy interventions on daily physical activities in chronic obstructive pulmonary disease. *Respir Med* 2008;**102**:505–11. doi:10.1016/j.rmed.2007.12.004

195 Marrara KT, Marino DM, Jamami M, *et al.* Responsiveness of the six-minute step test to a physical training program in patients with COPD. *J Bras Pneumol publicacao Of da Soc Bras Pneumol e Tisilogia* 2012;**38**:579–87.

196 Marrara KT, Di Lorenzo VAP, Jaenisch RB, *et al.* Noninvasive Ventilation as an Important Adjunct to an Exercise Training Program in Subjects With Moderate to Severe COPD. *Respir Care* 2018;**63**:1388–98.http://ovidsp.ovid.com/ovidweb.cgi?T=JS&PAGE=reference&D=med15&NEWS=N&AN=29945905 %25[20180626//

197 McCarroll ML. *Exercise and airway clearing devices in pulmonary rehabilitation programs for patients with chronic obstructive pulmonary disease*. 2005.https://search.ebscohost.com/login.aspx?direct=true&AuthType=sso&db=cin20&AN=109845051&site=ehost-live&custid=ns124898

198 McFarland C, Willson D, Sloan J, *et al.* A randomized trial comparing 2 types of in-home rehabilitation for chronic obstructive pulmonary disease: a pilot study. *J Geriatr Phys Ther* 2012;**35**:132–9. doi:10.1519/JPT.0b013e31824145f5

199 McKeough ZJ, Bye PTP, Alison JA. Arm exercise training in chronic obstructive pulmonary disease: a randomised controlled trial. *Chron Respir Dis* 2012;**9**:153–62. doi:10.1177/1479972312440814

200 Mekki M, Paillard T, Sahli S, *et al.* Effect of adding neuromuscular electrical stimulation training to pulmonary rehabilitation in patients with chronic obstructive pulmonary disease: randomized clinical trial. *Clin Rehabil* 2019;**33**:195–206. doi:10.1177/0269215518791658

201 Migliore A. *Health-related quality of life, functional status, and exercise tolerance of adults with chronic obstructive pulmonary disease following three treatment situations: Exercise alone, exercise with a lecture series, and exercise with activity training*. 2003.https://search.ebscohost.com/login.aspx?direct=true&AuthType=sso&db=cin20&AN=109842712&site=ehost-live&custid=ns124898

202 Mitchell KE, Johnson-Warrington V, Apps LD, *et al.* A self-management programme for COPD: A randomised controlled trial. *Eur Respir J* 2014;**44**:1538–47. doi:10.1183/09031936.00047814

203 Mkacher W, Mekki M, Chaieb F, *et al.* Balance Training in Pulmonary Rehabilitation: EFFECTS ON PSYCHOSOCIAL OUTCOMES. *J Cardiopulm Rehabil Prev* 2015;**35**:278–85. doi:10.1097/HCR.0000000000000122

204 Mkacher W, Mekki M, Tabka Z, *et al.* Effect of 6 Months of Balance Training During Pulmonary Rehabilitation in Patients With COPD. *J Cardiopulm Rehabil Prev* 2015;**35**:207–13. doi:10.1097/HCR.0000000000000109

205 Moezy A, Erfani A, Mazaherinezhad A, *et al.* Downhill walking influence on physical condition and quality of life in patients with COPD: A randomized controlled trial. *Med J Islam Repub Iran* 2018;**32**:49. doi:10.14196/MJIRI.32.49

206 Mohamed A, Ismail A. Stress Axis Response to Aerobic Exercise in Chronic Obstructive Pulmonary Disease Patients. *Adv Rehabil* 2022;**36**:24–32. doi:https://dx.doi.org/10.5114/areh.2022.123180

207 Moore J, Fiddler H, Seymour J, *et al.* Effect of a home exercise video programme in patients with chronic obstructive pulmonary disease. *J Rehabil Med* 2009;**41**:195–200. doi:10.2340/16501977-0308

208 Nakamura Y, Tanaka K, Shigematsu R, *et al.* Effects of aerobic training and recreational activities in patients with chronic obstructive pulmonary disease. *Int J Rehabil Res Int Zeitschrift fur Rehabil Rev Int Rech Readapt* 2008;**31**:275–83. doi:10.1097/MRR.0b013e3282fc0f81

209 Nalbant O, Nur H, Ogus C, *et al.* Effects of long-term aerobic exercise program in chronic obstructive pulmonary disease. *Turkiye Fiz Tip ve Rehabil Derg* 2011;**57**:8–13.http://www.ftrdergisi.com/eng/sayilar/78/8-13.pdf http://ovidsp.ovid.com/ovidweb.cgi?T=JS&PAGE=reference&D=emed12&NEWS=N&AN=361649513

210 Naseer BA, Al-Shenqiti AM, Ali ARH, *et al.* Effect of a short term pulmonary rehabilitation programme on exercise capacity, pulmonary function and health related quality of life in patients with COPD. *J Taibah Univ Med Sci* 2017;**12**:471–6. doi:10.1016/J.JTUMED.2017.07.005

211 Nasis IG, Vogiatzis I, Stratakos G, *et al.* Effects of interval-load versus constant-load training on the BODE index in COPD patients. *Respir Med* 2009;**103**:1392–8. doi:10.1016/j.rmed.2009.03.003

212 Neder JA, Sword D, Ward SA, *et al.* Home based neuromuscular electrical stimulation as a new rehabilitative strategy for severely disabled patients with chronic obstructive pulmonary disease (COPD). *Thorax* 2002;**57**:333–7.

213 Neunhauserer D, Hudelmaier M, Niederseer D, *et al.* The Impact of Exercise Training and Supplemental Oxygen on Peripheral Muscles in COPD: A Randomized Controlled Trial. *Med Sci Sports Exerc* Published Online First: 2023. doi:https://dx.doi.org/10.1249/MSS.0000000000003268

214 Ng L, Chiang LK, Tang R, *et al.* Effectiveness of incorporating Tai Chi in a pulmonary rehabilitation program for Chronic Obstructive Pulmonary Disease (COPD) in primary care-A pilot randomized controlled trial. *Eur J Integr Med* 2014;**6**:248–58.http://shop.elsevier.de/artikel/1209755 http://ovidsp.ovid.com/ovidweb.cgi?T=JS&PAGE=reference&D=emed15&NEWS=N&AN=53017620

215 Nguyen HQ, Donesky-Cuenco D, Wolpin S, *et al.* Randomized controlled trial of an internet-based versus face-to-face dyspnea self-management program for patients with chronic obstructive pulmonary disease: pilot study. *J Med Internet Res* 2008;**10**:e9. doi:10.2196/jmir.990

216 Nguyen HQ, Donesky D, Reinke LF, *et al.* Internet-based dyspnea self-management support for patients with chronic obstructive pulmonary disease. *J Pain Symptom Manage* 2013;**46**:43–55. doi:10.1016/j.jpainsymman.2012.06.015

217 Ninot G, Moullec G, Picot MC, *et al.* Cost-saving effect of supervised exercise associated to COPD self-management education program. *Respir Med* 2011;**105**:377–85. doi:10.1016/j.rmed.2010.10.002

218 Niu R, He R, Luo B-L, *et al.* The effect of tai chi on chronic obstructive pulmonary disease: a pilot randomised study of lung function, exercise capacity and diaphragm strength. *Heart Lung Circ* 2014;**23**:347–52. doi:10.1016/j.hlc.2013.10.057

219 Nolan CM, Maddocks M, Canavan JL, *et al.* Pedometer Step Count Targets during Pulmonary Rehabilitation in Chronic Obstructive Pulmonary Disease. A Randomized Controlled Trial. *Am J Respir Crit Care Med* 2017;**195**:1344–52.http://ovidsp.ovid.com/ovidweb.cgi?T=JS&PAGE=reference&D=med14&NEWS=N&AN=27911566

220 Normandin EA, McCusker C, Connors M, *et al.* An evaluation of two approaches to exercise conditioning in pulmonary rehabilitation. *Chest* 2002;**121**:1085–91.http://ovidsp.ovid.com/ovidweb.cgi?T=JS&PAGE=reference&D=med4&NEWS=N&AN=11948036

221 Norweg AM, Whiteson J, Malgady R, *et al.* The effectiveness of different combinations of pulmonary rehabilitation program components: a randomized controlled trial. *Chest* 2005;**128**:663–72. doi:10.1378/chest.128.2.663

222 Nyberg A, Lindstrom B, Rickenlund A, *et al.* Low-load/high-repetition elastic band resistance training in patients with COPD: a randomized, controlled, multicenter trial. *Clin Respir J* 2015;**9**:278–88. doi:10.1111/crj.12141

223 Nyberg A, Martin M, Saey D, *et al.* Effects of Low-Load/High-Repetition Resistance Training on Exercise Capacity, Health Status, and Limb Muscle Adaptation in Patients With Severe COPD: A Randomized Controlled Trial. *Chest* 2021;**159**:1821–32.http://ovidsp.ovid.com/ovidweb.cgi?T=JS&PAGE=reference&D=med18&NEWS=N&AN=33316237 %25[20201213//

224 O’Hara WJ, Lasachuk KE, Matheson PC. Weight training and backpacking in chronic obstructive pulmonary disease. *Respir Care* 1984;**29**:1202–10.http://ovidsp.ovid.com/ovidweb.cgi?T=JS&PAGE=reference&D=emed3&NEWS=N&AN=15199207

225 Oliveira JC, Filho FSL, Sampaio L, *et al.* Outpatient vs. home-based pulmonary rehabilitation in COPD: A randomized controlled trial. *Eur Respir J* 2011;**38**.http://erj.ersjournals.com/content/38/Suppl_55/p4805 http://ovidsp.ovid.com/ovidweb.cgi?T=JS&PAGE=reference&D=emed12&NEWS=N&AN=72122299

226 Ortega F, Toral J, Cejudo P, *et al.* Comparison of effects of strength and endurance training in patients with chronic obstructive pulmonary disease. *Am J Respir Crit Care Med* 2002;**166**:669–74. doi:10.1164/rccm.2107081

227 Ozdemir EP, Solak O, Fidan F, *et al.* The effect of water-based pulmonary rehabilitation on anxiety and quality of life in chronic pulmonary obstructive disease patients. *Turkiye Klin J Med Sci* 2010;**30**:880–7.http://tipbilimleri.turkiyeklinikleri.com/download_pdf.php?id=58241 http://ovidsp.ovid.com/ovidweb.cgi?T=JS&PAGE=reference&D=emed22&NEWS=N&AN=359659491

228 Pan Y, Wang Z, Min J. The effect of 24 simplified Taichi on pulmonary rehabilitation in patients with stable chronic obstructive pulmonary disease. *Chinese J Rehabil Med* 2018;**33**:681–6.http://www.rehabi.com.cn/ http://ovidsp.ovid.com/ovidweb.cgi?T=JS&PAGE=reference&D=emca3&NEWS=N&AN=623117169

229 Pancera S, Buraschi R, Bianchi LNC, *et al.* Effectiveness of Continuous Chest Wall Vibration With Concurrent Aerobic Training on Dyspnea and Functional Exercise Capacity in Patients With Chronic Obstructive Pulmonary Disease: A Randomized Controlled Trial. *Arch Phys Med Rehabil* 2021;**102**:1457–64.http://ovidsp.ovid.com/ovidweb.cgi?T=JS&PAGE=reference&D=med19&NEWS=N&AN=33781780 %25[20210326//

230 Pancera S, Lopomo NF, Porta R, *et al.* Effects of Combined Endurance and Resistance Eccentric Training on Muscle Function and Functional Performance in Patients With Chronic Obstructive Pulmonary Disease: Randomized Controlled Trial. *Arch Phys Med Rehabil* 2023;**105**. doi:https://dx.doi.org/10.1016/j.apmr.2023.09.004

231 Park SK, Bang CH, Lee SH. Evaluating the effect of a smartphone app-based self-management program for people with COPD: A randomized controlled trial. *Appl Nurs Res* 2020;**52**:151231.http://ovidsp.ovid.com/ovidweb.cgi?T=JS&PAGE=reference&D=med17&NEWS=N&AN=31955942 %25[20200109//

232 Patrizio G, D’Andria M, D’Abrosca F, *et al.* Airway Clearance with Expiratory Flow Accelerator Technology: Effectiveness of the ‘Free Aspire’ Device in Patients with Severe COPD. *Turkish Thorac J* 2019;**20**:209–15. doi:10.5152/TURKTHORACJ.2018.18053

233 Paulin FV, Viana a, Zagatto AM, *et al.* Addition of vitamin B12 to exercise training improves cycle ergometer endurance in advanced COPD patients: A randomized and controlled study. *Respir Med* 2017;**122**:23–9. doi:10.1016/J.RMED.2016.11.015

234 Pavitt MJ, Tanner RJ, Lewis AP, *et al.* Oral dietary nitrate supplementation to enhance pulmonary rehabilitation in chronic obstructive pulmonary disease: A multi-centre, double blind, placebo-controlled, parallel group study. *Thorax* 2018;**73**:A3.http://ovidsp.ovid.com/ovidweb.cgi?T=JS&PAGE=reference&D=emed19&NEWS=N&AN=627695905

235 Paz-Diaz H, Montes de Oca M, Lopez JM, *et al.* Pulmonary rehabilitation improves depression, anxiety, dyspnea and health status in patients with COPD. *Am J Phys Med Rehabil* 2007;**86**:30–6.

236 Peran L, Castel M, Beaumont M. Effects of combined triceps surae and quadriceps neuromuscular electrostimulation in pulmonary rehabilitation for severe and very severe COPD. *Kinesitherapie* 2018;**18**:03-Dec.http://www.journals.elsevier.com/kinesitherapie-la-revue http://ovidsp.ovid.com/ovidweb.cgi?T=JS&PAGE=reference&D=emed19&NEWS=N&AN=2000495690

237 Peran L, Beaumont M, Le Ber C, *et al.* Effect of neuromuscular electrical stimulation on exercise capacity in patients with severe chronic obstructive pulmonary disease: A randomised controlled trial. *Clin Rehabil* 2022;**36**:1072–82. doi:https://dx.doi.org/10.1177/02692155221091802

238 Pereira AM, Santa-Clara H, Pereira E, *et al.* Impact of combined exercise on chronic obstructive pulmonary patients’ state of health. *Rev Port Pneumol* 2010;**16**:737–57.

239 Petersen AMW, Mittendorfer B, Magkos F, *et al.* Physical activity counteracts increased whole-body protein breakdown in chronic obstructive pulmonary disease patients. *Scand J Med Sci Sports* 2008;**18**:557–64. doi:10.1111/j.1600-0838.2007.00727.x

240 Pleguezuelos E, Perez ME, Guirao L, *et al.* Effects of whole body vibration training in patients with severe chronic obstructive pulmonary disease. *Respirology* 2013;**18**:1028–34. doi:10.1111/resp.12122

241 Polkey MI, Qiu Z-H, Zhou L, *et al.* Tai Chi and Pulmonary Rehabilitation Compared for Treatment-Naive Patients With COPD: A Randomized Controlled Trial. *Chest* 2018;**153**:1116–24.http://ovidsp.ovid.com/ovidweb.cgi?T=JS&PAGE=reference&D=med15&NEWS=N&AN=29625777 %25[20180403//

242 Pothasak Y, Leelarungrayub J, Natakankitkul S, *et al.* Prototype star fruit-honey product and effectiveness on antixidants, inflammation and walking distance in participants with stable chronic obstructive pulmonary disease (COPD). *Pharmacogn J* 2020;**12**:1121–34.http://phcogj.com/article/1228 http://ovidsp.ovid.com/ovidweb.cgi?T=JS&PAGE=reference&D=emed21&NEWS=N&AN=2007647111

243 Pradella CO, Belmonte GM, Maia MN, *et al.* Home-based pulmonary rehabilitation for subjects with COPD: A randomized study. *Respir Care* 2015;**60**:526–32. doi:10.4187/respcare.02994

244 Probst VS, Kovelis D, Hernandes NA, *et al.* Effects of 2 exercise training programs on physical activity in daily life in patients with COPD. *Respir Care* 2011;**56**:1799–807. doi:10.4187/respcare.01110

245 Puente-Maestu L, Sanz ML, Sanz P, *et al.* Comparison of effects of supervised versus self-monitored training programmes in patients with chronic obstructive pulmonary disease. *Eur Respir J* 2000;**15**:517–25. doi:10.1034/j.1399-3003.2000.15.15.x

246 Ramos EMC, de Toledo-Arruda AC, Fosco LC, *et al.* The effects of elastic tubing-based resistance training compared with conventional resistance training in patients with moderate chronic obstructive pulmonary disease: a randomized clinical trial. *Clin Rehabil* 2014;**28**:1096–106. doi:10.1177/0269215514527842

247 Rausch Osthoff A-KK, Beyer S, Gisi D, *et al.* Effect of counselling during pulmonary rehabilitation on self-determined motivation to be physically active for people with chronic obstructive pulmonary disease: a pragmatic RCT. *BMC Pulm Med* 2021;**21**:317. doi:https://dx.doi.org/10.1186/s12890-021-01685-2

248 Reardon J, Awad E, Normandin E, *et al.* The effect of comprehensive outpatient pulmonary rehabilitation on dyspnea. *Chest* 1994;**105**:1046–52. doi:10.1378/chest.105.4.1046

249 Reuveny R, Ben-Dov I, Gaides M, *et al.* Ventilatory support during training improves training benefit in severe chronic airway obstruction. *Isr Med Assoc J* 2005;**7**:151–5. doi:10.1097/00008483-200507000-00013

250 Ries AL, Kaplan RM, Limberg TM, *et al.* Effects of Pulmonary Rehabilitation on Physiologic and Psychosocial Outcomes in Patients with Chronic Obstructive Pulmonary Disease. *Ann Intern Med* 1995;**122**:823. doi:10.7326/0003-4819-122-11-199506010-00003

251 Rinaldo N, Bacchi E, Coratella G, *et al.* Effects of Combined Aerobic-Strength Training vs Fitness Education Program in COPD Patients. *Int J Sports Med* 2017;**38**:1001–8. doi:10.1055/S-0043-112339

252 Ringbaek TJ, Broendum E, Hemmingsen L, *et al.* Rehabilitation of patients with chronic obstructive pulmonary disease. Exercise twice a week is not sufficient! *Respir Med* 2000;**94**:150–4. doi:10.1053/rmed.1999.0704

253 Rizk AK, Wardini R, Chan-Thim E, *et al.* Acute responses to exercise training and relationship with exercise adherence in moderate chronic obstructive pulmonary disease. *Chron Respir Dis* 2015;**12**:329–39. doi:10.1177/1479972315598691

254 Roman M, Larraz C, Gomez A, *et al.* Efficacy of pulmonary rehabilitation in patients with moderate chronic obstructive pulmonary disease: a randomized controlled trial. *BMC Fam Pract* 2013;**14**:21. doi:10.1186/1471-2296-14-21

255 Salhi B, Malfait TJ, Van Maele G, *et al.* Effects of Whole Body Vibration in Patients With COPD. *COPD* 2015;**12**:525–32. doi:10.3109/15412555.2015.1008693

256 Salve VT, Atram JS. N-Acetylcysteine combined with home based physical activity: Effect on health related quality of life in stable COPD patients- a randomised controlled trial. *J Clin Diagnostic Res* 2016;**10**:OC16–9.http://www.jcdr.net/articles/PDF/8980/23668_CE[Ra1]_F(GH)_PF1(PI_RK)_PFA(AK)_PF2(PAG).pdf http://ovidsp.ovid.com/ovidweb.cgi?T=JS&PAGE=reference&D=emed17&NEWS=N&AN=613292002

257 Santos C, Rodrigues F, Santos J, *et al.* Pulmonary Rehabilitation in COPD: Effect of 2 Aerobic Exercise Intensities on Subject-Centered Outcomes--A Randomized Controlled Trial. *Respir Care* 2015;**60**:1603–9. doi:10.4187/respcare.03663

258 Schultz K, Jelusic D, Wittmann M, *et al.* Inspiratory muscle training does not improve clinical outcomes in 3-week COPD rehabilitation: results from a randomised controlled trial. *Eur Respir J* 2018;**51**.http://ovidsp.ovid.com/ovidweb.cgi?T=JS&PAGE=reference&D=med15&NEWS=N&AN=29371382 %25[20180125//

259 Scorsone D, Bartolini S, Saporiti R, *et al.* Does a low-density gas mixture or oxygen supplementation improve exercise training in COPD? *Chest* 2010;**138**:1133–9. doi:10.1378/chest.10-0120

260 Sewell L, Singh SJ, Williams JEA, *et al.* How long should outpatient pulmonary rehabilitation be? A randomised controlled trial of 4 weeks versus 7 weeks. *Thorax* 2006;**61**:767–71. doi:10.1136/thx.2005.048173

261 Sharifabad MA, Hurewitz A, Spiegler P, *et al.* Written disclosure therapy for patients with chronic lung disease undergoing pulmonary rehabilitation. *J Cardiopulm Rehabil Prev* 2010;**30**:340–5. doi:10.1097/HCR.0b013e3181e174c4

262 Shui L-L, Cai J-J, Zhong X-Q, *et al.* Chronic Obstructive Pulmonary Disease Patients With High Peripheral Blood Eosinophil Counts Have Better Predicted Improvement in 6MWD After Rehabilitation: A PRELIMINARY STUDY. *J Cardiopulm Rehabil Prev* 2023;**43**:122–8. doi:https://dx.doi.org/10.1097/HCR.0000000000000726

263 Silva AB, Di Lorenzo VAP, Jamami M, *et al.* Influence of oral L-carnitine supplementation combined with physical training on exercise tolerance in patients with chronic obstructive pulmonary disease. *J Pneumol* 2003;**29**:379‐385.https://www.cochranelibrary.com/central/doi/10.1002/central/CN-00690587/full

264 Silva MGF, es, Fern, *et al.* Oral supplementation of L-carnitine combined with exercise and respiratory training in patients with chronic obstructive pulmonary disease: preliminary study. *Fisioter e Pesqui* 2012;**19**:320–5.https://search.ebscohost.com/login.aspx?direct=true&AuthType=sso&db=cin20&AN=108028157&site=ehost-live&custid=ns124898

265 Silva CM da SE, Gomes Neto M, Saquetto MB, *et al.* Effects of upper limb resistance exercise on aerobic capacity, muscle strength, and quality of life in COPD patients: a randomized controlled trial. *Clin Rehabil* 2018;**32**:1636–44.http://ovidsp.ovid.com/ovidweb.cgi?T=JS&PAGE=reference&D=med15&NEWS=N&AN=30012033 %25[20180716//

266 Silva BS, Ramos D, Camillo CA, *et al.* Resistance Training With Elastic Tubing Improves Muscle Strength, Exercise Capacity, and Post-Exercise Creatine Kinase Clearance in Subjects With COPD. *Respir Care* 2019;**64**:835–43.http://ovidsp.ovid.com/ovidweb.cgi?T=JS&PAGE=reference&D=med16&NEWS=N&AN=31138728 %25[20190528//

267 Simpson K, Killian K, McCartney N, *et al.* Randomised controlled trial of weightlifting exercise in patients with chronic airflow limitation. *Thorax* 1992;**47**:70–5. doi:10.1136/THX.47.2.70

268 Sivori M, Rhodius E, Kaplan P, *et al.* [Exercise training in chronic obstructive pulmonary disease. Comparative study of aerobic training of lower limbs vs. combination with upper limbs]. *Entren muscular en la Enferm Pulm Obstr Cron Sev Estud Comp del Entren Aerob miembros Infer vs Comb con miembros Super* 1998;**58**:717–27.http://www.ncbi.nlm.nih.gov/pubmed/10347965 (accessed 5 Nov 2018).

269 Spielmanns M, Fuchs-Bergsma C, Winkler A, *et al.* Effects of oxygen supply during training on subjects with COPD who are normoxemic at rest and during exercise: A blinded randomized controlled trial. *Respir Care* 2015;**60**:540–8. doi:10.4187/respcare.03647

270 Spielmanns M, Boeselt T, Gloeckl R, *et al.* Low-Volume Whole-Body Vibration Training Improves Exercise Capacity in Subjects With Mild to Severe COPD. *Respir Care* 2017;**62**:315–23.http://ovidsp.ovid.com/ovidweb.cgi?T=JS&PAGE=reference&D=med14&NEWS=N&AN=27923937 %25[20161206//

271 Spruit MA, Gosselink R, Troosters T, *et al.* Resistance versus endurance training in patients with COPD and peripheral muscle weakness. *Eur Respir J* 2002;**19**:1072–8.http://ovidsp.ovid.com/ovidweb.cgi?T=JS&PAGE=reference&D=med4&NEWS=N&AN=12108859

272 Steiner MC, Barton RL, Singh SJ, *et al.* Nutritional enhancement of exercise performance in chronic obstructive pulmonary disease: a randomised controlled trial. *Thorax* 2003;**58**:745–51.

273 Strijbos JH, Postma DS, van Altena R, *et al.* A comparison between an outpatient hospital-based pulmonary rehabilitation program and a home-care pulmonary rehabilitation program in patients with COPD. A follow-up of 18 months. *Chest* 1996;**109**:366–72.http://ovidsp.ovid.com/ovidweb.cgi?T=JS&PAGE=reference&D=med4&NEWS=N&AN=8620707

274 Stulbarg MS, Carrieri-Kohlman V, Demir-Deviren S, *et al.* Exercise training improves outcomes of a dyspnea self-management program. J. Cardiopulm. Rehabil. 2002;**22**:109–21.

275 Donesky D, Nguyen HQ, Paul SM, *et al.* The affective dimension of dyspnea improves in a dyspnea self-management program with exercise training. *J Pain Symptom Manage* 2014;**47**:757–71. doi:10.1016/j.jpainsymman.2013.05.019

276 Donesky-Cuenco D, Janson S, Neuhaus J, *et al.* Adherence to a home-walking prescription in patients with chronic obstructive pulmonary disease. *Heart Lung* 2007;**36**:348–63.http://ovidsp.ovid.com/ovidweb.cgi?T=JS&PAGE=reference&D=med6&NEWS=N&AN=17845881

277 Tsang AH. *Effectiveness of three strengths of education and exercise on self-efficacy for walking and self-efficacy for managing dyspnea in patients with chronic obstructive pulmonary disease*. 2000.https://search.ebscohost.com/login.aspx?direct=true&AuthType=sso&db=cin20&AN=109875242&site=ehost-live&custid=ns124898

278 Subin, Rao V, Prem V, *et al.* Effect of upper limb, lower limb and combined training on health-related quality of life in COPD. *Lung India* 2010;**27**:4–7.http://ovidsp.ovid.com/ovidweb.cgi?T=JS&PAGE=reference&D=pmnm2&NEWS=N&AN=20539763

279 Sunil Kumar T, Kilani K, Swathi G. Effectiveness of ground walking versus stair climbing on exercise capacity in subjects with moderate chronic obstructive pulmonary disease. *Indian J Physiother Occup Ther* 2020;**14**:209–14.http://www.indianjournals.com/ijor.aspx?target=ijor:ijpot&type=home http://ovidsp.ovid.com/ovidweb.cgi?T=JS&PAGE=reference&D=emca3&NEWS=N&AN=2004935532

280 Suresh Babu Reddy A, Srinivasan NM, Anil Kumar T, *et al.* Effect of balance training on health related quality of life in patients with chronic obstructive pulmonary disease (COPD). *Indian J Public Heal Res Dev* 2020;**11**:703–9.http://medicopublication.com/index.php/ijphrd http://ovidsp.ovid.com/ovidweb.cgi?T=JS&PAGE=reference&D=emed22&NEWS=N&AN=2004452466

281 Suresh Babu Reddy A, Srinivasan NM, Anil Kumar T, *et al.* The effect of balance training and conventional pulmonary rehabilitation in patients with moderate chronic obstructive pulmonary disease. *Int J Res Pharm Sci* 2021;**12**:757–67.https://pharmascope.org/ijrps/article/view/4179/11168 http://ovidsp.ovid.com/ovidweb.cgi?T=JS&PAGE=reference&D=emed22&NEWS=N&AN=2006002383

282 Sutanto YS, Makhabah DN, Aphridasari J, *et al.* Videogame assisted exercise training in patients with chronic obstructive pulmonary disease: A preliminary study. *Pulmonology* 2019;**25**:275–82.http://ovidsp.ovid.com/ovidweb.cgi?T=JS&PAGE=reference&D=med16&NEWS=N&AN=31076287 %25[20190507//

283 Sykes K, Hang H. Inspiratory muscle training in the treatment of chronic obstructive pulmonary disease: randomized controlled trial. *Am J Recreat Ther* 2005;**4**:39–48.

284 Tabak M, Brusse-Keizer M, van der Valk P, *et al.* A telehealth program for self-management of COPD exacerbations and promotion of an active lifestyle: A pilot randomized controlled trial. *Int J COPD* 2014;**9**:935–44. doi:10.2147/COPD.S60179

285 Tabka O, Sanaa I, Mekki M, *et al.* Effect of a pulmonary rehabilitation program combined with cognitive training on exercise tolerance and cognitive functions among Tunisian male patients with chronic obstructive pulmonary disease: A randomized controlled trial. *Chron Respir Dis* 2023;**20**:14799731231201644. doi:https://dx.doi.org/10.1177/14799731231201643

286 Takahashi H, Sugawara K, Kashiwagura T, *et al.* Effects of low-intensity exercise training (COPD sitting calisthenics) in patients with stable COPD. *Am J Respir Crit Care Med* 2014;**189**.http://www.atsjournals.org/doi/pdf/10.1164/ajrccm-conference.2014.189.1_MeetingAbstracts.A1797 http://ovidsp.ovid.com/ovidweb.cgi?T=JS&PAGE=reference&D=emed15&NEWS=N&AN=72044246

287 Tasdemir F, Inal-Ince D, Ergun P, *et al.* Neuromuscular electrical stimulation as an adjunct to endurance and resistance training during pulmonary rehabilitation in stable chronic obstructive pulmonary disease [abstract]. *Expert Rev Respir Med* 2015;**9**:2015. doi:10.1586/17476348.2015.1068691

288 Tavanaei Youssefian F, Pourghaznein T, Amini M, *et al.* The effect of lung rehabilitation at home and hospital on the distance traveled in 6 minutes in patients with chronic obstructive pulmonary disease. *J Zanjan Univ Med Sci Heal Serv* 2018;**26**:35–45.http://zums.ac.ir/journal/article-1-5048-en.pdf http://ovidsp.ovid.com/ovidweb.cgi?T=JS&PAGE=reference&D=emed19&NEWS=N&AN=620587108 (accessed 13 Nov 2023).

289 Thabitha P, Madhavi K, Charan K, *et al.* Effect of peripheral muscle strength training on exercise capacity in subjects with chronic obstructive pulmonary disease. *Indian J Physiother Occup Ther* 2012;**6**:91–5.http://www.indianjournals.com/ijor.aspx?target=ijor:ijpot&volume=6&issue=1&article=023&type=pdf http://ovidsp.ovid.com/ovidweb.cgi?T=JS&PAGE=reference&D=emca2&NEWS=N&AN=364986221

290 Theander K, Jakobsson P, Jorgensen N, *et al.* Effects of pulmonary rehabilitation on fatigue, functional status and health perceptions in patients with chronic obstructive pulmonary disease: a randomized controlled trial. *Clin Rehabil* 2009;**23**:125–36. doi:10.1177/0269215508096174

291 Titova ON, Ignat’ev VA, Didur MD, *et al.* [Combination of thiotropium bromide with almitrine and pulmonary rehabilitation in the treatment of patients with chronic obstructive pulmonary disease]. *Ter Arkh* 2008;**80**:28–33.http://ovidsp.ovid.com/ovidweb.cgi?T=JS&PAGE=reference&D=med7&NEWS=N&AN=18441680

292 Titova ON, Ignat’ev VA, Didur MD, *et al.* [Effect of tiotropium bromide on exercise tolerance in patients with chronic obstructive pulmonary disease]. *Vopr Kurortol Fizioter Lech Fiz Kult* 2008;:17–20.http://ovidsp.ovid.com/ovidweb.cgi?T=JS&PAGE=reference&D=med7&NEWS=N&AN=18822482

293 Toledo A, Borghi-Silva A, Sampaio LMM, *et al.* The impact of noninvasive ventilation during the physical training in patients with moderate-to-severe chronic obstructive pulmonary disease (COPD). *Clinics (Sao Paulo)* 2007;**62**:113–20.

294 Tounsi B, Acheche A, Lelard T, *et al.* Effects of specific inspiratory muscle training combined with whole-body endurance training program on balance in COPD patients: Randomized controlled trial. *PLoS One* 2021;**16**:e0257595.http://ovidsp.ovid.com/ovidweb.cgi?T=JS&PAGE=reference&D=med19&NEWS=N&AN=34555068 %25[20210923//

295 Troosters T, Gosselink R, Decramer M. Short- and long-term effects of outpatient rehabilitation in patients with chronic obstructive pulmonary disease: a randomized trial. *Am J Med* 2000;**109**:207–12. doi:10.1016/S0002-9343(00)00472-1

296 Tsai LLY, McNamara RJ, Moddel C, *et al.* Home-based telerehabilitation via real-time videoconferencing improves endurance exercise capacity in patients with COPD: The randomized controlled TeleR Study. *Respirology* 2017;**22**:699–707.http://ovidsp.ovid.com/ovidweb.cgi?T=JS&PAGE=reference&D=med14&NEWS=N&AN=27992099 %25[20161219//

297 Turnip H, Ratnawati A, Tulaar A, *et al.* Comparison of the effects of treadmill and ergocycle exercise on the functional capacity and quality of life of patients with chronic obstructive pulmonary disease. *Med J Indones* 2014;**23**:42–7. doi:10.13181/MJI.V23I1.726

298 Valderramas SR, Atallah AN. Effectiveness and safety of hypertonic saline inhalation combined with exercise training in patients with chronic obstructive pulmonary disease: a randomized trial. *Respir Care* 2009;**54**:327–33.

299 Valenza MC, Torres-Sanchez I, Lopez-Lopez L, *et al.* Effects of home-based neuromuscular electrical stimulation in severe chronic obstructive pulmonary disease patients: a randomized controlled clinical trial. *Eur J Phys Rehabil Med* 2018;**54**:323–32.http://ovidsp.ovid.com/ovidweb.cgi?T=JS&PAGE=reference&D=med15&NEWS=N&AN=29144103 %25[20171116//

300 Vallet G, Varray A, Fontaine JL, *et al.* Interest of individualized training program at the ventilatory threshold in mild to moderate COPD patients. *Rev Mal Respir* 1994;**11**:493–501.http://ovidsp.ovid.com/ovidweb.cgi?T=JS&PAGE=reference&D=emed5&NEWS=N&AN=24376072

301 Vallet G, Ahmaïdi S, Serres I, *et al.* Comparison of two training programmes in chronic airway limitation patients: Standardized versus individualized protocols. *Eur Respir J* 1997;**10**:114–22. doi:10.1183/09031936.97.10010114

302 van Gestel AJR, Kohler M, Steier J, *et al.* The effects of controlled breathing during pulmonary rehabilitation in patients with COPD. *Respiration* 2012;**83**:115–24. doi:10.1159/000324449

303 van Wetering CR, Hoogendoorn M, Mol SJMM, *et al.* Short- and long-term efficacy of a community-based COPD management programme in less advanced COPD: A randomised controlled trial. *Thorax* 2010;**65**:7–13. doi:10.1136/thx.2009.118620

304 Varga J, Boda K, Porszasz J, *et al.* Effectiveness of supervised high intensity continuous and interval training compared with self-controlled exercise training in COPD. *Proc Am Thorac Soc* 2006;:A813 [Poster 920].https://www.cochranelibrary.com/central/doi/10.1002/central/CN-00591877/full

305 Vasilopoulou M, Papaioannou AI, Kaltsakas G, *et al.* Home-based maintenance tele-rehabilitation reduces the risk for acute exacerbations of COPD, hospitalisations and emergency department visits. *Eur Respir J* 2017;**49**.http://ovidsp.ovid.com/ovidweb.cgi?T=JS&PAGE=reference&D=med14&NEWS=N&AN=28546268 %25[20170525//

306 Vivodtzev I, Debigare R, Gagnon P, *et al.* Functional and muscular effects of neuromuscular electrical stimulation in patients with severe COPD: a randomized clinical trial. *Chest* 2012;**141**:716–25. doi:10.1378/chest.11-0839

307 Vogiatzis I, Nanas S, Roussos C. Interval training as an alternative modality to continuous exercise in patients with COPD. *Eur Respir J* 2002;**20**:12–9.http://ovidsp.ovid.com/ovidweb.cgi?T=JS&PAGE=reference&D=med4&NEWS=N&AN=12166558

308 Vogiatzis I, Terzis G, Nanas S, *et al.* Skeletal muscle adaptations to interval training in patients with advanced COPD. *Chest* 2005;**128**:3838–45. doi:10.1378/chest.128.6.3838

309 Vonbank K, Strasser B, Mondrzyk J, *et al.* Strength training increases maximum working capacity in patients with COPD--randomized clinical trial comparing three training modalities. *Respir Med* 2012;**106**:557‐563. doi:10.1016/j.rmed.2011.11.005

310 Wada JT, Borges-Santos E, Porras DC, *et al.* Effects of aerobic training combined with respiratory muscle stretching on the functional exercise capacity and thoracoabdominal kinematics in patients with COPD: a randomized and controlled trial. *Int J Chron Obstruct Pulmon Dis* 2016;**11**:2691–700.http://ovidsp.ovid.com/ovidweb.cgi?T=JS&PAGE=reference&D=med13&NEWS=N&AN=27822031 %25[20161028//

311 Wadell K, Sundelin G, Henriksson-Larsen K, *et al.* High intensity physical group training in water--an effective training modality for patients with COPD. *Respir Med* 2004;**98**:428–38.http://ovidsp.ovid.com/ovidweb.cgi?T=JS&PAGE=reference&D=med5&NEWS=N&AN=15139572

312 Wadell K, Webb KA, Preston ME, *et al.* Impact of pulmonary rehabilitation on the major dimensions of dyspnea in COPD. *COPD* 2013;**10**:425–35. doi:10.3109/15412555.2012.758696

313 Wang K, Zeng GQ, Li R, *et al.* Cycle ergometer and inspiratory muscle training offer modest benefit compared with cycle ergometer alone: A comprehensive assessment in stable COPD patients. *Int J COPD* 2017;**12**:2655–68. doi:10.2147/COPD.S140093

314 Wang L, Wu K, Chen X, *et al.* The Effects of Tai Chi on Lung Function, Exercise Capacity and Health Related Quality of Life for Patients With Chronic Obstructive Pulmonary Disease: A Pilot Study. *Heart Lung Circ* 2019;**28**:1206–12.http://ovidsp.ovid.com/ovidweb.cgi?T=JS&PAGE=reference&D=med16&NEWS=N&AN=30166260 %25[20180802//

315 Wang J, Peng J, Zhang F. Effects of different intensity limb linkage training under the guidance of cardio-pulmonary exercise test on individualized cardiopulmonary function in patients with stable chronic obstructive pulmonary disease. *Chinese J Rehabil Med* 2023;**38**:954–60. doi:https://dx.doi.org/10.3969/j.issn.1001-1242.2023.07.014

316 Wanke T, Formanek D, Lahrmann H, *et al.* Effects of combined inspiratory muscle and cycle ergometer training on exercise performance in patients with COPD. *Eur Respir J* 1994;**7**:2205–11. doi:10.1183/09031936.94.07122205

317 Waterhouse JC, Walters SJ, Oluboyede Y, *et al.* A randomised 2 x 2 trial of community versus hospital pulmonary rehabilitation, followed by telephone or conventional follow-up. *Health Technol Assess* 2010;**14**:i–v, vii–xi, 1–140. doi:10.3310/hta14060

318 Garrod R, Bestall JC, Garnham R, *et al.* Randomised controlled trial of pulmonary rehabilitation in moderate and severe COPD: prolonged benefits. *Am J Respir Crit Care Med* 1998;**157**:A257.https://www.cochranelibrary.com/central/doi/10.1002/central/CN-00428238/full

319 Bestall JC, Paul EA, Garrod R, *et al.* Longitudinal trends in exercise capacity and health status after pulmonary rehabilitation in patients with COPD. *Respir Med* 2003;**97**:173–80.

320 Weiner P, Azgad Y, Ganam R. Inspiratory muscle training combined with general exercise reconditioning in patients with COPD. *Chest* 1992;**102**:1351–6. doi:10.1378/chest.102.5.1351

321 Wen H, Gao Y, An J-Y. [Comparison of high-intensity and anaerobic threshold programs in rehabilitation for patients with moderate to severe chronic obstructive pulmonary disease]. *Zhonghua Jie He He Hu Xi Za Zhi* 2008;**31**:571–6.http://www.ncbi.nlm.nih.gov/pubmed/19080398 (accessed 29 Oct 2018).

322 White RJ, Rudkin ST, Harrison ST, *et al.* Pulmonary rehabilitation compared with brief advice given for severe chronic obstructive pulmonary disease. J. Cardiopulm. Rehabil. 2002;**22**:338–44.

323 Whitsel LP. *Nutritional supplementation and exercise rehabilitation in chronic obstructive pulmonary disease*. 1998.https://search.ebscohost.com/login.aspx?direct=true&AuthType=sso&db=cin20&AN=109842038&site=ehost-live&custid=ns124898

324 Widyastuti K, Makhabah DN, Setijadi AR, *et al.* Benefits and costs of home pedometer assisted physical activity in patients with COPD. A preliminary randomized controlled trial. *Pulmonology* 2018;**24**:211–8. doi:10.1016/j.pulmoe.2018.01.006

325 Wijkstra PJ, Van Der Mark Th W, Kraan J, *et al.* Effects of home rehabilitation on physical performance in patients with chronic obstructive pulmonary disease (COPD). *Eur Respir J* 1996;**9**:104–10.http://ovidsp.ovid.com/ovidweb.cgi?T=JS&PAGE=reference&D=emed6&NEWS=N&AN=26037533

326 Wittmann M, Spohn S, Schultz K, *et al.* [Patient education in COPD during inpatient rehabilitation improves quality of life and morbidity]. *COPD-Schulung im Rahmen der Station Rehabil verbessert Leb und Morb* 2007;**61**:636–42.http://ovidsp.ovid.com/ovidweb.cgi?T=JS&PAGE=reference&D=med6&NEWS=N&AN=17886195 %25[20070920//

327 Spohn S, Wittmann M, Petro W. Impact of an education program on health-related control beliefs and self-efficacy expectancies in patients with COPD. *Pravention und Rehabil* 2002;**14**:163–70.http://ovidsp.ovid.com/ovidweb.cgi?T=JS&PAGE=reference&D=emed7&NEWS=N&AN=36004814

328 Wiyono WH, Riyadi J, Yunus F, *et al.* The benefit of pulmonary rehabilitation against quality of life alteration and functional capacity of chronic obstructive pulmonary disease (COPD) patient assessed using St George’s respiratory questionnaire (SGRQ) and 6 minutes walking distance test (6MW. *Med J Indones* 2006;**15**:165–72. doi:10.13181/MJI.V15I3.232

329 Wootton SL, Cindy Ng LW, McKeough ZJ, *et al.* Ground-based walking training improves quality of life and exercise capacity in COPD. *Eur Respir J* 2014;**44**:885–94. doi:10.1183/09031936.00078014

330 Wootton SL, Hill K, Alison JA, *et al.* Effects of ground-based walking training on daily physical activity in people with COPD: A randomised controlled trial. *Respir Med* 2017;**132**:139–45.http://ovidsp.ovid.com/ovidweb.cgi?T=JS&PAGE=reference&D=med14&NEWS=N&AN=29229086 %25[20171012//

331 Wright PR, Heck H, Langenkamp H. Effects of a resistance training on pulmonary function and performance measurements in patients with chronic obstructive pulmonary disease. *Eur J Sport Sci* 2003;**3**:1–10.http://www.tandf.co.uk/journals/journal.asp?issn=1746-1391&linktype=1 http://ovidsp.ovid.com/ovidweb.cgi?T=JS&PAGE=reference&D=emca1&NEWS=N&AN=370453696

332 Wu H, Sun X, Gu W, *et al.* [Clinical effects of sub-maximum ergometry exercise rehabilitation in patients with chronic obstructive pulmonary disease]. *Zhongguo Ying Yong Sheng Li Xue Za Zhi* 2015;**31**:382–4.http://ovidsp.ovid.com/ovidweb.cgi?T=JS&PAGE=reference&D=med12&NEWS=N&AN=26775516

333 Wu M, Zhou L-Q, Li S, *et al.* Efficacy of patients’ preferred exercise modalities in chronic obstructive pulmonary disease: A parallel-group, randomized, clinical trial. *Clin Respir J* 2018;**12**:1581–90.http://ovidsp.ovid.com/ovidweb.cgi?T=JS&PAGE=reference&D=med15&NEWS=N&AN=28925009 %25[20171012//

334 Wu W, Liu X, Li P, *et al.* Effect of Liuzijue Exercise Combined with Elastic Band Resistance Exercise on Patients with COPD: A Randomized Controlled Trial. *Evid Based Complement Alternat Med* 2018;**2018**:2361962.http://ovidsp.ovid.com/ovidweb.cgi?T=JS&PAGE=reference&D=pmnm4&NEWS=N&AN=29991952 %25[20180611//

335 Wurtemberger G, Bastian K. [Functional effects of different training in patients with COPD]. *Funktionelle Eff unterschiedlicher Trainingsformen bei Patienten mit COPD* 2001;**55**:553–62.http://ovidsp.ovid.com/ovidweb.cgi?T=JS&PAGE=reference&D=med4&NEWS=N&AN=11748505

336 Xi F, Wang Z, Qi Y, *et al.* Long-term effect of respiratory training for chronic obstructive pulmonary disease patients at an outpatient clinic: A randomised controlled trial. *Clin Transl Med* 2015;**4**:31.http://www.clintransmed.com/ http://ovidsp.ovid.com/ovidweb.cgi?T=JS&PAGE=reference&D=emed16&NEWS=N&AN=606362444

337 Xiao CM, Zhuang YC. Efficacy of liuzijue qigong in individuals with chronic obstructive pulmonary disease in remission. *J Am Geriatr Soc* 2015;**63**:1420–5. doi:10.1111/jgs.13478

338 Xu Y, Yang D, Lu B, *et al.* Efficacy of aerobic training and resistance training combined with external diaphragm pacing in patients with chronic obstructive pulmonary disease: A randomized controlled study. *Clin Rehabil* 2023;**37**:1479–91. doi:https://dx.doi.org/10.1177/02692155231172005

339 Yeh GY, Litrownik D, Wayne PM, *et al.* BEAM study (Breathing, Education, Awareness, Movement): a randomised controlled feasibility trial of tai chi exercise in patients with COPD. *BMJ open Respir Res* 2020;**7**. doi:10.1136/BMJRESP-2020-000697

340 Yekefallah L, Zohal MA, Keshavarzsarkar O, *et al.* Comparing the effects of upper limb and breathing exercises on six-minute walking distance among patients with chronic obstructive pulmonary disease: a three-group randomized controlled clinical trial. *Adv Respir Med* 2019;**87**:77–82.http://ovidsp.ovid.com/ovidweb.cgi?T=JS&PAGE=reference&D=med16&NEWS=N&AN=31038717

341 Yudhawati R, Rasjid Hs M. Effect of yoga on FEV1, 6-minute walk distance (6-MWD) and quality of life in patients with COPD group B. *Adv Respir Med* 2019;**87**:261–8.http://ovidsp.ovid.com/ovidweb.cgi?T=JS&PAGE=reference&D=med16&NEWS=N&AN=31680225

342 Zambom-Ferraresi F, Cebollero P, Gorostiaga EM, *et al.* Effects of combined resistance and endurance training versus resistance training alone on strength, exercise capacity, and quality of life in patients with COPD. *J Cardiopulm Rehabil Prev* 2015;**35**:446–53. doi:10.1097/HCR.0000000000000132

343 Zanini A, Aiello M, Cherubino F, *et al.* The one repetition maximum test and the sit-to-stand test in the assessment of a specific pulmonary rehabilitation program on peripheral muscle strength in COPD patients. *Int J COPD* 2015;**10**:2423–30. doi:10.2147/COPD.S91176

344 Zanotti E, Berardinelli P, Bizzarri C, *et al.* Osteopathic manipulative treatment effectiveness in severe chronic obstructive pulmonary disease: a pilot study. *Complement Ther Med* 2012;**20**:16–22. doi:10.1016/j.ctim.2011.10.008

345 Zanotti E, Bizzarri C, Grasso R. Combination of Pulmonary Rehabilitation and Neuromuscular Electrical Stimulation in COPD Patients: A Randomized Clinical Trial of Efficacy. *J Pulm Respir Med* 2012;**02**. doi:10.4172/2161-105X.1000112

346 Zhang LH, Wu JJ, Wang ZC. Effect of 24-form tai chi with respiratory rehabilitation training on pulmonary function and quality of life of patients with COPD. *Acta Univ Tradit medicalis Sin Pharmacol shanghai [ shanghai zhong yi yao da xue xue bao]* 2012;**26**:53‐56.https://www.cochranelibrary.com/central/doi/10.1002/central/CN-01156481/full

347 Zhang M, Xv G, Luo C, *et al.* Qigong Yi Jinjing Promotes Pulmonary Function, Physical Activity, Quality of Life and Emotion Regulation Self-Efficacy in Patients with Chronic Obstructive Pulmonary Disease: A Pilot Study. *J Altern Complement Med* 2016;**22**:810–7.http://ovidsp.ovid.com/ovidweb.cgi?T=JS&PAGE=reference&D=med13&NEWS=N&AN=27487437 %25[20160803//

348 Zhang H-L, Li J-S, Yu X-Q, *et al.* An evaluation of activity tolerance, patient-reported outcomes and satisfaction with the effectiveness of pulmonary daoyin on patients with chronic obstructive pulmonary disease. *Int J Chron Obstruct Pulmon Dis* 2017;**12**:2333–42.http://ovidsp.ovid.com/ovidweb.cgi?T=JS&PAGE=reference&D=med14&NEWS=N&AN=28831250 %25[20170804//

349 Zhang JH, Zhang LQ, Yang YP, *et al.* [Clinical effect of nutritional and psychological intervention combined with pulmonary rehabilitation exercise on patients with chronic obstructive pulmonary disease]. *Zhonghua Yi Xue Za Zhi* 2020;**100**:110–5.http://ovidsp.ovid.com/ovidweb.cgi?T=JS&PAGE=reference&D=med17&NEWS=N&AN=31937049

350 Zhuang M, Liu X, Li P, *et al.* Effects and mechanism of pulmonary-based Qigong exercise on cognitive function in patients with stable chronic obstructive pulmonary disease. *Chinese J Rehabil Med* 2023;**38**:904–10. doi:https://dx.doi.org/10.3969/j.issn.1001-1242.2023.07.006

351 陆绍勇, 盛艺璇, 韩松, *et al.* 机械性渐进抗阻呼吸肌训练对慢性阻塞性肺疾病 患者呼吸及运动功能的影响. *Chinese J Conval Med / Zhongguo Liaoyang Yixue* 2022;**31**:824–8. doi:10.13517/j.cnki.ccm.2022.08.009

352 Rosenstein B, Smyrnova A, Rizk A, *et al.* Short- and long-term changes in cognitive function after exercise-based rehabilitation in people with COPD: A pilot study. *Can J Respir Crit Care, Sleep Med* 2021;**5**:300–9.https://www.tandfonline.com/toc/ucts20/current http://ovidsp.ovid.com/ovidweb.cgi?T=JS&PAGE=reference&D=emexb&NEWS=N&AN=2005966572

353 Chan AWK, Lee A, Lee DTF, *et al.* Evaluation of the sustaining effects of Tai Chi Qigong in the sixth month in promoting psychosocial health in COPD patients: a single-blind, randomized controlled trial. *ScientificWorldJournal* 2013;**2013**:425082. doi:10.1155/2013/425082

354 Chan AWK, Lee A, Lee DTF, *et al.* The sustaining effects of Tai chi Qigong on physiological health for COPD patients: a randomized controlled trial. *Complement Ther Med* 2013;**21**:585–94. doi:10.1016/j.ctim.2013.09.008

355 He Y, Li G yuan, Tang C zhi, *et al.* Comparing penetrating needles and non-penetrating needles with electrical stimulation combined with exercise training for relief of dyspnea and improving exercise tolerance in chronic obstructive pulmonary disease patients: A single-blind randomized controlled trial. *Integr Med Res* 2025;**14**. doi:10.1016/j.imr.2024.101117

356 Zhu Z, Muhamad AS, Omar N, *et al.* Effects of Mawangdui exercise intervention on the pulmonary function, physical fitness and quality of life in stable chronic obstructive pulmonary disease patients: A randomised controlled trial. *Complement Ther Med* 2025;**89**. doi:https://dx.doi.org/10.1016/j.ctim.2025.103152

357 Jin S, Huang B, Kong Y, *et al.* Effect of neuromuscular electrical stimulation combined with respiratory rehabilitation training on pulmonary rehabilitation in patients with chronic obstructive pulmonary disease. *J Cardiothorac Surg* 2025;**20**. doi:https://dx.doi.org/10.1186/s13019-024-03329-y

358 Phantayuth D, Chuaychoo B, Supaporn S, *et al.* Effectiveness of a 12-week combining tai chi and yoga program on pulmonary function and functional fitness in COPD patients. *Respir Med* 2024;**234**. doi:https://dx.doi.org/10.1016/j.rmed.2024.107842

359 Chen Y, Zhang P, Dong Z, *et al.* Effect of Baduanjin exercise on health and functional status in patients with chronic obstructive pulmonary disease: a community-based, cluster-randomized controlled trial. *NPJ Prim care Respir Med* 2024;**34**. doi:https://dx.doi.org/10.1038/s41533-024-00400-y

360 Cui S, Ji H, Li L, *et al.* Effects and long-term outcomes of endurance versus resistance training as an adjunct to standard medication in patients with stable COPD: a multicenter randomized trial. *BMC Pulm Med* 2024;**24**. doi:https://dx.doi.org/10.1186/s12890-024-03010-z

361 Viana SM do NR, de Bruin VMS, Vasconcelos RS, *et al.* Melatonin supplementation enhances pulmonary rehabilitation outcomes in COPD: a randomized, double-blind, placebo-controlled study. *Respir Med* 2023;**220**. doi:https://dx.doi.org/10.1016/j.rmed.2023.107441

362 Wang X, Lu J, Niu J, *et al.* Effectiveness of high-intensity interval training in rehabilitation nursing for mild-to-moderate stable COPD patients: a randomized controlled clinical trial. *BMC Sport Sci Med Rehabil* 2025;**17**. doi:https://dx.doi.org/10.1186/s13102-025-01074-w

363 Nolasco T, Figueiredo R, Zanella P, *et al.* Combined Physical Exercise in Pulmonary Rehabilitation Does Not Alter Endothelial Function and Vascular Structure in Chronic Obstructive Pulmonary Disease: A Randomized Clinical Trial. *J Cardiopulm Rehabil Prev* 2025;**45**. doi:https://dx.doi.org/10.1097/HCR.0000000000000940

364 Engel RM, Gonski P, Vemulpad S, *et al.* The Long-Term Benefit of Exercise With and Without Manual Therapy for Mild Chronic Obstructive Pulmonary Disease: A Randomized Controlled Trial. *J Cardiopulm Rehabil Prev* 2024;**44**. doi:https://dx.doi.org/10.1097/HCR.0000000000000871

365 Jiang L, Li P, Shi J, *et al.* Effects of pulmonary-based Qigong exercise in stable patients with chronic obstructive pulmonary disease: a randomized controlled trial. *BMC Complement Med Ther* 2023;**23**. doi:10.1186/S12906-023-04238-8

366 Ito JT, Alves LH V, Oliveira LM, *et al.* Effect of exercise training on modulating the TH17/TREG imbalance in individuals with severe COPD: a randomized controlled trial. 2025;**31**. doi:10.1080/25310429.2024.2441069

367 Sevasta K, Agius TP, Sciriha A. Short term effects of MET programme in patients diagnosed with Chronic Obstructive Pulmonary Disease: a randomised controlled trial. *Eur J Physiother* 2025;**27**. doi:10.1080/21679169.2024.2310496

368 Gallo-Silva B, Cerezer-Silva V, Gullo Ferreira D, *et al.* Effect of water exercise on the respiratory function and functional capacity of patients with COPD: a randomized controlled trial. *Fisioter em Mov* 2024;**37**. doi:10.1590/fm.2024.37121

369 Zong M, Shen H, Ren L, *et al.* Effects of whey protein complex combined with low-intensity exercise in elderly inpatients with COPD at a stable stage. 2023;**32**. doi:10.6133/apjcn.202312_32(4).0001

370 Williams MT, Lewthwaite H, Paquet C, *et al.* Pulmonary Rehabilitation with and without a Cognitive Behavioral Intervention for Breathlessness in People Living with Chronic Obstructive Pulmonary Disease: randomized Controlled Trial. 2023;**12**. doi:10.3390/jcm12237286

371 Lee AL, Butler SJ, Jung P, *et al.* Participant-selected music listening during pulmonary rehabilitation in people with chronic obstructive pulmonary disease: a randomised controlled trial. 2024;**21**. doi:10.1177/14799731241291065

372 da Silva MMC, Ferreira Arcuri J, Viana DR, *et al.* Responders COPD patients to two different home-based rehabilitation programs: a blind, randomized, and controlled clinical trial. Published Online First: 2024. doi:10.1080/09638288.2024.2439574

373 Chen X, Fu C, Wang X, *et al.* Combined Effect of 12 Weeks Baduanjin and Tri-Ball Respiratory Training as a Home-Based Pulmonary Rehabilitation in Subjects With Moderate Chronic Obstructive Pulmonary Disease: A Multi-Center Randomized Controlled Trial. *Rehabil Nurs* 2025;**50**. doi:10.1097/RNJ.0000000000000493

374 Harvey-Dunstan TC, Baldwin MM, Tal-Singer R, *et al.* The Responsiveness of Exercise Tests in COPD: a Randomized Controlled Trial. 2025;**167**. doi:10.1016/j.chest.2024.05.051

375 Ataç A, Pehlivan E, Karaahmetoglu FS, *et al.* The Impact of Different Telerehabilitation Methods on Peripheral Muscle Strength and Aerobic Capacity in COPD Patients: a Randomized Controlled Trial. 2024;**92**. doi:10.3390/arm92050035

376 Karaca S, Yildiz Ozer A, Karakurt S, *et al.* Effects of body awareness therapy on balance and fear of falling in patients with chronic obstructive pulmonary disease: a randomized controlled trial. *Biopsychosoc Med* 2024;**18**. doi:https://dx.doi.org/10.1186/s13030-024-00303-x

377 Tsui AYY, Cheing GLY, Chau RMW, *et al.* Benefits of chest wall mobilization on respiratory efficiency and functional exercise capacity in people with severe chronic obstructive pulmonary disease (COPD): A randomized controlled trial. *Respirology* 2025;**30**. doi:https://dx.doi.org/10.1111/resp.14831

378 Bishop JA, Spencer LM, Dwyer TJ, *et al.* Effect of pulmonary rehabilitation duration on exercise capacity and health-related quality of life in people with chronic obstructive pulmonary disease (PuRe Duration Trial): A randomized controlled equivalence trial. *Respirology* 2025;**30**. doi:https://dx.doi.org/10.1111/resp.14820

379 Kohlbrenner D, Kuhn M, Klay A, *et al.* Hybrid Virtual Coaching and Telemonitoring in COPD Management: The CAir Randomised Controlled Study. *Int J Chron Obstruct Pulmon Dis* 2024;**19**. doi:https://dx.doi.org/10.2147/COPD.S487105

380 Jiang Y, Nuerdawulieti B, Chen Z, *et al.* Effectiveness of patient decision aid supported shared decision-making intervention in in-person and virtual hybrid pulmonary rehabilitation in older adults with chronic obstructive pulmonary disease: A pilot randomized controlled trial. *J Telemed Telecare* 2023;**30**:1357633X231156631. doi:https://dx.doi.org/10.1177/1357633X231156631

381 Kochamat A, Harnirattisai T, Juratovac E. The effects of a symptom management program on symptom experience and physical function in Thai adults with chronic obstructive pulmonary disease: A single-blind randomized controlled trial study. *Belitung Nurs J* 2024;**10**. doi:https://dx.doi.org/10.33546/bnj.3393

382 Gloeckl R, Spielmanns M, Stankeviciene A, *et al.* Smartphone application-based pulmonary rehabilitation in COPD: a multicentre randomised controlled trial. *Thorax* 2025;**80**. doi:https://dx.doi.org/10.1136/thorax-2024-221803

383 Zhang Y, Chen G, Xu C, *et al.* Efficacy of online pulmonary rehabilitation management among community‑dwelling patients with stable chronic obstructive pulmonary disease. 2024;**23**. doi:10.3760/cma.j.cn114798-20231107-00359

384 Rath J, Sahoo S. Optimizing pulmonary rehabilitation: the role of structured exercise interventions in enhancing functional and physiological outcomes in chronic obstructive pulmonary disease. 2025;**14**. doi:10.69605/ijlbpr_14.1.2025.120

385 Na C, Fei L. INFLUENCES OF AEROBIC EXERCISE ASSOCIATED WITH THE PHARMACOLOGICAL TREATMENT OF COPD. *Rev Bras Med do Esporte* 2023;**29**:e2023_0067. doi:https://dx.doi.org/10.1590/1517-8692202329012023_0067

386 Thokchom SK, Ray A, Menon BK, *et al.* A Clinical Study to Evaluate the Effects of Yogic Intervention on Pulmonary Functions, Inflammatory Marker and Health Status in Patients of Chronic Obstructive Pulmonary Disease. 2021;**53**.https://www.cochranelibrary.com/central/doi/10.1002/central/CN-02618898/full

387 Dua R, Malik S, Bhadoria AS, *et al.* Effectiveness of Telemedicine Interventions in Chronic Obstructive Pulmonary Disease (COPD) Management: A Randomized Controlled Trial Comparing Yoga Therapy and Pulmonary Rehabilitation Over Three Months. *Cureus* 2024;**16**. doi:https://dx.doi.org/10.7759/cureus.56060

388 B. Z, Y. L, M. L. Research on the effect of home-based pulmonary rehabilitation in patients with moderate chronic obstructive pulmonary disease. *Chinese J Rehabil Med* 2025;**40**. doi:https://dx.doi.org/10.3969/j.issn.1001-1242.2025.02.008

389 D. W, Z. Y, X. C, *et al.* Efficacy of remote home-monitored Baduanjin in patients with chronic obstructive pulmonary disease. *Chinese J Rehabil Med* 2024;**39**. doi:https://dx.doi.org/10.3969/j.issn.1001-1242.2024.05.003
